# Evolution of the global terrorist organizational cooperation network

**DOI:** 10.1371/journal.pone.0281615

**Published:** 2024-01-22

**Authors:** Donghao Cui, Chaomin Ou, Xin Lu

**Affiliations:** College of Systems Engineering, National University of Defense Technology, Changsha, China; East China Normal University, CHINA

## Abstract

Terrorism has shown a trend of organizational cooperation in a large number of terrorist attacks around the world, posting a great challenge to counter-terrorism efforts. To investigate the trend and pattern of global terrorist organizational cooperations and to propose effective measures for effectively enforcing and restricting terrorist attacks, based on the Global Terrorism Database and the UN sanctions list of terrorist groups, this study constructs a cooperative evolutionary network of terrorist organizations from 119,803 terrorist attacks that occurred globally between 2001 and 2018. The evolution of worldwide terrorist cooperation is evaluated in terms of network characteristics, including key nodes, cohesion, and motifs. The network keeps expanding, with a large number of new nodes emerging each year. On average, there are 13 additional organizations entering in the collaboration network each year, with a yearly survival rate of about 34.66%, and the rank of node importance iterate and update frequently. Through *k*-core decomposition, for which the breakdown of the network has increased from three to five partitions, we find that the core of the terrorist organization’s cooperation network changes much less frequently than the edges. The dominating modal structure of the network is the "star" motif (90%), and "triadic closed" motif (9%). We conclude that, over time, the cooperative network of terrorist groups has gradually evolved into a cluster of star-shaped networks, with various organizations serving as the centers of the networks and showing core-periphery structure in their individual communities. The core organizations are highly connected and stable, whereas the periphery organizations are loosely connected and highly variable.

## 1. Introduction

Terrorism has posed a huge threat to the international safety situation in recent years, with the creation of new terrorist groups, the recurrence of terrorist occurrences, and collusion and coordination among terrorist organizations. Due to the commonality of ideological origins, behavioral patterns, and the pursuit of goals, the forces of terrorist organizations have formed a loose coalition of international terrorist networks [1], a situation which makes it more difficult to counter terrorism and increases the danger it poses to the international security environment. Although most terrorist activities occur in the Middle East and other parts of Asia, the rest of the world is not immune to terrorism [2].

Social network analysis has been a key technique in modeling the structure of terrorist organizations and investigating their behaviors. The contribution of sociology and anthropology to social network analysis is the use of graph theory to visually express the connections between things. Physicists and computer scientists provide significant support for modeling network analysis. On September 11, 2001, the counter-terrorism application of social network analysis was developed when Krebs pioneered social network modeling of terrorists in a series of hijackings by studying [3]. In recent decades, organization-based terrorist acts have been the predominant form of terrorist activity, and terrorist organizations tend to be networked [4]. The question of how to correctly understand the network of cooperation among terrorist organizations and the effective implementation of the strategy of disrupting organizational alliances has attracted the common attention of scholars and security departments at home and abroad [5].

The holistic analysis of terrorist organizations cooperation network is the base of the network evolution analysis. Holistic network analysis mainly focuses on identification of core nodes by measuring the nodal centrality indicators through topological structure. For example, Memon and Larsen analyzed the characteristics of terrorist organization networks based on social network analysis methods, using indicators such as density and cohesion [6]; Chen studied network topological properties to understand terrorist organization networks through organizational structure and structural functions [7]. Rothenberg found that connectivity, network layer redundancy, and structural flexibility are the salient features of terrorist organization networks [8]. Bahgat and Medina found the existence of resources, information, and personnel sharing among terrorist organizations, which in turn forms complex collaborative networks [9]. Zhao and Fang analyzed the alliances among international terrorist organizations and found 22 large and similar terrorist groups in terms of ideology and geographical scope [10].

Dynamic network analysis takes into account the effect of temporal factors and can be used to understand the evolving dynamics of the cooperative network characteristics of terrorist organizations. Chen conducted real-time monitoring of terrorist activities and visualized network relationships by acquiring and processing open-source information [7]. Carley et al. combine traditional social network analysis with artificial intelligence and machine learning for the evolutionary analysis of terrorist organization network structure, terrorist organization performance assessment, and network disintegration strategies [11]. Moon and Carley analyzed the social relationships, and the spatial and temporal evolution of terrorists to infer potential leaders, hotspots, and organizational vulnerabilities [12].

In general, studies on the method of network analysis have shifted from static topological analysis to combined topological and dynamic evolutionary analysis [13]. Previous studies have focused mostly on the characteristics of the overall topology of the network and largely overlooked the evolution of microstructures such as changes of key nodes, motifs or groups in the network, which may reveal more in-depth patterns or mechanisms of cooperation in terrorist organization cooperation networks. To fill in this gap of knowledge, this paper analyzes terrorist organization cooperation networks based on the social network analysis method and establishes a dynamic evolution model of terrorist organization cooperation networks in order to analyze the evolution pattern of terrorist organization cooperation and provide a reference for the formulation of counter-terrorism strategies.

## 2. Data and methods

### 2.1 Data sources

**Fig 1:**
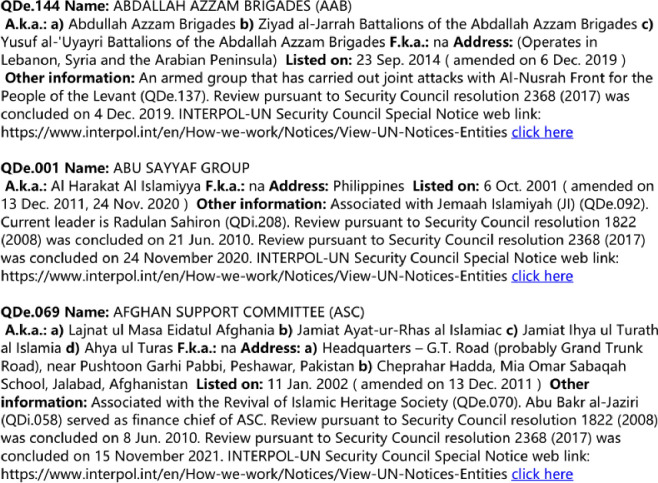
Examples of the United Nations Security Council consolidated list.

The Global Terrorism Database (GTD) is regarded as the most exhaustive database covering terrorist incidents to date in the field of global terrorism research [14]. The entry of any information recorded in GTD is strictly enforced according to a series of guidelines [15]. In this paper, we collected 119,803 terrorist attacks that occurred globally between 2001 and 2018, containing information such as the time of occurrence and the names of the terrorist organizations involved, as shown in Table 1. The sanctions list updated by the UN Security Council Sanctions Committee includes information on terrorist organizations’ origins, leaders, organizational advantages, and cooperative organizations, as shown in Fig 1.

**Table 1:** Sample data in the global terrorism database.

eventid	iyear	imonth	gname	gsubname	gname2
**200101020004**	**2001**	**1**	**Earth Liberation Front (ELF)**	**The Family**	**Animal Liberation Front (ALF)**
**200103300002**	**2001**	**3**	**Earth Liberation Front (ELF)**	**The Family**	**Animal Liberation Front (ALF)**
**200105210006**	**2001**	**5**	**Earth Liberation Front (ELF)**	**The Family**	**Animal Liberation Front (ALF)**
**200105210007**	**2001**	**5**	**Earth Liberation Front (ELF)**	**The Family**	**Animal Liberation Front (ALF)**
**200110150005**	**2001**	**10**	**Earth Liberation Front (ELF)**	**The Family**	**Animal Liberation Front (ALF)**
**200307070001**	**2003**	**7**	**Party for the Liberation of the Hutu People (PALIPEHUTU)**	**Forces for National Liberation (FNL)**	**National Council for Defense of Democracy (NCDD)**
**200307070002**	**2003**	**7**	**Party for the Liberation of the Hutu People (PALIPEHUTU)**	**Forces for National Liberation (FNL)**	**National Council for Defense of Democracy (NCDD)**
**200307070003**	**2003**	**7**	**Party for the Liberation of the Hutu People (PALIPEHUTU)**	**Forces for National Liberation (FNL)**	**National Council for Defense of Democracy (NCDD)**
**200307070004**	**2003**	**7**	**Party for the Liberation of the Hutu People (PALIPEHUTU)**	**Forces for National Liberation (FNL)**	**National Council for Defense of Democracy (NCDD)**
**200307080001**	**2003**	**7**	**Party for the Liberation of the Hutu People (PALIPEHUTU)**	**Forces for National Liberation (FNL)**	**National Council for Defense of Democracy (NCDD)**
**200307090001**	**2003**	**7**	**Party for the Liberation of the Hutu People (PALIPEHUTU)**	**Forces for National Liberation (FNL)**	**National Council for Defense of Democracy (NCDD)**

### 2.2 Network construction

Cooperation between terrorist organizations involves both direct cooperation in launching terrorist attacks and potential cooperation in finance, weapons, manpower, and information. This article also examines various forms of inter-organizational cooperation, which can more properly and exhaustively portray the cooperation ties of terrorist groups. The terrorist organization collaboration network is a weightless and undirected network; in the network diagram, if there is cooperation between two organizations, there is just one connected edge connecting them. Since the time of potential cooperation of terrorist organizations to establish cooperative relations cannot be determined, in order to avoid the influence of uncertainty of the time of potential cooperation to establish cooperative relations, the information of the year when the first cooperative activities with other terrorist organizations launched terrorist attacks and the year when some terrorist organizations were first mentioned in the UN sanctions list in GTD database is used as the basis to determine the time of establishing cooperative relations.

**Fig 2:**
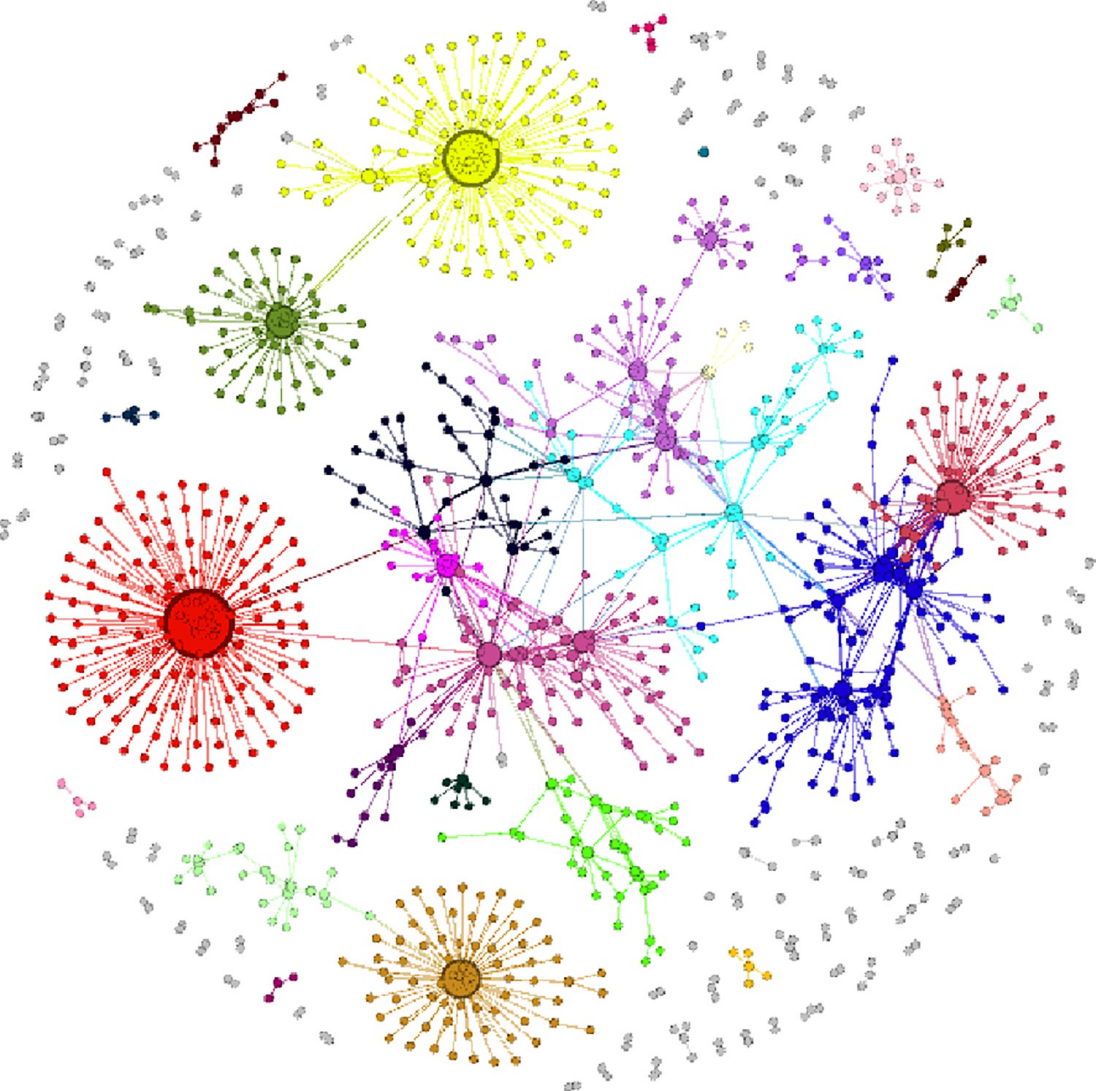
Global terrorist organizational cooperation network, 2001–2018.

Using each organization as a node, if two organizations launch attacks on the same event, it may be assumed that they pursue similar goals and ideologies, indicating that there is an edge between these nodes. In the GTD database, incident number "200101050003" indicates that on May 1, 2001, Hizbul Mujahideen (HM), Lashkar-e-Taiba, and Harkatul Mujahideen attacked the local army in Poonch, India. They killed 9 people and wounded 15 with machine guns and firearms. The coordinated assault demonstrates coordination between Hizbul Mujahideen (HM), Lashkar-e-Taiba, and Harkatul Mujahideen Islam. In addition, the inclusion of information on the exchange of finances, persons, weapons, and bases in the UN list of terrorist organization sanctions suggests coordination between the groups and a connection between the nodes at the edge. The global terrorist organization cooperation network from 2001 to 2018 was established by using SQL server software to clear the GTD data of individual organization events, collating and retaining the data of multiple organization cooperation, and incorporating the cooperation relationship of the UN sanctions list into the data to be added, as shown in Fig 2.

### 2.3 Network metrices

To introduce the basic indicators of the social network analysis method involved in this study, which will help to identify key nodes, discover important local network structures, and study the evolutionary dynamics of global terrorist cooperation networks.

(1) *Degree centrality*. The degree of a node is defined as the number of nodes with which the node has connected edges. Let *k*_*i*_ be the degree of node *i*. The larger the *k*_*i*_, the more neighbors the node can directly influence and the more important the node is. For instance, since the influence of nodes with the same degree value in networks of different sizes may be different, to facilitate comparison, the normalized degree centrality index of node *i* is defined as:

$$DC(i)=\frac{k_{i}}{N-1}$$ (1)

where *N* is the number of nodes in the network and the denominator *N*−1 is the maximum possible degree value of the node.

*(2) Betweenness centrality*. It is considered that among all the shortest paths of all node pairs in the network (in general, there are multiple shortest paths between a pair of nodes), the greater the number of shortest paths through a node, the more important the node is. The betweenness centrality index of node *i* is defined as:

$$BC(i)=\sum_{i\neq s,i\neq t,s\neq t}\frac{g_{st}^{i}}{g_{st}}$$ (2)

Where *g*_*st*_ is the number of all shortest paths from node *s* to *t*, and $g_{st}^{i}$ is the number of paths passing through node *i* among all shortest paths from node *s* to *t*. *BC*(*i*) can be normalized by the largest possible value of the betweenness centrality index in all connected networks of size *N*, i.e., when the network is star-like:

$$BC^{\prime }(i)=\frac{2}{\left(N-1)(N-2\right)}\sum \frac{g_{st}^{i}}{g_{st}}$$ (3)

*(3) k*-core. A *k*-core in a network is a connected maximal induced subgraph which has minimum degree greater than or equal to *k*. *k*-core can be obtained by the remaining sub-graph after all the nodes with degrees ≤*k*−1 have been removed, during which: when a node is removed, the degree of all remaining nodes will be re-calculated and those with a new degree ≤*k*−1 also need to be removed recursively. If a node belongs to a *k*-core of a graph but it will be removed from the (*k*+1)-*core*, then this node is said to have core (core value) *k*. The largest coreness in a graph is referred to as the graph’s coreness. The primary implication of the concept of coreness is that a network with a greater coreness will be more resistant to malicious attacks. Both star-shaped and ring-shaped networks are apparently susceptible to purposeful attacks.

*(4) Motif*. A motif is a subgraph of a network that satisfies the following conditions: (1) The probability that the subgraph appears more frequently in the random network matching to the real network than it does in the real network is minimal, and is typically less than some threshold *p*, such as *p* = 0.01; (2) the number of times the subgraph appears in the real network is not less than some lower limit *U*, e.g., *U* = 4; (3) The number of times *N*_*reali*_ the subgraph appears in the real network is significantly higher than the number of times *N*_*randi*_. It’s generally required that (*N*_*reali*_ – *N*_*randi*_) >0.1*N*_*rand*_. The motif has the following three characteristics:

① Frequency: For a given subgraph with a node, the number of times *N*_*reali*_ it appears in the real network is, and the total number of times all subgraphs with a node appear in the real network is, then the frequency of the subgraph is

$$f(V)=\frac{n(V)}{N}$$ (4)

If the subgraph is a motif of the network, the frequency is called the frequency of the motif.

② P-value: The probability that the number of times it appears in the random network is greater than the number of times it appears in the real network is the P-value of the motif *M*. The smaller the P-value, the more important the motif is in the network, and if the P-value is higher than the threshold of 0.01, the corresponding subgraph is not a motif.

③ Z-score: For a motif *Mi*, if the number of occurrences in the real network is *N*_*reali*_, the number of occurrences in the random network is *N*_*randi*_, the mean of *N*_*randi*_ is <*N*_*randi*_>, and the standard deviation is *σ*_*randi*_, then the Z-score of the modal in the real network is:

$$Z_{i}=\frac{N_{reali}-<N_{randi}>}{\sigma_{randi}}$$ (5)

And to normalize, there is

$${\mathrm{SP}}_{i}=\frac{Z_{i}}{\sqrt{\sum_{i}{Z_{i}}^{2}}}$$ (6)

The Z-score is used to measure the importance of the motif, and a larger Z-value indicates that the motif is more important in the network. The Z-values are normalized to emphasize the relative importance of the subgraphs. Due to the fact that the Z-score of the motif in large networks is greater than the Z-score of the motif in small networks, normalization allows us to compare networks of different scales.

## 3. Results

### 3.1 Overall structural and evolution characteristics

#### 3.1.1 Structural characteristics

**Fig 3:**
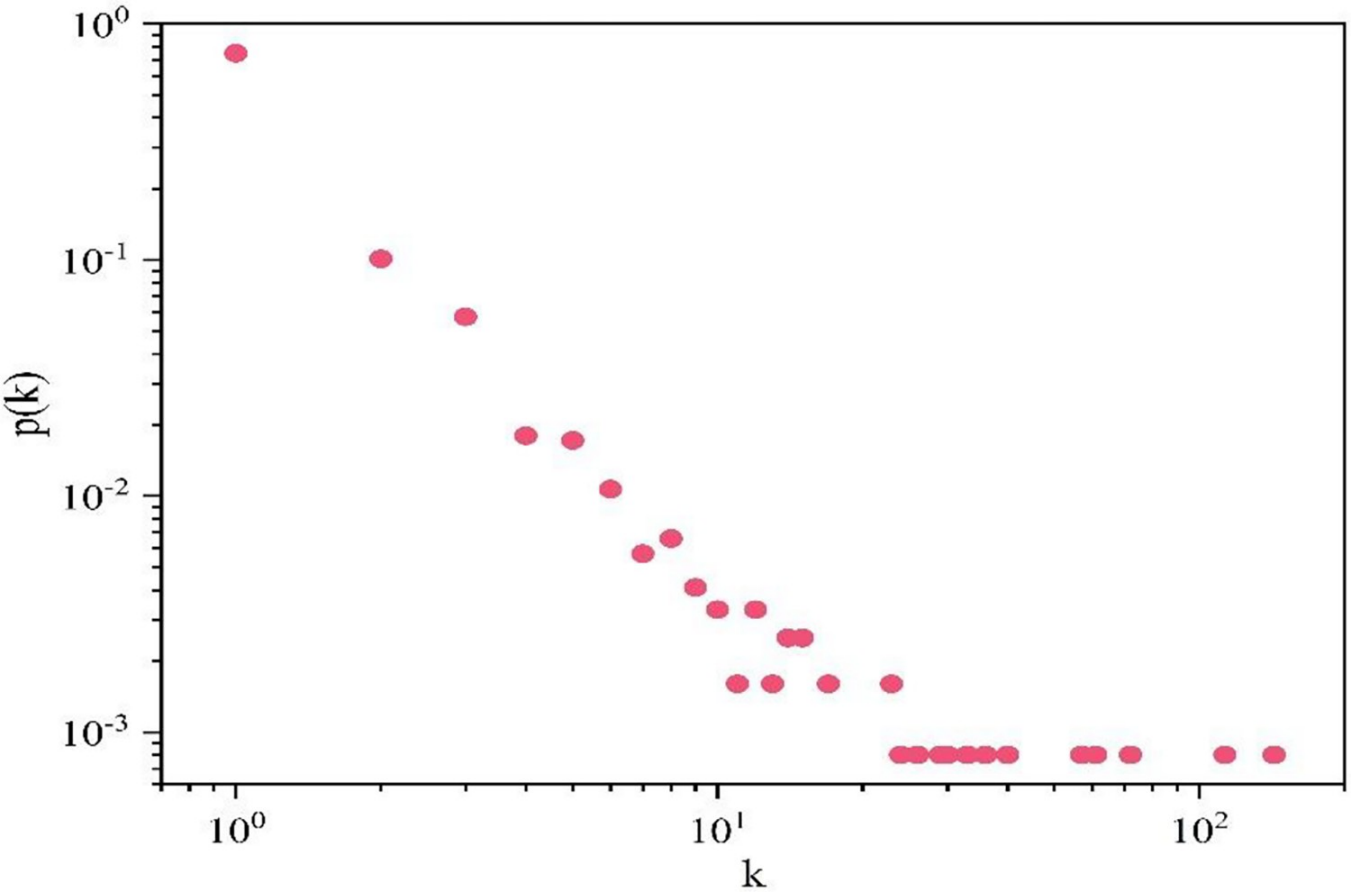
Degree distribution of terrorist organization cooperation networks, 2001–2018.

The degree distribution of the terrorist organization in the cooperative network is presented in Fig 3. With a degree of 143, the terrorist organization with the highest degree is the New People’s Army (NPA), whose principal objective is to "promote a long period of people’s war aimed at destroying the Philippine government." Founded in 1969, the New People’s Army (NPA) has a long history and a large number of personnel, and has long served as the armed wing of the Communist Party of the Philippines. Since the 1980s, the Communist Party of the Philippines (CPP) has reportedly had numerous splits with the NPA, leading to its split into multiple armed factions and terrorist organizations, which explains its numerous collaborative organizations. There are 29 terrorist organizations’ degree values in the network (29 hub nodes), and only a few hub nodes have several connections. Most of the nodes have only a small number of connections, and few hub nodes have more connected edges [16], reflecting the fact that the cooperative network of terrorist organizations is a scale-free network and follows the power law distribution.

The macro-level examination of network characteristics can evaluate the network topology, the information flow between nodes, and the network’s general characteristics [17]. Indicators of terrorist organization cooperation network features from 2001 to 2018 are presented in Table 1. The average degree of the network is 2.271, and each terrorist organization has a direct link with two or three other terrorist groups on average. The average weighted degree is derived from the average degree by considering the edge weight, which is the number of collaborations. The average weighted degree of 8.568 is much higher than the average degree value, which indicates that most terrorist organizations are not bent on establishing new cooperation relationships, and most terrorist organizations are more interested in improving the quality of cooperation and strengthening cooperation ties, thus reducing the risks associated with large-scale cooperation. In Fig 3, modularity is used for community discovery, with a modularity value of 0.89, a large value and a clear community differentiation. Larger communities are reflected in different colors in Fig 2.

#### 3.1.2 Network evolution

**Fig 4:**
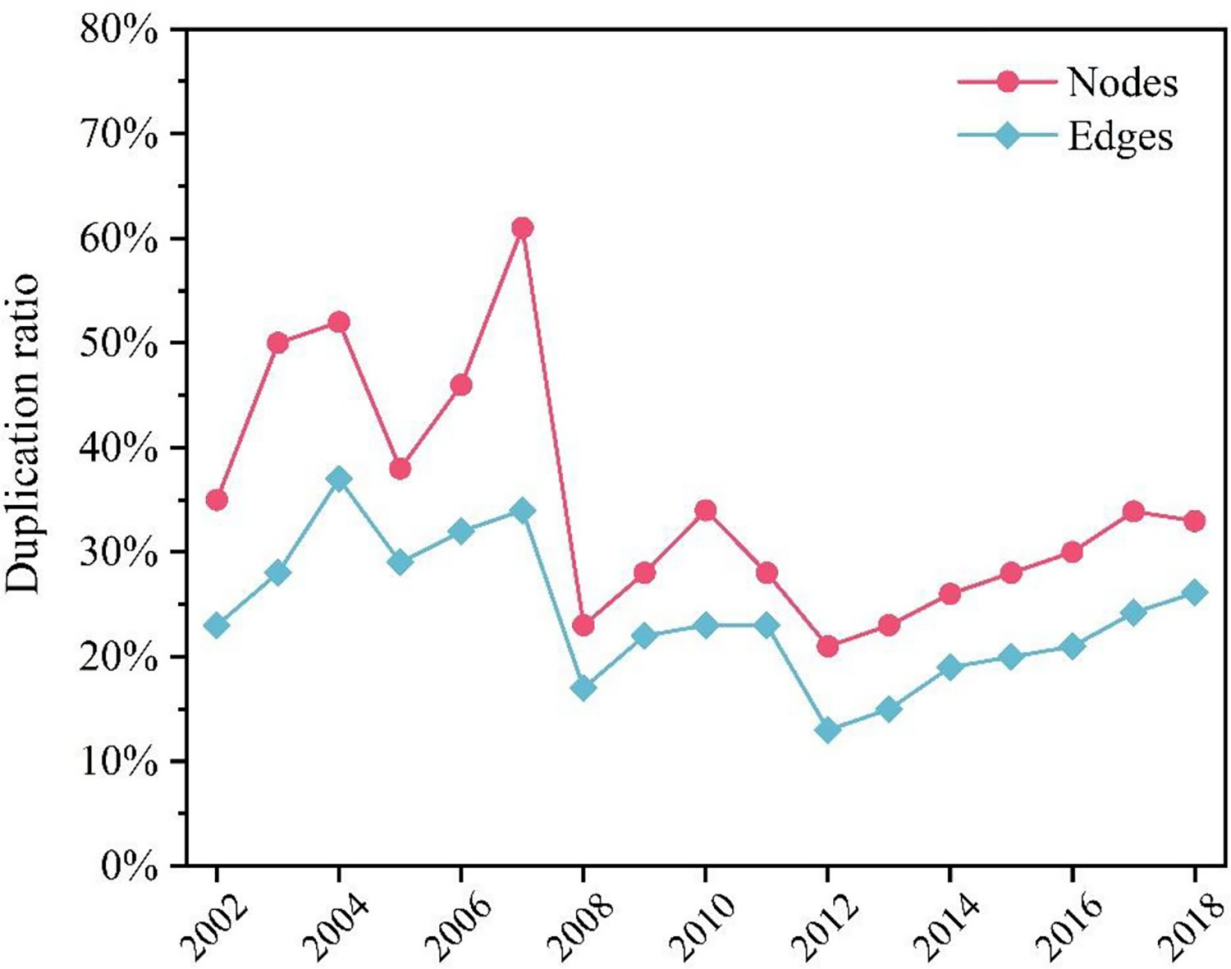
Percentage of nodes and edges of duplicated terrorist organization cooperation networks, 2002–2018.

As shown in Fig 4, by tallying the number of duplicated nodes and edges in each year and the previous year and calculating the ratio between them and the two years’ nodes and edges, we can determine the proportion of duplicated nodes and edges in the two adjacent years relative to the total number of nodes and edges in the two adjacent years. Over time, the proportion of duplicated nodes and edges evolves through a fluctuating-to-leveling process, with an overall decreasing trend. In 2006–2007, the percentage of duplicated nodes and edges reached 61% and 34%, respectively, as a significant number of terrorist organizations were active during these two years. The proportion of duplicated nodes and edges was unusually high between 2002 and 2007, but decreased after 2008, indicating that the concealing of terrorist group cooperation was strengthened.

A substantial body of research demonstrates that the cooperation networks of terrorist organizations are highly resilient; they can resume their fundamental organizational functions within a set amount of time after suffering a setback. Global Terrorism Databases (GTD) indicate that terrorist organizations appear and disappear at a rather rapid rate. From 1970 to the present, the GTD database has recorded more over 3,000 organizational actions, yet less than one in ten terrorist organizations are active each year and remain relatively constant. The life span of organizations shows a scale-free distribution; a large number of organizations have a short life span [18]. Only a relatively small number of organizations are able to remain viable for a longer period of time. The change trend of the time-slice network with 1 and 2 years and 3 years as the unit is consistent, so the time-slice network with 3 years as the unit can better record the process of creation and extinction of most organizations and can show the evolutionary changes of the cooperative network of terrorist organizations more clearly than the time-slice network with 1 and 2 years as the unit.

**Fig 5:**
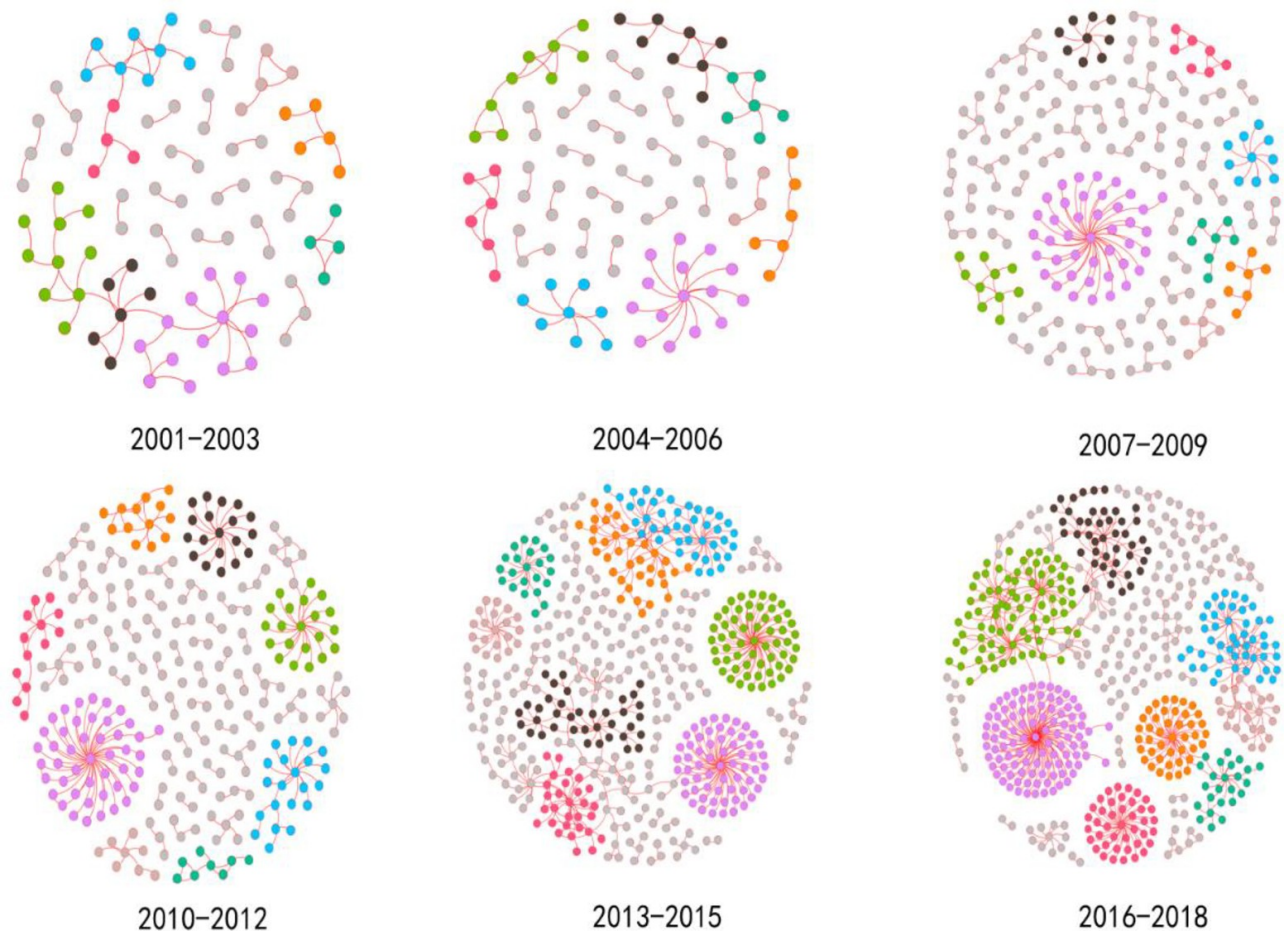
Evolution of global terrorist organizational cooperation networks in 3-year scale units of time slice, 2001–2018.

The overall network Is divided into six time slices according to the year in chronological order, and each time slice scale is 3 years. The data is used to visualize and analyze the network topology [19]. Fig 5 depicts the time-slice sub-network formed by slicing the years 2001–2018. As time passes, the number of nodes and edges increases, more organizations join the network, new relationships are formed, and community differentiation becomes clear.

The calculation of the indicators of the time-slice network, the results are shown in Table 2. The graph density in the table is decreasing, the terrorist organization cooperation network is becoming more sparse, the average path length as a whole is increasing, and the agglomeration coefficient as a whole is decreasing, all of which are caused by further differentiation of the terrorist organization community, resulting in the overall network information dissemination efficiency. The scale of terrorist organization cooperation networks is growing, terrorist organization activities are gradually shifting from traditional independent terrorist attacks to large-scale terrorist group cooperation attacks, and the community-based characteristics of terrorist organization cooperation networks are becoming more visible.

**Table 2:** Indicators of global terrorist organizational cooperation network characteristics, 2001–2018.

Time	Number of Nodes	Number of Edges	Density	Average Degree	Diameter	Average Path Length	Average Clustering Coefficient
2001–2018	1219	1384	0.002	2.271	14	5.233	0.296
2001–2003	90	80	0.02	1.778	8	3.484	0.449
2004–2006	99	82	0.017	1.657	6	2.146	0.435
2007–2009	222	172	0.007	1.55	4	1.932	0.258
2010–2012	284	239	0.006	1.683	5	2.11	0.305
2013–2015	501	518	0.004	2.068	16	5.675	0.281
2016–2018	558	653	0.004	2.341	12	4.013	0.308

### 3.2 Ranking of significant nodes

#### 3.2.1 Significant node identification

Quantifying the significance of nodes is essential to developing effective strategies to contain or accelerate any propagation phenomenon. The centrality metric is a well-known method used to quantify the influence of nodes by extracting information from the network structure [20]. Previous studies primarily used degree centrality to identify core terrorist organizations, but degree centrality only represents terrorist organizations’ importance and influence locally in the network to which they belong and does not measure their status and influence in terms of the overall network. Betweenness centrality, on the other hand, demonstrates the mediating influence of network nodes and reflects the ability to connect and aggregate other organizations [21]. As a result, the focus of this paper is on the effect of changes in betweenness centrality on the centrality of terrorist organizations. Betweenness centrality intuitively shows the relevance of nodes as "bridges". The betweenness centrality of terrorist organizations in the network is calculated per time slice from 2001 to 2018, and the betweenness centrality values are derived.

Ranking the centrality of terrorist organizations meshing in the network by numerical size for each year from 2001 to 2018 and counting the top five organizations (Table 3), it was discovered that JAISH-I-MOHAMMED, HARAKAT UL-MUJAHIDIN/HUM (Mujahideen Movement), AL-QAIDA, THE ORGANIZATION OF AL-QAIDA IN THE ISLAMIC MAGHREB, LASHKAR-E-TAYYIBA, and other organizations appear They are also rather well-known terrorist groups. A vast number of terrorist organizations rely on organizations that act as "bridges" to connect with other organizations and carry out terrorist activities together.

**Table 3:** Top 5 organizations in terms of betweenness centrality in each year, 2001–2018.

	1	2	3	4	5
2001	JAISH-I-MOHAMMED	HARAKAT UL-MUJAHIDIN / HUM	Army of Mohammed	AL-QAIDA	ISLAMIC MOVEMENT OF UZBEKISTAN
2002	AL-QAIDA	JAISH-I-MOHAMMED	National Movement for the Restoration of Pakista’’s Sovereignty	Lashkar-e-Taiba (LeT)	HARAKAT UL-MUJAHIDIN / HUM
2003	JAISH-I-MOHAMMED	LASHKAR-E-TAYYIBA	HARAKAT UL-MUJAHIDIN / HUM	LASHKAR I JHANGVI (LJ)	ISLAMIC MOVEMENT OF UZBEKISTAN
2004	ISLAMIC MOVEMENT OF UZBEKISTAN	AL-QAIDA	JAISH-I-MOHAMMED	LASHKAR-E-TAYYIBA	HARAKAT UL-MUJAHIDIN / HUM
2005	HARAKAT UL-MUJAHIDIN / HUM	AL-QAIDA	JAISH-I-MOHAMMED	EMARAT KAVKAZ	LASHKAR I JHANGVI (LJ)
2006	JAISH-I-MOHAMMED	Jamiat ul-Mujahedin (JuM)	LASHKAR-E-TAYYIBA	Salafist Group for Preaching and Fighting (GSPC)	El Feth Katibat
2007	Salafist Group for Preaching and Fighting (GSPC)	JAISH-I-MOHAMMED	LASHKAR-E-TAYYIBA	EMARAT KAVKAZ	HARAKAT UL-MUJAHIDIN / HUM
2008	Revolutionary Armed Forces of Colombia (FARC)	Moro Islamic Liberation Front (MILF)	JAISH-I-MOHAMMED	New Peopl’’s Army (NPA)	ABU SAYYAF GROUP
2009	Revolutionary Armed Forces of Colombia (FARC)	Frente 30	Frente 55	Communist Party of India–- Maoist (CPI-Maoist)	JAISH-I-MOHAMMED
2010	Revolutionary Armed Forces of Colombia (FARC)	TEHRIK-E TALIBAN PAKISTAN (TTP)	LASHKAR I JHANGVI (LJ)	HARAKAT UL-MUJAHIDIN / HUM	Communist Party of India–- Maoist (CPI-Maoist)
2011	Revolutionary Armed Forces of Colombia (FARC)	ABU SAYYAF GROUP	Alirio Torres Mobile Column	New Peopl’’s Army (NPA)	TEHRIK-E TALIBAN PAKISTAN (TTP)
2012	THE ORGANIZATION OF AL-QAIDA IN THE ISLAMIC MAGHREB	Free Syrian Army	TEHRIK-E TALIBAN PAKISTAN (TTP)	Revolutionary Armed Forces of Colombia (FARC)	ANSAR AL-ISLAM
2013	TEHRIK-E TALIBAN PAKISTAN (TTP)	Revolutionary Armed Forces of Colombia (FARC)	New Peopl’’s Army (NPA)	Jundallah (Pakistan)	Jandul Hafsa faction
2014	THE ORGANIZATION OF AL-QAIDA IN THE ISLAMIC MAGHREB	Ansar al-Sharia (Libya)	ISLAMIC STATE IN IRAQ AND THE LEVANT–- YEMEN	Al-Nusrah Front	Revolutionary Armed Forces of Colombia (FARC)
2015	New Peopl’’s Army (NPA)	JEMAAH ISLAMIYAH	THE ORGANIZATION OF AL-QAIDA IN THE ISLAMIC MAGHREB	ABU SAYYAF GROUP	RAJAH SOLAIMAN MOVEMENT
2016	JEMAAH ISLAMIYAH	ABU SAYYAF GROUP	RAJAH SOLAIMAN MOVEMENT	THE ORGANIZATION OF AL-QAIDA IN THE ISLAMIC MAGHREB	Islamic State of Iraq and the Levant (ISIL)
2017	New Peopl’’s Army (NPA)	JEMAAH ISLAMIYAH	ABU SAYYAF GROUP	ISLAMIC STATE IN IRAQ AND THE LEVANT–- YEMEN	JEMMAH ANSHORUT TAUHID (JAT)
2018	THE ORGANIZATION OF AL-QAIDA IN THE ISLAMIC MAGHREB	ANSAR AL-ISLAM	ISLAMIC STATE IN IRAQ AND THE LEVANT–- YEMEN	Ha’’at Tahrir al-Sham	AL-QAIDA

#### 3.2.2 Evolutionary analysis of significant nodes

If a node’s betweenness centrality ranks first among all nodes in the network for an extended length of time, it can reflect the node’s ongoing influence in the network, and changes in the node’s betweenness centrality ranking can also disclose the rise or fall of its status. In addition, the change in the ranking of the node’s betweenness centrality is shown by *R*:

$$R_{n}=r_{n+1}-r_{n}$$

Where, *R*_*n*_ is the change in the ranking of betweenness centrality in the *n* year, *r*_*n*+1_ is the ranking of betweenness centrality in the *n*+1 year, and *r*_*n*_ is the ranking of betweenness centrality in the *n* year. When *r*_*n*+1_≥*r*_*n*_, *R*_*n*_ can be considered as the progress of the ranking of betweenness centrality in the *n* year, and the opposite is the regression. The data on the shift in the betweenness centrality ranking values from 2001 to 2018 were counted, and the most backward organization ranking was 74. As a result, Fig 7 depicts the fluctuation of the ranking change, where the vertical coordinate represents the value of the ranking change and the three portions of the horizontal coordinate correspond to the first 1/3, 1/3-2/3, and last 1/3 of the organization ranking, respectively.

**Fig 6:**
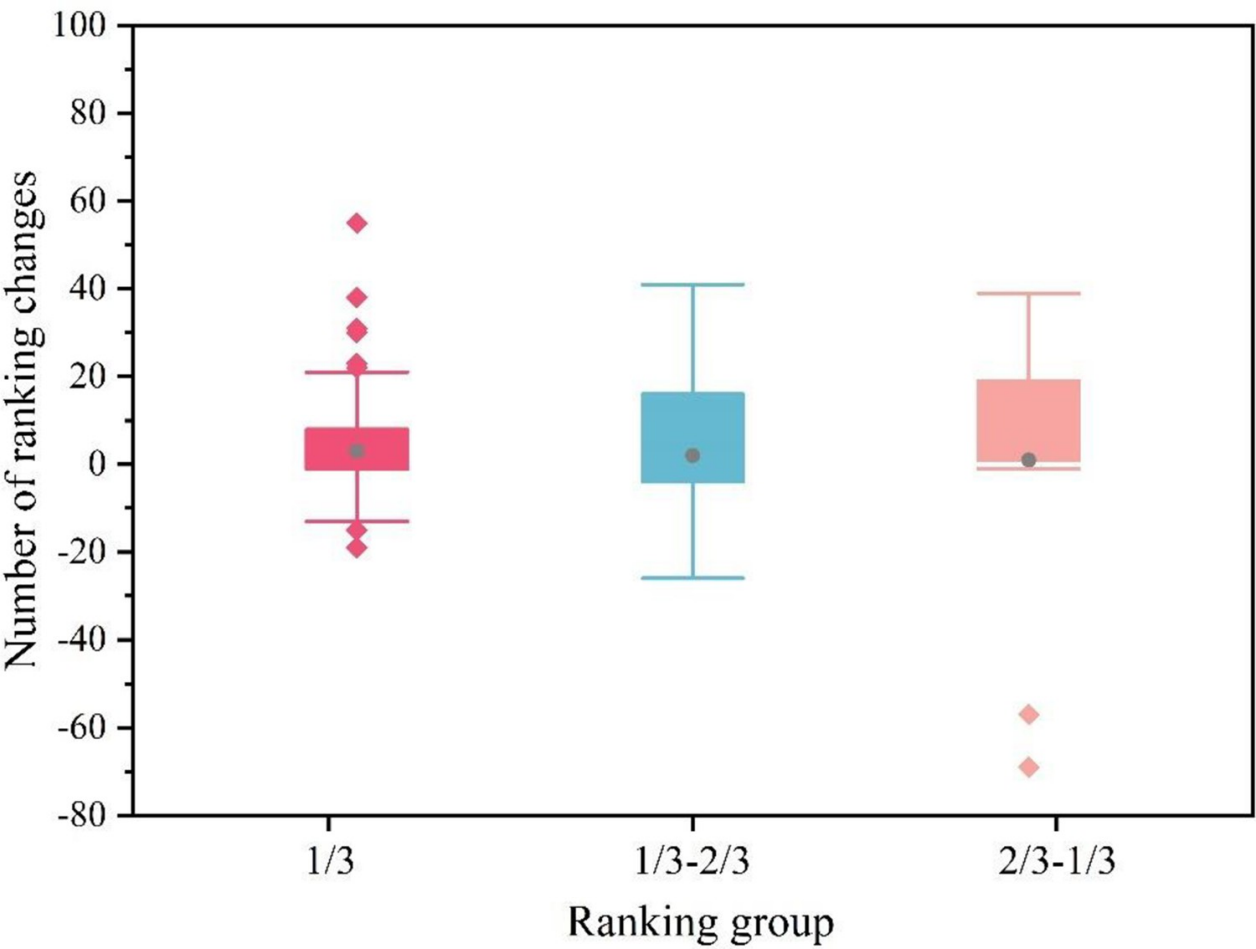
Box plot of the change in the ranking of the betweenness centrality of terrorist organizations.

Fig 6 depicts that in the cooperative network of terrorist organizations, the change in the ranking of the top 1/3 of terrorist organizations that serve as an important link and bridge between different organizations in the network betweenness centrality is less volatile, whereas the change in the ranking of the bottom 2/3 of terrorist organizations is more volatile.

At the same time, the weighted centrality indicator measures both the number and the frequency of terrorist organizations’ cooperation, and to some extent, it can also serve as a symbol of the importance of nodes. The weighted centrality of all terrorist organizations from 2001–2018 was ranked and collated to obtain 1242 ranking change data, 677 progress and 565 regressions. And the correlation analysis of progress, regression, change, and ranking was performed by the Spearman correlation coefficient. The results showed that the correlation coefficients of progress, regression, and change in ranking with the corresponding ranking data were less than 0.01, which showed significant correlation. The correlation coefficient between regression and ranking is 0.695, indicating a strong positive correlation; the larger the value of ranking, the greater the regression; the higher the ranking, the slower the organization’s regression; the correlation coefficient between progress and ranking is 0.22, indicating a weak degree of positive correlation; the larger the value of ranking, the greater the progress; the higher the betweenness centrality ranking, the slower the organization’s progress;

The ranking of terrorist organizations as significant nodes in the network is more stable during the dynamic evolution of terrorist organization cooperation networks, because when new terrorist organization nodes join the cooperation network, they need to communicate and exchange resources or information with the help of significant nodes. As a result, new terrorist organizations as edge nodes tend to connect directly to significant nodes with high betweenness centrality or strong influence rather than new nodes to each other, which may result in higher centrality index values for important nodes and, as a result, less fluctuation in the top organizations’ ranking. As more terrorist nodes join the network, the number of organizations with low centrality grows, and a little increase in the centrality index value may result in a considerable increase in the ranking of organizations, so that organizations with lower ranks change more. Overall, the node rankings are changing faster and faster, the rankings are fluctuating more and more, and the competition is becoming more and more intense. On the one hand, it reflects some significant achievements in the world’s counter-terrorism career; on the other hand, it shows that new terrorist forces are emerging and the counter-terrorism situation remains severe.

### 3.3 Network cohesiveness

#### 3.3.1 Cohesive identification analysis

**Fig 7:**
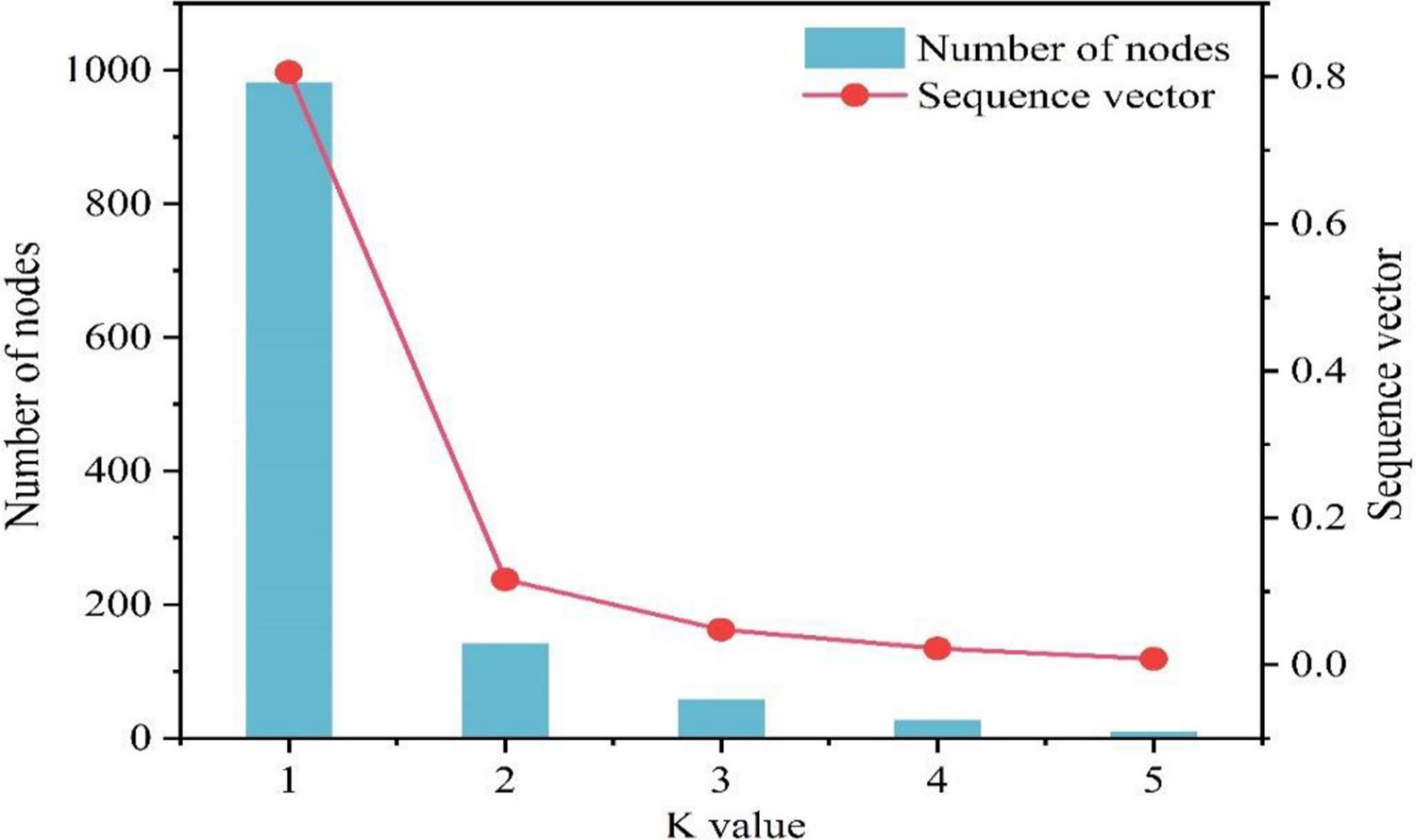
Distribution of core degree and nuclear collapse sequence of terrorist organizational cooperation networks.

The cooperative network of terrorist groups was *k*-core decomposed, yielding a total of 5 partitions for the cooperative network of 1219 terrorist organizations. The first core has 1219 organizations, the second core has 237 organizations, the third core has 95 organizations, the fourth core has 37 organizations, and the fifth core has 10 organizations. Each layer of the network, as it is clustered from the outside to the interior, may yield a certain number of residual nodes, resulting in a core collapse. When the *k* value is increased by one, the collapse sequence refers to the number and fraction of vertices lost. The network *k*-core analysis measured data were manually eliminated, and the number of collapsed nodes formed while increasing *k* from 1 to 5 was (982, 142, 58, 27, 10) in order, resulting in a kernel collapse sequence of (0.806, 0.116, 0.048, 0.022, 0.008), see Fig 7.

**Fig 8:**
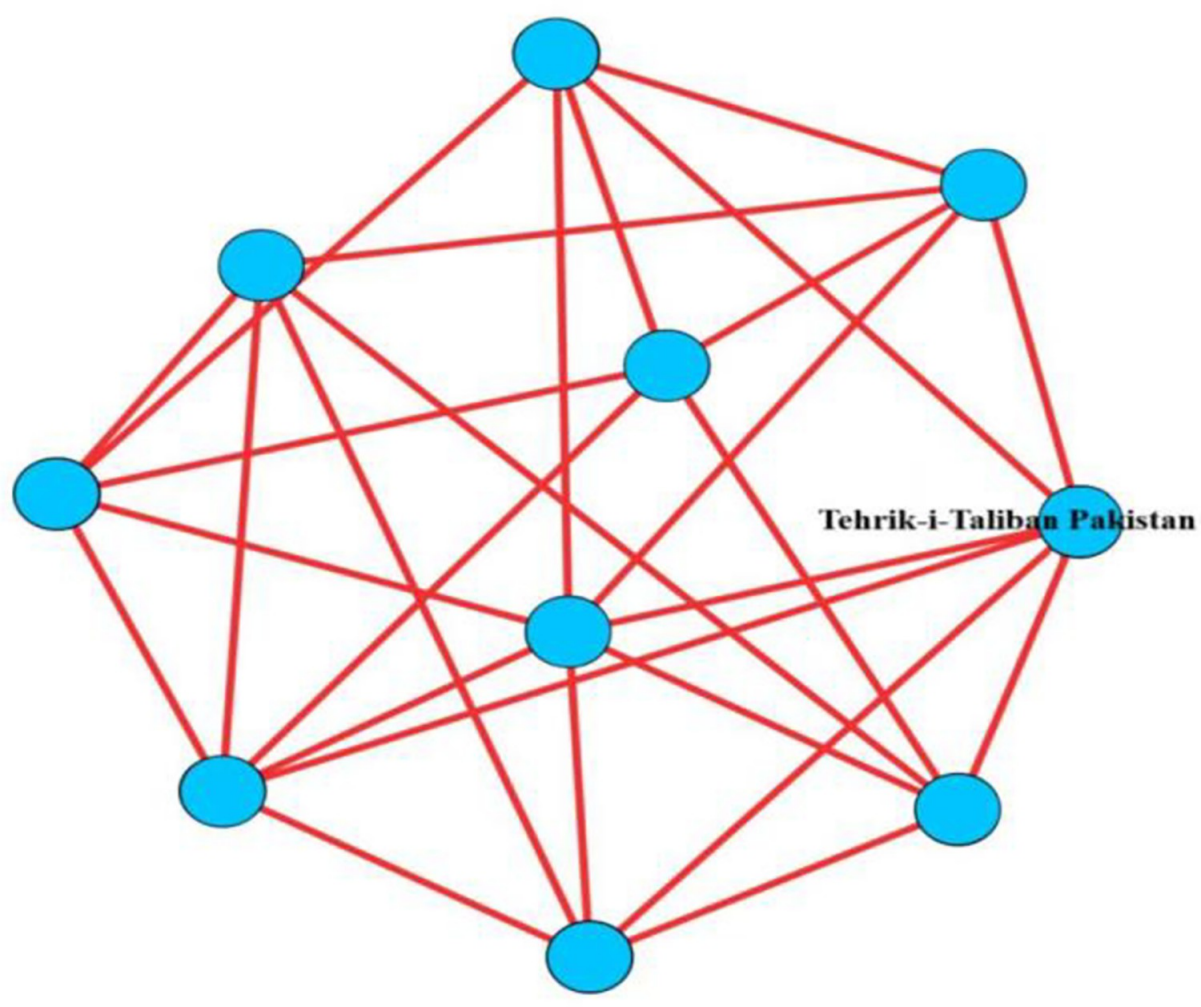
5-core groups in terrorist organizational cooperation networks.

Fig 7 shows that when the value of *k* increases, the number of nodes and the sequence vector as a whole decreases. When the value climbs from 1 to 2, the vector value decreases abruptly and sharply; when the *k* value is in the [2, 5] interval, the vector value decreases slowly. Most of the terrorist organizations are located in the region with a low clustering degree, and the remaining organizations in the region with a high clustering degree are less. The highest level of the terrorist organization cooperation network is the 5-core group, which contains a total of 10 terrorist organizations, which are closely connected to each other, and each organization has a direct cooperation relationship with at least 5 other organizations in the group, see Fig 8. Tehrik-i-Taliban Pakistan, the red node with the greatest degree value in the 5-core group diagram, is the organization with the highest degree value in the 5-nuclear core group. It has a degree of 61 and collaborates with 61 terrorist organizations. The high core and degree values show that this organization is not just central to the network but also has a significant influence.

#### 3.3.2 Cohesive evolutionary analysis

**Fig 9:**
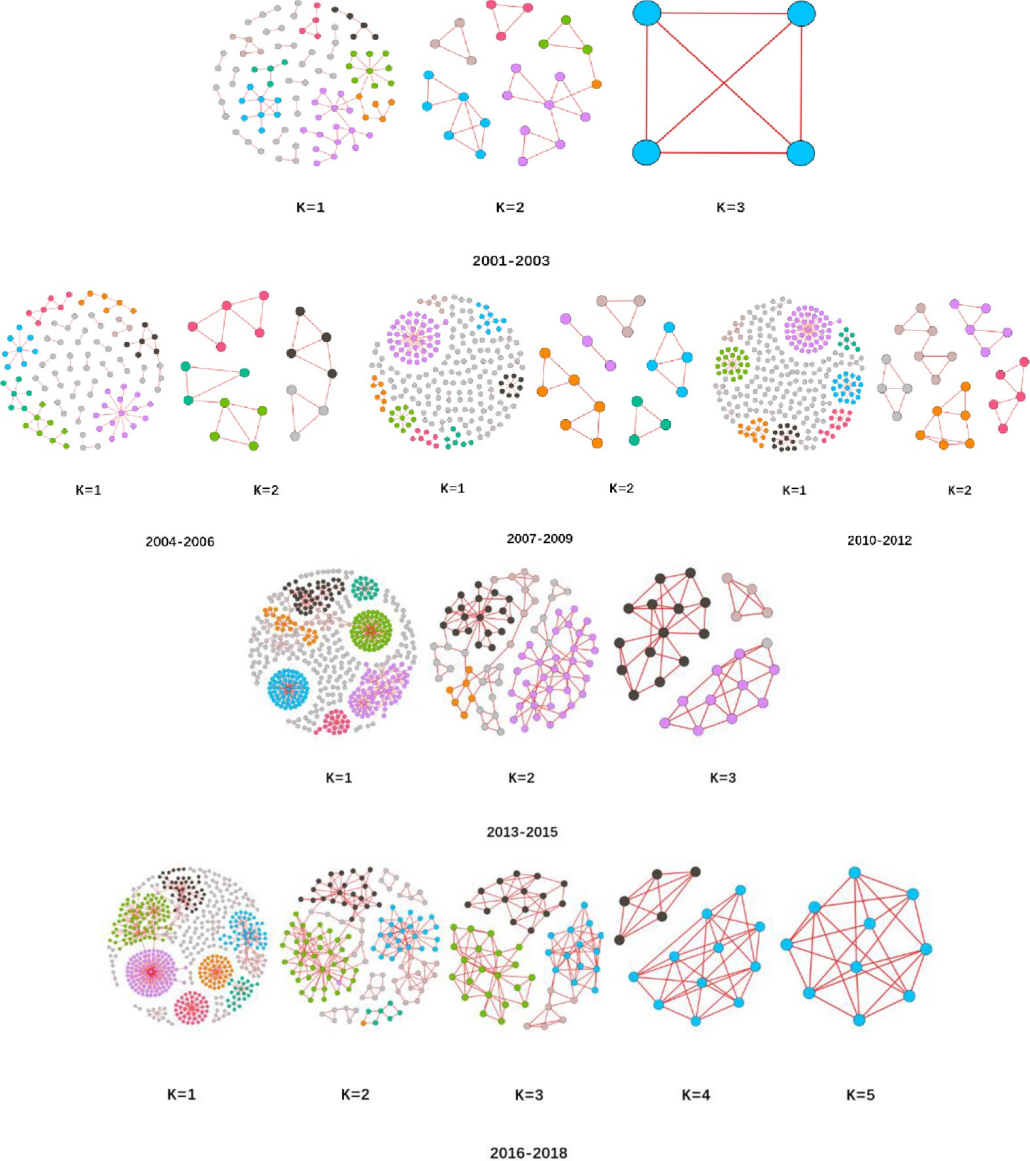
Stochastic core collapse process of terrorist organizational cooperation networks under time slice.

By doing *k*-core decomposition of the network for six time slices from 2001–2018, see Fig 9. The figure visualizes the stochastic core collapse process of the terrorist organization cooperation network, and the number of organizations with different *k*-core groups, the number share, and the corresponding number of core collapse nodes. The collapse sequences are shown in Table 4.

**Table 4:** Core degree distribution and collapse sequence of time-sliced networks.

	K value	1	2	3	4	5
2001–2003	Degree distribution	90	24	4		
	Collapse number	66	20	4		
	Nuclear collapse sequence	0.733	0.222	0.044		
2004–2006	Degree distribution	99	19			
	Collapse number	80	19			
	Nuclear collapse sequence	0.808	0.192			
2007–2009	Degree distribution	222	19			
	Collapse number	203	19			
	Nuclear collapse sequence	0.914	0.086			
2010–2012	Degree distribution	284	26			
	Collapse number	258	26			
	Nuclear collapse sequence	0.908	0.092			
2013–2015	Degree distribution	501	89	29		
	Collapse number	412	60	29		
	Nuclear collapse sequence	0.822	0.120	0.058		
2016–2018	Degree distribution	558	137	56	16	10
	Collapse number	421	81	40	6	10
	Nuclear collapse sequence	0.754	0.145	0.072	0.011	0.018

The overall increase in the number of *k*-core stratifications of the time-slice network of terrorist organization collaboration with time evolution seen in Fig 9 demonstrates the rising cohesiveness of the terrorist organization cooperation network’s core region. This is the statistical analysis of the nodes in the core position, in which the nodes and edges in the core position change less and the periphery position changes more. In other words, the changes in the terrorist organization’s network are more concentrated in the periphery, and the core is relatively stable.

Table 4 shows that the number of collapses has been increasing during the collapse of *k*-core from 1 to 2 in the four time-slice networks from 2001 to 2012, which indicates that the new cooperative relationships are mostly single-node pairs as the network size grows. The number of organizations at the edge of the "core-periphery" structure is increasing. Overall, the proportion of 1-core groups drops, while the proportion of 2-core groups gradually increases and 3 or higher groupings develop. When combined with the graph’s falling density, the overall trend of the terrorist group collaboration network is dropping, but local node aggregation is increasing.

### 3.4 Motif analysis

A motif is a network subgraph that occurs much more frequently in real networks than in random networks with the same number of nodes and connections. In most networks, the most common motifs consist of 3 or 4 nodes. Since the connectivity pattern of subgraphs increases rapidly with the number of nodes, the study of subgraph structure motif properties focuses on subgraphs with fewer nodes, and higher order subgraphs can be considered as a combination of 3-node and 4-node subgraphs. In this paper, we use the RAND-ESU (randomized enumerate subgraph) algorithm to obtain the percentage of each time slice network for 3 and 4 node motifs. The result shows that the themes of the time-slicing network of terrorist group collaboration all share the same characteristics. The "triadic closed" motif of 3 nodes and the "star" motif of 4 nodes have the highest proportion in each time slice network, and the highest proportion in the network may still be found by calculating the motif of 5 and 6 nodes. The "star" pattern remains the network’s most dominating motif. The number of "triadic closed" motifs declines throughout time, whereas the proportion of "star" motifs grows. At the same time, the proportion of the No. 3 motif is second only to the No. 2 "star" motif in the 4-node motif, but its structure can also be seen as a combination of the No. 1 "ternary closure" motif and the No. 2 “star” motif. Therefore, it can be concluded that the structure of the terrorist network is a hybrid topology with “star-like” motifs as the main part and “triadic closure” motifs as a supplement, and the proportion of each type of motif tends to be stable with time evolution.

The "triadic closure" shape of the motif makes it easy to form community structures in social interactions. The fully connected triad, as the smallest “clique” unit, has strong stability and cooperation with each other, which increases the success rate of terrorist attacks. The number of structured nodes and cooperative edges grows over time, and a small number of hub nodes emerge in the network, as hub nodes have preferential connectivity and tend to connect nodes with fewer connections than other hub nodes. Even if additional nodes are attacked, the network’s operation is unaffected from the perspective of the network subgraph with hub nodes as the core. Hub nodes tend to be built earlier, have more influence and strength, and are more difficult to hit, so the "star" subgraph network structure is more stable; the network structure is more stable from the perspective of other nodes connected to hub nodes. From the perspective of other nodes connected to hub nodes, cooperating with nodes with great influence and experience is more favorable to the successful execution of terrorist operations, which fall into the "the old guides the new" and "the strong teams up with the weak" modes. Unlike most legitimate and encouraged research collaborator networks and business collaborative networks, which are distinguished by a strong "triadic closure" paradigm [22, 23], terrorist collaborative networks are more "star-like" due to their concealment and necessity for long-term survival.

## 4. Conclusion and discussion

This research builds a worldwide terrorist organization cooperation network and then conducts an evolutionary investigation to determine if the terrorist organization cooperation network has scale-free network features based on the evolutionary dynamic characteristics of the pre-intervention control. The scale of the terrorist organization network is expanding, and new organization nodes and cooperative relationship connecting edges are emerging and tend to preferentially connect hub nodes. Hub nodes can attract new nodes and establish relationship connecting edge characteristics, strengthen control and combat for hub nodes, and reduce their activity and attractiveness to newborn terrorist organizations.

Community fragmentation is visible within the network, and by disconnecting the cooperative network, community bonds can be dissolved. The cooperative network’s community fragmentation feature is obvious; therefore, by attacking key nodes or nodes with high betweenness centrality ranking that connect different communities in the network, the exchange of resources between communities is disrupted, and the larger cooperative network is decomposed into smaller community networks to improve the striking efficiency.

The core node in the terrorist organization cooperation network is more stable, often playing the leadership role in the network. Compared to other organizations, mitigation operations targeting these nodes will be more difficult, as new leaders can be quickly re-produced by core organizations. At the same time, if the fight is targeting peripheral organizations, the effect of the fight against the network group will not be significant. Therefore, the most effective disintegration strategy is to separate the core and peripheral structures in the network, and make the terrorist organization cooperation network less connected by blocking the connection between the two types of nodes, such that the functioning of network can be largely reduced.

The network’s structure is mostly made up of "triadic closure" and star-shaped motif structures, and the proportion of each form of motif tends to stabilize over time. We use a combination of source control and hard and soft warfare techniques in response to the trend of organizational decentralization and member wandering. Terrorist organizations are getting increasingly networked, and their network structure is becoming increasingly sparse, posing a greater challenge to combat and dismantle. Coupled with the fact that terrorism will remain for a long time, tiny units, groups, and people with extremist views will continue to operate after fragmentation, even if large terrorist organizations are demolished through effective means. Intervene at the outset to prevent the establishment of terrorist organizations and terrorists and to limit the rise of terrorist follow-on forces through propaganda, education, and leadership.

## Supporting information

S1 Fig Examples of the United Nations Security Council consolidated list. The sanctions list updated by the UN Security Council Sanctions Committee includes information on terrorist organizations’ origins, leaders, organizational advantages, and cooperative organizations.
(PDF)

S2 Fig Global terrorist organizational cooperation network, 2001–2018. The global terrorist organization cooperation network from 2001 to 2018 was established by using SQL server software to clear the GTD data of individual organization events, collating and retaining the data of multiple organization cooperation, and incorporating the cooperation relationship of the UN sanctions list into the data to be added.
(PNG)

S3 Fig Degree distribution of terrorist organization cooperation networks, 2001–2018. Degree distribution is the statistics and display of the distribution of the number of cooperative relations between terrorist organizations.
(JPG)

S4 Fig Percentage of nodes and edges of duplicated terrorist organization cooperation networks, 2002–2018. By tallying the number of duplicated nodes and edges in each year and the previous year and calculating the ratio between them and the two years’ nodes and edges, we can determine the proportion of duplicated nodes and edges in the two adjacent years relative to the total number of nodes and edges in the two adjacent years.
(JPG)

S5 Fig Evolution of global terrorist organizational cooperation networks in 3-year scale units of time slice, 2001–2018. The overall network Is divided into six time slices according to the year in chronological order, and each time slice scale is 3 years. The data is used to visualize and analyze the network topology.
(PNG)

S6 Fig Box plot of the change in the ranking of the betweenness centrality of terrorist organizations. depicts the fluctuation of the ranking change, where the vertical coordinate represents the value of the ranking change and the three portions of the horizontal coordinate correspond to the first 1/3, 1/3-2/3, and last 1/3 of the organization ranking, respectively.
(JPG)

S7 Fig Distribution of core degree and nuclear collapse sequence of terrorist organizational cooperation networks. Each layer of the network, as it is clustered from the outside to the interior, may yield a certain number of residual nodes, resulting in a core collapse. When the k value is increased by one, the collapse sequence refers to the number and fraction of vertices lost. The network k-core analysis measured data were manually eliminated, and the number of collapsed nodes formed while increasing k from 1 to 5 was (982, 142, 58, 27, 10) in order, resulting in a kernel collapse sequence of (0.806, 0.116, 0.048, 0.022, 0.008).
(JPG)

S8 Fig 5-core groups in terrorist organizational cooperation networks. The highest level of the terrorist organization cooperation network is the 5-core group, which contains a total of 10 terrorist organizations, which are closely connected to each other, and each organization has a direct cooperation relationship with at least 5 other organizations in the group.
(JPG)

S9 Fig Stochastic core collapse process of terrorist organizational cooperation networks under time slice. By doing k-core decomposition of the network for six time slices from 2001–2018,the figure visualizes the stochastic core collapse process of the terrorist organization cooperation network.
(PNG)

S1 Table Sample data in the global terrorism database. This paper collected 119,803 terrorist attacks that occurred globally between 2001 and 2018, containing information such as the time of occurrence and the names of the terrorist organizations involved.
(XLSX)

S2 Table Indicators of global terrorist organizational cooperation network characteristics, 2001–2018. The overall network Is divided into six time slices according to the year in chronological order, and each time slice scale is 3 years. The data is used to analyze the network topology.
(XLSX)

S3 Table Top 5 organizations in terms of betweenness centrality in each year, 2001–2018. Betweenness centrality intuitively shows the relevance of nodes as "bridges". The betweenness centrality of terrorist organizations in the network is calculated per time slice from 2001 to 2018, and the betweenness centrality values are derived. Ranking the centrality of terrorist organizations meshing in the network by numerical size for each year from 2001 to 2018 and counting the top five organizations.
(XLSX)

S4 Table Core degree distribution and collapse sequence of time-sliced networks. By doing *k*-core decomposition of the network for six time slices from 2001–2018, the table shows the stochastic core collapse process of the terrorist organization cooperation network, and the number of organizations with different *k*-core groups, the number share, and the corresponding number of core collapse nodes.
(XLSX)

S5 Table Percentage of network by time slice for 3–6 node motifs. Higher order subgraphs can be considered as a combination of 3-node and 4-node subgraphs. By using the RAND-ESU (randomized enumerate subgraph) algorithm to obtain the percentage of each time slice network for 3 and 4 node motifs.
(XLSX)
