# Supplementary material for: Evolution of the global terrorist organizational cooperation network
Source: PLoS One. 2024 Jan 22;19(1):e0281615. doi: 10.1371/journal.pone.0281615 (PMC10824412; doi:10.1371/journal.pone.0281615)
Supplement: S1 Fig — The sanctions list updated by the UN Security Council Sanctions Committee includes information on terrorist organizations’ origins, leaders, organizational advantages, and cooperative organizations. (PDF) [file pone.0281615.s001.pdf]

The List established and maintained pursuant to Security Council res. 1267/1989/2253

Generated on: 8 February 2023

"Generated on refers to the date on which the user accessed the list and not the last date of substantive update to the list. Information on the substantive list updates are provided on the Council / Committee’ s website."

Composition of the List

The list consists of the two sections specified below:
A. **Individuals**
B. **Entities and other groups**

Information about de-listing may be found at:
https://www.un.org/securitycouncil/ombudsperson(for res. 1267)
https://www.un.org/securitycouncil/sanctions/delisting(for other Committees)
https://www.un.org/securitycouncil/content/2231/list(for res. 2231)

A. Individuals

QDi.430 Name: 1: EMRAAN 2: ALI 3: 4: na
Title: na **Designation:** na **DOB:** 4 Jul. 1967 **POB:** Rio Claro, Trinidad and Tobago **Good quality a.k.a.:** na **Low quality a.k.a.:** Abu Jihad TNT **Nationality: a)** Trinidad and Tobago **b)** United States of America **Passport no: a)** Trinidad and Tobago number TB162181 (issued on 27 January 2015, expired 26 January 2020) **b)** United States of America number 420985453 (expired 6 February 2017) **National identification no:** Trinidad and Tobago 19670704052 **Address: a)** United States of America (in detention, Federal Detention Center – Miami, Register Number: 10423-509) **b)** #12 Rio Claro Mayaro Road, Rio Claro, Trinidad and Tobago (previous location 2008-March 2015) **c)** #7 GUAYAGUAYARE Road, Rio Claro, Trinidad and Tobago (previous location circa 2003) **d)** United States of America (previous location- January 1991-2008) **Listed on:** 23 Nov. 2021 (amended on 1 Apr. 2022) **Other information:** Senior member of Islamic State in Iraq and the Levant (ISIL), listed as Al-Qaida in Iraq (QDe.115). Recruited for ISIL and instructed individuals to perpetrate terrorist acts. Physical description: height 176 cm, weight 73 kg, medium built, colour of eyes- brown, colour of hair- black/bald, complexion- brown. Speaks English. INTERPOL-UN Security Council Special Notice web link: https://www.interpol.int/en/How-we-work/Notices/View-UN-Notices-Individuals click here

QDi.400 Name: 1: IYAD 2: NAZMI 3: SALIH 4: KHALIL
Name (original script): إياد نظمي صالح خليل
Title: na **Designation:** na **DOB:** 1974 **POB:** Syrian Arab Republic **Good quality a.k.a.: a)** Ayyad Nazmi Salih Khalil **b)** Eyad Nazmi Saleh Khalil **Low quality a.k.a.: a)** Iyad al-Toubasi **b)** Iyad al-Tubasi **c)** Abu al-Darda' **d)** Abu-Julaybib al-Urduni **e)** Abu-Julaybib **Nationality:** Jordan **Passport no: a)** Jordan 654781 (approximately issued in 2009) **b)** Jordan 286062 (issued on 5 April 1999 at Zarqa, Jordan, expired on 4 April 2004) **National identification no:** na **Address:** Syrian Arab Republic (Coastal area of. Location as of April 2016) **Listed on:** 22 Feb. 2017 **Other information:** Leader of Al-Nusrah Front for the People of the Levant (QDe.137) for coastal area of Syrian Arab

Republic since March 2016. INTERPOL-UN Security Council Special Notice web link: https://www.interpol.int/en/How-we-work/Notices/View-UN-Notices-Individuals click here

QDi.343 Name: 1: ASHRAF 2: MUHAMMAD 3: YUSUF 4: 'UTHMAN 'ABD AL-SALAM
Name (original script): اشرف محمد يوسف عثمان عبد السلام
Title: na **Designation:** na **DOB:** 1984 **POB:** Iraq **Good quality a.k.a.: a)** Ashraf Muhammad Yusif 'Uthman 'Abd-al-Salam **b)** Ashraf Muhammad Yusuf 'Abd-al-Salam **c)** Ashraf Muhammad Yusif 'Abd al-Salam **Low quality a.k.a.: a)** Khattab **b)** Ibn al-Khattab **Nationality:** Jordan **Passport no: a)** K048787, issued in Jordan **b)** 486298, issued in Jordan **National identification no:** na **Address:** Syrian Arab Republic (located in as at Dec. 2014) **Listed on:** 23 Jan. 2015 (amended on 24 Nov. 2020, 23 Mar. 2021) **Other information:** A member of Al-Qaida (QDe.004) as of 2012 and a fighter in the Syrian Arab Republic since early 2014. Provided financial, material, and technological support for Al-Qaida, Al-Nusrah Front for the People of the Levant (QDe.137) and Al-Qaida in Iraq (AQI) (QDe.115).Review pursuant to Security Council resolution 2368 (2017) was concluded on 24 November 2020. INTERPOL-UN Security Council Special Notice web link: https://www.interpol.int/en/How-we-work/Notices/View-UN-Notices-Individuals click here

QDi.012 Name: 1: NASHWAN 2: ABD AL-RAZZAQ 3: ABD AL-BAQI 4: na
Name (original script): نشوان عبد الرزاق عبد الباقي
Title: na **Designation:** na **DOB:** 1961 **POB:** Mosul, Iraq **Good quality a.k.a.: a)** Abdal Al-Hadi Al-Iraqi **b)** Abd Al-Hadi Al-Iraqi **c)** Omar Uthman Mohammed **d)** Abdul Hadi Arif Ali **Low quality a.k.a.: a)** Abu Abdallah **b)** Abdul Hadi al-Taweel **c)** Abd al-Hadi al-Ansari **d)** Abd al-Muhayman **e)** Abu Ayub **Nationality:** Iraq **Passport no:** na **National identification no:** Ration Card no 0094195 **Address:** na **Listed on:** 6 Oct. 2001 (amended on 14 May 2007, 27 Jul. 2007, 10 Dec. 2015, 24 Nov. 2020) **Other information:** Joined Al-Qaida in 1996 and was at that time an important liaison to the Taliban in Afghanistan. Received money from Ansar al-Islam (QDe.098) in order to conduct attacks in Kirkuk and Ninveh in Iraq during spring and summer of 2005. Al-Qaida senior official. In custody of the United States of America, as of Aug. 2014. Father' s name: Abd al-Razzaq Abd al-Baqi. Mother' s name: Nadira Ayoub Asaad. Photo available for inclusion in the INTERPOL-UN Security Council Special Notice. Review pursuant to Security Council resolution 1822 (2008) was concluded on 15 Jun. 2010. Review pursuant to Security Council resolution 2368 (2017) was concluded on 24 November 2020. INTERPOL-UN Security Council Special Notice web link: https://www.interpol.int/en/How-we-work/Notices/View-UN-Notices-Individuals click here

QDi.192 Name: 1: ABD ALLAH 2: MOHAMED 3: RAGAB 4: ABDEL RAHMAN
Name (original script): عبد الله محمد رجب عبد الرحمن
Title: na **Designation:** na **DOB:** 3 Nov. 1957 **POB:** Kafr Al-Shaykh, Egypt **Good quality a.k.a.: a)** Abu Al-Khayr **b)** Ahmad Hasan **c)** Abu Jihad **Low quality a.k.a.:** na **Nationality:** Egypt **Passport no:** na **National identification no:** na **Address:** (Believed to be in Pakistan or Afghanistan) **Listed on:** 29 Sep. 2005 (amended on 13 Dec. 2011, 1 May 2019) **Other information:** Member of Egyptian Islamic Jihad (QDe.003). Review pursuant to Security Council resolution 1822 (2008) was concluded on 1 Jun. 2010. Review pursuant to Security Council resolution 2253 (2015) was concluded on 21 Feb. 2019. Review pursuant to Security Council resolution 2368 (2017) was concluded on 15 November 2021. INTERPOL-UN Security Council Special Notice web link: https://www.interpol.int/en/How-we-work/Notices/View-UN-Notices-Individuals click here

QDi.054 Name: 1: MAJEED 2: ABDUL CHAUDHRY 3: na 4: na
Title: na **Designation:** na **DOB: a)** 15 Apr. 1939 **b)** 1938 **POB:** na **Good quality a.k.a.: a)** Majeed, Abdul **b)** Majeed Chaudhry Abdul **c)** Majid, Abdul **Low quality a.k.a.:** na **Nationality:** Pakistan **Passport no:** na **National identification no:** na **Address:** na **Listed on:** 24 Dec. 2001 (amended on 1 May 2019, 2 Feb. 2023) **Other information:** Reportedly deceased. Review pursuant to Security Council resolution 1822 (2008) was concluded on 1 Jun. 2010. Review pursuant to Security Council resolution 2253 (2015) was concluded on 21 Feb. 2019. INTERPOL-UN Security

Council Special Notice web link: https://www.interpol.int/en/How-we-work/Notices/View-UN-Notices-Individuals click here

QDi.018 Name: 1: ABDUL MANAN AGHA 2: na 3: na 4: na
Name (original script): عبد المنان آغا
Title: Haji **Designation:** na **DOB:** na **POB:** na **Good quality a.k.a.:** Abdul Manan **Low quality a.k.a.:** **a)** Abdul Man' am Saiyid **b)** Saiyid Abd al-Man (formerly listed as) **Nationality:** na **Passport no:** na **National identification no:** na **Address:** na **Listed on:** 17 Oct. 2001 (amended on 26 Jun. 2013, 1 May 2019) **Other information:** Pakistan. Review pursuant to Security Council resolution 1822 (2008) was concluded on 15 Jun. 2010. Review pursuant to Security Council resolution 2253 (2015) was concluded on 21 Feb. 2019. Review pursuant to Security Council resolution 2610 (2021) was concluded on 8 November 2022.

QDi.295 Name: 1: MUHAMMAD 2: JIBRIL 3: ABDUL RAHMAN 4: na
Title: na **Designation:** na **DOB:** **a)** 28 May 1984 **b)** 3 Dec. 1979 **c)** 3 Mar. 1979 (from false passport) **POB:** East Lombok, West Nusa Tenggara, Indonesia **Good quality a.k.a.:** **a)** Mohammad Jibril Abdurrahman **b)** Muhammad Jibriel Abdul Rahman **c)** Mohammad Jibriel Abdurrahman **d)** Muhamad Ricky Ardhan born 8 Aug. 1980 (appears in false Indonesian passport number S335026) **e)** Muhammad Ricky Ardhan bin Muhammad Iqbal **f)** Muhammad Ricky Ardhan bin Abu Jibril **Low quality a.k.a.:** **a)** Muhammad Yunus **b)** Heris Syah **Nationality:** Indonesia **Passport no:** na **National identification no:** **a)** Indonesian national identity card number 3219222002.2181558 **b)** Identification number 2181558 **Address:** **a)** Jalan M. Saidi RT 010 RW 001 Pesanggrahan, South Petukangan, South Jakarta, Indonesia **b)** Jalan Nakula of Witana Harja Complex Block C, Pamulang, Banten, Indonesia **Listed on:** 12 Aug. 2011 (amended on 24 Nov. 2020) **Other information:** Senior member of Jemaah Islamiyah (QDe.092) directly involved in obtaining funding for terrorist attacks. Sentenced in Indonesia to five years in prison on 29 Jun. 2010. Father' s name is Mohamad Iqbal Abdurrahman (QDi.086). Review pursuant to Security Council resolution 2368 (2017) was concluded on 24 November 2020. Review pursuant to Security Council resolution 2368 (2017) was concluded on 15 November 2021. INTERPOL-UN Security Council Special Notice web link: https://www.interpol.int/en/How-we-work/Notices/View-UN-Notices-Individuals click here

QDi.229 Name: 1: ALY 2: SOLIMAN 3: MASSOUD 4: ABDUL SAYED
Title: na **Designation:** na **DOB:** 1969 **POB:** Tripoli, Libya **Good quality a.k.a.:** **a)** Ibn El Qaim **b)** Mohamed Osman **Low quality a.k.a.:** Adam **Nationality:** Libya **Passport no:** Libya number Libyan Passport No. 96/184442 **National identification no:** na **Address:** Ghout El Shamal, Tripoli, Libya **Listed on:** 8 Jun. 2007 (amended on 13 Dec. 2011, 1 May 2019) **Other information:** Member of Libyan Islamic Fighting Group (QDe.011). Review pursuant to Security Council resolution 1822 (2008) was concluded on 24 Nov. 2009. Review pursuant to Security Council resolution 2253 (2015) was concluded on 21 Feb. 2019. Review pursuant to Security Council resolution 2610 (2021) was concluded on 8 November 2022. INTERPOL-UN Security Council Special Notice web link: https://www.interpol.int/en/How-we-work/Notices/View-UN-Notices-Individuals click here

QDi.309 Name: 1: ABDUR REHMAN 2: na 3: na 4: na
Name (original script): عبد الرحمن
Title: na **Designation:** na **DOB:** 3 Oct. 1965 **POB:** Mirpur Khas, Pakistan **Good quality a.k.a.:** **a)** Abdul Rehman; Abd Ur-Rehman; Abdur Rahman **b)** عبد الرحمن السيندي (Abdul Rehman Sindhi; Abdul Rehman al-Sindhi; Abdur Rahman al-Sindhi; Abdur Rehman Sindhi; Abdurahman Sindhi) **c)** عبدالله السندي (Abdullah Sindhi) **Low quality a.k.a.:** Abdur Rehman Muhammad Yamin **Nationality:** Pakistan **Passport no:** Pakistan number CV9157521, issued on 8 Sep. 2008 (expires on 7 Sep. 2013) **National identification no:** Pakistan national identity card number 44103-5251752-5 **Address:** Karachi, Pakistan **Listed on:** 14 Mar. 2012 (amended on 1 May 2019) **Other information:** Has provided facilitation and financial services to Al-Qaida (QDe.004). Associated with Harakatul Jihad Islami (QDe.130), Jaish-I-Mohammed (QDe.019), and Al-Akhtar Trust International (QDe.121). Review pursuant to Security Council resolution 2253 (2015) was concluded on 21 Feb. 2019. Review

pursuant to Security Council resolution 2610 (2021) was concluded on 8 November 2022. INTERPOL-UN Security Council Special Notice web link: https://www.interpol.int/en/How-we-work/Notices/View-UN-Notices-Individuals click here

QDi.363 Name: 1: MAGHOMED 2: MAGHOMEDZAKIROVICH 3: ABDURAKHMANOV 4: na
Name (original script): Абдурахманов Магомед Магомедзакирович
Title: na **Designation:** na **DOB:** 24 Nov. 1974 **POB:** Khadzhalmahi Village, Levashinskiy District, Republic of Dagestan, Russian Federation **Good quality a.k.a.:** na **Low quality a.k.a.: a)** Abu Banat (original script: Абу Банат) **b)** Abu al Banat (original script: Абу аль Банат) **Nationality:** Russian Federation **Passport no:** Russian foreign travel passport number 515458008 (expires 30 May 2017) **National identification no:** Russian Federation national passport number 8200203535 **Address: a)** Turkey (possible location) **b)** Syrian Arab Republic (previous confirmed location since Sep. 2012) **Listed on:** 2 Oct. 2015 (amended on 24 Nov. 2020) **Other information:** As at Aug. 2015, leader of Jamaat Abu Banat terrorist group, which forms part of the Islamic State in Iraq and the Levant (ISIL), listed as Al-Qaida in Iraq (QDe.115), and operates on the outskirts of Syrian Arab Republic cities Aleppo and Idlib, extorting funds from and carrying out kidnappings and public executions of local Syrians. Physical description: eye colour brown, hair colour: dark, build: strong, straight nose, height: 180-185 cm, speaks Russian, English, Arabic. Wanted by the authorities of the Russian Federation for terrorist crimes committed in its territory. Photo available for inclusion in the INTERPOL-UN Security Council Special Notice. Review pursuant to Security Council resolution 2368 (2017) was concluded on 24 November 2020. INTERPOL-UN Security Council Special Notice web link: https://www.interpol.int/en/How-we-work/Notices/View-UN-Notices-Individuals click here

QDi.086 Name: 1: MOHAMAD 2: IQBAL 3: ABDURRAHMAN 4: na
Title: na **Designation:** na **DOB:** 17 Aug. 1957 **POB: a)** Korleko-Lombok Timur, Indonesia **b)** Tirpas-Selong Village, East Lombok, Indonesia **c)** Korleko-Lombok Timur, Indonesia **Good quality a.k.a.: a)** Rahman, Mohamad Iqbal **b)** A Rahman, Mohamad Iqbal **c)** Abu Jibril Abdurrahman **d)** Fikiruddin Muqti **e)** Fihiruddin Muqti **f)** Abdul Rahman, Mohamad Iqbal **Low quality a.k.a.:** na **Nationality:** Indonesia **Passport no:** na **National identification no:** 3603251708570001 **Address:** Jalan Nakula, Komplek Witana Harja III Blok C 106-107, Tangerang, Indonesia **Listed on:** 28 Jan. 2003 (amended on 26 Nov. 2004, 16 May 2011, 10 Jun. 2011, 6 Dec. 2019) **Other information:** Review pursuant to Security Council resolution 1822 (2008) was concluded on 8 Jun. 2010. Review pursuant to Security Council resolution 2368 (2017) was concluded on 4 Dec. 2019 INTERPOL-UN Security Council Special Notice web link: https://www.interpol.int/en/How-we-work/Notices/View-UN-Notices-Individuals click here

QDi.414 Name: 1: ABDULPATTA 2: ESCALON 3: ABUBAKAR 4: na
Title: na **Designation:** na **DOB: a)** 3 Mar. 1965 **b)** 1 Jan. 1965 **c)** 11 Jan. 1965 **POB:** Tuburan, Basilan Province, Philippines **Good quality a.k.a.: a)** Abdulpatta Abubakar Escalon **b)** Abdul Patta Escalon Abubakar **c)** Abdul Patta Abu Bakar **Low quality a.k.a.:** na **Nationality:** Philippines **Passport no: a)** Philippines number EC6530802 (expires 19 Jan. 2021) **b)** Philippines number EB2778599 **National identification no: a)** Saudi Arabia 2135314355 **b)** Saudi Arabia 202112421 **Address: a)** Philippines **b)** Jeddah, Saudi Arabia (previous address) **c)** Daina, Saudi Arabia (previous address) **Listed on:** 18 Jun. 2018 **Other information:** Facilitator for the Islamic State in Iraq and the Levant (ISIL), listed as Al-Qaida in Iraq (QDe.115). Gender: male. INTERPOL-UN Security Council Special Notice web link: https://www.interpol.int/en/How-we-work/Notices/View-UN-Notices-Individuals click here

QDi.304 Name: 1: MOCHAMMAD 2: ACHWAN 3: na 4: na
Title: na **Designation:** na **DOB: a)** 4 May 1948 **b)** 4 May 1946 **POB:** Tulungagung, Indonesia **Good quality a.k.a.: a)** Muhammad Achwan **b)** Muhammad Akhwan **c)** Mochtar Achwan **d)** Mochtar Akhwan **e)** Mochtar Akwan **Low quality a.k.a.:** na **Nationality:** Indonesia **Passport no:** na **National identification no: a)** Indonesia National Identity Card 3573010405480001 **b)** Indonesia National Identity Card 353010405480001 **Address:** Jalan Ir. H. Juanda 8/10, RT/RW 002/001, Jodipan, Blimbing, Malang, 65127, Indonesia **Listed on:** 12 Mar. 2012 (amended on 27 May 2022) **Other**

information: Acting emir of Jemmah Anshorut Tauhid (JAT) (QDe.133). Associated with Abu Bakar Ba' asyir (QDi.217), Abdul Rahim Ba' aysir (QDi.293) and Jemaah Islamiyah (QDe.092). Review pursuant to Security Council resolution 2368 (2017) was concluded on 15 November 2021. INTERPOL-UN Security Council Special Notice web link: https://www.interpol.int/en/How-we-work/Notices/View-UN-Notices-Individuals click here

QDi.316 Name: 1: IYAD 2: AG GHALI 3: na 4: na
Name (original script): اياد اغ غالي
Title: na **Designation:** na **DOB:** 1958 **POB:** Abeibara, Kidal Region, Mali **Good quality a.k.a.:** Sidi Mohamed Arhali born 1 Jan. 1958 in Bouressa, Bourem Region, Mali **Low quality a.k.a.:** na **Nationality:** Mali **Passport no:** Mali number A1037434, issued on 10 Aug. 2001 (expires on 31 Dec. 2014) **National identification no:** Mali Birth certificate 012546 **Address:** Mali **Listed on:** 25 Feb. 2013 (amended on 23 Sep. 2014, 1 May 2019) **Other information:** Founder and leader of Ansar Eddine (QDe.135). Member of the Tuareg Ifogas tribe. Linked to the Organization of Al-Qaida in the Islamic Maghreb (QDe.014) and Mouvement pour l' Unification et le Jihad en Afrique de l' Ouest (MUJAO) (QDe.134). Name of father is Ag Bobacer Arhali, name of mother is Rhiachatou Wallet Sidi. Review pursuant to Security Council resolution 2253 (2015) was concluded on 21 Feb. 2019. Review pursuant to Security Council resolution 2610 (2021) was concluded on 8 November 2022. INTERPOL-UN Security Council Special Notice web link: https://www.interpol.int/en/How-we-work/Notices/View-UN-Notices-Individuals click here

QDi.424 Name: 1: BAH 2: AG MOUSSA 3: na 4: na
Title: na **Designation:** na **DOB:** **a)** 1 Jan. 1958 **b)** 31 Dec. 1952 **c)** 28 Oct. 1956 **POB:** na **Good quality a.k.a.:** **a)** Ag Mossa **b)** Ammi Salim **Low quality a.k.a.:** na **Nationality:** Mali **Passport no:** na **National identification no:** na **Address:** na **Listed on:** 14 Aug. 2019 (amended on 20 Aug. 2019) **Other information:** Founding member of Ansar Eddine (QDe.135), operational leader of Jama'a Nusrat ul-Islam wa al-Muslimin (JNIM) (QDe.159). INTERPOL-UN Security Council Special Notice web link: https://www.interpol.int/en/How-we-work/Notices/View-UN-Notices-Individuals click here

QDi.203 Name: 1: FARHAD 2: KANABI 3: AHMAD 4: na
Name (original script): فرهاد كنابي أحمد
Title: na **Designation:** na **DOB:** 1 Jul. 1971 **POB:** Arbil, Iraq **Good quality a.k.a.:** **a)** Kaua Omar Achmed **b)** Kawa Hamawandi (previously listed as) **c)** Kawa Omar Ahmed **Low quality a.k.a.:** na **Nationality:** Iraq **Passport no:** German travel document ("Reiseausweis") A 0139243 (revoked as at Sep. 2012) **National identification no:** na **Address:** Arbil – Qushtuba – house no. SH 11, alley 5380, Iraq **Listed on:** 6 Dec. 2005 (amended on 31 Jul. 2006, 25 Jan. 2010, 13 Dec. 2011, 15 Nov. 2012, 10 Dec. 2015, 1 May 2019) **Other information:** Mother' s name: Farida Hussein Khadir. Released from custody in Germany on 10 Dec. 2010 and relocated to Iraq on 6 Dec. 2011. Review pursuant to Security Council resolution 1822 (2008) was concluded on 5 Oct. 2009. Review pursuant to Security Council resolution 2253 (2015) was concluded on 21 Feb. 2019. Review pursuant to Security Council resolution 2610 (2021) was concluded on 8 November 2022. INTERPOL-UN Security Council Special Notice web link: https://www.interpol.int/en/How-we-work/Notices/View-UN-Notices-Individuals click here

QDi.226 Name: 1: NAJMUDDIN 2: FARAJ 3: AHMAD 4: na
Title: na **Designation:** na **DOB:** **a)** 7 Jul. 1956 **b)** 17 Jun. 1963 **POB:** Olaqloo Sharbajer, Al-Sulaymaniyah Governorate, Iraq **Good quality a.k.a.:** **a)** Mullah Krekar **b)** Fateh Najm Eddine Farraj **c)** Faraj Ahmad Najmuddin **Low quality a.k.a.:** na **Nationality:** Iraq **Passport no:** na **National identification no:** Ration card no. 0075258 **Address:** Heimdalsgate 36-V, Oslo, 0578, Norway **Listed on:** 7 Dec. 2006 (amended on 10 Dec. 2015, 24 Nov. 2020) **Other information:** Mother' s name: Masouma Abd al-Rahman. Photo available for inclusion in the INTERPOL-UN Security Council Special Notice. Review pursuant to Security Council resolution 1822 (2008) was concluded on 20 May 2010. Review pursuant to Security Council resolution 2368 (2017) was concluded on 24

November 2020. INTERPOL-UN Security Council Special Notice web link: https://www.interpol.int/en/How-we-work/Notices/View-UN-Notices-Individuals click here

QDi.237 Name: 1: JABER 2: ABDALLAH 3: JABER 4: AHMAD AL-JALAHMAH
Name (original script): جابر عبد الله جابر أحمد الجلاهمة
Title: na **Designation:** na **DOB:** 24 Sep. 1959 **POB:** Al-Khitan area, Kuwait **Good quality a.k.a.: a)** Jaber Al-Jalamah **b)** Abu Muhammad Al-Jalahmah **c)** Jabir Abdallah Jabir Ahmad Jalahmah **d)** Jabir 'Abdallah Jabir Ahmad Al-Jalamah **e)** Jabir Al-Jalhami **Low quality a.k.a.: a)** Abdul-Ghani **b)** Abu Muhammad **Nationality:** Kuwait **Passport no: a)** 101423404 **b)** Kuwait number 2541451 (valid until 16 Feb. 2017) **c)** Kuwait number 002327881 **National identification no:** Kuwait 259092401188 **Address:** Kuwait (residence as at March 2009 and at December 2013) **Listed on:** 3 Jan. 2014 **Other information:** Previously listed between 16 Jan. 2008 and 3 Jan. 2014 (amended on 1 Jul. 2008, 23 Jul. 2008, 25 Jan. 2010). Review pursuant to Security Council resolution 1822 (2008) was concluded on 14 Sep. 2009. INTERPOL-UN Security Council Special Notice web link: https://www.interpol.int/en/How-we-work/Notices/View-UN-Notices-Individuals click here

QDi.014 Name: 1: TARIQ 2: ANWAR 3: EL SAYED 4: AHMED
Name (original script): طاريق أنور السيد احمد
Title: na **Designation:** na **DOB:** 15 Mar. 1963 **POB:** Alexandria, Egypt **Good quality a.k.a.: a)** Hamdi Ahmad Farag **b)** Amr Al-Fatih Fathi **c)** Tarek Anwar El Sayed Ahmad **Low quality a.k.a.:** na **Nationality:** Egypt **Passport no:** na **National identification no:** na **Address:** na **Listed on:** 6 Oct. 2001 (amended on 26 Nov. 2004, 18 Jul. 2007, 16 May 2011, 1 May 2019) **Other information:** Reportedly deceased in October 2001. Review pursuant to Security Council resolution 1822 (2008) was concluded on 29 Jul. 2010. Review pursuant to Security Council resolution 2253 (2015) was concluded on 21 Feb. 2019. Review pursuant to Security Council resolution 2368 (2017) was concluded on 15 November 2021. INTERPOL-UN Security Council Special Notice web link: https://www.interpol.int/en/How-we-work/Notices/View-UN-Notices-Individuals click here

QDi.193 Name: 1: ZAKI 2: EZAT 3: ZAKI 4: AHMED
Name (original script): زكي عزت زكي احمد
Title: na **Designation:** na **DOB:** 21 Apr. 1960 **POB: a)** Sharqiyah, Egypt **b)** Zaqaziq, Egypt **Good quality a.k.a.: a)** Rif'at Salim **b)** Abu Usama **Low quality a.k.a.:** na **Nationality:** Egypt **Passport no:** na **National identification no:** na **Address:** (May be on the Pakistani-Afghan border) **Listed on:** 29 Sep. 2005 (amended on 13 Dec. 2011, 1 May 2019) **Other information:** Father' s name is Ahmed Ezat Zaki. Member of Egyptian Islamic Jihad (QDe.003). Review pursuant to Security Council resolution 1822 (2008) was concluded on 1 Jun. 2010. Review pursuant to Security Council resolution 2253 (2015) was concluded on 21 Feb. 2019. Review pursuant to Security Council resolution 2368 (2017) was concluded on 15 November 2021. INTERPOL-UN Security Council Special Notice web link: https://www.interpol.int/en/How-we-work/Notices/View-UN-Notices-Individuals click here

QDi.313 Name: 1: DJAMEL 2: AKKACHA 3: na 4: na
Name (original script): جمال عكاشة
Title: na **Designation:** na **DOB:** 9 May 1978 **POB:** Rouiba, Algiers, Algeria **Good quality a.k.a.: a)** Yahia Abou el Hoummam **b)** Yahia Abou el Hammam **Low quality a.k.a.:** na **Nationality:** Algeria **Passport no:** na **National identification no:** na **Address:** Mali **Listed on:** 5 Feb. 2013 (amended on 1 May 2019, 2 Feb. 2023) **Other information:** Father' s name is Slimane. Mother' s name is Akrouf Khadidja. Coordinator of groups associated with The Organisation of Al-Qaida in the Islamic Maghreb (QDe.014) in northern Mali. Review pursuant to Security Council resolution 2253 (2015) was concluded on 21 Feb. 2019.Reportedly deceased in February 2019. INTERPOL-UN Security Council Special Notice web link: https://www.interpol.int/en/How-we-work/Notices/View-UN-Notices-Individuals click here

QDi.325 Name: 1: ABOU 2: MOHAMED 3: AL ADNANI 4: na

Title: na **Designation:** na **DOB:** Approximately 1977 **POB:** Binnish, Syrian Arab Republic **Good quality a.k.a.: a)** Yaser Khalaf Nazzal Alrawi **b)** Jaber Taha Falah **c)** Abou Khattab **d)** Abou Sadeq Alrawi **e)** Tah al Binchi **f)** Abu Mohammed al-Adnani **g)** Taha Sobhi Falaha **h)** Yasser Khalaf Hussein Nazal al-Rawi **i)** Abu Baker al-Khatab **j)** Abu Sadek al-Rawi **k)** Taha al-Banshi **l)** Abu Mohamed al-Adnani **m)** Abu-Mohammad al-Adnani al-Shami **n)** Hajj Ibrahim **Low quality a.k.a.:** na **Nationality:** Iraq **Passport no:** na **National identification no:** na **Address:** na **Listed on:** 15 Aug. 2014 (amended on 1 May 2019) **Other information:** Official spokesman of Islamic State in Iraq and the Levant (ISIL), listed as Al-Qaida in Iraq (QDe.115), and emir of ISIL in Syria, closely associated with Abu Mohammed al-Jawlani (QDi.317) and Abu Bakr al-Baghdadi, listed as Ibrahim Awwad Ibrahim Ali al-Badri al-Samarrai (QDi.299). Review pursuant to Security Council resolution 2253 (2015) was concluded on 21 Feb. 2019. Review pursuant to Security Council resolution 2368 (2017) was concluded on 15 November 2021. INTERPOL-UN Security Council Special Notice web link: https://www.interpol.int/en/How-we-work/Notices/View-UN-Notices-Individuals click here

QDi.328 Name: 1: HAJJAJ 2: BIN 3: FAHD 4: AL AJMI
Title: na **Designation:** na **DOB:** 10 Aug. 1987 **POB:** Kuwait **Good quality a.k.a.: a)** Hijaj Fahid Hijaj Muhammad Sahib al-Ajmi **b)** Hicac Fehid Hicac Muhammed Sebib al-Acmi **c)** Hajjaj bin-Fahad al-Ajmi **d)** Sheikh Hajaj al-Ajami **e)** Hajaj al-Ajami **f)** Ajaj Ajami **Low quality a.k.a.:** na **Nationality:** Kuwait **Passport no:** na **National identification no:** na **Address:** na **Listed on:** 15 Aug. 2014 (amended on 24 Nov. 2020) **Other information:** A Kuwait-based facilitator in charge of the ‘committee of zakat’ and financier for Al-Nusrah Front for the People of the Levant (QDe.137). Review pursuant to Security Council resolution 2368 (2017) was concluded on 24 November 2020. INTERPOL-UN Security Council Special Notice web link: https://www.interpol.int/en/How-we-work/Notices/View-UN-Notices-Individuals click here

QDi.324 Name: 1: ABDUL MOHSEN 2: ABDALLAH 3: IBRAHIM 4: AL CHAREKH
Title: na **Designation:** na **DOB:** 13 Jul. 1985 **POB:** Saqra, Saudi Arabia **Good quality a.k.a.: a)** Abdul Mohsen Abdullah Ibrahim Al-Sharikh **b)** Sanafi al Nasr **Low quality a.k.a.:** na **Nationality:** Saudi Arabia **Passport no:** na **National identification no:** na **Address:** na **Listed on:** 15 Aug. 2014 (amended on 1 May 2019) **Other information:** A long time facilitator and financier for Al-Qaida (QDe.004), appointed as a regional leader of Jabhat al-Nusrah, listed as Al-Nusrah Front for the People of the Levant (QDe.137). Review pursuant to Security Council resolution 2253 (2015) was concluded on 21 Feb. 2019. Review pursuant to Security Council resolution 2368 (2017) was concluded on 15 November 2021. INTERPOL-UN Security Council Special Notice web link: https://www.interpol.int/en/How-we-work/Notices/View-UN-Notices-Individuals click here

QDi.228 Name: 1: MOHAMMED 2: AL GHABRA 3: na 4: na
Title: na **Designation:** na **DOB:** 1 Jun. 1980 **POB:** Damascus, Syrian Arab Republic **Good quality a.k.a.: a)** Mohammed El’ Ghabra **b)** Danial Adam **Low quality a.k.a.:** na **Nationality:** United Kingdom of Great Britain and Northern Ireland **Passport no:** British number 094629366 **National identification no:** na **Address:** East London, United Kingdom **Listed on:** 12 Dec. 2006 (amended on 13 Dec. 2011, 20 Jul. 2015, 1 May 2019) **Other information:** Father’ s name is Mohamed Ayman Ghabra. Mother’ s name is Dalal. Review pursuant to Security Council resolution 1822 (2008) was concluded on 5 Oct. 2009. Review pursuant to Security Council resolution 2253 (2015) was concluded on 21 Feb. 2019. Review pursuant to Security Council resolution 2610 (2021) was concluded on 8 November 2022. INTERPOL-UN Security Council Special Notice web link: https://www.interpol.int/en/How-we-work/Notices/View-UN-Notices-Individuals click here

QDi.429 Name: 1: MOHAMMAD 2: ALI 3: AL HABBO 4: na
Name (original script): محمد علي الحبو
Title: na **Designation:** na **DOB: a)** 1 Oct. 1983 **b)** 15 Mar. 1983 **c)** 1 Jan. 1980 **POB:** Raqqa, Syrian Arab Republic **Good quality a.k.a.: a)** Mohamad Abdulkarim **b)** Muhammad Abd-al-Karim **Low quality a.k.a.: a)** Al-Hebo **b)** Al-Habu **c)** Alhobo **d)** Habo **e)** Hebbo **f)** Habu **Nationality:** Syrian Arab Republic **Passport no:** Syrian Arab Republic number 00814L001424 **National identification no: a)**

Syrian Arab Republic 10716775 **b)** Syrian Arab Republic 2020316097 **c)** Syrian Arab Republic 2020409266 **Address: a)** Gazantiep, Turkey (since 2016) **b)** Raqqa, Syrian Arab Republic **Listed on:** 17 Jun. 2021 **Other information:** Turkey-based facilitator who provides financial services to, or in support of, Islamic State in Iraq and the Levant, listed as Al-Qaida in Iraq (QDe.115). INTERPOL-UN Security Council Special Notice web link: https://www.interpol.int/en/How-we-work/Notices/View-UN-Notices-Individuals click here

QDi.327 Name: 1: ABDELRAHMAN 2: MOUHAMAD ZAFIR 3: AL DABIDI 4: AL JAHANI
Title: na **Designation:** na **DOB: a)** 4 Dec. 1971 **b)** 1977 **POB:** Kharj, Saudi Arabia **Good quality a.k.a.: a)** Abd Al-Rahman Muhammad Zafir Al-Dubaysi Al-Juhni **b)** Abd Al-Rahman Muhammad Zafir al-Dubaysi al-Jahni **c)** Abd Al-Rahman Muhammad Zafir al-Dubaysi al-Jahani **d)** Abd Al-Rahman Muhammad Zafir al-Dubaysi al-Juhani **e)** Abdulrhman Mohammed D. Aljahani **f)** Abu al-Wafa' **g)** Abu Anas **h)** Abd al-Rahman Muhammad Zafir al-Dabisi al-Jahani **i)** Abu Wafa al-Saudi **j)** Abu al-Wafa **k)** Abd al-Rahman Muhammad Thafir al-Jahni **l)** Abd al-Rahman Muhammad al-Juhani **m)**) Abdelrahman Mouhamad Zafir al Dabissi Juhan **n)** Abdelrahman Mouhamad Zafir al Dabissi Juhani **Low quality a.k.a.:** Abou Wafa al Saoudi **Nationality:** Saudi Arabia **Passport no:** F508591 **National identification no:** Saudi Arabia 1027508157 **Address:** na **Listed on:** 15 Aug. 2014 (amended on 6 Dec. 2019) **Other information:** A member and regional commander of Jabhat al-Nusrah, listed as Al-Nusrah Front for the People of the Levant (QDe.137)and a facilitator of foreign recruits for that group. Review pursuant to Security Council resolution 2368 (2017) was concluded on 4 Dec. 2019 INTERPOL-UN Security Council Special Notice web link: https://www.interpol.int/en/How-we-work/Notices/View-UN-Notices-Individuals click here

QDi.420 Name: 1: TAHA 2: IBRAHIM 3: ABDALLAH BAKR 4: AL KHUWAYT
Name (original script): طه إبراهيم عبد الله بكر ال خويت
Title: na **Designation:** na **DOB:** Between 1965 and 1969 **POB:** Tall 'Afar, Iraq **Good quality a.k.a.: a)** Hajji Abdelnasser **b)** Hajji Abd al-Nasr **c)** Hajji 'Abd Al-Nasir (formerly listed as) **Low quality a.k.a.: a)** Taha al-Khuwayt **b)** Mullah Taha **c)** Mullah Khuwayt **Nationality:** Iraq **Passport no:** na **National identification no:** na **Address:** Prison in Iraqna **Listed on:** 19 Nov. 2018 (amended on 27 May 2022) **Other information:** Former ISIL governor of al-Jazira Province, military leader in the Syrian Arab Republic as well as member and chair of the ISIL Delegated Committee, which exercises administrative control of ISIL's affairs. In custody of Iraq since 2019. Photo available for inclusion in the INTERPOL-UN Security Council Special Notice. INTERPOL-UN Security Council Special Notice web link: https://www.interpol.int/en/How-we-work/Notices/View-UN-Notices-Individuals click here

QDi.361 Name: 1: AMRU 2: AL-ABSI 3: na 4: na
Title: na **Designation:** na **DOB:** Approximately 1979 **POB:** Saudi Arabia **Good quality a.k.a.: a)** Amr al Absi **b)** Abu al Athir Amr al Absi **Low quality a.k.a.: a)** Abu al-Athir **b)** Abu al-Asir **c)** Abu Asir **d)** Abu Amr al Shami **e)** Abu al-Athir al-Shami **f)** Abu-Umar al-Absi **Nationality:** na **Passport no:** na **National identification no:** na **Address:** Homs, Syrian Arab Republic (location as at Sep. 2015) **Listed on:** 29 Sep. 2015 (amended on 1 May 2019) **Other information:** Shura council member of Islamic State in Iraq and the Levant, listed as Al-Qaida in Iraq (AQI) (QDe.115) and in charge of ISIL's media arm. ISIL's provincial leader for Homs, Syrian Arab Republic as of mid-2014. Dubbed as the ISIL's "kidnapper-in-chief". Review pursuant to Security Council resolution 2253 (2015) was concluded on 21 Feb. 2019. INTERPOL-UN Security Council Special Notice web link: https://www.interpol.int/en/How-we-work/Notices/View-UN-Notices-Individuals click here

QDi.338 Name: 1: SHAFI 2: SULTAN 3: MOHAMMED 4: AL-AJMI
Title: Doctor **Designation:** na **DOB:** 1 Jan. 1973 **POB:** Warah, Kuwait **Good quality a.k.a.: a)** Shafi al-Ajmi **b)** Sheikh Shafi al-Ajmi **Low quality a.k.a.:** Shaykh Abu-Sultan **Nationality:** Kuwait **Passport no:** 0216155930 **National identification no:** na **Address:** Area 3, Street 327, Building 41, Al-Uqaylah, Kuwait **Listed on:** 23 Sep. 2014 (amended on 24 Nov. 2020) **Other information:** Fundraiser for Al-Nusrah Front for the People of the Levant (QDe.137). Review pursuant to Security Council resolution 2368 (2017) was concluded on 24 November 2020. INTERPOL-UN Security

Council Special Notice web link: https://www.interpol.int/en/How-we-work/Notices/View-UN-Notices-Individuals click here

QDi.236 Name: 1: HAMID 2: ABDALLAH 3: AHMAD 4: AL-ALI
Name (original script): حامد عبد الله أحمد العلي
Title: na **Designation:** na **DOB:** 20 Jan. 1960 **POB:** Kuwait **Good quality a.k.a.: a)** Dr. Hamed Abdullah Al-Ali **b)** Hamed Al-'Ali **c)** Hamed bin 'Abdallah Al-'Ali **d)** Hamid 'Abdallah Al-'Ali **e)** Hamid 'Abdallah Ahmad Al-'Ali **f)** Hamid bin Abdallah Ahmed Al-Ali **g)** Hamid Abdallah Ahmed Al-Ali **Low quality a.k.a.:** Abu Salim **Nationality:** Kuwait **Passport no:** Kuwait number 1739010, issued on 26 May 2003, issued in Kuwait (and expired on 25 May 2008) **National identification no:** Kuwait 260012001546 **Address:** Kuwait (residence as at Mar. 2009) **Listed on:** 16 Jan. 2008 (amended on 1 Jul. 2008, 23 Jul. 2008, 25 Jan. 2010, 1 May 2019, 2 Feb. 2023) **Other information:** Review pursuant to Security Council resolution 1822 (2008) was concluded on 14 Sep. 2009. Review pursuant to Security Council resolution 2253 (2015) was concluded on 21 Feb. 2019. INTERPOL-UN Security Council Special Notice web link: https://www.interpol.int/en/How-we-work/Notices/View-UN-Notices-Individuals click here

QDi.092 Name: 1: MEHREZ 2: BEN MAHMOUD 3: BEN SASSI 4: AL-AMDOUNI
Name (original script): محرز بن محمود بن ساسي العمدوني
Title: na **Designation:** na **DOB:** 18 Dec. 1969 **POB:** Asima-Tunis, Tunisia **Good quality a.k.a.: a)** Fabio Fusco born 25 May 1968 in Naples, Italy **b)** Fabio Fusco born 18 Dec. 1968 in Tunisia **c)** Fabio Fusco born 25 May 1968 in Algeria **d)** Mohamed Hassan **e)** Meherez Hamdouni **f)** Amdouni Mehrez ben Tah born 14 Jul. 1969 in Tunisia **g)** Meherez ben Ahdoud ben Amdouni **Low quality a.k.a.:** Abu Thale **Nationality:** Tunisia **Passport no:** Tunisian number G737411, issued on 24 Oct. 1990 (expired on 20 Sep. 1997) **National identification no:** na **Address:** Italy **Listed on:** 25 Jun. 2003 (amended on 26 Nov. 2004, 20 Dec. 2005, 17 Oct. 2007, 16 Sep. 2008, 24 Mar. 2009, 12 Jul. 2010, 16 May 2011, 6 Dec. 2019) **Other information:** Father’ s name is Mahmoud ben Sasi. Mother’ s name is Maryam bint al-Tijani. Inadmissible to the Schengen area. Review pursuant to Security Council resolution 1822 (2008) was concluded on 22 Apr. 2010. Review pursuant to Security Council resolution 2368 (2017) was concluded on 4 Dec. 2019 INTERPOL-UN Security Council Special Notice web link: https://www.interpol.int/en/How-we-work/Notices/View-UN-Notices-Individuals click here

QDi.389 Name: 1: ABU UBAYDAH 2: YUSUF 3: AL-ANABI 4: na
Title: na **Designation:** na **DOB:** 7 Feb. 1969 **POB:** Annaba, Algeria **Good quality a.k.a.: a)** Abou Obeida Youssef Al-Annabi **b)** Abu-Ubaydah Yusuf Al-Inabi **Low quality a.k.a.: a)** Mebrak Yazid **b)** Youcef Abu Obeida **c)** Mibrak Yazid **d)** Yousif Abu Obayda Yazid **e)** Yazid Mebrak **f)** Yazid Mabrak **g)** Yusuf Abu Ubaydah **h)** Abou Youcef **Nationality:** Algeria **Passport no:** na **National identification no:** na **Address:** Algeria **Listed on:** 29 Feb. 2016 **Other information:** A leader of the Organization of Al-Qaida in the Islamic Maghreb (AQIM) (QDe.014). Photo available for inclusion in the INTERPOL-UN Security Council Special Notice. Review pursuant to Security Council resolution 2368 (2017) was concluded on 15 November 2021. INTERPOL-UN Security Council Special Notice web link: https://www.interpol.int/en/How-we-work/Notices/View-UN-Notices-Individuals click here

QDi.060 Name: 1: MOHAMED 2: BEN BELGACEM 3: BEN ABDALLAH 4: AL-AOUADI
Name (original script): محمد بن بلقاسم بن عبد الله العوادي
Title: na **Designation:** na **DOB:** 11 Dec. 1974 **POB:** Tunis, Tunisia **Good quality a.k.a.: a)** Mohamed Ben Belkacem Aouadi **b)** Fathi Hannachi **Low quality a.k.a.:** na **Nationality:** Tunisia **Passport no:** (Tunisian passport number L 191609 issued on 28 Feb. 1996, expired on 27 Feb. 2001) **National identification no: a)** (04643632 issued on 18 Jun. 1999) **b)** (Italian Fiscal Code: DAOMMD74T11Z352Z) **Address:** 50th Street, Number 23, Zehrouni, Tunis, Tunisia **Listed on:** 24 Apr. 2002 (amended on 10 Apr. 2003, 26 Nov. 2004, 9 Sep. 2005, 20 Dec. 2005, 31 Jul. 2006, 7 Jun. 2007, 23 Dec. 2010, 24 Nov. 2014, 23 Feb. 2016, 6 Dec. 2019, 7 May 2020) **Other information:** Head of security wing of Ansar al-Shari'a in Tunisia (AAS-T) (QDe.143). Mother's name is Ourida Bint Mohamed. Deported from Italy to Tunisia on 1 Dec. 2004. Arrested in Tunisia in Aug. 2013.

Imprisoned in the civilian prison of Burj al- ‘Amiri on 13 Sep. 2013. Review pursuant to Security Council resolution 1822 (2008) was concluded on 22 Apr. 2010. Review pursuant to Security Council resolution 2368 (2017) was concluded on 4 Dec. 2019 INTERPOL-UN Security Council Special Notice web link: https://www.interpol.int/en/How-we-work/Notices/View-UN-Notices-Individuals click here

QDi.291 Name: 1: IBRAHIM 2: HASSAN 3: TALI 4: AL-ASIRI
Name (original script): إبراهيم حسن طالع العسيري
Title: na **Designation:** na **DOB: a)** 19 Apr. 1982 **b)** 18 Apr. 1982 **c)** (24/06/1402 (Hijri Calendar)) **POB:** Riyadh, Saudi Arabia **Good quality a.k.a.: a)** Ibrahim Hassan Tali Asiri (إبراهيم حسن طالع عسيري) **b)** Ibrahim Hasan Talea Aseeri **c)** Ibrahim Hassan al-Asiri **d)** Ibrahim Hasan Tali Asiri **e)** Ibrahim Hassan Tali Assiri **f)** Ibrahim Hasan Tali'A 'Asiri **g)** Ibrahim Hasan Tali al-'Asiri **h)** Ibrahim al-'Asiri **i)** Ibrahim Hassan Al Asiri **Low quality a.k.a.: a)** Abu Saleh **b)** Abosslah **c)** Abu-Salaah **Nationality:** Saudi Arabia **Passport no:** Saudi Arabia number F654645, issued on 30 Apr. 2005 (expired on 7 Mar. 2010. Issue date in Hijri Calendar 24/06/1426. Expiry date in Hijri Calendar 21/03/1431.) **National identification no:** Saudi Arabia civil identification number 1028745097 **Address:** Yemen **Listed on:** 24 Mar. 2011 (amended on 15 Apr. 2014, 15 Jun. 2015, 9 May 2018, 2 Feb. 2023) **Other information:** Operative and principal bomb maker of Al-Qaida in the Arabian Peninsula (AQAP) (QDe.129). Believed to be hiding in Yemen as at Mar. 2011. Wanted by Saudi Arabia. Reportedly deceased. Also associated with Nasir 'abd-al-Karim 'Abdullah Al-Wahishi (deceased), Qasim Yahya Mahdi al-Rimi (QDi.282), and Anwar Nasser Abdulla Al-Aulaqi (QDi.283) (deceased). Review pursuant to Security Council resolution 2368 (2017) was concluded on 15 November 2021. INTERPOL-UN Security Council Special Notice web link: https://www.interpol.int/en/How-we-work/Notices/View-UN-Notices-Individuals click here

QDi.283 Name: 1: ANWAR 2: NASSER 3: ABDULLA 4: AL-AULAQI
Name (original script): انور ناصر عبدالله العولقي
Title: na **Designation:** na **DOB: a)** 21 Apr. 1971 **b)** 22 Apr. 1971 **POB:** Las Cruces, New Mexico, United States of America **Good quality a.k.a.: a)** Anwar al-Aulaqi **b)** Anwar al-Awlaki **c)** Anwar al-Awlaqi **d)** Anwar Nasser Aulaqi **e)** Anwar Nasser Abdullah Aulaqi **f)** Anwar Nasser Abdulla Aulaqi **Low quality a.k.a.:** na **Nationality: a)** United States of America **b)** Yemeni **Passport no:** na **National identification no:** na **Address:** na **Listed on:** 20 Jul. 2010 (amended on 30 Nov. 2011, 1 May 2019) **Other information:** Confirmed to have died on 30 Sep. 2011 in Yemen. Review pursuant to Security Council resolution 2253 (2015) was concluded on 21 Feb. 2019. Review pursuant to Security Council resolution 2368 (2017) was concluded on 15 November 2021. INTERPOL-UN Security Council Special Notice web link: https://www.interpol.int/en/How-we-work/Notices/View-UN-Notices-Individuals click here

QDi.344 Name: 1: IBRAHIM 2: 'ISA HAJJI 3: MUHAMMAD 4: AL-BAKR
Name (original script): ابراهيم عيسى حاجي محمد البكر
Title: na **Designation:** na **DOB:** 12 Jul. 1977 **POB:** Qatar **Good quality a.k.a.: a)** Ibrahim 'Issa Haji Muhammad al-Bakar **b)** Ibrahim 'Isa Haji al-Bakr **c)** Ibrahim Issa Hijji Mohd Albaker **d)** Ibrahim Issa Hijji Muhammad al-Baker **e)** Ibrahim 'Issa al-Bakar **f)** Ibrahim al-Bakr **Low quality a.k.a.:** Abu-Khalil **Nationality:** Qatar **Passport no:** Qatar number 01016646, issued in Qatar (expired on 11 Jan. 2017) **National identification no:** Qatar identification number 27763401255 **Address:** Al Rayyan, Qatar **Listed on:** 23 Jan. 2015 (amended on 14 Nov. 2020, 24 Nov. 2020, 23 Mar. 2021) **Other information:** Facilitator who provides financial support for and financial services to and in support of Al-Qaida (QDe.004). Review pursuant to Security Council resolution 2368 (2017) was concluded on 24 November 2020. INTERPOL-UN Security Council Special Notice web link: https://www.interpol.int/en/How-we-work/Notices/View-UN-Notices-Individuals click here

QDi.231 Name: 1: SALEM 2: NOR ELDIN 3: AMOHAMED 4: AL-DABSKI
Name (original script): سالم نور الدين امحمد الدبيسكي
Title: na **Designation:** na **DOB:** 1963 **POB:** Tripoli, Libya **Good quality a.k.a.: a)** Abu Al-Ward **b)** Abdullah Ragab **Low quality a.k.a.: a)** Abu Naim **b)** Abdallah al- Masri **Nationality:** Libya **Passport**

no: a) Libya number 1990/345751 **b)** Libya number 345751 **National identification no:** Libya national identification 220334 **Address:** Bab Ben Ghasheer, Tripoli, Libyan Arab Jamahiriya **Listed on:** 8 Jun. 2007 (amended on 13 Dec. 2011, 1 May 2019) **Other information:** Mother's name is Kalthoum Abdul Salam al-Shaftari. Senior member of Libyan Islamic Fighting Group (QDe.011) and member of Al-Qaida (QDe.004). Review pursuant to Security Council resolution 1822 (2008) was concluded on 24 Nov. 2009. Review pursuant to Security Council resolution 2253 (2015) was concluded on 21 Feb. 2019. Review pursuant to Security Council resolution 2610 (2021) was concluded on 8 November 2022. INTERPOL-UN Security Council Special Notice web link: https://www.interpol.int/en/How-we-work/Notices/View-UN-Notices-Individuals click here

QDi.278 Name: 1: MUTHANNA 2: HARITH 3: AL-DARI 4: na
Name (original script): مثنى حارث الضاري
Title: Doctor **Designation:** na **DOB:** 16 Jun. 1969 **POB:** Iraq **Good quality a.k.a.: a)** Dr. Muthanna Al Dari **b)** Muthana Harith Al Dari **c)** Muthanna Harith Sulayman Al-Dari **d)** Muthanna Harith Sulayman Al-Dhari **e)** Muthanna Hareth Al-Dhari **f)** Muthana Haris Al-Dhari **g)** Doctor Muthanna Harith Sulayman Al Dari Al-Zawba' **h)** Muthanna Harith Sulayman Al-Dari Al-Zobai **i)** Muthanna Harith Sulayman Al-Dari al-Zawba'i **j)** Muthanna Hareth al-Dari **k)** Muthana Haris al-Dari **l)** Doctor Muthanna al-Dari **m)** Dr. Muthanna Harith al-Dari al-Zowbai **Low quality a.k.a.:** na **Nationality:** Iraq **Passport no:** na **National identification no:** Ration card number: 1729765 **Address: a)** Amman, Jordan **b)** Khan Dari, Iraq (previous) **c)** Asas Village, Abu Ghurayb, Iraq (previous) **d)** Egypt (previous) **Listed on:** 25 Mar. 2010 (amended on 10 Dec. 2015, 6 Dec. 2019) **Other information:** Mother’ s name: Heba Khamis Dari. Provided operational guidance financial support and other services to or in support of Islamic State in Iraq and the Levant, listed as Al-Qaida in Iraq (AQI) (QDe.115). Involved in oil smuggling. Wanted by the Iraqi security forces. Photo available for inclusion in the INTERPOL-UN Security Council Special Notice. Review pursuant to Security Council resolution 2368 (2017) was concluded on 4 Dec. 2019. INTERPOL-UN Security Council Special Notice web link: https://www.interpol.int/en/How-we-work/Notices/View-UN-Notices-Individuals click here

QDi.149 Name: 1: NOUREDDINE 2: BEN ALI 3: BEN BELKASSEM 4: AL-DRISSI
Name (original script): نور الدين بن علي بن بلقاسم الدريسي
Title: na **Designation:** na **DOB:** 30 Apr. 1964 **POB:** Tunis, Tunisia **Good quality a.k.a.:** Drissi Noureddine **Low quality a.k.a.: a)** Abou Ali **b)** Faycal **Nationality:** Tunisia **Passport no:** Tunisian number L851940, issued on 9 Sep. 1998 (expired on 8 Sep. 2003) **National identification no:** na **Address:** Via Plebiscito 3, Cremona, Italy **Listed on:** 12 Nov. 2003 (amended on 20 Dec. 2005, 31 Jul. 2006, 21 Dec. 2007, 16 May 2011, 6 Dec. 2019, 10 Sep. 2020) **Other information:** Sentenced to six years of imprisonment for international terrorism in 2008. Deported from Italy to Tunisia on 10 Feb. 2013. Inadmissible to the Schengen area. Mother’ s name is Khadijah al-Drissi. Review pursuant to Security Council resolution 1822 (2008) was concluded on 22 Apr. 2010. Review pursuant to Security Council resolution 2368 (2017) was concluded on 4 Dec. 2019. INTERPOL-UN Security Council Special Notice web link: https://www.interpol.int/en/How-we-work/Notices/View-UN-Notices-Individuals click here

QDi.379 Name: 1: ABD AL-AZIZ 2: ADAY 3: ZIMIN 4: AL-FADHIL
Name (original script): عبدالعزيز عدي زمين الفضيل
Title: na **Designation:** na **DOB:** 27 Aug. 1981 **POB:** Kuwait **Good quality a.k.a.: a)** Abd al-Aziz Udai Samin al-Fadhli **b)** Abd al-Aziz Udai Samin al-Fadhl **c)** Abd al-Aziz Adhay Zimin al-Fadhli **d)** Abdalaziz Ad'ai Samin Fadhli al-Fadhali **Low quality a.k.a.:** na **Nationality:** na **Passport no:** na **National identification no:** 281082701081 **Address:** na **Listed on:** 21 Sep. 2015 (amended on 1 May 2019) **Other information:** Kuwait-based facilitator who provides financial services to, or in support of, Al-Nusrah Front for the People of the Levant (QDe.137) and Al-Qaida in the Arabian Peninsula (AQAP) (QDe.129). Review pursuant to Security Council resolution 2253 (2015) was concluded on 21 Feb. 2019. INTERPOL-UN Security Council Special Notice web link: https://www.interpol.int/en/How-we-work/Notices/View-UN-Notices-Individuals click here

QDi.059 Name: 1: KHALID 2: ABD AL-RAHMAN 3: HAMD 4: AL-FAWAZ
Name (original script): خالد عبد الرحمن حمد الفواز
Title: na **Designation:** na **DOB:** 24 Aug. 1962 **POB:** Kuwait **Good quality a.k.a.: a)** Khaled Al-Fauwaz **b)** Khaled A. Al-Fauwaz **c)** Khalid Al-Fawwaz **d)** Khalik Al Fawwaz **e)** Khaled Al-Fawwaz **f)** Khaled Al Fawwaz **g)** Khalid Abdulrahman H. Al Fawaz **Low quality a.k.a.:** na **Nationality:** Saudi Arabia **Passport no:** 456682, issued on 6 Nov. 1990 (expired on 13 Sep. 1995) **National identification no:** na **Address:** United States of America **Listed on:** 24 Apr. 2002 (amended on 26 Nov. 2004, 23 Apr. 2007, 21 Oct. 2010, 4 Aug. 2014, 6 Dec. 2019) **Other information:** Extradited from the United Kingdom to the United States of America on 5 Oct. 2012. Review pursuant to Security Council resolution 1822 (2008) was concluded on 22 Apr. 2010.. Review pursuant to Security Council resolution 2368 (2017) was concluded on 4 Dec. 2019 INTERPOL-UN Security Council Special Notice web link: https://www.interpol.int/en/How-we-work/Notices/View-UN-Notices-Individuals click here

QDi.292 Name: 1: OTHMAN 2: AHMED 3: OTHMAN 4: AL-GHAMDI
Name (original script): عثمان أحمد عثمان الغامدي
Title: na **Designation:** na **DOB:** 27 May 1979 **POB:** Saudi Arabia **Good quality a.k.a.: a)** Othman al-Ghamdi born 27 May 1979 in Saudi Arabia **b)** Uthman al-Ghamdi born 27 May 1979 in Saudi Arabia **c)** Uthman al-Ghamidi born 27 May 1979 in Saudi Arabia **d)** Othman bin Ahmed bin Othman Alghamdi **e)** Othman Ahmed Othman Al Omairah (born in 1973 in Shabwa, Yemen, nationality: Yemeni) **f)** Uthman Ahmad Uthman al-Ghamdi **g)** Othman Ahmed Othman al-Omirah **Low quality a.k.a.: a)** Al Umairah al-Ghamdi **b)** Othman Bin Ahmed Bin Othman **Nationality:** Saudi Arabia **Passport no:** na **National identification no:** Saudi Arabia National Identification Number 1089516791 **Address:** Yemen **Listed on:** 16 Jun. 2011 (amended on 15 Apr. 2014, 24 Nov. 2020) **Other information:** Operational commander of Al-Qaida in the Arabian Peninsula (AQAP) (QDe.129). Has been involved in raising funds and stockpiling arms for AQAP operations and activities in Yemen. Known associate of Qasim Yahya Mahdi al-Rimi (QDi.282) and Fahd Mohammed Ahmed al-Quso (deceased). Father' s name is Ahmed Othman Al Omirah. Review pursuant to Security Council resolution 2368 (2017) was concluded on 24 November 2020. INTERPOL-UN Security Council Special Notice web link: https://www.interpol.int/en/How-we-work/Notices/View-UN-Notices-Individuals click here

QDi.332 Name: 1: IBRAHIM 2: SULEIMAN 3: HAMAD 4: AL-HABLAIN
Title: na **Designation:** na **DOB:** 17 Dec. 1984 **POB:** Buraidah, Saudi Arabia **Good quality a.k.a.:** Barahim Suliman H. al Hblian **Low quality a.k.a.: a)** Abu Jabal **b)** Abu-Jabal **Nationality:** Saudi Arabia **Passport no:** Saudi Arabia number F800691 **National identification no:** 1047503170 **Address:** na **Listed on:** 23 Sep. 2014 (amended on 6 Dec. 2019, 10 Sep. 2020) **Other information:** Explosives expert and operative for the Abdallah Azzam Brigades (AAB) (QDe.144). Wanted by the Saudi Arabian Government for terrorism. Physical description: eye colour: dark; hair colour: dark; complexion: olive. Speaks Arabic. Photo available for inclusion in the INTERPOL-UN Security Council Special Notice. Review pursuant to Security Council resolution 2368 (2017) was concluded on 4 Dec. 2019. INTERPOL-UN Security Council Special Notice web link: https://www.interpol.int/en/How-we-work/Notices/View-UN-Notices-Individuals click here

QDi.140 Name: 1: KAMAL 2: BEN MAOELDI 3: BEN HASSAN 4: AL-HAMRAOUI
Name (original script): كمال بن المولدي بن حسن الحمراوي
Title: na **Designation:** na **DOB:** 21 Oct. 1977 **POB:** Beja, Tunisia **Good quality a.k.a.: a)** Hamroui Kamel ben Mouldi **b)** Hamraoui Kamel born 21 Nov. 1977 in Morocco **c)** Hamraoui Kamel born 21 Nov. 1977 in Tunisia **d)** Hamraoui Kamel born 21 Oct. 1977 in Tunisia **Low quality a.k.a.: a)** Kamel **b)** Kimo **Nationality:** Tunisia **Passport no:** Tunisian number P229856, issued on 1 Nov. 2002 (expires on 31 Oct. 2007) **National identification no:** na **Address: a)** Via Bertesi Number 27, Cremona, Italy **b)** Via Plebiscito Number 3, Cremona, Italy **Listed on:** 12 Nov. 2003 (amended on 20 Dec. 2005, 31 Jul. 2006, 21 Dec. 2007, 16 May 2011, 6 Dec. 2019, 10 Sep. 2020) **Other**

information: Mother’ s name is Khamisah al-Kathiri. Subject to a decree of expulsion, suspended on 17 Apr. 2007 by the European Court of Human Rights. Re-arrested in Italy on 20 May 2008. Deported from Italy to Tunisia on 6 May 2015. Inadmissible to the Schengen area. Review pursuant to Security Council resolution 1822 (2008) was concluded on 6 May 2010. Review pursuant to Security Council resolution 2368 (2017) was concluded on 4 Dec. 2019. INTERPOL-UN Security Council Special Notice web link: https://www.interpol.int/en/How-we-work/Notices/View-UN-Notices-Individuals click here

QDi.399 Name: 1: BASSAM 2: AHMAD 3: AL-HASRI 4: na
Name (original script): بسام أحمد الحصري
Title: na **Designation:** na **DOB: a)** 1 Jan. 1969 **b)** Approximately 1971 **POB: a)** Qalamun, Damascus Province, Syrian Arab Republic **b)** Ghutah, Damascus Province, Syrian Arab Republic **c)** Tadamon, Rif Dimashq, Syrian Arab Republic **Good quality a.k.a.:** Bassam Ahmad Husari **Low quality a.k.a.: a)** Abu Ahmad Akhlaq **b)** Abu Ahmad al-Shami **Nationality: a)** Syrian Arab Republic **b)** State of Palestine **Passport no:** na **National identification no:** na **Address:** Syrian Arab Republic (Southern. Location as of July 2016) **Listed on:** 22 Feb. 2017 **Other information:** Leader of Al-Nusrah Front for the People of the Levant (QDe.137) for southern Syrian Arab Republic since July 2016. INTERPOL-UN Security Council Special Notice web link: https://www.interpol.int/en/How-we-work/Notices/View-UN-Notices-Individuals click here

QDi.317 Name: 1: ABU MOHAMMED 2: AL-JAWLANI 3: na 4: na
Name (original script): أبو محمد الجولاني
Title: na **Designation:** na **DOB:** Between 1975 and 1979 **POB:** Syrian Arab Republic **Good quality a.k.a.: a)** Abu Mohamed al-Jawlani (Abu Muhammad al-Jawlani, Abu Mohammed al-Julani, Abu Mohammed al-Golani, Abu Muhammad al-Golani, Abu Muhammad Aljawlani, Muhammad al-Jawlani (transliterations of original script name)) **b)** Amjad Muzaffar Hussein Ali al-Naimi born 1980 in Syrian Arab Republic ((Mother’ s name: Fatma Ali Majour. Address: Mosul, Souq al-Nabi Yunis) **Low quality a.k.a.: a)** شيخ الفاتح ، الفاتح (transliterations: Shaykh al-Fatih; Al Fatih) (Translation: The Conqueror) (Nom de guerre) **b)** Abu Ashraf **Nationality:** Syrian Arab Republic **Passport no:** na **National identification no:** na **Address:** (Active in Syria as at Jun. 2013) **Listed on:** 24 Jul. 2013 (amended on 2 Jun. 2014, 10 Dec. 2015, 1 May 2019) **Other information:** Description: Dark complexion. Height: 1.70 m. Since Jan. 2012, he is the Leader of Al-Nusrah Front for the People of the Levant (QDe.137), a Syria-based group listed in May 2014, and previously listed as an alias of Al-Qaida in Iraq (AQI) (QDe.115) between 30 May 2013 and 13 May 2014. Associated with Aiman Muhammed Rabi al-Zawahiri (QDi.006). Wanted by the Iraqi security forces. Photo available for inclusion in the INTERPOL-UN Security Council Special Notice. Review pursuant to Security Council resolution 2253 (2015) was concluded on 21 Feb. 2019. Review pursuant to Security Council resolution 2610 (2021) was concluded on 8 November 2022. INTERPOL-UN Security Council Special Notice web link: https://www.interpol.int/en/How-we-work/Notices/View-UN-Notices-Individuals click here

QDi.337 Name: 1: MAYSAR ALI 2: MUSA 3: ABDALLAH 4: AL-JUBURI
Title: Amir **Designation:** na **DOB:** 1 Jun. 1976 **POB: a)** Al-Shura, Mosul, Iraq **b)** Harara, Ninawa Province, Iraq **Good quality a.k.a.: a)** Muyassir al-Jiburi **b)** Muyassir Harara **c)** Muyassir al-Shammari **d)** Muhammad Khalid Hassan **Low quality a.k.a.: a)** Al-Shammari **b)** Mus'ab al-Qahtani **c)** Abu Maria al-Qatani **Nationality:** Iraq **Passport no:** na **National identification no:** na **Address:** na **Listed on:** 23 Sep. 2014 (amended on 6 Dec. 2019) **Other information:** Sharia amir of Al-Nusrah Front for the People of the Levant (QDe.137) as of early 2014. Review pursuant to Security Council resolution 2368 (2017) was concluded on 4 Dec. 2019 INTERPOL-UN Security Council Special Notice web link: https://www.interpol.int/en/How-we-work/Notices/View-UN-Notices-Individuals click here

QDi.382 Name: 1: SA'D 2: BIN SA'D 3: MUHAMMAD SHARIYAN 4: AL-KA'BI
Name (original script): سعد بن سعد محمد شريان الكعبي

Title: na **Designation:** na **DOB:** 15 Feb. 1972 **POB:** na **Good quality a.k.a.: a)** Sa'd bin Sa'd Muhammad Shiryan al-Ka'bi **b)** Sa'd Sa'd Muhammad Shiryan al-Ka'bi **c)** Sa'd al-Sharyan al-Ka'bi **Low quality a.k.a.: a)** Abu Haza' **b)** Abu Hazza' **c)** Umar al-Afghani **d)** Abu Sa'd **e)** Abu Suad **Nationality:** Qatar **Passport no:** Qatar number 00966737 (expired 16 Feb. 2016) **National identification no:** Qatar 27263401275 **Address:** Umm Salal, Qatar **Listed on:** 21 Sep. 2015 (amended on 1 May 2019, 23 Mar. 2021) **Other information:** Qatar-based facilitator who provides financial services to, or in support of, Al-Nusrah Front for the People of the Levant (QDe.137). Review pursuant to Security Council resolution 2253 (2015) was concluded on 21 Feb. 2019. INTERPOL-UN Security Council Special Notice web link: https://www.interpol.int/en/How-we-work/Notices/View-UN-Notices-Individuals click here

QDi.318 Name: 1: MUHAMMAD 2: JAMAL 3: ABD-AL RAHIM AHMAD 4: AL-KASHIF
Name (original script): محمد جمال عبدالرحيم أحمد الكاشف
Title: na **Designation:** na **DOB: a)** 1 Jan. 1964 **b)** 1 Feb. 1964 **POB:** Cairo, Egypt **Good quality a.k.a.: a)** Muhammad Jamal Abdo Al-Kashif **b)** Muhammad Jamal Abdo Al Kashef **c)** Muhammad Jamal Abd-Al Rahim Ahmad Al-Kashif **d)** Muhammad Jamal Abd-Al Rahim Al-Kashif **e)** Muhammad Jamal Abdu **f)** Muhammad Jamal **Low quality a.k.a.: a)** Muhammad Jamal Abu Ahmad (nom de guerre) **b)** Abu Ahmad (nom de guerre) **c)** Abu Jamal (nom de guerre) **d)** Muhammad Gamal Abu Ahmed **e)** Mohammad Jamal Abdo Ahmed (nom de guerre) **f)** Muhammad Jamal Abduh (nom de guerre) **g)** Muhammad Jamal Ahmad Abdu (nom de guerre) **h)** Riyadh (nom de guerre) **Nationality:** Egypt **Passport no: a)** Egypt number 6487, issued on 30 Jan. 1986 (issued under name Muhammad Jamal Abdu) **b)** Egypt (issued in 1993, under name Muhammad Jamal Abd-Al Rahim Ahmad Al-Kashif) **c)** Yemen number 388181 (issued under name Muhammad Jamal Abd-Al Rahim Al-Kashif) **National identification no:** na **Address:** Egypt **Listed on:** 21 Oct. 2013 (amended on 1 May 2019) **Other information:** rained in Afghanistan in the late 1980s with Al-Qaida (QDe.004) to make bombs. Former top military commander of the Egyptian Islamic Jihad (QDe.003). Since 2011, established Muhammad Jamal Network (MJN) (QDe.136) and terrorist training camps in Egypt and Libya. Conducted MJN' s terrorist activities with support from Al-Qaida in the Arabian Peninsula (AQAP) (QDe.129). Reported to be involved in the attack on the United States Mission in Benghazi, Libya, on 11 Sep. 2012. Headed Nasr City terrorist cell in Egypt in 2012. Linked to Aiman al-Zawahiri (QDi.006) and the leadership of AQAP and the Organization of Al-Qaida in the Islamic Maghreb (AQIM) (QDe.014). Arrested and imprisoned multiple times by Egyptian authorities since ca. 2000. Released in 2011 but re-arrested by Egyptian authorities in Nov. 2012. Imprisoned in Egypt pending trial as of Sep. 2013. Wife' s name is Samah 'Ali Al-Dahabani (Yemeni national). Review pursuant to Security Council resolution 2253 (2015) was concluded on 21 Feb. 2019. Review pursuant to Security Council resolution 2610 (2021) was concluded on 8 November 2022. INTERPOL-UN Security Council Special Notice web link: https://www.interpol.int/en/How-we-work/Notices/View-UN-Notices-Individuals click here

QDi.380 Name: 1: ABD AL-LATIF 2: BIN ABDALLAH 3: SALIH MUHAMMAD 4: AL-KAWARI
Name (original script): عبداللطيف بن عبدلله صالح محمد الكواري
Title: na **Designation:** na **DOB:** 28 Sep. 1973 **POB:** na **Good quality a.k.a.: a)** Abd-al-Latif Abdallah Salih al-Kawari **b)** Abd-al-Latif Abdallah Salih al-Kuwari **c)** Abd-al-Latif Abdallah al-Kawwari **d)** Abd-al-Latif Abdallah al-Kawari **e)** Abu Ali al-Kawari **Low quality a.k.a.:** na **Nationality:** Qatar **Passport no: a)** Qatar number 01020802 **b)** Qatar number 00754833, issued on 20 May 2007 **c)** Qatar number 00490327, issued on 28 Jul. 2001 **d)** Qatar number 01538029 (expires 14 Mar. 2025) **National identification no:** Qatar 27363400684 **Address:** Al Kharaitiyat, Qatar **Listed on:** 21 Sep. 2015 (amended on 1 May 2019, 23 Mar. 2021) **Other information:** Qatar-based facilitator who provides financial services to, or in support of, Al-Qaida (QDe.004). Review pursuant to Security Council resolution 2253 (2015) was concluded on 21 Feb. 2019. INTERPOL-UN Security Council Special Notice web link: https://www.interpol.int/en/How-we-work/Notices/View-UN-Notices-Individuals click here

QDi.412 Name: 1: UMAR 2: MAHMUD 3: IRHAYYIM 4: AL-KUBAYSI

Name (original script): عمر محمود إرحيم الفياض الكبيسي
Title: na **Designation:** na **DOB:** **a)** 16 Jun. 1967 **b)** 1 Jan. 1967 **POB:** Al-Qaim, Al-Anbar Province, Iraq **Good quality a.k.a.:** **a)** Umar Mahmud Rahim al-Kubaysi **b)** Omar Mahmood Irhayyim Al-Fayyadh **c)** Umar Mahmud Rahim **d)** Umar Mahmud Rahim Al-Qubaysi **e)** Umar Mahmud Al-Kubaysi Arhaym **f)** Umar Mahmud Arhaym **g)** Omar Mahmood Irhayyim **h)** Omar Mahmood Irhayyim Al-Fayyadh Al-Kobaisi **i)** Umar al-Kubaysi **Low quality a.k.a.:** na **Nationality:** Iraq **Passport no:** Iraq number A4059346, issued on 29 May 2013, issued in Baghdad, Iraq (expires on 27 May 2021) **National identification no:** **a)** Iraq national identification card 00405771, issued on 20 May 2013, issued in Iraq (name in Arabic script: عمر محمود إرحيم الفياض) **b)** Iraq Certificate of Iraqi Nationality 540763, issued on 13 Feb. 1984 (name in Arabic script: عمر محمود إرحيم) **Address:** Al-Qaim, Al-Anbar Province, Iraq **Listed on:** 6 Mar. 2018 **Other information:** Financial facilitator for Islamic State in Iraq and the Levant, listed as Al-Qaida in Iraq (QDe.115). Director of Al-Kawthar Money Exchange (QDe.157). Physical description: sex: male, hair colour: black; height: 175 cm. Speaks Arabic. INTERPOL-UN Security Council Special Notice web link: https://www.interpol.int/en/How-we-work/Notices/View-UN-Notices-Individuals click here

QDi.177 Name: 1: HABIB 2: BEN 3: AHMED 4: AL-LOUBIRI
Name (original script): حبيب بن احمد اللوبيري
Title: na **Designation:** na **DOB:** 17 Nov. 1961 **POB:** Manzal Tmim, Nabul, Tunisia **Good quality a.k.a.:** Al-Habib ben Ahmad ben al-Tayib al-Lubiri **Low quality a.k.a.:** na **Nationality:** Tunisia **Passport no:** Tunisian number M788439, issued on 20 Oct. 2001 (expires on 19 Oct. 2006) **National identification no:** 01817002 **Address:** Salam Marnaq Ben Arous district, Sidi Mesoud, Tunisia **Listed on:** 23 Jun. 2004 (amended on 20 Dec. 2005, 17 Oct. 2007, 10 Aug. 2009, 13 Dec. 2011, 23 Feb. 2016, 6 Dec. 2019) **Other information:** Italian Fiscal Code: LBR HBB 61S17 Z352F. In detention in Tunisia as at Dec. 2009. Mother' s name is Fatima al-Galasi. Review pursuant to Security Council resolution 1822 (2008) was concluded on 9 Apr. 2010. Review pursuant to Security Council resolution 2368 (2017) was concluded on 4 Dec. 2019 INTERPOL-UN Security Council Special Notice web link: https://www.interpol.int/en/How-we-work/Notices/View-UN-Notices-Individuals click here

QDi.074 Name: 1: TAREK 2: BEN HABIB 3: BEN AL-TOUMI 4: AL-MAAROUFI
Name (original script): طارق بن الحبيب بن التومي المعروفي
Title: na **Designation:** na **DOB:** 23 Nov. 1965 **POB:** Ghardimaou, Tunisia **Good quality a.k.a.:** **a)** Abu Ismail **b)** Abou Ismail el Jendoubi **c)** Abou Ismail Al Djoundoubi **Low quality a.k.a.:** na **Nationality:** Tunisia **Passport no:** Tunisian number E590976, issued on 19 Jun. 1987 (expired on 18 Jun. 1992) **National identification no:** na **Address:** Rue Léon Théodore Number 107/1, 1090 Jette, Brussels, Belgium **Listed on:** 3 Sep. 2002 (amended on 26 Nov. 2004, 20 Dec. 2005, 31 Jul. 2006, 3 Jul. 2007, 10 Aug. 2009, 25 Jan. 2010, 23 Dec. 2010, 6 Dec. 2019) **Other information:** Belgian nationality withdrawn on 26 Jan. 2009. In detention in Nivelles, Belgium, as of Oct. 2010. Review pursuant to Security Council resolution 1822 (2008) was concluded on 8 Jun. 2010. Review pursuant to Security Council resolution 2368 (2017) was concluded on 4 Dec. 2019 INTERPOL-UN Security Council Special Notice web link: https://www.interpol.int/en/How-we-work/Notices/View-UN-Notices-Individuals click here

QDi.411 Name: 1: SALIM 2: MUSTAFA 3: MUHAMMAD 4: AL-MANSUR
Name (original script): سالم مصطفى محمد ال منصور
Title: na **Designation:** na **DOB:** **a)** 20 Feb. 1962 **b)** 1959 **POB:** **a)** Baghdad, Iraq **b)** Tel Afar, Nineveh Province, Iraq **Good quality a.k.a.:** **a)** Salim Mustafa Muhammad Mansur Al-Ifri **b)** Saleem Al-Ifri **c)** Salim Mansur Mustafa **d)** Salim Mansur **e)** Hajji Salim Al-Shaklar **Low quality a.k.a.:** na **Nationality:** Iraq **Passport no:** Iraq number A6489694, issued on 2 Sep. 2013 (expires on 31 Aug. 2021; name in Arabic script: سالم مصطفى محمد ال منصور) **National identification no:** **a)** Iraq national identification card 00813602, issued on 18 Sep. 2011 (name in Arabic script: سالم مصطفى محمد ال منصور) **b)** Iraq Certificate of Iraqi Nationality 300397, issued on 25 Jun. 2013 (name in Arabic script: سالم مصطفى محمد) **Address:** **a)** 17 Tamoz, Mosul, Iraq (previous address) **b)** Tel Afar – Al-Saad, Mosul, Iraq

(previous address) **Listed on:** 6 Mar. 2018 **Other information:** Finance "emir" for Islamic State in Iraq and the Levant, listed as Al-Qaida in Iraq (QDe.115). Physical description: hair colour: black; eye colour: honey; height: 170 cm. Speaks Arabic. INTERPOL-UN Security Council Special Notice web link: https://www.interpol.int/en/How-we-work/Notices/View-UN-Notices-Individuals click here

QDi.320 Name: 1: ABD-AL-HAMID 2: AL-MASLI 3: na 4: na
Name (original script): عبدالحميد المصلي
Title: na **Designation:** na **DOB:** 1976 **POB:** **a)** Darnah, Libya **b)** Danar, Libya **Good quality a.k.a.:** **a)** Abd-al-Hamid Muhammad Abd-al-Hamid Al-Masli **b)** Abd-al-Hamid Musalli **c)** Hamid Masli **Low quality a.k.a.:** **a)** Hamza al-Darnawi **b)** Hamzah al-Darnawi **c)** Hamza Darnawi **d)** Hamzah Darnawi **e)** Hamzah Dirnawi **f)** Hamza Darnavi **g)** Hamza al-Darnavi **h)** Abdullah Darnawi **i)** Abu-Hamzah al-Darnawi **Nationality:** Libya **Passport no:** na **National identification no:** na **Address:** (Reportedly located in Waziristan, Federally Administered Tribal Areas, Pakistan) **Listed on:** 26 Nov. 2013 (amended on 1 May 2019) **Other information:** Leader and trainer of an Al-Qaida electronics and explosives workshop producing improvised explosive device components. Review pursuant to Security Council resolution 2253 (2015) was concluded on 21 Feb. 2019. Review pursuant to Security Council resolution 2610 (2021) was concluded on 8 November 2022. INTERPOL-UN Security Council Special Notice web link: https://www.interpol.int/en/How-we-work/Notices/View-UN-Notices-Individuals click here

QDi.330 Name: 1: AZZAM 2: ABDULLAH 3: ZUREIK 4: AL-MAULID AL-SUBHI
Title: na **Designation:** na **DOB:** 12 Apr. 1976 **POB:** Al Baraka, Saudi Arabia **Good quality a.k.a.:** **a)** Mansur al-Harbi **b)** Azzam al-Subhi **c)** Azam Abdallah Razeeq al Mouled Alsbhua **d)** Abu Muslem al-Maky **e)** Abu Suliman al-Harbi **f)** Abu Abdalla al-Harbi **g)** Azam A.R. Alsbhua **Low quality a.k.a.:** na **Nationality:** Saudi Arabia **Passport no:** Saudi Arabia number C389664, issued on 15 Sep. 2000 **National identification no:** 1024026187 **Address:** na **Listed on:** 23 Sep. 2014 (amended on 6 Dec. 2019, 10 Sep. 2020) **Other information:** Has ties to numerous senior Al-Qaida (QDe.004) leaders. Wanted by the Saudi Arabian Government for terrorism. Father's name is Abdullah Razeeq al Mouled al Sbhua. Physical description: eye colour: dark; hair colour: dark; complexion: dark. Speaks Arabic. Photo available for inclusion in the INTERPOL-UN Security Council Special Notice. Review pursuant to Security Council resolution 2368 (2017) was concluded on 4 Dec. 2019. INTERPOL-UN Security Council Special Notice web link: https://www.interpol.int/en/How-we-work/Notices/View-UN-Notices-Individuals click here

QDi.276 Name: 1: AKRAM 2: TURKI 3: HISHAN 4: AL-MAZIDIH
Name (original script): أكرم تركي هاشم المزيده
Title: na **Designation:** na **DOB:** **a)** 1974 **b)** 1975 **POB:** na **Good quality a.k.a.:** Akram Turki Al-Hishan **Low quality a.k.a.:** **a)** Abu Jarrah **b)** Abu Akram **Nationality:** na **Passport no:** na **National identification no:** na **Address:** **a)** Deir ez-Zor Governorate, Syrian Arab Republic **b)** Iraq **Listed on:** 11 Mar. 2010 (amended on 15 Jan. 2016, 10 Dec. 2015, 1 May 2019) **Other information:** Other possible date of birth: 1979. He is a cousin of Ghazy Fezza Hishan Al Mazidih (QDi.277). Financial facilitator of the Islamic State in Iraq and the Levant, listed as Al-Qaida in Iraq (AQI) (QDe.115) as of 2015. Review pursuant to Security Council resolution 2253 (2015) was concluded on 21 Feb. 2019. Review pursuant to Security Council resolution 2610 (2021) was concluded on 8 November 2022. INTERPOL-UN Security Council Special Notice web link: https://www.interpol.int/en/How-we-work/Notices/View-UN-Notices-Individuals click here

QDi.277 Name: 1: GHAZY 2: FEZZA 3: HISHAN 4: AL-MAZIDIH
Name (original script): غازي فيزا هاشم المزيده
Title: na **Designation:** na **DOB:** **a)** 1974 **b)** 1975 **POB:** na **Good quality a.k.a.:** **a)** Ghazy Fezzaa Hishan **b)** Mushari Abd Aziz Saleh Shlash **Low quality a.k.a.:** **a)** Abu Faysal **b)** Abu Ghazzy **Nationality:** na **Passport no:** na **National identification no:** na **Address:** **a)** Syrian Arab Republic **b)** Iraq **Listed on:** 11 Mar. 2010 (amended on 10 Dec. 2015, 1 May 2019) **Other information:** He is a cousin of Akram Turki Hishan Al Mazidih (QDi.276). Terrorist attack organizer

for the Islamic State in Iraq and the Levant, listed as Al-Qaida in Iraq (AQI) (QDe.115) as of 2015. Review pursuant to Security Council resolution 2253 (2015) was concluded on 21 Feb. 2019. Review pursuant to Security Council resolution 2610 (2021) was concluded on 8 November 2022. INTERPOL-UN Security Council Special Notice web link: https://www.interpol.int/en/How-we-work/Notices/View-UN-Notices-Individuals click here

QDi.377 Name: 1: MUHANNAD 2: AL-NAJDI 3: na 4: na
Title: na **Designation:** na **DOB:** 19 May 1984 **POB:** al-Duwadmi, Saudi Arabia **Good quality a.k.a.:** 'Ali Manahi 'Ali al-Mahaydali al-'Utaybi **Low quality a.k.a.:** Ghassan al-Tajiki **Nationality:** Saudi Arabia **Passport no:** na **National identification no:** na **Address:** na **Listed on:** 29 Feb. 2016 (amended on 24 Nov. 2020) **Other information:** Syria-based Al-Qaida (QDe.004) facilitator. Involved in the development of improvised explosive devices for use in Afghanistan and Syrian Arab Republic since at least 2010. Review pursuant to Security Council resolution 2368 (2017) was concluded on 24 November 2020. INTERPOL-UN Security Council Special Notice web link: https://www.interpol.int/en/How-we-work/Notices/View-UN-Notices-Individuals click here

QDi.334 Name: 1: ‘ABD AL-RAHMAN 2: BIN ‘UMAYR 3: AL-NU’AYMI 4: na
Title: na **Designation:** na **DOB:** 1954 **POB:** Doha, Qatar **Good quality a.k.a.: a)** Abd al-Rahman bin 'Amir al-Na'imi **b)** 'Abd al-Rahman al-Nu'aimi **c)** 'Abd al-Rahman bin 'Amir al-Nu'imi **d)** 'Abd al-Rahman bin 'Amir al-Nu'aymi **e)** 'Abdallah Muhammad al-Nu'aymi **f)** 'Abd al-Rahman al-Nua'ymi **g)** A. Rahman al-Naimi **h)** Abdelrahman Imer al Jaber al Naimeh **i)** A. Rahman Omair J Alnaimi **j)** Abdulrahman Omair al Neaimi **Low quality a.k.a.:** na **Nationality:** Qatar **Passport no: a)** Qatar number 01461558 (expiring 20 Jan. 2024) **b)** Qatari passport number 00868774 (expired on 27 Apr. 2014) **National identification no: a)** Qatar 25463400086 **b)** Qatar 25463401784 (expires on 6 Dec. 2019) **Address:** Al-Waab, Qatar **Listed on:** 23 Sep. 2014 (amended on 15 Feb. 2017, 23 Mar. 2021) **Other information:** Financier and facilitator for Al-Qaida (QDe.004) and Al-Qaida in Iraq (QDe.115). INTERPOL-UN Security Council Special Notice web link: https://www.interpol.int/en/How-we-work/Notices/View-UN-Notices-Individuals click here

QDi.273 Name: 1: FAZEEL-A-TUL 2: SHAYKH ABU MOHAMMED 3: AMEEN 4: AL-PESHAWARI
Title: na **Designation:** na **DOB: a)** Approximately 1967 **b)** Approximately 1961 **c)** Approximately 1973 **POB:** Shunkrai village, Sarkani District, Konar Province, Afghanistan **Good quality a.k.a.: a)** Shaykh Aminullah **b)** Sheik Aminullah **c)** Abu Mohammad Aminullah Peshawari **d)** Abu Mohammad Amin Bishawri **e)** Abu Mohammad Shaykh Aminullah Al-Bishauri **f)** Shaykh Abu Mohammed Ameen al-Peshawari **g)** Shaykh Aminullah Al-Peshawari **Low quality a.k.a.:** na **Nationality:** Afghan **Passport no:** na **National identification no:** na **Address:** Ganj District, Peshawar, Pakistan **Listed on:** 29 Jun. 2009 (amended on 24 Jul. 2013, 1 May 2019) **Other information:** Associated with Al-Qaida (QDe.004). Head of Ganj madrasa, a.k.a. Madrasa Jamia Taleemul Quran wal Hadith, a.k.a. Madrasa Taleemul Quran wal Sunnah, located at the Ganj Gate, Phandu Road, Peshawar, Pakistan. Review pursuant to Security Council resolution 2253 (2015) was concluded on 21 Feb. 2019. Review pursuant to Security Council resolution 2610 (2021) was concluded on 8 November 2022. INTERPOL-UN Security Council Special Notice web link: https://www.interpol.int/en/How-we-work/Notices/View-UN-Notices-Individuals click here

QDi.339 Name: 1: ‘ABD AL-RAHMAN 2: MUHAMMAD 3: MUSTAFA 4: AL-QADULI
Title: na **Designation:** na **DOB: a)** 1959 **b)** 1957 **POB:** Mosul, Ninawa Province, Iraq **Good quality a.k.a.: a)** ‘Abd al-Rahman Muhammad Mustafa Shaykhlari **b)** Umar Muhammad Khalil Mustafa **c)** Abdul Rahman Muhammad al-Bayati **d)** Tahir Muhammad Khalil Mustafa al-Bayati **e)** Aliazra Ra’ad Ahmad **Low quality a.k.a.: a)** Abu-Shuayb **b)** Hajji Iman **c)** Abu Iman **d)** Abu Ala **e)** Abu Hasan **f)** Abu Muhammad **g)** Abu Zayna **Nationality:** Iraq **Passport no:** na **National identification no:** na **Address:** na **Listed on:** 23 Sep. 2014 (amended on 6 Dec. 2019) **Other information:** Senior Islamic State in Iraq and the Levant (ISIL), listed as Al-Qaida in Iraq (AQI) (QDe.115), official. Previously served as a representative of AQI to Al-Qaida (QDe.004) senior leadership in Pakistan. Review pursuant to Security Council resolution 2368 (2017) was concluded on 4 Dec. 2019

INTERPOL-UN Security Council Special Notice web link: https://www.interpol.int/en/How-we-work/Notices/View-UN-Notices-Individuals click here

QDi.432 Name: 1: ASHRAF 2: AL-QIZANI 3: na 4: na
Name (original script): أشرف القيزاني
Title: na **Designation:** na **DOB:** 5 Oct. 1991 **POB:** Gouazine, Dahmani, Governorate of Le Kef, Tunisia **Good quality a.k.a.: a)** Ashraf al-Gizani **b)** Abu ‘Ubaydah al-Kafi **c)** Achref Ben Fethi Ben Mabrouk Guizani **d)** Achraf Ben Fathi Ben Mabrouk Guizani **Low quality a.k.a.:** na **Nationality:** Tunisia **Passport no:** na **National identification no:** Tunisia 13601334 **Address:** na **Listed on:** 29 Dec. 2021 **Other information:** Senior member of Islamic State in Iraq and the Levant (ISIL), listed as Al-Qaida in Iraq (QDe.115). Recruited for ISIL and instructed individuals to perpetrate terrorist acts via online video. INTERPOL-UN Security Council Special Notice web link: https://www.interpol.int/en/How-we-work/Notices/View-UN-Notices-Individuals click here

QDi.282 Name: 1: QASIM 2: MOHAMED 3: MAHDI 4: AL-RIMI
Name (original script): قاسم محمد مهدي الريمي
Title: na **Designation:** na **DOB:** 5 Jun. 1978 **POB:** Raymah village, Sanaa Governorate (ريمة), Yemen **Good quality a.k.a.: a)** Qasim Al-Rimi **b)** Qasim al-Raymi **c)** Qassim al-Raymi **d)** Qasim al-Rami **e)** Qasim Mohammed Mahdi Al Remi **f)** Qassim Mohammad Mahdi Al Rimi **Low quality a.k.a.: a)** Qasim Yahya Mahdi 'Abd al-Rimi **b)** Abu Hurayah al-Sana'ai **c)** Abu 'Ammar **d)** Abu Hurayrah **Nationality:** Yemen **Passport no:** Yemeni number 00344994, issued on 3 Jul. 1999, issued in Sanaa **National identification no:** Yemeni national identification number 973406, issued on 3 Jul. 1996 **Address:** Yemen **Listed on:** 11 May 2010 (amended on 15 Apr. 2014, 24 Jun. 2016, 24 Nov. 2020) **Other information:** Mother’s name: Fatima Muthanna Yahya. Photo available for inclusion in the INTERPOL-UN Security Council Special Notice. Leader of Al-Qaida in the Arabian Peninsula (QDe.129) since Jun. 2015, pledged loyalty to Aiman al-Zawahiri (QDi.006). Review pursuant to Security Council resolution 2368 (2017) was concluded on 24 November 2020. INTERPOL-UN Security Council Special Notice web link: https://www.interpol.int/en/How-we-work/Notices/View-UN-Notices-Individuals click here

QDi.369 Name: 1: MU’TASSIM 2: YAHYA 3: ‘ALI 4: AL-RUMAYSH
Title: na **Designation:** na **DOB:** 4 Jan. 1973 **POB:** Jeddah, Saudi Arabia **Good quality a.k.a.:** na **Low quality a.k.a.: a)** Rayhanah **b)** Abu-Rayhanah **c)** Handalah **d)** Abu-Rayhanah al-'Ansari al-Jeddawi **Nationality:** Yemen **Passport no:** Yemen number 01055336 **National identification no:** Saudi Arabia alien registration number 2054275397, issued on 22 Jul. 1998 **Address:** na **Listed on:** 29 Sep. 2015 (amended on 6 Dec. 2019) **Other information:** Financial and foreign fighter facilitator for Islamic State in Iraq and the Levant, listed as Al-Qaida in Iraq (QDe.115). Member of Al-Qaida in the Arabian Peninsula (AQAP) (QDe.129) since at least Jun. 2014. Review pursuant to Security Council resolution 2368 (2017) was concluded on 4 Dec. 2019 INTERPOL-UN Security Council Special Notice web link: https://www.interpol.int/en/How-we-work/Notices/View-UN-Notices-Individuals click here

QDi.415 Name: 1: ADNAN 2: ABOU WALID 3: AL-SAHRAOUI 4: na
Name (original script): عدنان أبو وليد الصحراوي
Title: na **Designation:** na **DOB:** 16 Feb. 1973 **POB:** Laayoune **Good quality a.k.a.: a)** Lahbib Idrissi ould Sidi Abdi ould Said ould El Bachir **b)** Adnan Abu Walid al-Sahrawi **c)** Abu Walid al Sahrawi **d)** Adnan Abu Walid al-Sahraoui **e)** Adnan Abu Waleed al-Sahrawi **f)** Lehbib Ould Ali Ould Said Ould Joumani **Low quality a.k.a.:** na **Nationality:** na **Passport no:** na **National identification no:** na **Address:** Ménaka, Gao Region, Mali **Listed on:** 9 Aug. 2018 (amended on 2 Feb. 2023) **Other information:** Reportedly Deceased. Former spokesperson of the Mouvement pour l’Unification et le Jihad en Afrique de l’Ouest (MUJAO) (QDe.134). Emir of the Al-Mourabitoun (QDe.141) group in Mali. Pledged allegiance to Islamic State in Iraq and the Levant (ISIL), listed as Al-Qaida in Iraq (QDe.115) in May 2015. INTERPOL-UN Security Council Special Notice web link: https://www.interpol.int/en/How-we-work/Notices/View-UN-Notices-Individuals click here

QDi.381 Name: 1: HAMAD 2: AWAD 3: DAHI SARHAN 4: AL-SHAMMARI
Name (original script): حمد عوض ضاحي سرحان الشمري
Title: na **Designation:** na **DOB:** 31 Jan. 1984 **POB:** na **Good quality a.k.a.:** na **Low quality a.k.a.:** Abu Uqlah al-Kuwaiti **Nationality:** Kuwait **Passport no:** Kuwait number 155454275 **National identification no:** Kuwait identity card 284013101406 **Address:** na **Listed on:** 21 Sep. 2015 (amended on 1 May 2019) **Other information:** Kuwait-based facilitator who provides financial services to, or in support of, Al-Qaida (QDe.004) and Al-Nusrah Front for the People of the Levant (QDe.137). Review pursuant to Security Council resolution 2253 (2015) was concluded on 21 Feb. 2019. INTERPOL-UN Security Council Special Notice web link: https://www.interpol.int/en/How-we-work/Notices/View-UN-Notices-Individuals click here

QDi.384 Name: 1: ALI MUSA 2: AL-SHAWAKH 3: na 4: na
Title: na **Designation:** na **DOB:** 1973 **POB:** Sahl Village, Raqqa Province, Syrian Arab Republic **Good quality a.k.a.: a)** 'Ali Musa al-Shawagh **b)** Ali al-Hamoud al-Shawakh **c)** Ibrahim al-Shawwakh **d)** Muhammad 'Ali al-Shawakh **Low quality a.k.a.: a)** Abu Luqman **b)** Ali Hammud **c)** Abdullah Shuwar al-Aujayd **d)** Ali Awas **e)** 'Ali Derwish **f)** 'Ali al-Hamud **g)** Abu Luqman al-Sahl **h)** Abu Luqman al-Suri **i)** Abu Ayyub **Nationality:** Syrian Arab Republic **Passport no:** na **National identification no:** na **Address:** Syrian Arab Republic **Listed on:** 29 Feb. 2016 (amended on 6 Dec. 2019, 18 Mar. 2020) **Other information:** A leader of Islamic State in Iraq and the Levant (ISIL), listed as Al-Qaida in Iraq (QDe.115). As of Jun, 2015, al-Shawakh was the ISIL governor of Aleppo. Review pursuant to Security Council resolution 2368 (2017) was concluded on 4 Dec. 2019 INTERPOL-UN Security Council Special Notice web link: https://www.interpol.int/en/How-we-work/Notices/View-UN-Notices-Individuals click here

QDi.385 Name: 1: HASAN 2: AL-SALAHAYN 3: SALIH 4: AL-SHA' ARI
Title: na **Designation:** na **DOB:** 1975 **POB:** Derna, Libya **Good quality a.k.a.:** Husayn al-Salihin Salih al-Sha 'iri **Low quality a.k.a.: a)** Abu Habib al-Libi **b)** Hasan Abu Habib **Nationality:** Libya **Passport no:** Libya number 542858 **National identification no:** Libya national identification number 55252, issued in Derna, Libya **Address:** Libya **Listed on:** 29 Feb. 2016 **Other information:** Facilitator for Islamic State in Iraq and the Levant (ISIL), listed as Al-Qaida in Iraq (QDe.115). Review pursuant to Security Council resolution 2368 (2017) was concluded on 15 November 2021. INTERPOL-UN Security Council Special Notice web link: https://www.interpol.int/en/How-we-work/Notices/View-UN-Notices-Individuals click here

QDi.015 Name: 1: MAHFOUZ 2: OULD 3: AL-WALID 4: na
Name (original script): محفوظ ولد الوليد
Title: na **Designation:** na **DOB:** 1 Jan. 1975 **POB:** Mauritania **Good quality a.k.a.: a)** Abu Hafs the Mauritanian **b)** Khalid Al-Shanqiti **c)** Mafouz Walad Al-Walid **Low quality a.k.a.:** na **Nationality:** Mauritania **Passport no:** na **National identification no:** na **Address:** na **Listed on:** 6 Oct. 2001 (amended on 1 Jun. 2007, 10 Jun. 2011, 24 Nov. 2020) **Other information:** Review pursuant to Security Council resolution 1822 (2008) was concluded on 15 Jun. 2010. Review pursuant to Security Council resolution 2368 (2017) was concluded on 24 November 2020. INTERPOL-UN Security Council Special Notice web link: https://www.interpol.int/en/How-we-work/Notices/View-UN-Notices-Individuals click here

QDi.329 Name: 1: AHMED 2: ABDULLAH 3: SALEH AL-KHAZMARI 4: AL-ZAHRANI
Title: na **Designation:** na **DOB:** 15 Sep. 1978 **POB:** Dammam, Saudi Arabia **Good quality a.k.a.: a)** Abu Maryam al-Zahrani **b)** Abu Maryam al-Saudi **c)** Ahmed Abdullah S al-Zahrani **d)** Ahmad Abdullah Salih al-Zahrani **e)** Abu Maryam al-Azadi **f)** Ahmed bin Abdullah Saleh bin al-Zahrani **g)** Ahmed Abdullah Saleh al-Zahrani al-Khozmri **Low quality a.k.a.:** na **Nationality:** Saudi Arabia **Passport no:** Saudi Arabia number E126785, issued on 27 May 2002 (expired on 3 Apr. 2007) **National identification no:** na **Address:** (Located in Syria) **Listed on:** 23 Sep. 2014 (amended on 6 Dec. 2019) **Other information:** Senior member of Al-Qaida (QDe.004). Wanted by the Saudi

Arabian Government for terrorism. Father's name is Abdullah Saleh al Zahrani. Physical description: eye colour: dark; hair colour: dark; complexion: olive. Speaks Arabic. Photo available for inclusion in the INTERPOL-UN Security Council Special Notice. Review pursuant to Security Council resolution 2368 (2017) was concluded on 4 Dec. 2019. INTERPOL-UN Security Council Special Notice web link: https://www.interpol.int/en/How-we-work/Notices/View-UN-Notices-Individuals click here

QDi.392 Name: 1: FAYSAL 2: AHMAD 3: BIN ALI 4: AL-ZAHRANI
Name (original script): فيصل احمد بن علي الزهراني
Title: na **Designation:** na **DOB:** 19 Jan. 1986 **POB:** na **Good quality a.k.a.:** Faisal Ahmed Ali Alzahrani **Low quality a.k.a.: a)** Abu Sarah al-Saudi **b)** Abu Sara Zahrani **Nationality:** Saudi Arabia **Passport no: a)** Saudi Arabia number K142736, issued on 14 Jul. 2011, issued in Al-Khafji, Saudi Arabia **b)** Saudi Arabia number G579315 **National identification no:** na **Address:** Syrian Arab Republic **Listed on:** 20 Apr. 2016 (amended on 1 May 2019) **Other information:** Was the lead oil and gas division official of Islamic State in Iraq and the Levant (ISIL), listed as Al-Qaida in Iraq (QDe.115), for Al Barakah Governorate, Syrian Arab Republic, as of May 2015. Review pursuant to Security Council resolution 2253 (2015) was concluded on 21 Feb. 2019. INTERPOL-UN Security Council Special Notice web link: https://www.interpol.int/en/How-we-work/Notices/View-UN-Notices-Individuals click here

QDi.401 Name: 1: GHALIB 2: ABDULLAH 3: AL-ZAIDI 4: na
Name (original script): غالب عبدالله الزيدي
Title: na **Designation:** na **DOB: a)** 1975 **b)** 1970 **POB:** Raqqah Region, Marib Governorate, Yemen **Good quality a.k.a.: a)** Ghalib Abdallah al-Zaydi **b)** Ghalib Abdallah Ali al-Zaydi **Low quality a.k.a.:** Ghalib al Zaydi **Nationality:** Yemen **Passport no:** na **National identification no:** na **Address:** na **Listed on:** 22 Feb. 2017 (amended on 6 Dec. 2019) **Other information:** A leader of Al-Qaida in the Arabian Peninsula (AQAP) (QDe.129) in Marib Governorate, Yemen since 2015. Provided AQAP with weapons, funding and recruits. Review pursuant to Security Council resolution 2368 (2017) was concluded on 4 Dec. 2019 INTERPOL-UN Security Council Special Notice web link: https://www.interpol.int/en/How-we-work/Notices/View-UN-Notices-Individuals click here

QDi.006 Name: 1: AIMAN 2: MUHAMMED 3: RABI 4: AL-ZAWAHIRI
Name (original script): أيمن محمد ربيع الظواهري
Title: a) Doctor **b)** Dr. **Designation:** na **DOB:** 19 Jun. 1951 **POB:** Giza, Egypt **Good quality a.k.a.: a)** Ayman Al-Zawahari **b)** Ahmed Fuad Salim **c)** Al Zawahry Aiman Mohamed Rabi Abdel Muaz **d)** Al Zawahiri Ayman **e)** Abdul Qader Abdul Aziz Abdul Moez Al Doctor **f)** Al Zawahry Aiman Mohamed Rabi **g)** Al Zawahry Aiman Mohamed Rabie **h)** Al Zawahry Aiman Mohamed Robi **i)** Dhawahri Ayman **j)** Eddaouahiri Ayman **k)** Nur Al Deen Abu Mohammed **l)** Ayman Al Zawahari **m)** Ahmad Fuad Salim **Low quality a.k.a.: a)** Abu Fatma **b)** Abu Mohammed **Nationality:** Egypt **Passport no: a)** Egypt number 1084010 **b)** 19820215 **National identification no:** na **Address:** na **Listed on:** 25 Jan. 2001 (amended on 2 Jul. 2007, 18 Jul. 2007, 13 Aug. 2007, 16 Dec. 2010, 22 May 2015, 24 Nov. 2020) **Other information:** Leader of Al-Qaida (QDe.004). Former operational and military leader of Egyptian Islamic Jihad (QDe.003), was a close associate of Usama Bin Laden (deceased). Believed to be in the Afghanistan/Pakistan border area. Review pursuant to Security Council resolution 1822 (2008) was concluded on 21 Jun. 2010. Review pursuant to Security Council resolution 2368 (2017) was concluded on 24 November 2020. INTERPOL-UN Security Council Special Notice web link: https://www.interpol.int/en/How-we-work/Notices/View-UN-Notices-Individuals click here

QDi.156 Name: 1: ABD-AL-MAJID 2: AZIZ 3: AL-ZINDANI 4: na
Name (original script): عبد المجيد عزيز الزنداني
Title: Sheikh **Designation:** na **DOB:** 1950 **POB:** Yemen **Good quality a.k.a.: a)** Abdelmajid Al-Zindani **b)** Shaykh 'Abd Al-Majid Al-Zindani **c)** Sheikh Abd Al-Meguid Al-Zandani **Low quality a.k.a.:** na **Nationality:** Yemen **Passport no:** Yemen number A005487, issued on 13 Aug. 1995 **National identification no:** na **Address:** P.O. Box 8096, Sana'a, Yemen **Listed on:** 27 Feb. 2004 (amended on 25 Jul. 2006, 10 Jun. 2011, 1 May 2019) **Other information:** Review pursuant

to Security Council resolution 1822 (2008) was concluded on 2 Jun. 2010. Review pursuant to Security Council resolution 2253 (2015) was concluded on 21 Feb. 2019. Review pursuant to Security Council resolution 2610 (2021) was concluded on 8 November 2022. INTERPOL-UN Security Council Special Notice web link: https://www.interpol.int/en/How-we-work/Notices/View-UN-Notices-Individuals click here

QDi.326 Name: 1: HAMID 2: HAMAD 3: HAMID 4: AL- ‘ALI
Title: na **Designation:** na **DOB:** 17 Nov. 1960 **POB:** Kuwait **Good quality a.k.a.:** na **Low quality a.k.a.:** na **Nationality:** Kuwait **Passport no: a)** Kuwait number 001714467 **b)** Kuwait number 101505554 **National identification no:** na **Address:** na **Listed on:** 15 Aug. 2014 (amended on 23 Mar. 2021) **Other information:** A Kuwait-based financier, recruiter and facilitator for Islamic State in Iraq and the Levant, listed as Al-Qaida in Iraq (QDe.115), and Jabhat al-Nusrah, listed as Al-Nusrah Front for the People of the Levant (QDe.137). Associated with Ibrahim Awwad Ibrahim Ali al-Badri al-Samarrai (QDi.299) and Abu Mohammed al-Jawlani (QDi.317). Review pursuant to Security Council resolution 2368 (2017) was concluded on 15 November 2021. INTERPOL-UN Security Council Special Notice web link: https://www.interpol.int/en/How-we-work/Notices/View-UN-Notices-Individuals click here

QDi.335 Name: 1: ‘ABD AL-RAHMAN 2: KHALAF 3: ‘UBAYD JUDAY’ 4: AL- ‘ANIZI
Title: na **Designation:** na **DOB:** 6 Mar. 1973 **POB:** na **Good quality a.k.a.: a)** ‘Abd al-Rahman Khalaf al-Anizi **b)** ‘Abd al-Rahman Khalaf al- ‘Anzi **Low quality a.k.a.: a)** Abu Usamah al-Rahman **b)** Abu Shaima’ Kuwaiti **c)** Abu Usamah al-Kuwaiti **d)** Abu Usama **e)** Yusuf **Nationality:** Kuwait **Passport no:** na **National identification no:** Kuwait 273030601222 **Address:** Syrian Arab Republic (located in since 2013) **Listed on:** 23 Sep. 2014 (amended on 15 Feb. 2017, 1 May 2019, 2 Feb. 2023) **Other information:** A sentence of imprisonment for 15 years was issued against him by Kuwait in absentia on 30 July 2015. Provides support to Al-Qaida (QDe.004) and Islamic State in Iraq and the Levant, listed as Al-Qaida in Iraq (AQI) (QDe.115), in Syria and Iraq. Review pursuant to Security Council resolution 2253 (2015) was concluded on 21 Feb. 2019. INTERPOL-UN Security Council Special Notice web link: https://www.interpol.int/en/How-we-work/Notices/View-UN-Notices-Individuals click here

QDi.154 Name: 1: SULAIMAN 2: JASSEM 3: SULAIMAN 4: ALI ABO GHAITH
Name (original script): سليمان جاسم سليمان علي أبوغيث
Title: na **Designation:** na **DOB:** 14 Dec. 1965 **POB:** Kuwait **Good quality a.k.a.:** na **Low quality a.k.a.:** Abo Ghaith **Nationality:** Kuwaiti citizenship withdrawn in 2002 **Passport no:** Kuwaiti number 849594, issued on 27 Nov. 1998, issued in Kuwait (and expired on 24 Jun. 2003) **National identification no:** na **Address:** na **Listed on:** 16 Jan. 2004 (amended on 23 Jul. 2008, 10 Jun. 2011, 24 Nov. 2020) **Other information:** Left Kuwait for Pakistan in June 2001. Review pursuant to Security Council resolution 1822 (2008) was concluded on 21 Jun. 2010. Review pursuant to Security Council resolution 2368 (2017) was concluded on 24 November 2020. INTERPOL-UN Security Council Special Notice web link: https://www.interpol.int/en/How-we-work/Notices/View-UN-Notices-Individuals click here

QDi.299 Name: 1: IBRAHIM 2: AWWAD 3: IBRAHIM 4: ALI AL-BADRI AL-SAMARRAI
Name (original script): إبراهيم عواد إبراهيم علي البدري السامرائي
Title: Dr. **Designation:** na **DOB:** 1971 **POB:** Iraq **Good quality a.k.a.:** Dr. Ibrahim ‘Awwad Ibrahim ’ Ali al-Badri al-Samarrai’ (born in 1971 in Samarra, Iraq (Ibrahim ‘Awad Ibrahim al-Badri al-Samarrai; Ibrahim ‘Awad Ibrahim al-Samarra’ i; Dr. Ibrahim Awwad Ibrahim al-Samarra’ i)) **Low quality a.k.a.: a)** أبو دعاء ((Abu Du’ a; Abu Duaa’) prominently known by this nom de guerre) **b)** Dr. Ibrahim **c)** أبو بكر البغدادي الحسيني القريشي (Abu Bakr al-Baghdadi al-Husayni al-Quraishi; Abu Bakr al-Baghdadi) **Nationality:** Iraq **Passport no:** na **National identification no:** Ration card number: 0134852 **Address: a)** Iraq **b)** Syrian Arab Republic **Listed on:** 5 Oct. 2011 (amended on 20 Jul. 2012, 10 Dec. 2015, 24 Nov. 2020) **Other information:** Description: Height: 1.65 m. Weight: 85 kg. Black hair and eyes. White skin. Leader of Islamic State in Iraq and the Levant, listed as Al-Qaida in

Iraq (AQI) (QDe.115). Currently based in Iraq and Syria. Declared himself "caliph" in Mosul in 2014. Responsible for managing and directing AQI large scale operations. Wife's name: Saja Hamid al-Dulaimi. Wife's name: Asma Fawzi Mohammed al-Kubaissi. Wanted by the Iraqi security forces. Photo available for inclusion in the INTERPOL-UN Security Council Special Notice. Review pursuant to Security Council resolution 2368 (2017) was concluded on 24 November 2020. Review pursuant to Security Council resolution 2368 (2017) was concluded on 15 November 2021. INTERPOL-UN Security Council Special Notice web link: https://www.interpol.int/en/How-we-work/Notices/View-UN-Notices-Individuals click here

QDi.296 Name: 1: MATI UR-REHMAN 2: ALI MUHAMMAD 3: na 4: na
Name (original script): مطيع الرحمن علی محمد
Title: na **Designation:** na **DOB:** Approximately 1977 **POB:** Chak number 36/DNB, Rajkan, Madina Colony, Bahawalpur District, Punjab Province, Pakistan **Good quality a.k.a.: a)** Mati-ur Rehman **b)** Mati ur Rehman **c)** Matiur Rahman **d)** Matiur Rehman **e)** Matti al-Rehman **f)** Abdul Samad **g)** Samad Sial **h)** Abdul Samad Sial **i)** Ustad Talha **j)** Qari Mushtaq **Low quality a.k.a.: a)** Tariq **b)** Hussain **Nationality:** Pakistan **Passport no:** na **National identification no:** na **Address:** na **Listed on:** 22 Aug. 2011 (amended on 10 May 2012, 17 Oct. 2013, 24 Nov. 2020, 2 Feb. 2023) **Other information:** Physical description: 5 feet 2 inches; 157.4 cm. Name of father: Ali Muhammad. Mati ur-Rehman is the chief operational commander of Lashkar i Jhangvi (LJ) (QDe.096). Associated with Harakat-ul Jihad Islami (QDe.130). Reportedly deceased. Review pursuant to Security Council resolution 2368 (2017) was concluded on 24 November 2020. INTERPOL-UN Security Council Special Notice web link: https://www.interpol.int/en/How-we-work/Notices/View-UN-Notices-Individuals click here

QDi.370 Name: 1: TARAD 2: MOHAMMAD 3: Alnori Alfares 4: ALJARBA
Title: na **Designation:** na **DOB:** 20 Nov. 1979 **POB:** Iraq **Good quality a.k.a.:** Tarad Aljarba **Low quality a.k.a.:** Abu-Muhammad al-Shimali **Nationality:** Saudi Arabia **Passport no:** E704088, issued on 26 Aug. 2003 (expired on 2 Jul. 2008) **National identification no:** 1121628414 **Address:** na **Listed on:** 29 Sep. 2015 (amended on 6 Dec. 2019, 10 Sep. 2020) **Other information:** Border emir of Islamic State in Iraq and the Levant (ISIL), listed as Al-Qaida in Iraq (QDe.115) as of Apr. 2015, and ISIL's leader for operations outside of the Syrian Arab Republic and Iraq as of mid-2014. Facilitated the travel from Turkey to the Syrian Arab Republic of prospective ISIL fighters from Australia, Europe, and the Middle East. Managed ISIL's guesthouse in Azaz, Syrian Arabic Republic as of 2014. Review pursuant to Security Council resolution 2368 (2017) was concluded on 4 Dec. 2019 INTERPOL-UN Security Council Special Notice web link: https://www.interpol.int/en/How-we-work/Notices/View-UN-Notices-Individuals click here

QDi.076 Name: 1: ISAM 2: ALI 3: MOHAMED 4: ALOUCHE
Name (original script): عصام علي محمد علوش
Title: na **Designation:** na **DOB: a)** 1972 **b)** 21 Mar. 1974 **POB:** Baghdad, Iraq **Good quality a.k.a.:** Mansour Thaer born 21 Mar. 1974 in Baghdad, Iraq **Low quality a.k.a.:** na **Nationality:** Jordan **Passport no:** na **National identification no:** na **Address:** na **Listed on:** 3 Sep. 2002 (amended on 18 Aug. 2006, 30 Jan. 2009, 6 Dec. 2019) **Other information:** Was deported from Germany to Jordan in Feb. 2005. Review pursuant to Security Council resolution 1822 (2008) was concluded on 21 Jun. 2010. Review pursuant to Security Council resolution 2368 (2017) was concluded on 4 Dec. 2019. INTERPOL-UN Security Council Special Notice web link: https://www.interpol.int/en/How-we-work/Notices/View-UN-Notices-Individuals click here

QDi.422 Name: 1: MOHAMMED 2: MASOOD 3: AZHAR 4: ALVI
Name (original script): محمد مسعود اظہر علوی
Title: na **Designation:** na **DOB: a)** 10 Jul. 1968 **b)** 10 Jun. 1968 **POB:** Bahawalpur, Punjab Province, Pakistan **Good quality a.k.a.:** na **Low quality a.k.a.: a)** Masud Azhar **b)** Wali Adam Isah **c)** Wali Adam Esah **Nationality:** Pakistan **Passport no:** na **National identification no:** na **Address:** na **Listed on:** 1 May 2019 **Other information:** Founder of Jaish-i-Mohammed (QDe.019). Former

leader of Harakat ul-Mujahidin / HUM (QDe.008). INTERPOL-UN Security Council Special Notice web link: https://www.interpol.int/en/How-we-work/Notices/View-UN-Notices-Individuals click here

QDi.152 Name: 1: SAIFI 2: AMMARI 3: na 4: na
Name (original script): سيفي عماري
Title: na **Designation:** na **DOB:** **a)** 1 Jan. 1968 **b)** 24 Apr. 1968 **POB:** **a)** Kef Rih, Algeria **b)** Guelma, Algeria **Good quality a.k.a.:** **a)** El Para (combat name) **b)** Abderrezak Le Para (combat name) **c)** Abou Haidara **d)** El Ourassi **e)** Abderrezak Zaimeche **f)** Abdul Rasak ammane Abu Haidra **g)** Abdalarak **Low quality a.k.a.:** na **Nationality:** Algeria **Passport no:** na **National identification no:** na **Address:** Algeria **Listed on:** 4 Dec. 2003 (amended on 7 Apr. 2008, 16 May 2011, 6 Dec. 2019) **Other information:** In detention in Algeria since Oct. 2004. Former member of the GSPC listed as The Organization of Al-Qaida in the Islamic Maghreb (QDe.014). Review pursuant to Security Council resolution 1822 (2008) was concluded on 27 Jul. 2010. Review pursuant to Security Council resolution 2368 (2017) was concluded on 4 Dec. 2019. INTERPOL-UN Security Council Special Notice web link: https://www.interpol.int/en/How-we-work/Notices/View-UN-Notices-Individuals click here

QDi.404 Name: 1: MUHAMMAD 2: BAHRUM 3: NAIM 4: ANGGIH TAMTOMO
Title: na **Designation:** na **DOB:** 6 Sep. 1983 **POB:** Surakarta, Indonesia **Good quality a.k.a.:** **a)** Bahrun Naim **b)** Anggih Tamtomo **Low quality a.k.a.:** **a)** Abu Rayyan **b)** Abu Rayan **c)** Abu Aisyah **Nationality:** Indonesia **Passport no:** na **National identification no:** na **Address:** **a)** Aleppo, Syrian Arab Republic **b)** Raqqa, Syrian Arab Republic **Listed on:** 20 Jul. 2017 (amended on 2 Feb. 2023) **Other information:** Syrian-based Indonesian national who has served in a variety of roles supporting the Islamic State in Iraq and the Levant, listed as Al-Qaida in Iraq (QDe.115). INTERPOL-UN Security Council Special Notice web link: https://www.interpol.int/en/How-we-work/Notices/View-UN-Notices-Individuals click here

QDi.216 Name: 1: ABDULLAH 2: ANSHORI 3: na 4: na
Title: na **Designation:** na **DOB:** 1958 **POB:** Pacitan, East Java, Indonesia **Good quality a.k.a.:** **a)** Abu Fatih **b)** Thoyib, Ibnu **c)** Toyib, Ibnu **d)** Abu Fathi **Low quality a.k.a.:** na **Nationality:** Indonesia **Passport no:** Indonesia number T710219 (issued in Sukoharjo, Central Java, Indonesia) **National identification no:** na **Address:** na **Listed on:** 21 Apr. 2006 (amended on 6 Dec. 2019, 2 Feb. 2023) **Other information:** Senior leader of Jemaah Islamiyah (QDe.092). Review pursuant to Security Council resolution 1822 (2008) was concluded on 8 Jun. 2010. Review pursuant to Security Council resolution 2368 (2017) was concluded on 4 Dec. 2019. INTERPOL-UN Security Council Special Notice web link: https://www.interpol.int/en/How-we-work/Notices/View-UN-Notices-Individuals click here

QDi.323 Name: 1: SAID 2: ARIF 3: na 4: na
Title: na **Designation:** na **DOB:** **a)** 25 Jun. 1964 **b)** 5 Dec. 1965 **POB:** Oran, Algeria **Good quality a.k.a.:** **a)** Said Mohamed Arif **b)** Omar Gharib **c)** Abderahmane **d)** Abdallah al-Jazairi **e)** Slimane Chabani **f)** Souleiman **Low quality a.k.a.:** na **Nationality:** Algeria **Passport no:** na **National identification no:** na **Address:** na **Listed on:** 15 Aug. 2014 (amended on 6 Dec. 2019) **Other information:** A veteran member of the ‘Chechen Network’ (not listed) and other terrorist groups. He was convicted of his role and membership in the ‘Chechen Network’ in France in 2006. Joined Jabhat al-Nusrah, listed as Al-Nusrah Front for the People of the Levant (QDe.137) in October 2013. Review pursuant to Security Council resolution 2368 (2017) was concluded on 4 Dec. 2019 INTERPOL-UN Security Council Special Notice web link: https://www.interpol.int/en/How-we-work/Notices/View-UN-Notices-Individuals click here

QDi.398 Name: 1: RUSTAM 2: MAGOMEDOVICH 3: ASELDEROV 4: na
Name (original script): Рустам Магомедович Асельдеров
Title: na **Designation:** na **DOB:** 9 Mar. 1981 **POB:** Iki-Burul Village, Iki-Burulskiy District, Republic of Kalmykia, Russian Federation **Good quality a.k.a.:** na **Low quality a.k.a.:** **a)** Abu Muhammad

(original script: Абу Мухаммад) **b)** Abu Muhammad Al-Kadari (original script: Абу Мухаммад Аль-Кадари) **c)** Muhamadmuhtar (original script: Мухамадмухтар) **Nationality:** Russian Federation **Passport no:** Russian passport number 8208 No. 555627 (issued by Leninskiy Office, Directorate of the Federal Migration Service of the Russian Federation for the Republic of Dagestan) **National identification no:** na **Address:** na **Listed on:** 12 Dec. 2016 (amended on 9 Aug. 2017, 1 May 2019) **Other information:** Led a group of over 160 terrorist fighters, which operates in the Republics of Dagestan, Chechnya and Ingushetia, Russian Federation. Killed on 3 December 2016 in Makhachkala, the Republic of Dagestan, Russian Federation. Photo available for inclusion in the INTERPOL-UN Security Council Special Notice. Review pursuant to Security Council resolution 2253 (2015) was concluded on 21 Feb. 2019. Review pursuant to Security Council resolution 2368 (2017) was concluded on 15 November 2021. INTERPOL-UN Security Council Special Notice web link: https://www.interpol.int/en/How-we-work/Notices/View-UN-Notices-Individuals click here

QDi.184 Name: 1: MUHSIN 2: FADHIL 3: AYED 4: ASHOUR AL-FADHLI
Name (original script): محسن فاضل عايد عاشور الفضلي
Title: na **Designation:** na **DOB:** 24 Apr. 1981 **POB:** Kuwait **Good quality a.k.a.: a)** Muhsin Fadhil 'Ayyid al Fadhli **b)** Muhsin Fadil Ayid Ashur al Fadhli **c)** Abu Majid Samiyah **d)** Abu Samia **Low quality a.k.a.:** na **Nationality:** Kuwait **Passport no: a)** Kuwait number 106261543 **b)** Kuwait number 1420529, issued in Kuwait (and expired on 31 Mar. 2006) **National identification no:** na **Address:** Block Four, Street 13, House #179 , Kuwait City, Al-Riqqa area, Kuwait **Listed on:** 17 Feb. 2005 (amended on 23 Jul. 2008, 10 Dec. 2015, 24 Nov. 2020) **Other information:** Wanted by the Kuwaiti Security Authorities. Wanted by the Saudi security forces. Fugitive as of Jul. 2008. Review pursuant to Security Council resolution 1822 (2008) was concluded on 1 Jun. 2010. Review pursuant to Security Council resolution 2368 (2017) was concluded on 24 November 2020. INTERPOL-UN Security Council Special Notice web link: https://www.interpol.int/en/How-we-work/Notices/View-UN-Notices-Individuals click here

QDi.265 Name: 1: HAJI 2: MUHAMMAD 3: ASHRAF 4: na
Title: na **Designation:** na **DOB: a)** 1 Mar. 1965 **b)** 1955 **POB:** Faisalabad, Pakistan **Good quality a.k.a.: a)** Haji M. Ashraf **b)** Muhammad Ashraf Manshah **c)** Muhammad Ashraf Munsha **Low quality a.k.a.:** na **Nationality:** Pakistan **Passport no: a)** Pakistani number AT0712501, issued on 12 Mar. 2008 (expired 11 Mar 2013) **b)** Pakistani number A-374184 **National identification no: a)** Pakistani 6110125312507 **b)** Pakistani 24492025390 **Address:** na **Listed on:** 10 Dec. 2008 (amended on 17 Jul. 2009, 24 Jul. 2013, 1 May 2019) **Other information:** Chief of finance of Lashkar-e-Tayyiba (QDe.118). His father' s name is Noor Muhammad. Review pursuant to Security Council resolution 2253 (2015) was concluded on 21 Feb. 2019. Review pursuant to Security Council resolution 2610 (2021) was concluded on 8 November 2022. INTERPOL-UN Security Council Special Notice web link: https://www.interpol.int/en/How-we-work/Notices/View-UN-Notices-Individuals click here

QDi.364 Name: 1: ISLAM 2: SEIT-UMAROVICH 3: ATABIEV 4: na
Name (original script): Ислам Сеит-Умарович Атабиев
Title: na **Designation:** na **DOB:** 29 Sep. 1983 **POB:** Ust-Dzheguta, Republic of Karachayevo-Cherkessia, Russian Federation **Good quality a.k.a.:** na **Low quality a.k.a.:** Abu Jihad (original script: Абу Джихад) **Nationality:** Russian Federation **Passport no:** Russian foreign travel passport number 620169661 **National identification no:** Russian Federation national passport 9103314932, issued on 15 Aug. 2003 (issued by Department of the Federal Migration Service of the Russian Federation for the Republic Karachayevo-Cherkessia) **Address: a)** Moscovskiy Microrayon 6, App. 96, Ust- Dzheguta, Republic of Karachayevo-Cherkessia, Russian Federation **b)** Syrian Arab Republic (located in as at Aug. 2015) **c)** Iraq (possible alternative location as at Aug. 2015) **Listed on:** 2 Oct. 2015 (amended on 24 Nov. 2020) **Other information:** As at Aug. 2015, emir of Russian-speaking militants of the Islamic State of Iraq and the Levant (ISIL), listed as Al-Qaida in Iraq (QDe.115). Controls the Syrian Arab Republic cities of Al Dana and Idlib as an ISIL chief. Wanted by the authorities of the Russian Federation for terrorist crimes committed in its territory. Photo available for inclusion in the INTERPOL-UN Security Council Special Notice. Review pursuant to Security

Council resolution 2368 (2017) was concluded on 24 November 2020. INTERPOL-UN Security Council Special Notice web link: https://www.interpol.int/en/How-we-work/Notices/View-UN-Notices-Individuals click here

QDi.042 Name: 1: HASSAN 2: DAHIR 3: AWEYS 4: na
Name (original script): حسن ظاهرعويس
Title: a) Sheikh **b)** Colonel **Designation:** na **DOB:** 1935 **POB:** Somalia **Good quality a.k.a.: a)** Ali, Sheikh Hassan Dahir Aweys **b)** Awes, Shaykh Hassan Dahir **c)** Hassen Dahir Aweyes **d)** Ahmed Dahir Aweys **e)** Mohammed Hassan Ibrahim **f)** Aweys Hassan Dahir **g)** Hassan Tahir Oais **h)** Hassan Tahir Uways **i)** Hassan Dahir Awes **Low quality a.k.a.: a)** Sheikh Aweys **b)** Sheikh Hassan **c)** Sheikh Hassan Dahir Aweys **Nationality:** Somalia **Passport no:** na **National identification no:** na **Address: a)** (Active in Southern Somalia as of Nov. 2012.) **b)** (Also reported to be in Eritrea as of Nov. 2007.) **Listed on:** 9 Nov. 2001 (amended on 21 Dec. 2007, 11 May 2010, 16 May 2011, 18 Mar. 2013, 6 Dec. 2019) **Other information:** Family background: from the Hawiye's Habergidir, Ayr clan. Senior leader of Al-Itihaad Al-Islamiya (AIAI) (QDe.002) and Hizbul Islam in Somalia. Since 12 April 2010, also subject to the sanctions measures set out in Security Council resolution 1844 (2008) concerning Somalia and Eritrea (seehttps://www.un.org/sc/suborg/en/sanctions/751). Review pursuant to Security Council resolution 1822 (2008) was concluded on 22 Jun. 2010. Review pursuant to Security Council resolution 2368 (2017) was concluded on 4 Dec. 2019 INTERPOL-UN Security Council Special Notice web link: https://www.interpol.int/en/How-we-work/Notices/View-UN-Notices-Individuals click here

QDi.248 Name: 1: RICARDO 2: PEREZ 3: AYERAS 4: na
Title: na **Designation:** na **DOB:** 15 Sep. 1973 **POB:** 24 Paraiso Street, Barangay Poblacion, Mandaluyong City, Philippines **Good quality a.k.a.: a)** Abdul Kareem Ayeras **b)** Abdul Karim Ayeras **Low quality a.k.a.: a)** Ricky Ayeras **b)** Jimboy **c)** Isaac Jay Galang Perez **d)** Abdul Mujib **Nationality:** Philippines **Passport no:** na **National identification no:** na **Address: a)** Barangay Mangayao, Tagkawayan, Quezon, Philippines **b)** Barangay Tigib, Ayungon, Negros Oriental, Philippines **Listed on:** 4 Jun. 2008 (amended on 13 Dec. 2011, 6 Dec. 2019) **Other information:** Member of the Rajah Solaiman Movement (QDe.128). Arrested by the Philippines authorities on 14 Mar. 2011. Review pursuant to Security Council resolution 1822 (2008) was concluded on 13 May 2010. Review pursuant to Security Council resolution 2368 (2017) was concluded on 4 Dec. 2019. INTERPOL-UN Security Council Special Notice web link: https://www.interpol.int/en/How-we-work/Notices/View-UN-Notices-Individuals click here

QDi.371 Name: 1: ABD AL-BASET 2: AZZOUZ 3: na 4: na
Title: na **Designation:** na **DOB:** 7 Feb. 1966 **POB:** Doma, Libya **Good quality a.k.a.: a)** Abdelbassed Azouz **b)** Abdul Baset Azouz **Low quality a.k.a.:** AA (initials) **Nationality:** Libya **Passport no: a)** Libya number 223611 **b)** British passport number C00146605 **National identification no:** na **Address:** Libya (last known location) **Listed on:** 29 Feb. 2016 (amended on 24 Nov. 2020) **Other information:** Key operative in Al-Qaida (QDe.004). Under the direction of Aiman al-Zawahiri (QDi.006), recruited 200 militants in the eastern part of Libya. Review pursuant to Security Council resolution 2368 (2017) was concluded on 24 November 2020. INTERPOL-UN Security Council Special Notice web link: https://www.interpol.int/en/How-we-work/Notices/View-UN-Notices-Individuals click here

QDi.305 Name: 1: ABDUL 2: ROSYID 3: RIDHO 4: BA'ASYIR
Title: na **Designation:** na **DOB:** 31 Jan. 1974 **POB:** Sukoharjo, Indonesia **Good quality a.k.a.: a)** Abdul Rosyid Ridho Bashir **b)** Rashid Rida Ba' aysir **c)** Rashid Rida Bashir **Low quality a.k.a.:** na **Nationality:** Indonesia **Passport no:** na **National identification no:** (Indonesian National Identity Card number 1127083101740003 under name Abdul Rosyid Ridho Ba' asyir) **Address:** Podok Pesantren AL Wayain Ngrandu, Sumber Agung Magetan, East Java, Indonesia **Listed on:** 12 Mar. 2012 **Other information:** Father's name is Abu Bakar Ba'asyir (QDi.217). Brother of Abdul Rahim Ba' aysir (QDi.293). Belongs to the leadership of and is involved in recruitment and

fundraising for Jemmah Anshorut Tauhid (JAT) (QDe.133) Associated with Jemaah Islamiyah (QDe.092). Review pursuant to Security Council resolution 2368 (2017) was concluded on 15 November 2021. INTERPOL-UN Security Council Special Notice web link: https://www.interpol.int/en/How-we-work/Notices/View-UN-Notices-Individuals click here

QDi.217 Name: 1: ABU BAKAR 2: BA'ASYIR 3: na 4: na
Title: na **Designation:** na **DOB:** 17 Aug. 1938 **POB:** Jombang, East Java, Indonesia **Good quality a.k.a.: a)** Abu Bakar Baasyir born 17 Aug. 1938 in Jombang, East Java, Indonesia **b)** Abu Bakar Bashir born 17 Aug. 1938 in Jombang, East Java, Indonesia **c)** Abdus Samad **d)** Abdus Somad **Low quality a.k.a.:** na **Nationality:** Indonesia **Passport no:** na **National identification no:** na **Address:** Indonesia **Listed on:** 21 Apr. 2006 (amended on 14 Oct. 2015, 24 Nov. 2020, 6 Apr. 2021) **Other information:** Formed Jemmah Anshorut Tauhid (JAT) (QDe.133) in 2008. In 2010, arrested for incitement to commit terrorism and fundraising with respect to a training camp in Aceh, Indonesia and sentenced to 15 years in 2011. Ba'asyir was released from prison on 8 January 2021 after serving his sentence in accordance with Indonesian laws and regulations. Review pursuant to Security Council resolution 1822 (2008) was concluded on 8 Jun. 2010. Review pursuant to Security Council resolution 2368 (2017) was concluded on 24 November 2020. INTERPOL-UN Security Council Special Notice web link: https://www.interpol.int/en/How-we-work/Notices/View-UN-Notices-Individuals click here

QDi.293 Name: 1: ABDUL RAHIM 2: BA'AYSIR 3: na 4: na
Title: na **Designation:** na **DOB: a)** 16 Nov. 1977 **b)** 16 Nov. 1974 **POB: a)** Solo, Indonesia **b)** Sukoharjo, Central Java, Indonesia **Good quality a.k.a.: a)** Abdul Rahim Bashir **b)** 'Abd Al-Rahim Ba'asyir **c)** 'Abd Al-Rahim Bashir **d)** Abdurrahim Ba'asyir **e)** Abdurrahim Bashir **f)** Abdul Rachim Ba'asyir **g)** Abdul Rachim Bashir **h)** Abdul Rochim Ba'asyir **i)** Abdul Rochim Bashir **j)** Abdurochim Ba'asyir **k)** Abdurochim Bashir **l)** Abdurrochim Ba'asyir **m)** Abdurrochim Bashir **n)** Abdurrahman Ba'asyir **o)** Abdurrahman Bashir **Low quality a.k.a.:** na **Nationality:** Indonesia **Passport no:** na **National identification no:** na **Address:** Indonesia **Listed on:** 19 Jul. 2011 (amended on 24 Nov. 2020) **Other information:** Senior Jemaah Islamiyah (QDe.092.) leader. Father's name is Abu Bakar Ba'asyir (QDi.217). Review pursuant to Security Council resolution 2368 (2017) was concluded on 24 November 2020. INTERPOL-UN Security Council Special Notice web link: https://www.interpol.int/en/How-we-work/Notices/View-UN-Notices-Individuals click here

QDi.080 Name: 1: SAID 2: BAHAJI 3: na 4: na
Name (original script): سعيد باهاجى
Title: na **Designation:** na **DOB:** 15 Jul. 1975 **POB:** Haselünne, Lower Saxony, Germany **Good quality a.k.a.:** Zouheir Al Maghribi **Low quality a.k.a.: a)** Mohamed Abbattay **b)** Abderrahmane Al Maghribi **Nationality: a)** Germany **b)** Morocco **Passport no: a)** Germany number Provisional passport No.: 28642163, issued in Hamburg **b)** Morocco number 954242, issued on 28 Jun. 1995, issued in Meknas, Morocco (expired) **National identification no:** Germany Identity document ("Bundespersonalausweis") 1336597587 **Address:** Bunatwiete 23, Hamburg, 21073, Germany (formerly resident at) **Listed on:** 30 Sep. 2002 (amended on 26 Nov. 2004, 9 Sep. 2005, 2 Jul. 2007, 23 Dec. 2010, 9 Sep. 2014, 1 May 2019) **Other information:** Deputy head of the media committee of Al-Qaida (QDe.004) as at Apr. 2010. German authorities issued an arrest warrant for him on 21 Sep. 2001. Review pursuant to Security Council resolution 1822 (2008) was concluded on 20 May 2010. Reportedly deceased in September 2013 in the Afghanistan/Pakistan border area. Review pursuant to Security Council resolution 2253 (2015) was concluded on 21 Feb. 2019. Review pursuant to Security Council resolution 2368 (2017) was concluded on 15 November 2021. INTERPOL-UN Security Council Special Notice web link: https://www.interpol.int/en/How-we-work/Notices/View-UN-Notices-Individuals click here

QDi.266 Name: 1: MAHMOUD 2: MOHAMMAD 3: AHMED 4: BAHAZIQ
Title: na **Designation:** na **DOB: a)** 17 Aug. 1943 **b)** 1943 **c)** 1944 **POB:** India **Good quality a.k.a.: a)** Bahaziq Mahmoud **b)** Abu Abd al- 'Aziz **c)** Abu Abdul Aziz **d)** Shaykh Sahib **Low quality a.k.a.:**

na **Nationality:** Saudi Arabia **Passport no:** na **National identification no:** Saudi Arabia 4-6032-0048-1 **Address:** Saudi Arabia **Listed on:** 10 Dec. 2008 (amended on 1 May 2019, 2 Feb. 2023) **Other information:** Financier of Lashkar-e-Tayyiba (listed under permanent reference number QDe.118). Has served as the leader of Lashkar-e-Tayyiba in Saudi Arabia. Review pursuant to Security Council resolution 2253 (2015) was concluded on 21 Feb. 2019. INTERPOL-UN Security Council Special Notice web link: https://www.interpol.int/en/How-we-work/Notices/View-UN-Notices-Individuals click here

QDi.311 Name: 1: AYYUB 2: BASHIR 3: na 4: na
Name (original script): ایوب بشیر
Title: a) Qari **b)** Alhaj **Designation:** na **DOB: a)** 1966 **b)** 1964 **c)** 1969 **d)** 1971 **POB:** na **Good quality a.k.a.: a)** Alhaj Qari Ayub Bashar **b)** Qari Muhammad Ayub **Low quality a.k.a.:** na **Nationality: a)** Uzbekistan **b)** Afghanistan **Passport no:** na **National identification no:** na **Address:** Mir Ali, North Waziristan Agency, Federal Administered Tribal Areas, Pakistan **Listed on:** 18 Oct. 2012 (amended on 17 Jul. 2018, 1 May 2019) **Other information:** Member of leadership council as of early 2010 and head of finance for the Islamic Movement of Uzbekistan (QDe.010). Coordinated financial and logistical support for the Islamic Movement of Uzbekistan in Afghanistan and Pakistan between 2009-2012. Transferred and delivered funds to Fazal Rahim (QDi.303). Reportedly deceased in an airstrike in Chordar, Kunduz Province of Afghanistan in Dec. 2015. Review pursuant to Security Council resolution 2253 (2015) was concluded on 7 Jun. 2018. Review pursuant to Security Council resolution 2253 (2015) was concluded on 21 Feb. 2019. Review pursuant to Security Council resolution 2368 (2017) was concluded on 15 November 2021. INTERPOL-UN Security Council Special Notice web link: https://www.interpol.int/en/How-we-work/Notices/View-UN-Notices-Individuals click here

QDi.055 Name: 1: MAHMOOD 2: SULTAN 3: BASHIR-UD-DIN 4: na
Title: na **Designation:** na **DOB: a)** 1937 **b)** 1938 **c)** 1939 **d)** 1940 **e)** 1941 **f)** 1942 **g)** 1943 **h)** 1944 **i)** 1945 **POB:** na **Good quality a.k.a.: a)** Mahmood, Sultan Bashiruddin **b)** Mehmood, Dr. Bashir Uddin **c)** Mekmud, Sultan Baishiruddin **Low quality a.k.a.:** na **Nationality:** Pakistan **Passport no:** na **National identification no:** na **Address:** Street 13, Wazir Akbar Khan, Kabul, Afghanistan **Listed on:** 24 Dec. 2001 (amended on 1 May 2019) **Other information:** Review pursuant to Security Council resolution 1822 (2008) was concluded on 1 Jun. 2010. Review pursuant to Security Council resolution 2253 (2015) was concluded on 21 Feb. 2019. Review pursuant to Security Council resolution 2610 (2021) was concluded on 8 November 2022. INTERPOL-UN Security Council Special Notice web link: https://www.interpol.int/en/How-we-work/Notices/View-UN-Notices-Individuals click here

QDi.345 Name: 1: TARKHAN 2: TAYUMURAZOVICH 3: BATIRASHVILI 4: na
Title: na **Designation:** na **DOB: a)** 11 Jan. 1986 **b)** 1982 **POB:** Akhmeta, Village Birkiani, Georgia **Good quality a.k.a.: a)** Tarkhan Tayumurazovich Batyrashvili **b)** Tarkhan Batirashvili **Low quality a.k.a.: a)** Omar Shishani **b)** Umar Shishani **c)** Abu Umar al-Shishani **d)** Omar al-Shishani **e)** Chechen Omar **f)** Omar the Chechen **g)** Omer the Chechen **h)** Umar the Chechen **i)** Abu Umar **j)** Abu Hudhayfah **Nationality:** Georgia **Passport no:** 09AL14455, issued in Georgia (expires on 26 Jun. 2019) **National identification no:** 08001007864, issued in Georgia **Address:** Syrian Arab Republic (located in as at Dec. 2014) **Listed on:** 23 Jan. 2015 (amended on 24 Nov. 2020) **Other information:** As of mid-2014, Syria-based senior military commander and shura council member of Islamic State in Iraq and the Levant, listed as Al-Qaida in Iraq (AQI) (QDe.115). Led approximately 1,000 foreign fighters for ISIL and committed a number of attacks in northern Syria. Review pursuant to Security Council resolution 2368 (2017) was concluded on 24 November 2020. INTERPOL-UN Security Council Special Notice web link: https://www.interpol.int/en/How-we-work/Notices/View-UN-Notices-Individuals click here

QDi.279 Name: 1: MOHAMED 2: BELKALEM 3: na 4: na
Name (original script): محمد بلكلام

Title: na **Designation:** na **DOB:** 19 Dec. 1969 **POB:** Hussein Dey, Algiers, Algeria **Good quality a.k.a.:** na **Low quality a.k.a.:** **a)** Abdelali Abou Dher (عبد العالي ابو ذر) **b)** El Harrachi (الحراشي) **Nationality:** Algerian **Passport no:** na **National identification no:** na **Address:** Mali **Listed on:** 22 Apr. 2010 (amended on 15 Apr. 2014, 6 Dec. 2019) **Other information:** Convicted in absentia by Algerian tribunal on 28 Mar. 1996. Algerian international arrest warrant number 03/09 of 6 Jun. 2009 issued by the Tribunal of Sidi Mhamed, Algiers, Algeria. Algerian extradition request number 2307/09 of 3 Sep. 2009, presented to Malian authorities. Father' s name is Ali Belkalem. Mother' s name is Fatma Saadoudi. Member of The Organization of Al-Qaida in the Islamic Maghreb (QDe.014). Review pursuant to Security Council resolution 2368 (2017) was concluded on 4 Dec. 2019. INTERPOL-UN Security Council Special Notice web link: https://www.interpol.int/en/How-we-work/Notices/View-UN-Notices-Individuals click here

QDi.136 Name: 1: MOKHTAR 2: BELMOKHTAR 3: na 4: na
Name (original script): مختار بلمختار
Title: na **Designation:** na **DOB:** 1 Jun. 1972 **POB:** Ghardaia, Algeria **Good quality a.k.a.:** **a)** Abou Abbes Khaled **b)** Belaouar Khaled Abou El Abass **c)** Belaouer Khaled Abou El Abass **d)** Belmokhtar Khaled Abou El Abes **e)** Khaled Abou El Abass **f)** Khaled Abou El Abbes **g)** Khaled Abou El Abes **h)** Khaled Abulabbas Na Oor **i)** Mukhtar Belmukhtar **Low quality a.k.a.:** **a)** Belaoua **b)** Belaour **Nationality:** Algeria **Passport no:** na **National identification no:** na **Address:** na **Listed on:** 11 Nov. 2003 (amended on 12 Apr. 2006, 2 Jul. 2007, 7 Apr. 2008, 25 Jan. 2010, 16 May 2011, 9 Sep. 2014, 1 May 2019, 2 Feb. 2023) **Other information:** Reportedly deceased in November 2016. Father's name is Mohamed. Mother's name is Zohra Chemkha. Member of the Council of the Organization of Al-Qaida in the Islamic Maghreb (QDe.014) (AQIM). Head of Al Mouakaoune Biddam (QDe.139), Al Moulathamoun (QDe.140) and Al Mourabitoun (QDe.141). Review pursuant to Security Council resolution 1822 (2008) was concluded on 30 Jul. 2009. Review pursuant to Security Council resolution 2253 (2015) was concluded on 21 Feb. 2019. INTERPOL-UN Security Council Special Notice web link: https://www.interpol.int/en/How-we-work/Notices/View-UN-Notices-Individuals click here

QDi.375 Name: 1: BOUBAKER 2: BEN HABIB 3: BEN AL-HAKIM 4: na
Title: na **Designation:** na **DOB:** 1 Aug. 1983 **POB:** Paris, France **Good quality a.k.a.:** **a)** Boubakeur el-Hakim **b)** Boubaker el Hakim **Low quality a.k.a.:** **a)** Abou al Moukatel **b)** Abou Mouqatel **c)** Abu-Muqatil al-Tunisi **d)** El Hakim Boubakeur **Nationality:** **a)** France **b)** Tunisia **Passport no:** na **National identification no:** na **Address:** Syrian Arab Republic (as at Sep. 2015) **Listed on:** 29 Sep. 2015 (amended on 24 Jun. 2016) **Other information:** French-Tunisian foreign terrorist fighter for Islamic State in Iraq and the Levant (ISIL), listed as Al-Qaida in Iraq (QDe.115). Review pursuant to Security Council resolution 2368 (2017) was concluded on 15 November 2021. INTERPOL-UN Security Council Special Notice web link: https://www.interpol.int/en/How-we-work/Notices/View-UN-Notices-Individuals click here

QDi.355 Name: 1: SOFIANE 2: BEN GOUMO 3: na 4: na
Title: na **Designation:** na **DOB:** 26 Jun. 1959 **POB:** Derna, Libya **Good quality a.k.a.:** Sufyan bin Qumu **Low quality a.k.a.:** Abou Fares al Libi **Nationality:** Libya **Passport no:** na **National identification no:** na **Address:** Libya **Listed on:** 3 Sep. 2015 (amended on 24 Nov. 2020) **Other information:** Leader of Ansar al Charia Derna (QDe.145). Review pursuant to Security Council resolution 2368 (2017) was concluded on 24 November 2020. INTERPOL-UN Security Council Special Notice web link: https://www.interpol.int/en/How-we-work/Notices/View-UN-Notices-Individuals click here

QDi.333 Name: 1: SEIFALLAH 2: BEN OMAR 3: BEN MOHAMED 4: BEN HASSINE
Name (original script): سيف الله بن عمر بن محمد بنحسين
Title: na **Designation:** na **DOB:** 8 Nov. 1965 **POB:** Tunis, Tunisia **Good quality a.k.a.:** **a)** Seif Allah ben Hocine **b)** Saifallah ben Hassine **c)** Sayf Allah 'Umar bin Hassayn **d)** Seifallah ben Amor ben Hassine **e)** Sayf Allah bin Hussayn **Low quality a.k.a.:** **a)** Abu Iyyadh al-Tunisi **b)** Abou Iyadh el-

Tounsi **c)** Abu Ayyad al-Tunisi **d)** Abou Aayadh **e)** Abou Iyadh **Nationality:** Tunisia **Passport no:** Tunisia number G557170, issued on 16 Nov. 1989 **National identification no:** Tunisia National Identification Card 05054425, issued on 3 May 2011 (issued in Hammam Lif) **Address: a)** 60 Rue de la Libye, Hammam Lif, Ben Arous, Tunisia **b)** Libya (possible location as at Jul. 2017) **Listed on:** 23 Sep. 2014 (amended on 15 Feb. 2017, 26 Dec. 2017, 24 Nov. 2020) **Other information:** Founder of the Tunisian Combatant Group (QDe.090) and leader of Ansar al-Shari'a in Tunisia (AAS-T) (QDe.143). Arrest warrant issued by Tunisian Court of First Instance on 23 Aug. 2013. Review pursuant to Security Council resolution 2368 (2017) was concluded on 24 November 2020. INTERPOL-UN Security Council Special Notice web link: https://www.interpol.int/en/How-we-work/Notices/View-UN-Notices-Individuals click here

QDi.386 Name: 1: MOUNIR 2: BEN DHAOU 3: BEN BRAHIM 4: BEN HELAL
Title: na **Designation:** na **DOB:** 10 May 1983 **POB:** Ben Guerdane, Tunisia **Good quality a.k.a.:** na **Low quality a.k.a.: a)** Mounir Helel **b)** Mounir Hilel **c)** Abu Rahmah **d)** Abu Maryam al-Tunisi **Nationality:** Tunisia **Passport no:** na **National identification no:** 08619445 **Address:** Amria Ben Guerdane, Medenine, Tunisia **Listed on:** 29 Feb. 2016 (amended on 27 May 2022) **Other information:** Foreign terrorist fighter facilitator experienced in establishing and securing travel routes. Deeply involved in providing material support to the Organization of Al-Qaida in the Islamic Maghreb (QDe.014) in North Africa. Assisted foreign terrorist fighters' travel throughout North Africa and to Syrian Arab Republic to join Islamic State in Iraq and the Levant, listed as Al-Qaida in Iraq (QDe.115). Profession: farm worker. Mother's name: Mbarka Helali. Review pursuant to Security Council resolution 2368 (2017) was concluded on 15 November 2021. INTERPOL-UN Security Council Special Notice web link: https://www.interpol.int/en/How-we-work/Notices/View-UN-Notices-Individuals click here

QDi.388 Name: 1: SALIM 2: BENGHALEM 3: na 4: na
Title: na **Designation:** na **DOB:** 6 Jul. 1980 **POB:** Bourg la Reine, France **Good quality a.k.a.:** na **Low quality a.k.a.:** na **Nationality:** France **Passport no:** na **National identification no:** na **Address:** Syrian Arab Republic (as at Sep. 2015) **Listed on:** 29 Feb. 2016 **Other information:** Syria-based French violent extremist and member of Islamic State in Iraq and the Levant, listed as Al-Qaida in Iraq (QDe.115). Subject to a European Arrest Warrant. Review pursuant to Security Council resolution 2368 (2017) was concluded on 15 November 2021. INTERPOL-UN Security Council Special Notice web link: https://www.interpol.int/en/How-we-work/Notices/View-UN-Notices-Individuals click here

QDi.307 Name: 1: HAFIZ 2: ABDUL SALAM 3: BHUTTAVI 4: na
Title: a) Maulavi **b)** Mullah **Designation:** na **DOB:** 1940 **POB:** Gujranwala, Punjab Province, Pakistan **Good quality a.k.a.: a)** Hafiz Abdul Salam Bhattvi **b)** Hafiz Abdusalam Budvi **c)** Hafiz Abdussalaam Bhutvi **d)** Abdul Salam Budvi **e)** Abdul Salam Bhattwi **f)** Abdul Salam Bhutvi **g)** Mullah Abdul Salaam Bhattvi **h)** Molvi Abdursalam Bhattvi **Low quality a.k.a.:** na **Nationality:** Pakistan **Passport no:** na **National identification no:** na **Address:** na **Listed on:** 14 Mar. 2012 **Other information:** Founding member of Lashkar-e-Tayyiba (QDe.118) and deputy to Lashkar-e-Tayyiba leader Hafiz Muhammad Saeed (QDi.263). Review pursuant to Security Council resolution 2368 (2017) was concluded on 15 November 2021. INTERPOL-UN Security Council Special Notice web link: https://www.interpol.int/en/How-we-work/Notices/View-UN-Notices-Individuals click here

QDi.421 Name: 1: HAMZA 2: USAMA 3: MUHAMMAD 4: BIN LADEN
Name (original script): حمزة أسامة محمد بن لادن
Title: na **Designation:** na **DOB:** 9 May 1989 **POB:** Jeddah, Saudi Arabia **Good quality a.k.a.:** na **Low quality a.k.a.:** na **Nationality:** na **Passport no:** na **National identification no:** na **Address:** na **Listed on:** 28 Feb. 2019 (amended on 13 Mar. 2019) **Other information:** Son of Usama bin Laden (deceased). Announced by Aiman Muhammed Rabi al-Zawahiri (QDi.006) as an official member of Al-Qaida (QDe.004). Has called for followers of Al-Qaida to commit terror attacks. Is

seen as the most probable successor of al-Zawahiri. INTERPOL-UN Security Council Special Notice web link: https://www.interpol.int/en/How-we-work/Notices/View-UN-Notices-Individuals click here

QDi.009 Name: 1: BILAL 2: BIN MARWAN 3: na 4: na
Name (original script): بلال بن مروان
Title: na **Designation:** na **DOB:** 1947 **POB:** na **Good quality a.k.a.:** na **Low quality a.k.a.:** na **Nationality:** na **Passport no:** na **National identification no:** na **Address:** na **Listed on:** 25 Jan. 2001 (amended on 1 May 2019) **Other information:** Senior lieutenant of UBL. Review pursuant to Security Council resolution 1822 (2008) was concluded on 21 Jun. 2010. Review pursuant to Security Council resolution 2253 (2015) was concluded on 21 Feb. 2019. Review pursuant to Security Council resolution 2610 (2021) was concluded on 8 November 2022. INTERPOL-UN Security Council Special Notice web link: https://www.interpol.int/en/How-we-work/Notices/View-UN-Notices-Individuals click here

QDi.417 Name: 1: MOHAMAD 2: RAFI 3: BIN UDIN 4: na
Title: na **Designation:** na **DOB:** 3 Jun. 1966 **POB:** Negri Sembilan, Malaysia **Good quality a.k.a.:** na **Low quality a.k.a.: a)** Mohd Radi Bin Udin **b)** Abu Awn al Malizi **c)** Muhammad Ratin **d)** Muhammad Rafiuddin **e)** Abu Una al Malayzie **f)** Mhammad Rahim Bin Udin **g)** Abu Ayn Tok Cit **h)** Muhammad Ratin Bin Nurdin **Nationality: a)** Malaysia **b)** Indonesia **Passport no:** Malaysia number A31142734, issued on 6 Nov. 2013 (issued by the Immigration Department of Malaysia, expiration date 6 Nov. 2015) **National identification no:** Malaysia National Identification Card 660603-05-5267 (issued by National Registration Department of Malaysia; issued to Mohd Rafi bin Udin) **Address: a)** B-3B-19 Glenview Villa, Jalan 49 Off Jalan Kuari, Taman Pinggiran Cheras, 56000, Kuala Lumpur, Wilayah Persekutuan Kuala Lumpur, Malaysia (as at 30 Jan. 2014) **b)** 90-00-04 Flat Sri Kota, Bandar Tun Razak, 56100, Kuala Lumpur, Wilayah Persekutuan Kuala Lumpur, Malaysia (as at 23 Apr. 2010) **c)** 96-06-06 Flat Sri Kota, Bandar Tun Razak, 56100, Kuala Lumpur, Wilayah Persekutuan Kuala Lumpur, Malaysia (as at 6 Apr. 2007) **d)** Syrian Arab Republic (location since 2014) **Listed on:** 23 Aug. 2018 **Other information:** Senior member of Islamic State in Iraq and the Levant (ISIL), listed as Al-Qaida in Iraq (QDe.115). Recruited for ISIL and instructed individuals to perpetrate terrorist acts via online video. Physical description: eye colour: brown; hair colour: brown; complexion: dark. Speaks Malay, English, limited Arabic. INTERPOL-UN Security Council Special Notice web link: https://www.interpol.int/en/How-we-work/Notices/View-UN-Notices-Individuals click here

QDi.081 Name: 1: RAMZI 2: MOHAMED 3: ABDULLAH 4: BINALSHIBH
Name (original script): رمزي محمد عبد الله بن الشيبة
Title: na **Designation:** na **DOB: a)** 1 May 1972 **b)** 16 Sep. 1973 **POB: a)** Gheil Bawazir, Hadramawt, Yemen **b)** Khartoum, Sudan **Good quality a.k.a.: a)** Binalsheidah, Ramzi Mohamed Abdullah **b)** Bin Al Shibh, Ramzi **c)** Omar, Ramzi Mohamed Abdellah **d)** Mohamed Ali Abdullah Bawazir **e)** Binalshibh Ramzi Mohammed Abdullah **f)** Ramzi Binalshib **g)** Ramzi Mohamed Abdellah Omar Hassan Alassiri **h)** Binalshibh Ramsi Mohamed Abdullah **i)** Abu Ubaydah **j)** 'Umar Muhammad 'Abdallah Ba' Amar **Low quality a.k.a.:** Ramzi Omar **Nationality:** Yemen **Passport no:** 00085243, issued on 17 Nov. 1997, issued in Sanaa, Yemen **National identification no:** na **Address:** na **Listed on:** 30 Sep. 2002 (amended on 26 Nov. 2004, 25 Jul. 2006, 2 Jul. 2007, 27 Jul. 2007, 23 Dec. 2010, 6 Dec. 2019) **Other information:** Arrested in Karachi, Pakistan, 30 Sep. 2002. In custody of the United States of America, as of May 2010. Review pursuant to Security Council resolution 1822 (2008) was concluded on 25 May 2010. Review pursuant to Security Council resolution 2368 (2017) was concluded on 4 Dec. 2019 INTERPOL-UN Security Council Special Notice web link: https://www.interpol.int/en/How-we-work/Notices/View-UN-Notices-Individuals click here

QDi.188 Name: 1: FAYCAL 2: BOUGHANEMI 3: na 4: na
Name (original script): فيصل بوغانمي
Title: na **Designation:** na **DOB:** 28 Oct. 1966 **POB:** Tunis, Tunisia **Good quality a.k.a.: a)** Faical Boughanmi **b)** Faysal al-Bughanimi **Low quality a.k.a.:** na **Nationality:** Tunisia **Passport no:** na **National identification no:** na **Address:** Number 5/B viale Cambonino, Cremona, Italy **Listed**

on: 29 Jul. 2005 (amended on 7 Jun. 2007, 10 Aug. 2009, 1 Sep. 2009, 13 Dec. 2011, 1 May 2019) **Other information:** Italian Fiscal code: BGHFCL66R28Z352G. Sentenced to 7 years imprisonment in Italy on 29 Jun. 2007 by the Brescia Second Appeals Court. In detention in Italy as at Jun. 2009. Review pursuant to Security Council resolution 1822 (2008) was concluded on 20 Jul. 2009. Review pursuant to Security Council resolution 2253 (2015) was concluded on 21 Feb. 2019. INTERPOL-UN Security Council Special Notice web link: https://www.interpol.int/en/How-we-work/Notices/View-UN-Notices-Individuals click here

QDi.058 Name: 1: BOUBEKEUR 2: BOULGHITI 3: na 4: na
Title: na **Designation:** na **DOB:** 13 Feb. 1970 **POB:** Rouiba, Algiers, Algeria **Good quality a.k.a.:** Boubakeur Boulghit **Low quality a.k.a.: a)** Abu Bakr al-Jaziri (Previously listed as. In Arabic: أبوبكر الجزائري) **b)** Abou Bakr Al Djazairi **c)** Abou Yasser El Djazairi **d)** Yasir Al-Jazari **e)** Abou Yasser Al-Jaziri **Nationality: a)** Algeria **b)** Palestinian **Passport no:** na **National identification no:** na **Address:** Algeria **Listed on:** 11 Jan. 2002 (amended on 18 Jul. 2007, 1 Feb. 2008, 16 May 2011, 20 Jun. 2017, 1 May 2019, 2 Feb. 2023) **Other information:** Finance chief of the Afghan Support Committee (ASC) (QDe.069). Al-Qaida (QDe.004) facilitator and communication expert. Believed to be in Algeria as of Apr. 2010 and May 2022. Son of Mohamed and Fatma Aribi. Review pursuant to Security Council resolution 1822 (2008) was concluded on 21 Jun. 2010. Review pursuant to Security Council resolution 2253 (2015) was concluded on 21 Feb. 2019. INTERPOL-UN Security Council Special Notice web link: https://www.interpol.int/en/How-we-work/Notices/View-UN-Notices-Individuals click here

QDi.143 Name: 1: HAMADI 2: BEN ABDUL AZIZ 3: BEN ALI 4: BOUYEHIA
Name (original script): حمادي بن عبد العزيز بن علي بويحي
Title: na **Designation:** na **DOB:** 29 May 1966 **POB:** Tunis, Tunisia **Good quality a.k.a.: a)** Gamel Mohamed born 25 May 1966 in Morocco **b)** Abd el Wanis Abd Gawwad Abd el Latif Bahaa born 9 May 1986 in Egypt **c)** Mahmoud Hamid **Low quality a.k.a.:** na **Nationality:** Tunisia **Passport no:** (Tunisian passport number L723315, issued on 5 May 1998, expired on 4 May 2003) **National identification no:** na **Address:** Corso XXII Marzo Number 39, Milan, Italy **Listed on:** 12 Nov. 2003 (amended on 20 Dec. 2005, 31 Jul. 2006, 30 Jan. 2009, 16 May 2011, 6 Dec. 2019, 10 Sep. 2020) **Other information:** In prison in Italy until 6 February 2026. Review pursuant to Security Council resolution 1822 (2008) was concluded on 21 Jun. 2010. Review pursuant to Security Council resolution 2368 (2017) was concluded on 4 Dec. 2019. INTERPOL-UN Security Council Special Notice web link: https://www.interpol.int/en/How-we-work/Notices/View-UN-Notices-Individuals click here

QDi.396 Name: 1: ASLAN 2: AVGAZAROVICH 3: BYUTUKAEV 4: na
Name (original script): Аслан Авгазарович Бютукаев
Title: na **Designation:** na **DOB:** 22 Oct. 1974 **POB:** Kitaevka, Novoselitskiy District, Stavropol Region, Russian Federation **Good quality a.k.a.:** na **Low quality a.k.a.: a)** Amir Khazmat (original script: Амир Хазмат) **b)** Abubakar (original script: Абубакар) **Nationality:** Russian Federation **Passport no:** na **National identification no:** na **Address:** Akharkho Street, 11, Katyr-Yurt, Achkhoy-Martanovskiy District, Republic of Chechnya, Russian Federation **Listed on:** 3 Aug. 2016 **Other information:** Wanted by the authorities of the Russian Federation for terrorist crimes. Commands a suicide battalion of Riyadus-Salikhin Reconnaissance and Sabotage Battalion of Chechen Martyrs (RSRSBCM) (QDe.100). Review pursuant to Security Council resolution 2368 (2017) was concluded on 15 November 2021. INTERPOL-UN Security Council Special Notice web link: https://www.interpol.int/en/How-we-work/Notices/View-UN-Notices-Individuals click here

QDi.405 Name: 1: Malik 2: Ruslanovich 3: Barkhanoev 4: na
Title: na **Designation:** na **DOB:** 14 Mar. 1992 **POB:** Ordzhonikidzevskaya village, Sunzhenskiy district, Ingushetia, Russian Federation **Good quality a.k.a.:** na **Low quality a.k.a.: a)** Saifuddin **b)** Saifuddin al-Ingushi **c)** Saifuddin Ingushi **Nationality:** Russian Federation **Passport no:** na **National identification no:** na **Address:** Mosul, Iraq **Listed on:** 20 Jul. 2017 **Other information:** Joined the

Islamic State in Iraq and the Levant (ISIL), listed as Al-Qaida in Iraq (QDe.115) in September 2016. Review pursuant to Security Council resolution 2610 (2021) was concluded on 8 November 2022. INTERPOL-UN Security Council Special Notice web link: https://www.interpol.int/en/How-we-work/Notices/View-UN-Notices-Individuals click here

QDi.365 Name: 1: AKHMED 2: RAJAPOVICH 3: CHATAEV 4: na
Name (original script): Ахмед Ражапович Чатаев
Title: na **Designation:** na **DOB:** 4 Jul. 1980 **POB:** Vedeno Village, Vedenskiy District, Republic of Chechnya, Russian Federation **Good quality a.k.a.: a)** Akhmad Shishani (original script: Ахмад Шишани) **b)** David Mayer (original script: Давид Майер) **c)** Elmir Sene (original script: Эльмир Сене) **Low quality a.k.a.:** Odnorukiy (original script: Однорукий) **Nationality:** Russian Federation **Passport no:** na **National identification no:** Russian Federation national passport 9600133195, issued in Vedensiky District, Republic of Chechnya, Russian Federation (issued by Department of Internal Affairs) **Address: a)** Syrian Arab Republic (located in as at Aug. 2015) **b)** Iraq (possible alternative location as at Aug. 2015) **Listed on:** 2 Oct. 2015 (amended on 30 Dec. 2015, 10 Dec. 2015, 24 Nov. 2020) **Other information:** As at Aug. 2015, one of the leaders of the Islamic State in Iraq and the Levant (ISIL), listed as Al-Qaida in Iraq (QDe.115), commanding directly 130 militants. Physical description: eye colour: brown, hair colour: black, build: solid; distinguishing marks: oval face, beard, missing a right hand and left leg, speaks Russian, Chechen and possibly German and Arabic. Wanted by the authorities of the Russian Federation for terrorist crimes committed in its territory. Photo available for inclusion in the INTERPOL-UN Security Council Special Notice. Review pursuant to Security Council resolution 2368 (2017) was concluded on 24 November 2020. INTERPOL-UN Security Council Special Notice web link: https://www.interpol.int/en/How-we-work/Notices/View-UN-Notices-Individuals click here

QDi.312 Name: 1: AAMIR 2: ALI 3: CHAUDHRY 4: na
Name (original script): عامر علی چوہدری
Title: na **Designation:** na **DOB:** 3 Aug. 1986 **POB:** na **Good quality a.k.a.: a)** Aamir Ali Chaudary **b)** Aamir Ali Choudry **c)** Amir Ali Chaudry **Low quality a.k.a.:** Huzaifa **Nationality:** Pakistan **Passport no:** Pakistani number BN 4196361, issued on 28 Oct. 2008 (expiring 27 Oct. 2013) **National identification no:** Pakistani 33202-7126636-9 **Address:** na **Listed on:** 18 Oct. 2012 (amended on 1 May 2019, 2 Feb. 2023) **Other information:** Electronics and explosives expert for Tehrik-e Taliban Pakistan (TTP) (QDe.132). Involved in attack planning for TTP. Provided financial and logistical support for TTP and participated in TTP-sponsored militant training. Reportedly deceased. Review pursuant to Security Council resolution 2253 (2015) was concluded on 21 Feb. 2019. INTERPOL-UN Security Council Special Notice web link: https://www.interpol.int/en/How-we-work/Notices/View-UN-Notices-Individuals click here

QDi.070 Name: 1: YASSINE 2: CHEKKOURI 3: na 4: na
Name (original script): ياسين شكوري
Title: na **Designation:** na **DOB:** 6 Oct. 1966 **POB:** Safi, Morocco **Good quality a.k.a.:** na **Low quality a.k.a.:** na **Nationality:** Morocco **Passport no:** Moroccan number F46947 **National identification no:** Morocco H-135467 **Address:** 7th Street, Number 7, Hay Anas Safi, Morocco **Listed on:** 3 Sep. 2002 (amended on 7 Jun. 2007, 23 Dec. 2010, 6 Dec. 2019) **Other information:** Mother' s name is Feue Hlima Bent Barka and father' s name is Abderrahmane Mohammed Ben Azzouz. Deported from Italy to Morocco on 26 Feb. 2004. Review pursuant to Security Council resolution 1822 (2008) was concluded on 21 Jun. 2010. Review pursuant to Security Council resolution 2368 (2017) was concluded on 4 Dec. 2019 INTERPOL-UN Security Council Special Notice web link: https://www.interpol.int/en/How-we-work/Notices/View-UN-Notices-Individuals click here

QDi.376 Name: 1: PETER 2: CHERIF 3: na 4: na
Title: na **Designation:** na **DOB:** 26 Aug. 1982 **POB:** Paris, 20th district, France **Good quality a.k.a.:** na **Low quality a.k.a.:** na **Nationality:** France **Passport no:** na **National identification no:** na **Address:** Al Mukalla, Hadramawt province, Yemen **Listed on:** 29 Sep. 2015 (amended on 24

Jun. 2016) **Other information:** Member of Al-Qaida in the Arabian Peninsula (AQAP) (QDe.129). Convicted in absentia to five years in prison in France in 2012. Wanted by French authorities as of 2015. Review pursuant to Security Council resolution 2368 (2017) was concluded on 15 November 2021. INTERPOL-UN Security Council Special Notice web link: https://www.interpol.int/en/How-we-work/Notices/View-UN-Notices-Individuals click here

QDi.419 Name: 1: ANJEM 2: CHOUDARY 3: na 4: na
Title: na **Designation:** na **DOB:** 18 Jan. 1967 **POB:** Welling, London, United Kingdom of Great Britain and Northern Ireland **Good quality a.k.a.:** na **Low quality a.k.a.:** Abu Luqman **Nationality:** United Kingdom of Great Britain and Northern Ireland **Passport no:** United Kingdom of Great Britain and Northern Ireland number 516384722, issued on 6 May 2013 (issued by Passport Office Glasgow, expires 06 Jun. 2023) **National identification no:** na **Address:** London, United Kingdom of Great Britain and Northern Ireland **Listed on:** 15 Oct. 2018 (amended on 11 Oct. 2019) **Other information:** Pledged allegiance to Islamic State in Iraq and the Levant (ISIL), listed as Al-Qaida in Iraq (QDe.115) in July 2014 and subsequently released on licence in October 2018 which expires in July 2021. INTERPOL-UN Security Council Special Notice web link: https://www.interpol.int/en/How-we-work/Notices/View-UN-Notices-Individuals click here

QDi.300 Name: 1: MONIR 2: CHOUKA 3: na 4: na
Title: na **Designation:** na **DOB:** 30 Jul. 1981 **POB:** Bonn, Germany **Good quality a.k.a.:** na **Low quality a.k.a.:** Abu Adam **Nationality: a)** Germany **b)** Morocco **Passport no:** Germany number 5208323009, issued on 2 Feb. 2007, issued in Stadt Bonn, Germany (expires on 1 Feb. 2012) **National identification no:** Germany National Identification Number 5209530116, issued on 21 Jun. 2006, issued in Stadt Bonn, Germany (expired on 20 Jun. 2011) **Address:** Ungartenstraße 6, Bonn, 53229, Germany (previous) **Listed on:** 25 Jan. 2012 **Other information:** Associated with Islamic Movement of Uzbekistan (QDe.010). Brother of Yassin Chouka (QDi.301) Arrest warrant issued by the investigating judge of the German Federal Court of Justice on 5 Oct. 2010. Review pursuant to Security Council resolution 2368 (2017) was concluded on 15 November 2021. INTERPOL-UN Security Council Special Notice web link: https://www.interpol.int/en/How-we-work/Notices/View-UN-Notices-Individuals click here

QDi.301 Name: 1: YASSIN 2: CHOUKA 3: na 4: na
Title: na **Designation:** na **DOB:** 11 Dec. 1984 **POB:** Bonn, Germany **Good quality a.k.a.:** na **Low quality a.k.a.:** Abu Ibraheem **Nationality: a)** Germany **b)** Morocco **Passport no:** Germany number 5204893014, issued on 5 Oct. 2000, issued in Stadt Bonn, Germany (expired on 5 Oct. 2005) **National identification no:** Germany National Identification Number 5209445304, issued on 5 Sep. 2005, issued in Stadt Bonn, Germany (expired on 4 Sep. 2010) **Address:** Karl-Barth-Straße 14, Bonn, 53129, Germany (previous) **Listed on:** 25 Jan. 2012 **Other information:** Associated with Islamic Movement of Uzbekistan (QDe.010). Brother of Monir Chouka (QDi.300). Arrest warrant issued by the investigating judge of the German Federal Court of Justice on 5 Oct. 2010. Review pursuant to Security Council resolution 2368 (2017) was concluded on 15 November 2021. INTERPOL-UN Security Council Special Notice web link: https://www.interpol.int/en/How-we-work/Notices/View-UN-Notices-Individuals click here

QDi.141 Name: 1: MAXAMED 2: CABDULLAAH 3: CIISE 4: na
Title: na **Designation:** na **DOB:** 8 Oct. 1974 **POB:** Kismaayo, Somalia **Good quality a.k.a.: a)** Maxamed Cabdullaahi Ciise **b)** Maxammed Cabdullaahi **c)** Cabdullah Mayamed Ciise **Low quality a.k.a.:** na **Nationality:** Somalia **Passport no:** na **National identification no:** na **Address:** Somalia **Listed on:** 12 Nov. 2003 (amended on 9 Sep. 2005, 30 Jan. 2009, 20 Apr. 2009, 21 Oct. 2010, 24 Nov. 2020, 17 Aug. 2015) **Other information:** Present in Somalia as of Apr. 2009 following transfer from United Kingdom. Review pursuant to Security Council resolution 1822 (2008) was concluded on 21 Jun. 2010. Review pursuant to Security Council resolution 2368 (2017) was concluded on 24 November 2020. INTERPOL-UN Security Council Special Notice web link: https://www.interpol.int/en/How-we-work/Notices/View-UN-Notices-Individuals click here

QDi.410 Name: 1: SHANE 2: DOMINIC 3: CRAWFORD 4: na
Title: na **Designation:** na **DOB:** 22 Feb. 1986 **POB:** Mount Hope, Trinidad and Tobago **Good quality a.k.a.:** na **Low quality a.k.a.: a)** Asadullah **b)** Abu Sa’ d at-Trinidadi **c)** Asad **Nationality:** Trinidad and Tobago **Passport no: a)** Trinidad and Tobago number TA959547, issued on 19 Nov. 2013 (issued by Immigration Division of Trinidad and Tobago, expiration date 18 Nov. 2018) **b)** Trinidad and Tobago number T1071839, issued on 8 Nov. 2004 (issued by Immigration Division of Trinidad and Tobago, expiration date 7 Nov. 2014) **National identification no: a)** Trinidad and Tobago National Identification Card 19860222007, issued on 16 Jun. 2011 (expiration date 16 Jun. 2016) **b)** Trinidad and Tobago Birth Certificate B394445, issued on 23 Jan. 2007 **c)** Trinidad and Tobago Driver's Permit 892124B, issued on 30 Aug. 2007 (expiration date 30 Aug. 2010) **Address: a)** Syrian Arab Republic (as at May 2014) **b)** Reyhanli, Hatay, Turkey (previous location from Nov. 2013 to May 2014) **c)** 349 Dass Branch Trace, Dass Trace, Enterprise Chaguanas, Trinidad and Tobago (from birth until 27 Nov. 2013) **d)** LP# 41 Ballisier Road, Smith Field Lands, Wallerfield, County of St. George East, Trinidad and Tobago (alternative location as at Sep. 2011) **Listed on:** 18 Aug. 2017 **Other information:** English language propagandist for Islamic State in Iraq and the Levant (ISIL), listed as Al-Qaida in Iraq (AQI) (QDe.115). Wanted in Trinidad and Tobago for possession of ammunition and firearms and receiving stolen goods. Physical description: eye colour: brown; hair colour: dark; complexion: light brown; build: medium; height: 174cm; weight: 64kg; speaks English, Arabic. Review pursuant to Security Council resolution 2610 (2021) was concluded on 8 November 2022. INTERPOL-UN Security Council Special Notice web link: https://www.interpol.int/en/How-we-work/Notices/View-UN-Notices-Individuals click here

QDi.331 Name: 1: ANDERS 2: CAMEROON 3: OSTENSVIG 4: DALE
Title: na **Designation:** na **DOB:** 19 Oct. 1978 **POB:** Oslo, Norway **Good quality a.k.a.:** na **Low quality a.k.a.: a)** Muslim Abu Abdurrahman **b)** Abu Abdurrahman the Norwegian **c)** Abu Abdurrahman the Moroccan **Nationality:** Norway **Passport no:** na **National identification no:** na **Address:** na **Listed on:** 23 Sep. 2014 (amended on 6 Dec. 2019) **Other information:** Member of Al-Qaida in the Arabian Peninsula (AQAP) (QDe.129). Physical description: eye colour: brown; hair colour: brown; height: 185 cm. Review pursuant to Security Council resolution 2368 (2017) was concluded on 4 Dec. 2019 INTERPOL-UN Security Council Special Notice web link: https://www.interpol.int/en/How-we-work/Notices/View-UN-Notices-Individuals click here

QDi.245 Name: 1: PIO 2: ABOGNE 3: DE VERA 4: na
Title: na **Designation:** na **DOB:** 19 Dec. 1969 **POB:** Bagac, Bagamanok, Catanduanes, Philippines **Good quality a.k.a.:** Ismael De Vera **Low quality a.k.a.: a)** Khalid **b)** Ismael **c)** Ismail **d)** Manex **e)** Tito Art **f)** Dave **g)** Leo **Nationality:** Philippines **Passport no:** na **National identification no:** na **Address:** Concepcion, Zaragosa, Nueva Ecija, Philippines **Listed on:** 4 Jun. 2008 (amended on 3 Jun. 2009, 13 Dec. 2011, 6 Dec. 2019) **Other information:** Member of the Rajah Solaiman Movement (QDe.128), Abu Sayyaf Group (QDe.001) and Jemaah Islamiyah (QDe.092). Father's name is Honorio Devera. Mother's name is Fausta Abogne. In detention in the Philippines as of May 2011. Review pursuant to Security Council resolution 1822 (2008) was concluded on 13 May 2010. Review pursuant to Security Council resolution 2368 (2017) was concluded on 4 Dec. 2019. INTERPOL-UN Security Council Special Notice web link: https://www.interpol.int/en/How-we-work/Notices/View-UN-Notices-Individuals click here

QDi.252 Name: 1: AHMED 2: DEGHDEGH 3: na 4: na
Name (original script): أحمد دغداغ
Title: na **Designation:** na **DOB:** 17 Jan. 1967 **POB:** Anser, Wilaya (province) of Jijel, Algeria **Good quality a.k.a.: a)** Abd El Illah **b)** Abdellillah dit Abdellah Ahmed dit Said **Low quality a.k.a.:** na **Nationality:** Algeria **Passport no:** na **National identification no:** na **Address:** Algeria **Listed on:** 3 Jul. 2008 (amended on 24 Mar. 2009, 15 Nov. 2012) **Other information:** Belongs to the leadership and is the finance chief of the Organization of Al-Qaida in the Islamic Maghreb (QDe.014). Mother’ s name is Zakia Chebira. Father’ s name is Lakhdar. Review pursuant to

Security Council resolution 2368 (2017) was concluded on 15 November 2021. INTERPOL-UN Security Council Special Notice web link: https://www.interpol.int/en/How-we-work/Notices/View-UN-Notices-Individuals click here

QDi.246 Name: 1: REDENDO 2: CAIN 3: DELLOSA 4: na
Title: na **Designation:** na **DOB:** 15 May 1972 **POB:** Punta, Santa Ana, Manila, Philippines **Good quality a.k.a.: a)** Abu Ilonggo **b)** Brandon Berusa **c)** Abu Muadz **d)** Arnulfo Alvarado **e)** Habil Ahmad Dellosa **Low quality a.k.a.: a)** Dodong **b)** Troy **c)** Uthman **Nationality:** Philippines **Passport no:** na **National identification no:** na **Address:** 3111, Ma. Bautista, Punta, Santa Ana, Manila, Philippines **Listed on:** 4 Jun. 2008 (amended on 3 Jun. 2009, 13 Dec. 2011, 6 Dec. 2019) **Other information:** Member of the Rajah Solaiman Movement (QDe.128) and linked to the Abu Sayyaf Group (QDe.001). Father's name is Fernando Rafael Dellosa. Mother's name is Editha Parado Cain. In detention in the Philippines as of Jan. 2010. Review pursuant to Security Council resolution 1822 (2008) was concluded on 13 May 2010. Review pursuant to Security Council resolution 2368 (2017) was concluded on 4 Dec. 2019. INTERPOL-UN Security Council Special Notice web link: https://www.interpol.int/en/How-we-work/Notices/View-UN-Notices-Individuals click here

QDi.243 Name: 1: FELICIANO 2: SEMBORIO 3: DELOS REYES JR. 4: na
Name (original script): فلسيانو سمبوريو ديلوس رييس الإبن
Title: Ustadz **Designation:** na **DOB:** 4 Nov. 1963 **POB:** Arco, Lamitan, Basilan, Philippines **Good quality a.k.a.: a)** Abubakar Abdillah **b)** Abdul Abdillah **Low quality a.k.a.:** na **Nationality:** Philippines **Passport no:** na **National identification no:** na **Address:** Philippines **Listed on:** 4 Jun. 2008 (amended on 3 Jun. 2009, 13 Dec. 2011, 6 Dec. 2019) **Other information:** Member of the Rajah Solaiman Movement (QDe.128). Father's name is Feliciano Delos Reyes Sr. Mother's name is Aurea Semborio. In detention in the Philippines as of May 2011. Review pursuant to Security Council resolution 1822 (2008) was concluded on 13 May 2010. Review pursuant to Security Council resolution 2368 (2017) was concluded on 4 Dec. 2019. INTERPOL-UN Security Council Special Notice web link: https://www.interpol.int/en/How-we-work/Notices/View-UN-Notices-Individuals click here

QDi.342 Name: 1: OUMAR 2: DIABY 3: na 4: na
Title: na **Designation:** na **DOB:** 5 Aug. 1975 **POB:** Dakar, Senegal **Good quality a.k.a.:** na **Low quality a.k.a.: a)** Omsen **b)** Oumar Omsen **Nationality:** Senegal **Passport no:** na **National identification no:** na **Address:** Syrian Arab Republic (located in) **Listed on:** 23 Sep. 2014 (amended on 6 Dec. 2019) **Other information:** A leader of an armed group linked to Al-Nusrah Front for the People of the Levant (QDe.137) and a key facilitator for a Syrian foreign terrorist fighter network. Active in terrorist propaganda through the Internet. Review pursuant to Security Council resolution 2368 (2017) was concluded on 4 Dec. 2019 INTERPOL-UN Security Council Special Notice web link: https://www.interpol.int/en/How-we-work/Notices/View-UN-Notices-Individuals click here

QDi.167 Name: 1: KAMEL 2: DJERMANE 3: na 4: na
Name (original script): كمال جرمان
Title: na **Designation:** na **DOB:** 12 Oct. 1965 **POB:** Oum el Bouaghi, Algeria **Good quality a.k.a.: a)** Bilal **b)** Adel **c)** Fodhil **d)** Abou Abdeljalil **Low quality a.k.a.:** na **Nationality:** Algeria **Passport no:** na **National identification no:** na **Address:** Algeria **Listed on:** 3 May 2004 (amended on 7 Apr. 2008, 13 Dec. 2011, 6 Dec. 2019) **Other information:** In detention in Algeria as at April 2010. Arrest warrant issued by the German authorities on 9 Oct. 2003 for involvement in kidnapping. Former member of the Katibat Tarek Ibn Ziad of The Organization of Al-Qaida in the Islamic Maghreb (QDe.014). Review pursuant to Security Council resolution 1822 (2008) was concluded on 27 Jul. 2010. Review pursuant to Security Council resolution 2368 (2017) was concluded on 4 Dec. 2019. INTERPOL-UN Security Council Special Notice web link: https://www.interpol.int/en/How-we-work/Notices/View-UN-Notices-Individuals click here

QDi.249 Name: 1: YAHIA 2: DJOUADI 3: na 4: na

Name (original script): يحيى جوادي
Title: na **Designation:** na **DOB:** 1 Jan. 1967 **POB:** M' Hamid, Wilaya (province) of Sidi Bel Abbes, Algeria **Good quality a.k.a.: a)** Yahia Abou Ammar **b)** Abou Ala **Low quality a.k.a.:** na **Nationality:** Algeria **Passport no:** na **National identification no:** na **Address:** na **Listed on:** 3 Jul. 2008 (amended on 15 Nov. 2012, 2 Feb. 2023) **Other information:** Reportedly deceased as of February 2022. Belonged to the leadership of the Organization of Al-Qaida in the Islamic Maghreb (listed under permanent reference number QDe.014). Located in Northern Mali as of Jun. 2008. Mother' s name is Zohra Fares. Father' s name is Mohamed. Review pursuant to Security Council resolution 2368 (2017) was concluded on 15 November 2021. INTERPOL-UN Security Council Special Notice web link: https://www.interpol.int/en/How-we-work/Notices/View-UN-Notices-Individuals click here

QDi.232 Name: 1: ABDELMALEK 2: DROUKDEL 3: na 4: na
Name (original script): عبد المالك دروكدال
Title: na **Designation:** na **DOB:** 20 Apr. 1970 **POB:** Meftah, Wilaya of Blida, Algeria **Good quality a.k.a.:** Abou Mossaab Abdelouadoud **Low quality a.k.a.:** na **Nationality:** Algeria **Passport no:** na **National identification no:** na **Address:** Algeria **Listed on:** 27 Aug. 2007 (amended on 7 Apr. 2008, 13 Dec. 2011, 1 May 2019, 2 Feb. 2023) **Other information:** Reportedly deceased in June 2020. Head of The Organization of Al-Qaida in the Islamic Maghreb (QDe.014). Sentenced in absentia to life imprisonment in Algeria on 21 March 2007. Father's name is Rabah Droukdel. Mother's name is Z'hour Zdigha. Review pursuant to Security Council resolution 1822 (2008) was concluded on 4 May 2009. Review pursuant to Security Council resolution 2253 (2015) was concluded on 21 Feb. 2019. INTERPOL-UN Security Council Special Notice web link: https://www.interpol.int/en/How-we-work/Notices/View-UN-Notices-Individuals click here

QDi.095 Name: 1: LIONEL 2: DUMONT 3: na 4: na
Title: na **Designation:** na **DOB:** 29 Jan. 1971 **POB:** Roubaix, France **Good quality a.k.a.: a)** Jacques Brougere **b)** Abu Hamza **c)** Di Karlo Antonio **d)** Merlin Oliver Christian Rene **e)** Arfauni Imad Ben Yousset Hamza **f)** Imam Ben Yussuf Arfaj **g)** Abou Hamza **h)** Arfauni Imad **Low quality a.k.a.: a)** Bilal **b)** Hamza **c)** Koumkal **d)** Kumkal **e)** Merlin **f)** Tinet **g)** Brugere **h)** Dimon **Nationality:** France **Passport no:** na **National identification no:** na **Address:** France **Listed on:** 25 Jun. 2003 (amended on 22 Nov. 2004, 26 Nov. 2004, 2 Jul. 2007, 17 Oct. 2007, 24 Mar. 2009, 6 Aug. 2010, 24 Jun. 2016) **Other information:** In custody in France as of May 2004. Sentenced to 25 years imprisonment in France in 2007. His sentence is due to end on 13 Jul. 2023 and his unconditional detention to end on 13 Aug. 2020. Review pursuant to Security Council resolution 1822 (2008) was concluded on 15 Jun. 2010. INTERPOL-UN Security Council Special Notice web link: https://www.interpol.int/en/How-we-work/Notices/View-UN-Notices-Individuals click here

QDi.111 Name: 1: AGUS 2: DWIKARNA 3: na 4: na
Title: na **Designation:** na **DOB:** 11 Aug. 1964 **POB:** Makassar, South Sulawesi, Indonesia **Good quality a.k.a.:** na **Low quality a.k.a.:** na **Nationality:** Indonesia **Passport no:** Indonesia travel document number XD253038 **National identification no:** na **Address:** Indonesia **Listed on:** 9 Sep. 2003 (amended on 26 Nov. 2004, 14 May 2014, 6 Dec. 2019) **Other information:** Arrested 13 Mar. 2002, sentenced 12 July 2002 in the Philippines. Released from custody in the Philippines on 1 Jan. 2014 and subsequently deported to Indonesia. Physical description: height 165 cm. Photo available for inclusion in the INTERPOL-UN Security Council Special Notice. Review pursuant to Security Council resolution 1822 (2008) was concluded on 25 May 2010. Review pursuant to Security Council resolution 2368 (2017) was concluded on 4 Dec. 2019. INTERPOL-UN Security Council Special Notice web link: https://www.interpol.int/en/How-we-work/Notices/View-UN-Notices-Individuals click here

QDi.019 Name: 1: ABDULLAH 2: AHMED 3: ABDULLAH 4: EL ALFI
Name (original script): عبد الله احمد عبدالله الالفي
Title: na **Designation:** na **DOB:** 6 Jun. 1963 **POB:** Gharbia, Egypt **Good quality a.k.a.:** na **Low quality a.k.a.: a)** Abu Mariam **b)** Al-Masri, Abu Mohamed **c)** Saleh **Nationality:** Egypt **Passport no:**

na **National identification no:** na **Address:** na **Listed on:** 17 Oct. 2001 (amended on 26 Nov. 2004, 6 Dec. 2019) **Other information:** Afghanistan. Review pursuant to Security Council resolution 1822 (2008) was concluded on 21 Jun. 2010. Review pursuant to Security Council resolution 2368 (2017) was concluded on 4 Dec. 2019. Review pursuant to Security Council resolution 2610 (2021) was concluded on 8 November 2022. INTERPOL-UN Security Council Special Notice web link: https://www.interpol.int/en/How-we-work/Notices/View-UN-Notices-Individuals click here

QDi.142 Name: 1: RADI 2: ABD EL SAMIE 3: ABOU EL YAZID 4: EL AYASHI
Name (original script): راضي عبد السميع أبواليزيد العياشي
Title: na **Designation:** na **DOB:** 2 Jan. 1972 **POB:** El Gharbia Governorate, Egypt **Good quality a.k.a.:** na **Low quality a.k.a.:** Mera' i **Nationality:** Egypt **Passport no:** na **National identification no:** na **Address:** Via Cilea 40, Milan, Italy (Domicile) **Listed on:** 12 Nov. 2003 (amended on 9 Sep. 2005, 21 Dec. 2007, 16 May 2011, 29 Mar. 2017, 1 May 2019) **Other information:** Sentenced to ten years of imprisonment by the Court of first instance of Milan on 21 Sep. 2006. In custody in Italy. Due for release on 6 Jan. 2012. Subject to expulsion from Italy after serving the sentence. Review pursuant to Security Council resolution 1822 (2008) was concluded on 21 Jun. 2010. Review pursuant to Security Council resolution 2253 (2015) was concluded on 21 Feb. 2019. Review pursuant to Security Council resolution 2610 (2021) was concluded on 8 November 2022. INTERPOL-UN Security Council Special Notice web link: https://www.interpol.int/en/How-we-work/Notices/View-UN-Notices-Individuals click here

QDi.262 Name: 1: REDOUANE 2: EL HABHAB 3: na 4: na
Title: na **Designation:** na **DOB:** 20 Dec. 1969 **POB:** Casablanca, Morocco **Good quality a.k.a.:** Abdelrahman **Low quality a.k.a.:** na **Nationality: a)** Germany **b)** Morocco **Passport no:** German number 1005552350, issued on 27 Mar. 2001, issued in Municipality of Kiel, Germany (expired on 26 Mar. 2011) **National identification no:** Germany federal identity card number 1007850441, issued on 27 Mar. 2001, issued in Municipality of Kiel, Germany (expired on 26 Mar. 2011) **Address:** lltisstrasse 58, 24143 Kiel, Germany (previous address) **Listed on:** 12 Nov. 2008 (amended on 30 Jan. 2009, 24 Mar. 2009, 15 Nov. 2012) **Other information:** Released from custody in Germany in Apr. 2012. Review pursuant to Security Council resolution 2368 (2017) was concluded on 15 November 2021. INTERPOL-UN Security Council Special Notice web link: https://www.interpol.int/en/How-we-work/Notices/View-UN-Notices-Individuals click here

QDi.082 Name: 1: MOUNIR 2: EL MOTASSADEQ 3: na 4: na
Name (original script): منير المتصدق
Title: na **Designation:** na **DOB:** 3 Apr. 1974 **POB:** Marrakesh, Morocco **Good quality a.k.a.:** Mounir el Moutassadeq **Low quality a.k.a.:** na **Nationality:** Morocco **Passport no:** Morocco number H 236483 **National identification no:** Morocco E-491591 **Address:** (In prison in Germany) **Listed on:** 30 Sep. 2002 (amended on 26 Nov. 2004, 7 Sep. 2007, 23 Dec. 2010, 6 Dec. 2019) **Other information:** Arrested on 28 Nov. 2001 and found guilty in Germany of being an accessory to murder and of membership in a terrorist organization and sentenced to 15 years of imprisonment on 8 Jan. 2007. Father's name is Brahim Brik. Mother's name is Habiba Abbes. Review pursuant to Security Council resolution 1822 (2008) was concluded on 20 May 2010. Review pursuant to Security Council resolution 2368 (2017) was concluded on 4 Dec. 2019 INTERPOL-UN Security Council Special Notice web link: https://www.interpol.int/en/How-we-work/Notices/View-UN-Notices-Individuals click here

QDi.065 Name: 1: ABD EL KADER 2: MAHMOUD 3: MOHAMED 4: EL SAYED
Name (original script): عبد القادر محمود محمد السيد
Title: na **Designation:** na **DOB:** 26 Dec. 1962 **POB:** Egypt **Good quality a.k.a.: a)** Es Sayed, Kader **b)** Abdel Khader Mahmoud Mohamed el Sayed **Low quality a.k.a.:** na **Nationality:** Egypt **Passport no:** na **National identification no:** na **Address:** na **Listed on:** 24 Apr. 2002 (amended on 26 Nov. 2004, 7 Jun. 2007, 16 May 2011, 1 May 2019) **Other information:** Italian Fiscal Code: SSYBLK62T26Z336L. Sentenced to 8 years imprisonment in Italy on 2 February 2004. Considered a fugitive from justice

by the Italian authorities. Review pursuant to Security Council resolution 1822 (2008) was concluded on 22 Apr. 2010. Review pursuant to Security Council resolution 2253 (2015) was concluded on 21 Feb. 2019. Review pursuant to Security Council resolution 2368 (2017) was concluded on 15 November 2021. INTERPOL-UN Security Council Special Notice web link: https://www.interpol.int/en/How-we-work/Notices/View-UN-Notices-Individuals click here

QDi.409 Name: 1: ELSHAFEE 2: EL SHEIKH 3: na 4: na
Title: na **Designation:** na **DOB:** 16 Jul. 1988 **POB:** London, United Kingdom of Great Britain and Northern Ireland **Good quality a.k.a.: a)** El Shafee Elsheikh **b)** Alshafee El-Sheikh **Low quality a.k.a.:** na **Nationality:** United Kingdom of Great Britain and Northern Ireland **Passport no:** United Kingdom of Great Britain and Northern Ireland number 801121547, issued on 16 Jun. 2009 (issued by UK Passport Office with expiry date of 16 Jun. 2019, cancelled in Dec. 2014) **National identification no:** na **Address:** United States of America **Listed on:** 20 Jul. 2017 (amended on 2 Feb. 2023) **Other information:** Foreign terrorist fighter with Islamic State in Iraq and the Levant (ISIL), listed as Al-Qaida in Iraq (QDe.115), in the Syrian Arab Republic. Sentenced to life imprisonment on 19 August 2022 in the United States of America, Federal Bureau of Prisons inmate number 11698-509. Physical description: eye colour: dark brown; hair colour: black; complexion: dark. Distinguishing marks: beard. Mother's name: Maha Elgizouli. INTERPOL-UN Security Council Special Notice web link: https://www.interpol.int/en/How-we-work/Notices/View-UN-Notices-Individuals click here

QDi.064 Name: 1: SAMI 2: BEN KHAMIS 3: BEN SALEH 4: ELSSEID
Name (original script): سامي بن خميس بن صالح الصيد
Title: na **Designation:** na **DOB:** 10 Feb. 1968 **POB:** Menzel Jemil, Bizerte, Tunisia **Good quality a.k.a.:** Omar El Mouhajer **Low quality a.k.a.:** Saber **Nationality:** Tunisia **Passport no:** Tunisia number K929139, issued on 14 Feb. 1995 (expired on 13 Feb. 2000) **National identification no: a)** 00319547, issued on 8 Dec. 1994 **b)** SSDSBN68B10Z352F (Italian Fiscal Code) **Address:** Ibn Al-Haythman Street, Number 6, Manubah, Tunis, Tunisia **Listed on:** 24 Apr. 2002 (amended on 10 Apr. 2003, 26 Nov. 2004, 9 Sep. 2005, 20 Dec. 2005, 7 Jun. 2007, 21 Dec. 2007, 10 Aug. 2009, 23 Dec. 2010, 24 Nov. 2014, 6 Dec. 2019) **Other information:** Mother's name is Beya Al-Saidani. Deported from Italy to Tunisia on 2 Jun. 2008. Imprisoned in Tunisia in Aug. 2014. Review pursuant to Security Council resolution 1822 (2008) was concluded on 22 Apr. 2010. Review pursuant to Security Council resolution 2368 (2017) was concluded on 4 Dec. 2019 INTERPOL-UN Security Council Special Notice web link: https://www.interpol.int/en/How-we-work/Notices/View-UN-Notices-Individuals click here

QDi.096 Name: 1: MOUSSA 2: BEN OMAR 3: BEN ALI 4: ESSAADI
Name (original script): موسى بن عمر بن علي السعدي
Title: na **Designation:** na **DOB:** 4 Dec. 1964 **POB:** Tabarka, Tunisia **Good quality a.k.a.:** na **Low quality a.k.a.: a)** Dah Dah **b)** Abdelrahmman **c)** Bechir **Nationality:** Tunisia **Passport no:** Tunisia number L335915, issued on 8 Nov. 1996, issued in Milan, Italy (expired on 7 Nov. 2001) **National identification no:** na **Address:** Tunisia **Listed on:** 25 Jun. 2003 (amended on 20 Dec. 2005, 17 Oct. 2007, 10 Aug. 2009, 16 May 2011, 20 Jul. 2012, 6 Dec. 2019, 10 Sep. 2020) **Other information:** Considered a fugitive from justice by the Italian authorities (as of Oct 2019). Left Sudan to Tunisia in 2011. Review pursuant to Security Council resolution 1822 (2008) was concluded on 15 Jun. 2010. Review pursuant to Security Council resolution 2368 (2017) was concluded on 4 Dec. 2019. INTERPOL-UN Security Council Special Notice web link: https://www.interpol.int/en/How-we-work/Notices/View-UN-Notices-Individuals click here

QDi.083 Name: 1: ZAKARYA 2: ESSABAR 3: na 4: na
Name (original script): زكريا الصبار
Title: na **Designation:** na **DOB:** 3 Apr. 1977 **POB:** Essaouria, Morocco **Good quality a.k.a.:** Zakariya Essabar **Low quality a.k.a.:** na **Nationality:** Morocco **Passport no: a)** Morocco number M 271351, issued on 24 Oct. 2000, issued in Berlin,by the Embassy of Morocco **b)** Morocco number K-

348486 **National identification no: a)** Morocco National Identity number E-189935 **b)** Morocco National Identity Card number G-0343089 **Address:** na **Listed on:** 30 Sep. 2002 (amended on 26 Nov. 2004, 10 Jun. 2011) **Other information:** Father's name is Mohamed ben Ahmed. Mother's name is Sfia bent Toubali. Review pursuant to Security Council resolution 1822 (2008) was concluded on 20 May 2010. Review pursuant to Security Council resolution 2368 (2017) was concluded on 15 November 2021. INTERPOL-UN Security Council Special Notice web link: https://www.interpol.int/en/How-we-work/Notices/View-UN-Notices-Individuals click here

QDi.352 Name: 1: MAULANA 2: FAZLULLAH 3: na 4: na
Title: na **Designation:** na **DOB:** 1974 **POB:** Kuza Bandai village, Swat Valley, Khyber Pakhtunkhawa Province, Pakistan **Good quality a.k.a.: a)** Mullah Fazlullah **b)** Fazal Hayat **Low quality a.k.a.:** Mullah Radio **Nationality:** na **Passport no:** na **National identification no:** na **Address:** (Afghanistan / Pakistan border region) **Listed on:** 7 Apr. 2015 (amended on 1 May 2019) **Other information:** Commander of Tehrik-e Taliban Pakistan (TTP) (QDe.132) since 7 Nov. 2013. Led the local TTP in Pakistan' s northwest valley of Swat from 2007 to 2009. Review pursuant to Security Council resolution 2253 (2015) was concluded on 21 Feb. 2019. INTERPOL-UN Security Council Special Notice web link: https://www.interpol.int/en/How-we-work/Notices/View-UN-Notices-Individuals click here

QDi.251 Name: 1: SALAH EDDINE 2: GASMI 3: na 4: na
Name (original script): صالح قاسمي
Title: na **Designation:** na **DOB:** 13 Apr. 1971 **POB:** Zeribet El Oued, Wilaya (province) of Biskra, Algeria **Good quality a.k.a.:** Abou Mohamed Salah **Low quality a.k.a.:** Bounouadher **Nationality:** Algeria **Passport no:** na **National identification no:** na **Address:** Algeria **Listed on:** 3 Jul. 2008 (amended on 24 Mar. 2009, 15 Nov. 2012, 14 Mar. 2013, 11 Feb. 2016, 24 Nov. 2020) **Other information:** Belongs to the leadership and is in charge of information committee of the Organization of Al-Qaida in the Islamic Maghreb (QDe.014). Mother' s name is Yamina Soltane. Father' s name is Abdelaziz. Associated with Abdelmalek Droukdel (QDi.232). Arrested in Algeria on 16 Dec. 2012. Incarcerated at the El-Harrach prison in Algiers, as of August 2015. Review pursuant to Security Council resolution 2368 (2017) was concluded on 24 November 2020. INTERPOL-UN Security Council Special Notice web link: https://www.interpol.int/en/How-we-work/Notices/View-UN-Notices-Individuals click here

QDi.366 Name: 1: TARKHAN 2: ISMAILOVICH 3: GAZIEV 4: na
Name (original script): Тархан Исмаилович Газиев
Title: na **Designation:** na **DOB:** 11 Nov. 1965 **POB:** Itum-Kale, Itum-Kalinskiy District, Republic of Chechnya, Russian Federation **Good quality a.k.a.: a)** Ramzan Oduev (original script: Рамзан Одуев) **b)** Tarkhan Isaevich Gaziev (original script: Тархан Исаевич Газиев) **c)** Husan Isaevich Gaziev (original script: Хусан Исаевич Газиев) **d)** Umar Sulimov (original script: Умар Сулимов) **Low quality a.k.a.: a)** Wainakh (original script: Вайнах) **b)** Sever (original script: Север) **c)** Abu Bilal (original script: Абу-Билал) **d)** Abu Yasir (original script: Абу Ясир) **e)** Abu Asim (original script: Абу Ясим) **f)** Husan (original script: Хусан) **g)** Ab-Bilal **h)** Abu-Naser **Nationality:** Russian Federation **Passport no:** na **National identification no:** na **Address: a)** Syrian Arab Republic (located in as at Aug. 2015) **b)** Iraq (possible alternative location as at Aug. 2015) **Listed on:** 2 Oct. 2015 (amended on 10 Dec. 2015, 6 Sep. 2016) **Other information:** As at Aug. 2015, leads Jamaat Tarkhan, a terrorist group that forms part of the Islamic State in Iraq and the Levant, listed as Al-Qaida in Iraq (QDe.115). Wanted by the authorities of the Russian Federation for terrorist crimes committed in its territory, including through an international arrest warrant. Photo available for inclusion in the INTERPOL-UN Security Council Special Notice. Review pursuant to Security Council resolution 2368 (2017) was concluded on 15 November 2021. INTERPOL-UN Security Council Special Notice web link: https://www.interpol.int/en/How-we-work/Notices/View-UN-Notices-Individuals click here

QDi.431 Name: 1: SANAULLAH 2: GHAFARI 3: na 4: na

Name (original script): ثناء الله غفارى
Title: Dr. **Designation:** na **DOB:** 28 Oct. 1994 **POB:** Afghanistan **Good quality a.k.a.: a)** Dr. Shahab al Muhajir **b)** Shahab Muhajer **c)** Shahab Mohajir **d)** Shahab Mahajar **e)** Shihab al Muhajir **f)** Shihab Muhajer **g)** Shihab Mohajir **h)** Shihab Mahajar **Low quality a.k.a.:** na **Nationality:** Afghanistan **Passport no:** na **National identification no:** na **Address: a)** Afghanistan (2021) **b)** Kunduz, Afghanistan (previous) **Listed on:** 21 Dec. 2021 **Other information:** Leader of the Islamic State of Iraq and the Levant - Khorasan (ISIL - K) (QDe.161). Information Technology Expert. Photo is available for inclusion in the INTERPOL-UN Security Council Special Notice. INTERPOL-UN Security Council Special Notice web link: https://www.interpol.int/en/How-we-work/Notices/View-UN-Notices-Individuals click here

QDi.028 Name: 1: AHMED 2: KHALFAN 3: GHAILANI 4: na
Title: na **Designation:** na **DOB: a)** 14 Mar. 1974 **b)** 13 Apr. 1974 **c)** 14 Apr. 1974 **d)** 1 Aug. 1970 **POB:** Zanzibar, United Republic of Tanzania **Good quality a.k.a.: a)** Ahmad, Abu Bakr **b)** Ahmed, Abubakar **c)** Ahmed, Abubakar K. **d)** Ahmed, Abubakar Khalfan **e)** Ahmed, Abubakary K. **f)** Ahmed, Ahmed Khalfan **g)** Ali, Ahmed Khalfan **h)** Ghailani, Abubakary Khalfan Ahmed **i)** Ghailani, Ahmed **j)** Ghilani, Ahmad Khalafan **k)** Hussein, Mahafudh Abubakar Ahmed Abdallah **l)** Khalfan, Ahmed **m)** Mohammed, Shariff Omar **n)** Haythem al-Kini **Low quality a.k.a.: a)** Ahmed The Tanzanian **b)** Foopie **c)** Fupi **d)** Ahmed, A **e)** Al Tanzani, Ahmad **f)** Bakr, Abu **g)** Khabar, Abu **Nationality:** United Republic of Tanzania **Passport no:** na **National identification no:** na **Address:** United States of America **Listed on:** 17 Oct. 2001 (amended on 27 Jul. 2007, 21 Oct. 2010, 6 Dec. 2019) **Other information:** Apprehended in July 2004 and in custody for trial in the United States of America, as at October 2010. Review pursuant to Security Council resolution 1822 (2008) was concluded on 21 Jun. 2010. Review pursuant to Security Council resolution 2368 (2017) was concluded on 4 Dec. 2019. INTERPOL-UN Security Council Special Notice web link: https://www.interpol.int/en/How-we-work/Notices/View-UN-Notices-Individuals click here

QDi.250 Name: 1: AMOR 2: MOHAMED 3: GHEDEIR 4: na
Name (original script): عمر محمد قدير
Title: na **Designation:** na **DOB:** Approximately 1958 **POB:** Deb-Deb, Amenas, Wilaya (province) of Illizi, Algeria **Good quality a.k.a.: a)** Abdelhamid Abou Zeid **b)** Youcef Adel **c)** Abou Abdellah **d)** Abid Hammadou born 12 Dec. 1965 in Algeria (previously listed as) **Low quality a.k.a.:** na **Nationality:** Algeria **Passport no:** na **National identification no:** na **Address:** na **Listed on:** 3 Jul. 2008 (amended on 10 May 2012, 15 Nov. 2012, 29 Mar. 2019, 1 May 2019) **Other information:** Associated with the Organization of Al-Qaida in the Islamic Maghreb (QDe.014). Located in Northern Mali as of Jun. 2008. Mother's name is Benarouba Bachira. Father's name is Mabrouk. He usurped the identity of Abid Hammadou, who allegedly died in Chad in 2004. Reportedly deceased as of 24 February 2013. Review pursuant to Security Council resolution 2253 (2015) was concluded on 21 Feb. 2019. Review pursuant to Security Council resolution 2368 (2017) was concluded on 15 November 2021. INTERPOL-UN Security Council Special Notice web link: https://www.interpol.int/en/How-we-work/Notices/View-UN-Notices-Individuals click here

QDi.367 Name: 1: ZAURBEK 2: SALIMOVICH 3: GUCHAEV 4: na
Name (original script): Заурбек Салимович Гучаев
Title: na **Designation:** na **DOB:** 7 Sep. 1975 **POB:** Chegem-1 Village, Chegemskiy District, Republic of Kabardino-Balkaria, Russian Federation **Good quality a.k.a.:** na **Low quality a.k.a.: a)** Bach (original script: Бэч) **b)** Fackih (original script: Факих) **c)** Vostochniy (original script: Восточный) **d)** Muslim (original script: Муслим) **e)** Aziz (original script: Азиз) **f)** Abdul Aziz (original script: Абул Азиз) **Nationality:** Russian Federation **Passport no:** Russian foreign travel passport number 622641887 **National identification no:** Russian Federation national passport 8304661431 **Address: a)** Syrian Arab Republic (located in as at Aug. 2015) **b)** Iraq (possible alternative location as at Aug. 2015) **Listed on:** 2 Oct. 2015 (amended on 24 Nov. 2020) **Other information:** As at Aug. 2015, one of the leaders of the Army of Emigrants and Supporters (QDe.148). Wanted by the authorities of the Russian Federation for terrorist crimes committed in its territory. Photo available for inclusion

in the INTERPOL-UN Security Council Special Notice. Review pursuant to Security Council resolution 2368 (2017) was concluded on 24 November 2020. INTERPOL-UN Security Council Special Notice web link: https://www.interpol.int/en/How-we-work/Notices/View-UN-Notices-Individuals click here

QDi.341 Name: 1: KEVIN 2: JORDAN 3: AXEL 4: GUIAVARCH
Title: na **Designation:** na **DOB:** 12 Mar. 1993 **POB:** Paris, France **Good quality a.k.a.:** na **Low quality a.k.a.:** na **Nationality:** France **Passport no:** France number 12CP63882.3FRA, issued on 31 Jul. 2012 (valid until 30 Jul. 2022) **National identification no:** France national identity card 070275Q007873, issued on 16 Feb. 2007 (valid until 15 Feb. 2017) **Address: a)** Grenoble, France (domicile from 1993 to 2012) **b)** Syrian Arab Republic (located in between 2012 and 2016) **c)** Turkey (from Jun. 2016 to Jan. 2017) **d)** France (in detention since Jan. 2017) **Listed on:** 23 Sep. 2014 (amended on 24 Jun. 2016, 27 Aug. 2017) **Other information:** French terrorist fighter associated with Al-Nusrah Front for the People of the Levant (QDe.137) and the Islamic State in Iraq and the Levant (ISIL), listed as Al-Qaida in Iraq (QDe.115). Facilitated foreign terrorist fighters travel from France to Syria. Activist in violent propaganda through the Internet. A warrant for his arrest was issued in 2014 by French authorities and executed in Jan. 2017 upon his expulsion from Turkey where he was arrested in Jun. 2016. Review pursuant to Security Council resolution 2368 (2017) was concluded on 15 November 2021. INTERPOL-UN Security Council Special Notice web link: https://www.interpol.int/en/How-we-work/Notices/View-UN-Notices-Individuals click here

QDi.218 Name: 1: GUN GUN 2: RUSMAN 3: GUNAWAN 4: na
Title: na **Designation:** na **DOB:** 6 Jul. 1977 **POB:** Cianjur, West Java, Indonesia **Good quality a.k.a.: a)** Gunawan, Rusman **b)** Abd Al-Hadi **c)** Abdul Hadi **d)** Abdul Karim **e)** Bukhori **f)** Bukhory **Low quality a.k.a.:** na **Nationality:** Indonesia **Passport no:** na **National identification no:** na **Address:** na **Listed on:** 21 Apr. 2006 (amended on 13 Dec. 2011, 6 Dec. 2019) **Other information:** Brother of Nurjaman Riduan Isamuddin (QDi.087). Review pursuant to Security Council resolution 1822 (2008) was concluded on 8 Jun. 2010. Review pursuant to Security Council resolution 2368 (2017) was concluded on 4 Dec. 2019. Review pursuant to Security Council resolution 2610 (2021) was concluded on 8 November 2022. INTERPOL-UN Security Council Special Notice web link: https://www.interpol.int/en/How-we-work/Notices/View-UN-Notices-Individuals click here

QDi.003 Name: 1: SALIM 2: AHMAD 3: SALIM 4: HAMDAN
Name (original script): سالم أحمد سالم حمدان
Title: na **Designation:** na **DOB:** 1965 **POB: a)** Al-Mukalla, Yemen **b)** Al-Mukala, Yemen **Good quality a.k.a.: a)** Saqr Al-Jaddawi (الكنية صقر الجداوي) **b)** Saqar Al Jadawi **c)** Saqar Aljawadi **d)** Salem Ahmed Salem Hamdan **Low quality a.k.a.:** na **Nationality:** Yemen **Passport no:** Yemen number 00385937 **National identification no:** na **Address: a)** Shari Tunis, Sana'a, Yemen (previous address) **b)** (Located in Yemen since Nov. 2008) **Listed on:** 25 Jan. 2001 (amended on 25 Jul. 2006, 23 Apr. 2007, 30 Jan. 2009, 17 Jul. 2009, 25 Jan. 2010, 6 Dec. 2019) **Other information:** Driver and private bodyguard to Usama bin Laden (deceased) from 1996 until 2001. Transferred from United States custody to Yemen in Nov. 2008. Review pursuant to Security Council resolution 1822 (2008) was concluded on 8 Jul. 2010. Review pursuant to Security Council resolution 2368 (2017) was concluded on 4 Dec. 2019 INTERPOL-UN Security Council Special Notice web link: https://www.interpol.int/en/How-we-work/Notices/View-UN-Notices-Individuals click here

QDi.068 Name: 1: ADEL 2: BEN AL-AZHAR 3: BEN YOUSSEF 4: HAMDI
Name (original script): عادل بن الأزهر بن يوسف حمدي
Title: na **Designation:** na **DOB:** 14 Jul. 1970 **POB:** Tunis, Tunisia **Good quality a.k.a.:** Adel ben al-Azhar ben Youssef ben Soltane born 14 Jul. 1970 in Tunis, Tunisia (formerly listed as) **Low quality a.k.a.:** Zakariya **Nationality:** Tunisia **Passport no:** Tunisia number M408665, issued on 4 Oct. 2000 (expired 3 Oct. 2005) **National identification no: a)** (Tunisian national identity number W334061 issued on 9 Mar. 2011) **b)** Italian Fiscal Code: BNSDLA70L14Z352B **Address:** Tunisia **Listed on:** 3 Sep. 2002 (amended on 20 Dec. 2005, 7 Jun. 2007, 23 Dec. 2010, 24 Nov. 2014, 6 Dec. 2019) **Other information:** Deported from Italy to Tunisia on 28 February 2004. Serving a 12-year prison sentence

in Tunisia for membership in a terrorist organization abroad as at Jan. 2010. Arrested in Tunisia in 2013. Legally changed family name from Ben Soltane to Hamdi in 2014. Review pursuant to Security Council resolution 1822 (2008) was concluded on 21 Jun. 2010. Review pursuant to Security Council resolution 2368 (2017) was concluded on 4 Dec. 2019. INTERPOL-UN Security Council Special Notice web link: https://www.interpol.int/en/How-we-work/Notices/View-UN-Notices-Individuals click here

QDi.204 Name: 1: ISNILON 2: TOTONI 3: HAPILON 4: na
Title: na **Designation:** na **DOB: a)** 18 Mar. 1966 **b)** 10 Mar. 1967 **POB:** Bulanza, Lantawan, Basilan, Philippines **Good quality a.k.a.: a)** Isnilon Hapilun **b)** Isnilun Hapilun **c)** Tuan Isnilon **Low quality a.k.a.: a)** Abu Musab **b)** Salahudin **Nationality:** Philippines **Passport no:** na **National identification no:** na **Address: a)** Basilan, Philippines (previous location until 2016) **b)** Lanao del Sur, Philippines (location since 2016) **Listed on:** 6 Dec. 2005 (amended on 4 Oct. 2017, 6 Dec. 2019, 2 Feb. 2023) **Other information:** Senior leader of Abu Sayyaf Group (ASG) (QDe.001). Leader of local affiliates of the Islamic State in Iraq and the Levant (ISIL), listed as Al-Qaida in Iraq (AQI) (QDe.115), in the southern Philippines as of May 2017. Reportedly deceased in 2017. Physical description: eye colour: brown; hair colour: brown; height: 5 feet 6 inches – 168 cm; weight: 120 pounds – 54 kg; build: slim; complexion: light-skinned; has facial birthmarks. Review pursuant to Security Council resolution 1822 (2008) was concluded on 8 Jun. 2010. Wanted by the Philippines authorities for terrorist offences and by authorities of the United States of America for involvement in terrorist acts. Review pursuant to Security Council resolution 2368 (2017) was concluded on 4 Dec. 2019. Photos included in INTERPOL-UN Security Council Special Notice web link: https://www.interpol.int/en/How-we-work/Notices/View-UN-Notices-Individuals click here

QDi.268 Name: 1: ABDUL 2: HAQ 3: na 4: na
Name (original script): 阿不都·哈克
Title: na **Designation:** na **DOB:** 10 Oct. 1971 **POB:** Hetian Area, Xinjiang Uighur Autonomous Region, China **Good quality a.k.a.: a)** Maimaitiming Maimaiti **b)** Abdul Heq **c)** Abudu Hake **d)** Abdul Heq Jundullah **e)** 'Abd Al-Haq **f)** Memetiming Memeti **g)** Memetiming Aximu **h)** Memetiming Qekeman **i)** Maiumaitimin Maimaiti **j)** Abdul Saimaiti **k)** Muhammad Ahmed Khaliq **Low quality a.k.a.: a)** Maimaiti Iman **b)** Muhelisi **c)** Qerman **d)** Saifuding **Nationality:** China **Passport no:** na **National identification no:** China 653225197110100533 (Chinese national identity card number) **Address: a)** Afghanistan (current location as at Jul. 2016) **b)** Pakistan (previous location as at Apr. 2009) **Listed on:** 15 Apr. 2009 (amended on 13 Dec. 2011, 20 Jun. 2017, 24 Nov. 2020) **Other information:** Overall leader and commander of the Eastern Turkistan Islamic Movement (QDe.088). Involved in fundraising and recruitment for this organization. Review pursuant to Security Council resolution 2368 (2017) was concluded on 24 November 2020. INTERPOL-UN Security Council Special Notice web link: https://www.interpol.int/en/How-we-work/Notices/View-UN-Notices-Individuals click here

QDi.378 Name: 1: MAXIME 2: HAUCHARD 3: na 4: na
Title: na **Designation:** na **DOB:** 17 Mar. 1992 **POB:** Saint Aubin les Elbeuf, Normandy, France **Good quality a.k.a.:** na **Low quality a.k.a.:** Abou Abdallah al Faransi **Nationality:** France **Passport no:** na **National identification no:** French national identity card number 101127200129 (issued by the Sous-Préfecture of Bernay, France and expires 4 Nov. 2020) **Address:** Syrian Arab Republic (as at Sep. 2015) **Listed on:** 29 Sep. 2015 (amended on 24 Jun. 2016) **Other information:** French foreign terrorist fighter for Islamic State in Iraq and the Levant, listed as Al-Qaida in Iraq (QDe.115). French arrest warrant issued on 20 Jan. 2015 by a magistrate of the anti-terrorism division of the Prosecutor's Office in Paris for murder in connection with a terrorist entity and participation in a terrorist criminal association. Review pursuant to Security Council resolution 2368 (2017) was concluded on 15 November 2021. INTERPOL-UN Security Council Special Notice web link: https://www.interpol.int/en/How-we-work/Notices/View-UN-Notices-Individuals click here

QDi.029 Name: 1: RAED 2: MUHAMMAD HASAN 3: MUHAMMAD 4: HIJAZI

Name (original script): رائد محمد حسن محمد حجازي
Title: na **Designation:** na **DOB:** 30 Dec. 1968 **POB:** California, United States of America **Good quality a.k.a.: a)** Raed M. Hijazi **b)** Ri' ad Muhammad Hasan Muhammad Hijazi (Previously listed as. In Arabic: رياض محمد حسن محمد الحجازي) **Low quality a.k.a.: a)** Rashid Al-Maghribi (The Moroccan) **b)** Abu-Ahmad Al-Amriki (The American) **c)** Abu-Ahmad Al-Hawen **d)** Abu-Ahmad Al-Shahid **Nationality: a)** Jordan **b)** United States of America **Passport no:** na **National identification no: a)** United States Social Security Number: 548-91-5411 **b)** Jordanian national number: 9681029476 **Address:** na **Listed on:** 17 Oct. 2001 (amended on 10 Apr. 2003, 16 May 2011, 21 Mar. 2017, 24 Nov. 2020) **Other information:** In custody in Jordan since 26 Feb. 2015 for recruitment and support to Islamic State in Iraq and the Levant (ISIL), listed as Al-Qaida in Iraq (QDe.115). Father' s name is Mohammad Hijazi. Mother' s name is Sakina. Review pursuant to Security Council resolution 1822 (2008) was concluded on 21 Jun. 2010. Review pursuant to Security Council resolution 2368 (2017) was concluded on 24 November 2020. INTERPOL-UN Security Council Special Notice web link: https://www.interpol.int/en/How-we-work/Notices/View-UN-Notices-Individuals click here

QDi.359 Name: 1: OMAR 2: ALI 3: HUSSAIN 4: na
Title: na **Designation:** na **DOB:** 21 Mar. 1987 **POB:** High Wycombe, Buckinghamshire, United Kingdom of Great Britain and Northern Ireland **Good quality a.k.a.:** na **Low quality a.k.a.:** Abu-Sa'id Al Britani **Nationality:** United Kingdom of Great Britain and Northern Ireland **Passport no:** United Kingdom of Great Britain and Northern Ireland number 205939411, issued on 21 Jul. 2004 (expired on 21 Apr. 2015) **National identification no:** na **Address: a)** Syrian Arab Republic (as at Jan. 2014) **b)** United Kingdom of Great Britain and Northern Ireland (previous address until Jan. 2014) **Listed on:** 28 Sep. 2015 (amended on 24 Nov. 2020) **Other information:** Foreign terrorist fighter with Islamic State in Iraq and the Levant (ISIL), listed as Al-Qaida in Iraq (QDe.115), in the Syrian Arab Republic. Physical description: eye colour: brown; hair colour: brown/black. Photo available for inclusion in the INTERPOL-UN Security Council Special Notice. Review pursuant to Security Council resolution 2368 (2017) was concluded on 24 November 2020. INTERPOL-UN Security Council Special Notice web link: https://www.interpol.int/en/How-we-work/Notices/View-UN-Notices-Individuals click here

QDi.144 Name: 1: MOHAMMAD 2: TAHIR 3: HAMMID 4: HUSSEIN
Name (original script): محمد طاهر حامد حسين
Title: Imam **Designation:** na **DOB:** 1 Nov. 1975 **POB:** Poshok, Iraq **Good quality a.k.a.:** Abdelhamid Al Kurdi **Low quality a.k.a.:** na **Nationality:** Iraq **Passport no:** na **National identification no:** na **Address:** Sulaymaniya, Iraq **Listed on:** 12 Nov. 2003 (amended on 9 Sep. 2005, 21 Dec. 2007, 16 May 2011, 10 Dec. 2015, 29 Mar. 2019, 1 May 2019) **Other information:** Mother's name: Attia Mohiuddin Taha. A deportation order was issued by the Italian authorities on 18 Oct. 2004. Considered a fugitive from justice by the Italian authorities as of Sep. 2007. Review pursuant to Security Council resolution 1822 (2008) was concluded on 8 Jun. 2010. Review pursuant to Security Council resolution 2253 (2015) was concluded on 21 February 2019. Review pursuant to Security Council resolution 2610 (2021) was concluded on 8 November 2022. INTERPOL-UN Security Council Special Notice web link: https://www.interpol.int/en/How-we-work/Notices/View-UN-Notices-Individuals click here

QDi.067 Name: 1: MOSTAFA 2: KAMEL 3: MOSTAFA 4: IBRAHIM
Name (original script): مصطفى كمال مصطفى ابراهيم
Title: na **Designation:** na **DOB:** 15 Apr. 1958 **POB:** Alexandria, Egypt **Good quality a.k.a.: a)** Mustafa Kamel Mustafa **b)** Adam Ramsey Eaman **c)** Kamel Mustapha Mustapha **d)** Mustapha Kamel Mustapha **e)** Mostafa Kamel Mostafa **Low quality a.k.a.: a)** Abu Hamza Al-Masri **b)** Abu Hamza **c)** Abu Hamza Al-Misri **Nationality:** United Kingdom of Great Britain and Northern Ireland **Passport no:** na **National identification no:** na **Address:** United States of America **Listed on:** 24 Apr. 2002 (amended on 26 Nov. 2004, 25 Jul. 2006, 14 Mar. 2008, 21 Oct. 2010, 4 Aug. 2014, 6 Dec. 2019, 18 Mar. 2020) **Other information:** Extradited from the United Kingdom to the United States of

America on 5 Oct. 2012. Convicted on terrorism charges by a court in the United States of America in May 2014. Review pursuant to Security Council resolution 1822 (2008) was concluded on 22 Apr. 2010. Review pursuant to Security Council resolution 2368 (2017) was concluded on 4 Dec. 2019. INTERPOL-UN Security Council Special Notice web link: https://www.interpol.int/en/How-we-work/Notices/View-UN-Notices-Individuals click here

QDi.395 Name: 1: MUHAMMAD 2: SHOLEH 3: IBRAHIM 4: na
Title: Ustad **Designation:** na **DOB:** 1958 (Sep.) **POB:** Demak, Indonesia **Good quality a.k.a.: a)** Mohammad Sholeh Ibrahim **b)** Muhammad Sholeh Ibrohim **c)** Muhammad Soleh Ibrahim **d)** Sholeh Ibrahim **e)** Muh Sholeh Ibrahim **Low quality a.k.a.:** na **Nationality:** Indonesia **Passport no:** na **National identification no: a)** Indonesia 3311092409580002 **b)** Indonesia 3311092409580003 **Address: a)** Masjid Baitul Amin, Waringinrejo RT 01 RW 02, Grogol, Cemani, Sukoharjo, Jawa Tengah 57572, Indonesia **b)** Desa Cemani, Waringinrejo RT 001/021, Kecamatan Grogol, Kabupaten Sukoharjo, Jawa Tengah, Indonesia **Listed on:** 20 Apr. 2016 (amended on 27 May 2022) **Other information:** Has served as the acting emir of Jemmah Anshorut Tauhid (JAT) (QDe.133) since 2014 and has supported Islamic State in Iraq and the Levant (ISIL), listed as Al-Qaida in Iraq (QDe.115). Profession: Lecturer/Private Teacher. Review pursuant to Security Council resolution 2368 (2017) was concluded on 15 November 2021. INTERPOL-UN Security Council Special Notice web link: https://www.interpol.int/en/How-we-work/Notices/View-UN-Notices-Individuals click here

QDi.374 Name: 1: NUSRET 2: IMAMOVIC 3: na 4: na
Title: na **Designation:** na **DOB: a)** 26 Sep. 1971 **b)** 26 Sep. 1977 **POB:** Miljanovci, Kalesija Municipality, Bosnia and Herzegovina **Good quality a.k.a.:** Nusret Sulejman Imamovic **Low quality a.k.a.:** na **Nationality:** Bosnia and Herzegovina **Passport no: a)** Bosnia and Herzegovina number 349054 **b)** Bosnia and Herzegovina number 3490054 **National identification no:** na **Address:** Syrian Arab Republic (location as at Sep. 2015) **Listed on:** 29 Feb. 2016 (amended on 29 Mar. 2019, 1 May 2019) **Other information:** Believed to be fighting with Al-Nusrah Front for the People of the Levant (QDe.137) in Syrian Arab Republic and reported to be a leader in the group as of Apr. 2015. Review pursuant to Security Council resolution 2253 (2015) was concluded on 21 February 2019. INTERPOL-UN Security Council Special Notice web link: https://www.interpol.int/en/How-we-work/Notices/View-UN-Notices-Individuals click here

QDi.308 Name: 1: ZAFAR 2: IQBAL 3: na 4: na
Title: na **Designation:** na **DOB:** 4 Oct. 1953 **POB:** na **Good quality a.k.a.: a)** Zaffer Iqbal **b)** Malik Zafar Iqbal Shehbaz **c)** Malik Zafar Iqbal Shahbaz **d)** Malik Zafar Iqbal **Low quality a.k.a.: a)** Zafar Iqbal Chaudhry **b)** Muhammad Zafar Iqbal **Nationality:** Pakistan **Passport no:** DG5149481, issued on 22 Aug. 2006 (expired on 21 Aug. 2011, passport booklet number A2815665) **National identification no: a)** 35202- 4135948-7 **b)** Alternate national identification number 29553654234 **Address:** Masjid al-Qadesia, 4 Lake Road, Lahore, Pakistan **Listed on:** 14 Mar. 2012 (amended on 1 May 2019) **Other information:** Senior leader and co-founder of Lashkar-e-Tayyiba (QDe.118) (LeT) who has held various senior leader positions in LeT and its front organization, Jamaat-ud-Dawa (JUD) (listed as an alias of LeT). As of 2010, in charge of LeT/JUD finance department, director of its education department and president of its medical wing. Other title: Professor. Review pursuant to Security Council resolution 2253 (2015) was concluded on 21 Feb. 2019. Review pursuant to Security Council resolution 2610 (2021) was concluded on 8 November 2022. INTERPOL-UN Security Council Special Notice web link: https://www.interpol.int/en/How-we-work/Notices/View-UN-Notices-Individuals click here

QDi.087 Name: 1: NURJAMAN 2: RIDUAN 3: ISAMUDDIN 4: na
Title: na **Designation:** na **DOB:** 4 Apr. 1964 **POB:** Cianjur, West Java, Indonesia **Good quality a.k.a.: a)** Hambali **b)** Nurjaman **c)** Isomuddin, Nurjaman Riduan **d)** Hambali Bin Ending **e)** Encep Nurjaman (birth name) **f)** Hambali Ending Hambali **g)** Isamuddin Riduan **h)** Isamudin Ridwan **Low quality a.k.a.:** na **Nationality:** Indonesia **Passport no:** na **National identification no:** na **Address:** na **Listed**

on: 28 Jan. 2003 (amended on 2 Jul. 2007, 27 Jul. 2007, 16 May 2011, 6 Dec. 2019) **Other information:** Senior leader of Jemaah Islamiyah (QDe.092). Brother of Gun Gun Rusman Gunawan (QDi.218). In custody of the United States of America, as of July 2007. Review pursuant to Security Council resolution 1822 (2008) was concluded on 13 Apr. 2010. Review pursuant to Security Council resolution 2368 (2017) was concluded on 4 Dec. 2019. Review pursuant to Security Council resolution 2610 (2021) was concluded on 8 November 2022. INTERPOL-UN Security Council Special Notice web link: https://www.interpol.int/en/How-we-work/Notices/View-UN-Notices-Individuals click here

QDi.368 Name: 1: SHAMIL 2: MAGOMEDOVICH 3: ISMAILOV 4: na
Name (original script): Шамиль Магомедович Измайлов
Title: na **Designation:** na **DOB:** 29 Oct. 1980 **POB:** Astrakhan, Russian Federation **Good quality a.k.a.:** Shamil Magomedovich Aliev (original script: Шамиль Магомедович Алиев) **Low quality a.k.a.:** Abu Hanifa (original script: Абу Ханифа) **Nationality:** Russian Federation **Passport no:** Russian foreign travel passport number 514448632, issued on 8 Sep. 2010, issued in Alexandria, Egypt (issued by Consulate General of the Russian Federation) **National identification no:** Russian Federation national passport 1200075689, issued on 15 Dec. 2000 (issued by Russian Federation) **Address: a)** Syrian Arab Republic (located in as at Aug. 2015) **b)** Iraq (possible alternative location as at Aug. 2015) **Listed on:** 2 Oct. 2015 (amended on 24 Nov. 2020) **Other information:** As at Aug. 2015, leader of Jamaat Abu Hanifa, a terrorist group that is part of the Al-Nusrah Front for the People of the Levant (QDe.137). Physical description: eye colour: brown, hair colour: black, build: slim, height 175-180 cm. Distinguishing marks: long face, speech defect. Wanted by the authorities of the Russian Federation for terrorist crimes committed in its territory. Photo available for inclusion in the INTERPOL-UN Security Council Special Notice. Review pursuant to Security Council resolution 2368 (2017) was concluded on 24 November 2020. INTERPOL-UN Security Council Special Notice web link: https://www.interpol.int/en/How-we-work/Notices/View-UN-Notices-Individuals click here

QDi.360 Name: 1: SALLY-ANNE 2: FRANCES 3: JONES 4: na
Title: na **Designation:** na **DOB:** 17 Nov. 1968 **POB:** Greenwich, Greater London, United Kingdom of Great Britain and Northern Ireland **Good quality a.k.a.:** na **Low quality a.k.a.: a)** Umm Hussain al-Britani **b)** Sakinah Hussain **Nationality:** United Kingdom of Great Britain and Northern Ireland **Passport no:** 519408086, issued on 23 Sep. 2013, issued in United Kingdom of Great Britain and Northern Ireland (expires on 23 Sep. 2023) **National identification no:** na **Address: a)** Syrian Arab Republic (as at 2013) **b)** United Kingdom of Great Britain and Northern Ireland (previous location until 2013) **Listed on:** 28 Sep. 2015 (amended on 24 Nov. 2020) **Other information:** Recruiter for Islamic State in Iraq and the Levant (ISIL), listed as Al-Qaida in Iraq (QDe.115), in the Syrian Arab Republic. Sex: female. Husband’ s name is: Junaid Hussain. Photo available for inclusion in the INTERPOL-UN Security Council Special Notice. Review pursuant to Security Council resolution 2368 (2017) was concluded on 24 November 2020. INTERPOL-UN Security Council Special Notice web link: https://www.interpol.int/en/How-we-work/Notices/View-UN-Notices-Individuals click here

QDi.394 Name: 1: HUSAYN 2: JUAYTHINI 3: na 4: na
Title: na **Designation:** na **DOB:** 3 May 1977 **POB:** Nuseirat Refugee Camp, Gaza Strip, Palestinian Territories **Good quality a.k.a.: a)** Hussein Mohammed Hussein Aljeithni **b)** Husayn Muhammad al-Juaythini **c)** Husayn Muhammad Husayn al-Juaythini **d)** Husayn Muhamad Husayn al-Juaythini **e)** Husayn Muhammad Husayn Juaythini **Low quality a.k.a.:** Abu Muath al-Juaitni **Nationality:** Palestinian **Passport no:** 0363464 (issued by Palestinian Authority) **National identification no:** na **Address:** Gaza Strip, Palestinian Territories **Listed on:** 20 Apr. 2016 **Other information:** Link between Islamic State in Iraq and the Levant (ISIL), listed as Al-Qaida in Iraq (QDe.115), leader Abu Bakr al-Baghdadi, listed as Ibrahim Awwad Ibrahim Ali al-Badri al-Samarrai (QDi.299), and armed groups in Gaza. Was using money to build an ISIL presence in Gaza. Review pursuant to Security Council resolution 2368 (2017) was concluded on 15 November 2021. INTERPOL-UN Security Council Special Notice web link: https://www.interpol.int/en/How-we-work/Notices/View-UN-Notices-Individuals click here

QDi.114 Name: 1: SALIM Y SALAMUDDIN 2: JULKIPLI 3: na 4: na
Title: na **Designation:** na **DOB:** 20 Jun. 1967 **POB:** Tulay, Jolo Sulu, Philippines **Good quality a.k.a.:** **a)** Kipli Sali **b)** Julkipli Salim **Low quality a.k.a.:** na **Nationality:** Philippines **Passport no:** na **National identification no:** na **Address:** na **Listed on:** 9 Sep. 2003 (amended on 23 Feb. 2009, 13 Dec. 2011, 6 Dec. 2019) **Other information:** In detention in the Philippines as at May 2011. Review pursuant to Security Council resolution 1822 (2008) was concluded on 25 May 2010. Review pursuant to Security Council resolution 2368 (2017) was concluded on 4 Dec. 2019. Review pursuant to Security Council resolution 2610 (2021) was concluded on 8 November 2022. INTERPOL-UN Security Council Special Notice web link: https://www.interpol.int/en/How-we-work/Notices/View-UN-Notices-Individuals click here

QDi.072 Name: 1: MEHDI 2: BEN MOHAMED 3: BEN MOHAMED 4: KAMMOUN
Name (original script): المهدي بن محمد بن محمد كمون
Title: na **Designation:** na **DOB:** 3 Apr. 1968 **POB:** Tunis, Tunisia **Good quality a.k.a.:** na **Low quality a.k.a.:** Salmane **Nationality:** Tunisia **Passport no:** Tunisia number M307707, issued on 12 Apr. 2000 (expired on 11 Apr. 2005) **National identification no:** na **Address:** Via Masina Number 7, Milan, Italy **Listed on:** 3 Sep. 2002 (amended on 20 Dec. 2005, 7 Jun. 2007, 23 Dec. 2010, 6 Dec. 2019) **Other information:** Italian Fiscal Code: KMMMHD68D03Z352N. Deported from Italy to Tunisia on 22 July 2005. Serving an eight-year prison term in Tunisia for membership of a terrorist organization abroad as at Jan. 2010. Review pursuant to Security Council resolution 1822 (2008) was concluded on 21 Jun. 2010. Review pursuant to Security Council resolution 2368 (2017) was concluded on 4 Dec. 2019. INTERPOL-UN Security Council Special Notice web link: https://www.interpol.int/en/How-we-work/Notices/View-UN-Notices-Individuals click here

QDi.416 Name: 1: MOHAMMED 2: YUSIP 3: KARIM 4: na
Title: na **Designation:** na **DOB:** 11 Oct. 1978 **POB:** Indonesia **Good quality a.k.a.:** na **Low quality a.k.a.:** **a)** Abu Walid al Indunisi **b)** Zidni Elma **c)** Udtadz Syaifudin **d)** Mohammad Yusef Karim Faiz **e)** Muh Saifudin **f)** Kembar Khalid **g)** Mohamad Yusuf Karim Saifullah Faiz **h)** Mohammad Saifuddin Mohammad Yusuf Faiz **i)** Ustadz Faiz **j)** Saifudin Faiz **k)** Kholid Faiz **l)** Abdullah Faiz **m)** Fauz Faturohman **Nationality:** Indonesia **Passport no:** na **National identification no:** na **Address:** Syrian Arab Republic (location since 2015) **Listed on:** 23 Aug. 2018 **Other information:** Senior member of Islamic State in Iraq and the Levant (ISIL), listed as Al-Qaida in Iraq (QDe.115). Recruited for ISIL and instructed individuals to perpetrate terrorist acts via online video. Physical description: hair colour: black; build: slight. Speaks Indonesian, Arabic and Mindanao dialect. INTERPOL-UN Security Council Special Notice web link: https://www.interpol.int/en/How-we-work/Notices/View-UN-Notices-Individuals click here

QDi.135 Name: 1: DAWOOD 2: IBRAHIM 3: KASKAR 4: na
Title: Sheikh **Designation:** na **DOB:** 26 Dec. 1955 **POB:** Kher, Ratnagiri, Maharashtra, India **Good quality a.k.a.:** **a)** Dawood Ebrahim **b)** Sheikh Dawood Hassan **c)** Abdul Hamid Abdul Aziz **d)** Anis Ibrahim **e)** Aziz Dilip **f)** Daud Hasan Shaikh Ibrahim Kaskar **g)** Daud Ibrahim Memon Kaskar **h)** Dawood Hasan Ibrahim Kaskar **i)** Dawood Ibrahim Memon **j)** Dawood Sabri **k)** Kaskar Dawood Hasan **l)** Shaikh Mohd Ismail Abdul Rehman **m)** Dowood Hassan Shaikh Ibrahim **n)** Dawood Bhai **Low quality a.k.a.:** **a)** Ibrahim Shaikh Mohd Anis **b)** Shaikh Ismail Abdul **c)** Hizrat **d)** Sheikh Farooqi **e)** Bada Seth **f)** Bada Bhai **g)** Iqbal Bhai **h)** Mucchad **i)** Haji Sahab **Nationality:** India **Passport no:** **a)** India number A-333602, issued on 4 Jun. 1985, issued in Bombay, India (passport subsequently revoked by the Government of India) **b)** India number M110522, issued on 13 Nov. 1978, issued in Bombay, India **c)** India number R841697, issued on 26 Nov. 1981, issued in Bombay **d)** India number F823692, issued on 2 Sep. 1989 ((JEDDAH) issued by CGI in Jeddah) **e)** India number A501801, issued on 26 Jul. 1985, issued in BOMBAY **f)** India number K560098, issued on 30 Jul. 1975, issued in BOMBAY **g)** V57865, issued on 3 Oct. 1983, issued in BOMBAY **h)** India number P537849, issued on 30 Jul. 1979, issued in BOMBAY **i)** A717288, issued on 18 Aug. 1985, issued in Dubai (MISUSE) **j)** Pakistan number G866537, issued on 12 Aug. 1991, issued in Rawalpindi

(MISUSE) **k)** C-267185 (issued in Karachi in Jul.1996) **l)** H-123259 (issued in Rawalpindi in Jul. 2001) **m)** G-869537 (issued in Rawalpindi) **n)** KC-285901 **National identification no:** na **Address: a)** Karachi, Pakistan (White House, Near Saudi Mosque, Clifton) **b)** House Nu 37 - 30th Street - defence, Housing Authority, Karachi, Pakistan **c)** Palatial bungalow in the hilly area of Noorabad in Karachi, Pakistan **Listed on:** 3 Nov. 2003 (amended on 21 Mar. 2006, 25 Jul. 2006, 2 Jul. 2007, 11 Mar. 2010, 22 Aug. 2016, 24 Nov. 2020) **Other information:** Father' s name is Sheikh Ibrahim Ali Kaskar, mother' s name is Amina Bi, wife' s name is Mehjabeen Shaikh. International arrest warrant issued by the Government of India. Review pursuant to Security Council resolution 1822 (2008) was concluded on 20 May 2010. Review pursuant to Security Council resolution 2368 (2017) was concluded on 24 November 2020. INTERPOL-UN Security Council Special Notice web link: https://www.interpol.int/en/How-we-work/Notices/View-UN-Notices-Individuals click here

QDi.372 Name: 1: GULMUROD 2: KHALIMOV 3: na 4: na
Title: na **Designation:** na **DOB: a)** 14 May 1975 **b)** Approximately 1975 **POB: a)** Varzob area, Tajikistan **b)** Dushanbe, Tajikistan **Good quality a.k.a.:** na **Low quality a.k.a.:** na **Nationality:** Tajikistan **Passport no:** na **National identification no:** na **Address:** Syrian Arab Republic (location as at Sep. 2015) **Listed on:** 29 Feb. 2016 (amended on 24 Nov. 2020) **Other information:** Syria-based military expert, member and recruiter of Islamic State in Iraq and the Levant, listed as Al-Qaida in Iraq (QDe.115). Wanted by the Government of Tajikistan. Review pursuant to Security Council resolution 2368 (2017) was concluded on 24 November 2020. INTERPOL-UN Security Council Special Notice web link: https://www.interpol.int/en/How-we-work/Notices/View-UN-Notices-Individuals click here

QDi.306 Name: 1: MUSTAFA 2: HAJJI 3: MUHAMMAD 4: KHAN
Name (original script): مصطفى حجي محمد خان
Title: na **Designation:** na **DOB: a)** (Between Aug. and Sep. 1977) **b)** 1976 **POB: a)** Al-Madinah, Saudi Arabia **b)** Sangrar, Sindh Province, Pakistan **Good quality a.k.a.: a)** حسن غول (Hassan Ghul; Hassan Gul; Hasan Gul) **b)** Khalid Mahmud **Low quality a.k.a.: a)** Ahmad Shahji **b)** Mustafa Muhammad **c)** Abu Gharib al-Madani **d)** أبو شيماء (Abu-Shaima; Abu- Shayma) **Nationality: a)** Pakistan **b)** Saudi Arabian **Passport no:** na **National identification no:** na **Address:** na **Listed on:** 14 Mar. 2012 **Other information:** Al-Qaida (QDe.004) facilitator, courier and operative. As of 2010, facilitated activities for senior Pakistan-based Al-Qaida operatives. Review pursuant to Security Council resolution 2368 (2017) was concluded on 15 November 2021. INTERPOL-UN Security Council Special Notice web link: https://www.interpol.int/en/How-we-work/Notices/View-UN-Notices-Individuals click here

QDi.336 Name: 1: ANAS 2: HASAN 3: KHATTAB 4: na
Title: na **Designation:** na **DOB:** 7 Apr. 1986 **POB:** Damascus, Syrian Arab Republic **Good quality a.k.a.:** Samir Ahmed al-Khayat **Low quality a.k.a.: a)** Hani **b)** Abu Hamzah **c)** Abu-Ahmad Hadud **Nationality:** Syrian Arab Republic **Passport no:** na **National identification no:** na **Address:** na **Listed on:** 23 Sep. 2014 (amended on 25 Oct. 2016, 24 Nov. 2020) **Other information:** Administrative amir of Al-Nusrah Front for the People of the Levant (QDe.137). Review pursuant to Security Council resolution 2368 (2017) was concluded on 24 November 2020. INTERPOL-UN Security Council Special Notice web link: https://www.interpol.int/en/How-we-work/Notices/View-UN-Notices-Individuals click here

QDi.418 Name: 1: MUHAMMED 2: REZA 3: LAHAMAN 4: KIRAM
Title: na **Designation:** na **DOB:** 3 Mar. 1990 **POB:** Zamboanga City, Zamboanga del Sur, Philippines **Good quality a.k.a.:** na **Low quality a.k.a.: a)** Abdul Rahman **b)** Abu Abdul Rahman al Filipini **c)** Abtol Rahman **Nationality:** Philippines **Passport no: a)** Philippines number XX3966391, issued on 25 Feb. 2015 (issued by the Department of Foreign Affairs of Philippines, expiration date 24 Feb. 2020) **b)** Philippines number EC3524065 **National identification no:** na **Address: a)** Brgy Recodo, Zamboanga City, Western Mindanao, Philippines (previous address) **b)** 96 IlangIlang, Sarmiento Subdivision, Panabo, Davao City, Eastern Mindanao, Philippines (previous address) **c)** Syrian Arab Republic (location since 2015) **Listed on:** 23 Aug. 2018 **Other information:** Senior member of

Islamic State in Iraq and the Levant (ISIL), listed as Al-Qaida in Iraq (QDe.115). Recruited for ISIL and instructed individuals to perpetrate terrorist acts via online video. Physical description: height: 156cm; weight: 60 kg (as at Sep. 2016); eye colour: black; hair colour: black; build: medium; high cheekbones. Speaks Tagalog, English, Arabic. INTERPOL-UN Security Council Special Notice web link: https://www.interpol.int/en/How-we-work/Notices/View-UN-Notices-Individuals click here

QDi.340 Name: 1: EMILIE 2: EDWIGE 3: KONIG 4: na
Title: na **Designation:** na **DOB:** 9 Dec. 1984 **POB:** Ploemeur, France **Good quality a.k.a.:** na **Low quality a.k.a.:** Emilie Samra Konig **Nationality:** France **Passport no:** French passport number 05AT521433, issued on 30 Nov. 2005 (issued by the sous-prefecture of police of Lorient, France) **National identification no: a)** French national identity card number 050456101445, issued on 19 May 2005 (issued by the sous-prefecture of police of Lorient, France) **b)** French identity card number 0205561020089, issued on 30 May 2002 (issued under name Emilie Edwige Konig) **Address:** Syrian Arab Republic (located in since 2013) **Listed on:** 23 Sep. 2014 (amended on 24 Jun. 2016) **Other information:** French terrorist fighter who travelled to Syria and joined Islamic State in Iraq and the Levant, listed as Al-Qaida in Iraq (AQI) (QDe.115). Active in radicalizing and propagating Al-Qaida’ s (QDe.004) ideology through the Internet. Incites violent activities against France. French arrest warrant issued on 12 Jun. 2015 by a magistrate of the anti-terrorism division of the Prosecutor’ s Office in Paris for her participation in a terrorist criminal association. Review pursuant to Security Council resolution 2368 (2017) was concluded on 15 November 2021. INTERPOL-UN Security Council Special Notice web link: https://www.interpol.int/en/How-we-work/Notices/View-UN-Notices-Individuals click here

QDi.408 Name: 1: ALEXANDA 2: AMON 3: KOTEY 4: na
Title: na **Designation:** na **DOB:** 13 Dec. 1983 **POB:** London, United Kingdom of Great Britain and Northern Ireland **Good quality a.k.a.: a)** Alexe Kotey **b)** Alexanda Kote **Low quality a.k.a.:** na **Nationality:** United Kingdom of Great Britain and Northern Ireland **Passport no:** United Kingdom of Great Britain and Northern Ireland number 094477324, issued on 5 Mar. 2005 **National identification no:** na **Address:** United States of America **Listed on:** 20 Jul. 2017 (amended on 2 Feb. 2023) **Other information:** Foreign terrorist fighter with Islamic State in Iraq and the Levant (ISIL), listed as Al-Qaida in Iraq (QDe.115), in the Syrian Arab Republic. Sentenced to life imprisonment on 29 April 2022 in the United States of America, Federal Bureau of Prisons inmate number 11685-509. Physical description: eye colour: dark brown; hair colour: black; complexion: dark. Distinguishing marks: beard. Ethnic background: Ghanaian Cypriot. INTERPOL-UN Security Council Special Notice web link: https://www.interpol.int/en/How-we-work/Notices/View-UN-Notices-Individuals click here

QDi.425 Name: 1: AMADOU 2: KOUFA 3: na 4: na
Title: na **Designation:** na **DOB:** Approximately 1958 **POB:** Koufa, Mali **Good quality a.k.a.: a)** Amadou Barry **b)** Amadou Kouffa **c)** Hamadoun Koufa **d)** Hamadoun Kouffa **e)** Hamadou Koufa **f)** Hamadou Kouffa **Low quality a.k.a.:** na **Nationality:** na **Passport no:** na **National identification no:** na **Address:** Mali **Listed on:** 4 Feb. 2020 **Other information:** Founder of the Katiba Macina of Jama'a Nusrat ul-Islam wa al-Muslimin (JNIM) (QDe.159), executive of the Organization of Al-Qaida in the Islamic Maghreb (AQIM) (QDe.014). Eye colour: brown. Hair colour: dark. INTERPOL-UN Security Council Special Notice web link: https://www.interpol.int/en/How-we-work/Notices/View-UN-Notices-Individuals click here

QDi.383 Name: 1: MORAD 2: LAABOUDI 3: na 4: na
Title: na **Designation:** na **DOB:** 26 Feb. 1993 **POB:** Morocco **Good quality a.k.a.:** na **Low quality a.k.a.: a)** Abu Ismail **b)** Abu Ismail al-Maghribi **Nationality:** Morocco **Passport no:** Morocco number UZ6430184 **National identification no:** Morocco CD595054 **Address:** Turkey **Listed on:** 29 Feb. 2016 **Other information:** Facilitator for travel of foreign terrorist fighters to join Islamic State in Iraq and the Levant, listed as Al-Qaida in Iraq (QDe.115), in Syrian Arab Republic. Review pursuant to Security Council resolution 2368 (2017) was concluded on 15 November 2021. INTERPOL-UN

Security Council Special Notice web link: https://www.interpol.int/en/How-we-work/Notices/View-UN-Notices-Individuals click here

QDi.190 Name: 1: ABDELKADER 2: LAAGOUB 3: na 4: na
Name (original script): عبد القادر لاغوب
Title: na **Designation:** na **DOB:** 23 Apr. 1966 **POB:** Casablanca, Morocco **Good quality a.k.a.:** na **Low quality a.k.a.:** Rachid **Nationality:** Morocco **Passport no:** Morocco number D-379312 **National identification no:** (Moroccan national identity card DE- 473900) **Address:** Number 4, Via Europa, Paderno Ponchielli, Cremona, Italy **Listed on:** 29 Jul. 2005 (amended on 21 Dec. 2007, 13 Dec. 2011, 6 Dec. 2019) **Other information:** Italian Fiscal code: LGBBLK66D23Z330U. Father' s name is Mamoune Mohamed. Mother' s name is Fatna Ahmed. Review pursuant to Security Council resolution 1822 (2008) was concluded on 8 Jun. 2010. Review pursuant to Security Council resolution 2368 (2017) was concluded on 4 Dec. 2019. INTERPOL-UN Security Council Special Notice web link: https://www.interpol.int/en/How-we-work/Notices/View-UN-Notices-Individuals click here

QDi.319 Name: 1: MOHAMED 2: LAHBOUS 3: na 4: na
Name (original script): محمد لحبوس
Title: na **Designation:** na **DOB:** 1978 **POB:** Mali **Good quality a.k.a.: a)** Mohamed Ennouini **b)** Hassan **c)** Hocine **Low quality a.k.a.:** na **Nationality:** Mali **Passport no:** na **National identification no:** na **Address:** Mali **Listed on:** 24 Oct. 2013 (amended on 29 Mar. 2019, 1 May 2019) **Other information:** Member of the Mouvement pour l' Unification et le Jihad en Afrique de l' Ouest (MUJAO) (QDe.134). Reportedly deceased as of 14 February 2018 . Review pursuant to Security Council resolution 2253 (2015) was concluded on 21 February 2019. Review pursuant to Security Council resolution 2610 (2021) was concluded on 8 November 2022. INTERPOL-UN Security Council Special Notice web link: https://www.interpol.int/en/How-we-work/Notices/View-UN-Notices-Individuals click here

QDi.062 Name: 1: MOHAMED 2: LAKHAL 3: na 4: na
Name (original script): محمد لكحل
Title: na **Designation:** na **DOB:** 5 Feb. 1970 **POB:** Tunis, Tunisia **Good quality a.k.a.: a)** Lased Ben Heni born 5 Feb. 1969 in Tripoli, Libya **b)** Al-As'ad Ben Hani born 5 Feb. 1969 in Tripoli, Libya **c)** Mohamed Ben Belgacem Awani **d)** Mohamed Aouani born 5 Feb. 1970 in Tunis, Tunisia (formerly listed as) **Low quality a.k.a.: a)** Mohamed Abu Abda **b)** Abu Obeida **Nationality:** Tunisia **Passport no:** na **National identification no:** Tunisia W374031, issued on 11 Apr. 2011 **Address:** na **Listed on:** 24 Apr. 2002 (amended on 26 Nov. 2004, 9 Sep. 2005, 31 Jul. 2006, 23 Dec. 2010, 24 Nov. 2014, 6 Dec. 2019, 7 May 2020) **Other information:** Professor of Chemistry. Deported from Italy to Tunisia on 27 Aug. 2006. Legally changed family name from Aouani to Lakhal in 2014. Review pursuant to Security Council resolution 1822 (2008) was concluded on 22 Apr. 2010. Review pursuant to Security Council resolution 2368 (2017) was concluded on 4 Dec. 2019 INTERPOL-UN Security Council Special Notice web link: https://www.interpol.int/en/How-we-work/Notices/View-UN-Notices-Individuals click here

QDi.264 Name: 1: ZAKI-UR-REHMAN 2: LAKHVI 3: na 4: na
Title: na **Designation:** na **DOB:** 30 Dec. 1960 **POB:** Okara, Pakistan **Good quality a.k.a.: a)** Zakir Rehman Lakvi **b)** Zaki Ur-Rehman Lakvi **c)** Kaki Ur-Rehman **d)** Zakir Rehman **e)** Abu Waheed Irshad Ahmad Arshad **Low quality a.k.a.:** Chachajee **Nationality:** Pakistan **Passport no:** na **National identification no:** Pakistani 61101-9618232-1 **Address: a)** Barahkoh, P.O. DO, Tehsil and District Islamabad, Pakistan (location as at May 2008) **b)** Chak No. 18/IL, Rinala Khurd, Tehsil Rinala Khurd, District Okara, Pakistan (previous location) **Listed on:** 10 Dec. 2008 (amended on 1 May 2019) **Other information:** Chief of operations of Lashkar-e-Tayyiba (listed under permanent reference number QDe.118). Review pursuant to Security Council resolution 2253 (2015) was concluded on 21 Feb. 2019. Review pursuant to Security Council resolution 2610 (2021) was concluded on 8

November 2022. INTERPOL-UN Security Council Special Notice web link: https://www.interpol.int/en/How-we-work/Notices/View-UN-Notices-Individuals click here

QDi.247 Name: 1: RUBEN 2: PESTANO 3: LAVILLA, JR 4: na
Title: Sheik **Designation:** na **DOB:** 4 Oct. 1972 **POB:** Sitio Banga Maiti, Barangay Tranghawan, Lambunao, Iloilo, Philippines **Good quality a.k.a.: a)** Reuben Lavilla **b)** Sheik Omar **c)** Mile D Lavilla **d)** Reymund Lavilla **e)** Ramo Lavilla **f)** Mike de Lavilla **g)** Abdullah Muddaris **h)** Ali Omar **i)** Omar Lavilla **j)** Omar Labella **Low quality a.k.a.: a)** So **b)** Eso **c)** Junjun **Nationality:** Philippines **Passport no: a)** Philippines number MM611523 (2004) **b)** Philippines number EE947317 (2000-2001) **c)** Philippines number P421967 (1995-1997) **National identification no:** na **Address:** 10th Avenue, Caloocan City, Philippines **Listed on:** 4 Jun. 2008 (amended on 16 Sep. 2008, 13 Dec. 2011, 9 May 2018, 6 Dec. 2019) **Other information:** Spiritual leader of the Rajah Solaiman Movement (QDe.128). Associated with Khadafi Abubakar Janjalani (deceased). In detention in the Philippines as of May 2011. Review pursuant to Security Council resolution 1822 (2008) was concluded on 13 May 2010. Review pursuant to Security Council resolution 2368 (2017) was concluded on 4 Dec. 2019. INTERPOL-UN Security Council Special Notice web link: https://www.interpol.int/en/How-we-work/Notices/View-UN-Notices-Individuals click here

QDi.155 Name: 1: DJAMEL 2: LOUNICI 3: na 4: na
Name (original script): جمال لونيسي
Title: na **Designation:** na **DOB:** 1 Feb. 1962 **POB:** Algiers, Algeria **Good quality a.k.a.:** Jamal Lounici **Low quality a.k.a.:** na **Nationality:** Algeria **Passport no:** na **National identification no:** na **Address:** Algeria **Listed on:** 16 Jan. 2004 (amended on 7 Apr. 2008, 2 Dec. 2008, 30 Jan. 2009, 16 May 2011, 14 Feb. 2018, 29 May 2018, 24 Nov. 2020) **Other information:** Father's name is Abdelkader. Mother's name is Djohra Birouch. Returned from France to Algeria where he resides since Sep. 2008. Review pursuant to Security Council resolution 1822 (2008) was concluded on 27 Jul. Review pursuant to Security Council resolution 2368 (2017) was concluded on 24 November 2020. INTERPOL-UN Security Council Special Notice web link: https://www.interpol.int/en/How-we-work/Notices/View-UN-Notices-Individuals click here

QDi.413 Name: 1: MYRNA 2: AJIJUL 3: MABANZA 4: na
Title: na **Designation:** na **DOB:** 11 Jul. 1991 **POB:** na **Good quality a.k.a.: a)** Myrna Adijul Mabanza **b)** Myrna Ajilul Mabanza **Low quality a.k.a.:** na **Nationality:** Philippines **Passport no:** na **National identification no: a)** Voter ID 73320881AG1191MAM20000 **b)** Student ID 200801087 **c)** Other ID 140000900032 **Address: a)** Basilan Province, Philippines **b)** Zamboanga City, Philippines (previous address) **c)** Jeddah, Saudi Arabia (previous address) **d)** Daina, Saudi Arabia (previous address) **Listed on:** 18 Jun. 2018 **Other information:** Facilitator for the Islamic State in Iraq and the Levant (ISIL), listed as Al-Qaida in Iraq (QDe.115). Gender: female. INTERPOL-UN Security Council Special Notice web link: https://www.interpol.int/en/How-we-work/Notices/View-UN-Notices-Individuals click here

QDi.356 Name: 1: AQSA 2: MAHMOOD 3: na 4: na
Title: na **Designation:** na **DOB:** 11 May 1994 **POB:** Glasgow, Scotland, United Kingdom of Great Britain and Northern Ireland **Good quality a.k.a.:** na **Low quality a.k.a.:** Umm Layth **Nationality:** United Kingdom of Great Britain and Northern Ireland **Passport no:** United Kingdom of Great Britain and Northern Ireland number 720134834, issued on 27 Jun. 2012 (expires on 27 Jun. 2022) **National identification no:** na **Address: a)** Syrian Arab Republic (as at Nov. 2013) **b)** United Kingdom of Great Britain and Northern Ireland (previous address) **Listed on:** 28 Sep. 2015 (amended on 24 Nov. 2020) **Other information:** Recruiter for Islamic State in Iraq and the Levant, listed as Al-Qaida in Iraq (QDe.115), in the Syrian Arab Republic, and a key figure in the the Al-Khanssaa brigade, a female ISIL brigade established in Al-Raqqa to enforce ISIL’s interpretation of Sharia law. Sex: female. Photo available for inclusion in the INTERPOL-UN Security Council Special Notice. Review pursuant to Security Council resolution 2368 (2017) was concluded on 24 November

2020. INTERPOL-UN Security Council Special Notice web link: https://www.interpol.int/en/How-we-work/Notices/View-UN-Notices-Individuals click here

QDi.433 Name: 1: ABDUL 2: REHMAN 3: MAKKI 4: na
Title: na **Designation:** na **DOB:** 10 Dec. 1954 **POB:** Bahawalpur, Punjab Province, Pakistan **Good quality a.k.a.: a)** Abdur Rehman Makki **b)** Abdur Rahman Makki **c)** Abdul Rahman Makki **d)** Hafiz Abdul Rahman Makki **e)** Hafiz Abdul Rehman Makki **f)** Hafiz Abdul Rehman **Low quality a.k.a.:** na **Nationality:** Pakistan **Passport no: a)** Pakistan number CG9153881, issued on 2 Nov. 2007 **b)** Pakistan number A5199819 **National identification no: a)** Pakistan 6110111883885 **b)** Pakistan 34454009709 **Address:** Tayyiba Markaz, Muridke, Punjab Province, Pakistan **Listed on:** 16 Jan. 2023 **Other information:** He is deputy Amir/Chief of LASHKAR-E-TAYYIBA (LET) (QDe.118) a.k.a JAMAAT-UD-DAWA (JUD) and Head of Political Affairs Wing JUD/LET. He also served as head of LET's foreign relations department and member of Shura (governing body). He is the brother-in-law of JUD/LET Chief Hafiz Muhammad Saeed (QDi.263). Father's name is Hafiz Abdullah Bahwalpuri. Photo is available for inclusion in the INTERPOL-UN Security Council Special Notice. INTERPOL-UN Security Council Special Notice web link: https://www.interpol.int/en/How-we-work/Notices/View-UN-Notices-Individuals click here

QDi.423 Name: 1: ALI 2: MAYCHOU 3: na 4: na
Name (original script): علي ما يشو
Title: na **Designation:** na **DOB:** 25 May 1983 **POB:** Taza, Morocco **Good quality a.k.a.: a)** Abderahmane al Maghrebi **b)** Abderrahmane le Marocain **Low quality a.k.a.:** Abou Abderahmane Sanhaji **Nationality:** Morocco **Passport no:** Morocco number V06359364 **National identification no:** Morocco identity card AB704306 **Address:** Mali **Listed on:** 14 Aug. 2019 **Other information:** Member of Al Qaida in the Islamic Maghreb (AQIM) (QDe.014), Ansar Eddine (QDe.135), and Jama'a Nusrat ul-Islam wa al-Muslimin (JNIM) (QDe.159). Physical description: height: 185 cm; weight: 80 kg INTERPOL-UN Security Council Special Notice web link: https://www.interpol.int/en/How-we-work/Notices/View-UN-Notices-Individuals click here

QDi.126 Name: 1: YUNOS 2: UMPARA 3: MOKLIS 4: na
Title: na **Designation:** na **DOB:** 7 Jul. 1966 **POB:** Lanao del Sur, Philippines **Good quality a.k.a.: a)** Muklis Yunos **b)** Mukhlis Yunos (previously listed as) **c)** Saifullah Mukhlis Yunos **d)** Saifulla Moklis Yunos **Low quality a.k.a.:** Hadji Onos **Nationality:** Philippines **Passport no:** na **National identification no:** na **Address:** Philippines (remains incarcerated as of May 2017) **Listed on:** 9 Sep. 2003 (amended on 9 Sep. 2005, 23 Feb. 2009, 3 Jun. 2009, 16 May 2011, 22 Sep. 2017, 24 Nov. 2020) **Other information:** Sentenced to life without parole in the Philippines on 23 Jan. 2009 for his involvement in the bombings of 30 Dec. 2000 in Manila, the Philippines. Review pursuant to Security Council resolution 1822 (2008) was concluded on 25 May 2010. Review pursuant to Security Council resolution 2368 (2017) was concluded on 24 November 2020. Photos included in INTERPOL-UN Security Council Special Notice web link: https://www.interpol.int/en/How-we-work/Notices/View-UN-Notices-Individuals click here

QDi.147 Name: 1: MOHAMED 2: AMIN 3: MOSTAFA 4: na
Name (original script): محمد أمين مصطفى
Title: na **Designation:** na **DOB:** 11 Oct. 1975 **POB:** Kirkuk, Iraq **Good quality a.k.a.:** na **Low quality a.k.a.:** na **Nationality:** Iraq **Passport no:** na **National identification no:** na **Address:** Via della Martinella 132, Parma, Italy (Domicile) **Listed on:** 12 Nov. 2003 (amended on 9 Sep. 2005, 7 Jun. 2007, 16 May 2011, 25 Oct. 2016, 1 May 2019) **Other information:** Under administrative control measure in Italy scheduled to expire on 15 Jan. 2012. Review pursuant to Security Council resolution 1822 (2008) was concluded on 21 Jun. 2010. Review pursuant to Security Council resolution 2253 (2015) was concluded on 21 Feb. 2019. Review pursuant to Security Council resolution 2610 (2021) was concluded on 8 November 2022. INTERPOL-UN Security Council Special Notice web link: https://www.interpol.int/en/How-we-work/Notices/View-UN-Notices-Individuals click here

QDi.129 Name: 1: DJAMEL 2: MOUSTFA 3: na 4: na
Name (original script): جمال مصطفى
Title: na **Designation:** na **DOB:** 28 Sep. 1973 **POB:** Tiaret, Algeria **Good quality a.k.a.: a)** Kalad Belkasam born 31 Dec. 1979 **b)** Mostafa Djamel born 31 Dec. 1979 in Maskara, Algeria **c)** Mostefa Djamel born 26 Sep. 1973 in Mahdia, Algeria **d)** Mustafa Djamel born 31 Dec. 1979 in Mascara, Algeria **e)** Balkasam Kalad born 26 Aug. 1973 in Algiers, Algeria **f)** Bekasam Kalad born 26 Aug. 1973 in Algiers, Algeria **g)** Belkasam Kalad born 26 Aug. 1973 in Algiers, Algeria **h)** Damel Mostafa born 31 Dec. 1979 in Algiers, Algeria **i)** Djamal Mostafa born 31 Dec. 1979 in Maskara, Algeria **j)** Djamal Mostafa born 10 Jun. 1982 **k)** Djamel Mostafa born 31 Dec. 1979 in Maskara, Algeria **l)** Djamel Mostafa born 31 Dec. 1979 in Algiers, Algeria **m)** Fjamel Moustfa born 28 Sep. 1973 in Tiaret, Algeria **n)** Djamel Mustafa born 31 Dec. 1979 **o)** Ali Barkani born 22 Aug. 1973 in Morocco **p)** Djamel Mustafa born 31 Dec. 1979 in Mascara, Algeria **Low quality a.k.a.:** Mustafa **Nationality:** Algeria **Passport no:** na **National identification no: a)** Counterfeit Danish driving licence number 20645897 (made out to Ali Barkani, born on 22 Aug. 1973 in Morocco) **b)** Algeria Birth certificate , issued in Algeria (issued for Djamel Mostefa, born on 25 Sep. 1973 in Mehdia, Tiaret province, Algeria) **Address:** Algeria **Listed on:** 23 Sep. 2003 (amended on 7 Sep. 2007, 7 Apr. 2008, 25 Jan. 2010, 16 May 2011, 1 May 2019) **Other information:** Father's name is Djelalli Moustfa. Mother's name is Kadeja Mansore. Deported from Germany to Algeria in Sep. 2007. Review pursuant to Security Council resolution 1822 (2008) was concluded on 19 Oct. 2009. Review pursuant to Security Council resolution 2253 (2015) was concluded on 21 Feb. 2019. Review pursuant to Security Council resolution 2610 (2021) was concluded on 8 November 2022. INTERPOL-UN Security Council Special Notice web link: https://www.interpol.int/en/How-we-work/Notices/View-UN-Notices-Individuals click here

QDi.238 Name: 1: MUBARAK 2: MUSHAKHAS 3: SANAD 4: MUBARAK AL-BATHALI
Name (original script): مبارك مشخص سند مبارك البذالي
Title: na **Designation:** na **DOB:** 1 Oct. 1961 **POB:** Kuwait **Good quality a.k.a.: a)** Mubarak Mishkhis Sanad Al-Bathali **b)** Mubarak Mishkhis Sanad Al-Badhali **c)** Mubarak Al-Bathali **d)** Mubarak Mishkhas Sanad Al-Bathali **e)** Mubarak Mishkhas Sanad Al-Bazali **f)** Mobarak Meshkhas Sanad Al-Bthaly **Low quality a.k.a.:** Abu Abdulrahman **Nationality:** Kuwait **Passport no: a)** Kuwait number 101856740, issued on 12 May 2005 (and expired on 11 May 2007) **b)** Kuwait number 002955916 **National identification no:** Kuwait 261122400761 **Address:** Al-Salibekhat area, Kuwait (residence as at Mar. 2009) **Listed on:** 16 Jan. 2008 (amended on 1 Jul. 2008, 23 Jul. 2008, 25 Jan. 2010, 1 May 2019, 2 Feb. 2023) **Other information:** Sentenced to prison by Kuwait on 24 August 2018. Review pursuant to Security Council resolution 1822 (2008) was concluded on 14 Sep. 2009. Review pursuant to Security Council resolution 2253 (2015) was concluded on 21 Feb. 2019. INTERPOL-UN Security Council Special Notice web link: https://www.interpol.int/en/How-we-work/Notices/View-UN-Notices-Individuals click here

QDi.272 Name: 1: MOHAMMED 2: YAHYA 3: MUJAHID 4: na
Title: na **Designation:** na **DOB:** 12 Mar. 1961 **POB:** Lahore, Punjab Province, Pakistan **Good quality a.k.a.:** Mohammad Yahya Aziz **Low quality a.k.a.:** na **Nationality:** Pakistan **Passport no:** na **National identification no:** Pakistani 35404-1577309-9 **Address:** na **Listed on:** 29 Jun. 2009 (amended on 1 May 2019) **Other information:** Associated with Lashkar-e-Tayyiba (QDe.118). In detention as at June 2009. Review pursuant to Security Council resolution 2253 (2015) was concluded on 21 Feb. 2019. Review pursuant to Security Council resolution 2610 (2021) was concluded on 8 November 2022. INTERPOL-UN Security Council Special Notice web link: https://www.interpol.int/en/How-we-work/Notices/View-UN-Notices-Individuals click here

QDi.119 Name: 1: ARIS 2: MUNANDAR 3: na 4: na
Title: na **Designation:** na **DOB:** 1 Jan. 1971 **POB:** Sambi, Boyolali, Java, Indonesia **Good quality a.k.a.:** na **Low quality a.k.a.:** na **Nationality:** Indonesia (as at Dec. 2003) **Passport no:** na **National identification no:** na **Address:** na **Listed on:** 9 Sep. 2003 (amended on 9 Sep. 2005, 4 Oct. 2006, 12 Dec. 2014, 1 May 2019, 2 Feb. 2023) **Other information:** Review pursuant to Security Council

resolution 1822 (2008) was concluded on 25 May 2010. Review pursuant to Security Council resolution 2253 (2015) was concluded on 21 Feb. 2019. INTERPOL-UN Security Council Special Notice web link: https://www.interpol.int/en/How-we-work/Notices/View-UN-Notices-Individuals click here

QDi.120 Name: 1: ABDUL HAKIM 2: MURAD 3: na 4: na
Name (original script): عبد الحكيم مراد
Title: na **Designation:** na **DOB:** 11 Apr. 1968 **POB:** Kuwait **Good quality a.k.a.:** **a)** Murad, Abdul Hakim Hasim **b)** Murad, Abdul Hakim Ali Hashim **c)** Murad, Abdul Hakim al Hashim **d)** Saeed Akman **e)** Saeed Ahmed **f)** Abdul Hakim Ali al-Hashem Murad **Low quality a.k.a.:** na **Nationality:** Pakistan **Passport no:** **a)** Pakistan number 665334, issued in Kuwait **b)** Pakistan number 917739, issued on 8 Aug. 1991, issued in Pakistan (expired on 7 Aug. 1996) **National identification no:** na **Address:** na **Listed on:** 9 Sep. 2003 (amended on 16 May 2011, 6 Dec. 2019) **Other information:** Mother's name is Aminah Ahmad Sher al-Baloushi. In custody of the United States. Review pursuant to Security Council resolution 1822 (2008) was concluded on 25 May 2010. Review pursuant to Security Council resolution 2368 (2017) was concluded on 4 Dec. 2019. INTERPOL-UN Security Council Special Notice web link: https://www.interpol.int/en/How-we-work/Notices/View-UN-Notices-Individuals click here

QDi.196 Name: 1: ALI 2: SAYYID 3: MUHAMED 4: MUSTAFA BAKRI
Name (original script): على السيد محمد مصطفى بكري
Title: na **Designation:** na **DOB:** 18 Apr. 1966 **POB:** Beni-Suef, Egypt **Good quality a.k.a.:** **a)** Ali Salim **b)** Abd Al-Aziz al-Masri **Low quality a.k.a.:** na **Nationality:** Egypt **Passport no:** na **National identification no:** na **Address:** na **Listed on:** 29 Sep. 2005 (amended on 13 Dec. 2011, 6 Dec. 2019) **Other information:** Member of the Shura Council of Al-Qaida (QDe.004) and Egyptian Islamic Jihad (QDe.003). Review pursuant to Security Council resolution 1822 (2008) was concluded on 1 Jun. 2010. Review pursuant to Security Council resolution 2368 (2017) was concluded on 4 Dec. 2019. Review pursuant to Security Council resolution 2610 (2021) was concluded on 8 November 2022. INTERPOL-UN Security Council Special Notice web link: https://www.interpol.int/en/How-we-work/Notices/View-UN-Notices-Individuals click here

QDi.357 Name: 1: ASEEL 2: MUTHANA 3: na 4: na
Title: na **Designation:** na **DOB:** 22 Nov. 1996 **POB:** Cardiff, United Kingdom of Great Britain and Northern Ireland **Good quality a.k.a.:** na **Low quality a.k.a.:** na **Nationality:** United Kingdom of Great Britain and Northern Ireland **Passport no:** United Kingdom of Great Britain and Northern Ireland number 516088643, issued on 7 Jan. 2014 (expires on 7 Jan. 2024) **National identification no:** na **Address:** **a)** Syrian Arab Republic (as at Feb. 2014) **b)** United Kingdom of Great Britain and Northern Ireland (previous address) **Listed on:** 30 Sep. 2015 (amended on 24 Nov. 2020) **Other information:** Foreign terrorist fighter with Islamic State in Iraq and the Levant, listed as Al-Qaida in Iraq (QDe.115), in the Syrian Arab Republic. Wanted by the authorities of the United Kingdom. Physical description: hair colour: brown/black. Review pursuant to Security Council resolution 2368 (2017) was concluded on 24 November 2020. INTERPOL-UN Security Council Special Notice web link: https://www.interpol.int/en/How-we-work/Notices/View-UN-Notices-Individuals click here

QDi.358 Name: 1: NASSER 2: AHMED 3: MUTHANA 4: na
Title: na **Designation:** na **DOB:** 29 Apr. 1994 **POB:** Heath, Cardiff, United Kingdom of Great Britain and Northern Ireland **Good quality a.k.a.:** Nasir Muthana **Low quality a.k.a.:** **a)** Abdul Muthana **b)** Abu Muthana **c)** Abu Al-Yemeni Muthana **d)** Abu Muthanna **Nationality:** United Kingdom of Great Britain and Northern Ireland **Passport no:** United Kingdom of Great Britain and Northern Ireland number 210804241, issued on 27 Jul. 2010 (expires on 27 Jul. 2020) **National identification no:** na **Address:** **a)** Syrian Arab Republic (as at Nov. 2013) **b)** United Kingdom of Great Britain and Northern Ireland (previous address until Nov. 2013) **Listed on:** 28 Sep. 2015 (amended on 24 Nov. 2020) **Other information:** Foreign terrorist fighter with Islamic State in Iraq and the Levant (ISIL), listed as Al-Qaida in Iraq (QDe.115), in the Syrian Arab Republic. Wanted by the authorities of the

United Kingdom. Physical description: hair colour: brown/black. Photo available for inclusion in the INTERPOL-UN Security Council Special Notice. Review pursuant to Security Council resolution 2368 (2017) was concluded on 24 November 2020. INTERPOL-UN Security Council Special Notice web link: https://www.interpol.int/en/How-we-work/Notices/View-UN-Notices-Individuals click here

QDi.406 Name: 1: Murad 2: Iraklievich 3: Margoshvili 4: na
Title: na **Designation:** na **DOB:** 15 Jan. 1970 **POB:** Grozny, Chechen Republic, Russian Federation **Good quality a.k.a.: a)** Zurab Iraklievich Margoshvili **b)** Murad Akhmedovich Madayev **c)** Lova Madayev **d)** Abu-Muslim Al-Shishani **Low quality a.k.a.: a)** Muslim **b)** Lava **c)** John **d)** George **e)** Arthur **f)** Sedoy **Nationality: a)** Russian Federation **b)** Georgia **Passport no:** na **National identification no:** na **Address:** na **Listed on:** 20 Jul. 2017 **Other information:** Associated with Jabhat al-Nusrah, listed as Al-Nusrah Front for the People of the Levant (QDe.137).Review pursuant to Security Council resolution 2610 (2021) was concluded on 8 November 2022. INTERPOL-UN Security Council Special Notice web link: https://www.interpol.int/en/How-we-work/Notices/View-UN-Notices-Individuals click here

QDi.427 Name: 1: Noor 2: Wali 3: Mehsud 4: na
Title: Mufti **Designation:** na **DOB:** 26 Jun. 1978 **POB:** Gurguray, Pakistan **Good quality a.k.a.:** Abu Mansoor Asim **Low quality a.k.a.:** na **Nationality:** Pakistan **Passport no:** na **National identification no:** na **Address:** na **Listed on:** 16 Jul. 2020 **Other information:** Leader of Tehrik-e Taliban Pakistan (TTP) (QDe.132). INTERPOL-UN Security Council Special Notice web link: https://www.interpol.int/en/How-we-work/Notices/View-UN-Notices-Individuals click here

QDi.280 Name: 1: TAYEB 2: NAIL 3: na 4: na
Name (original script): الطيب نايل
Title: na **Designation:** na **DOB:** Approximately 1972 **POB:** Faidh El Batma, Djelfa, Algeria **Good quality a.k.a.: a)** Djaafar Abou Mohamed (جعفر ابو محمد) **b)** Abou Mouhadjir (ابو مهاجر) **c)** Mohamed Ould Ahmed Ould Ali (born in 1976) **Low quality a.k.a.:** na **Nationality:** Algeria **Passport no:** na **National identification no:** na **Address:** Mali **Listed on:** 22 Apr. 2010 (amended on 15 Apr. 2014, 6 Dec. 2019) **Other information:** Convicted in absentia by Algerian tribunal on 28 Mar. 1996. Algerian international arrest warrant number 04/09 of 6 Jun. 2009 issued by the Tribunal of Sidi Mhamed, Algiers, Algeria. Algerian extradition request number 2307/09 of 3 Sep. 2009, presented to Malian authorities. Father's name was Benazouz Nail. Mother's name is Belkheiri Oum El Kheir. Member of The Organization of Al-Qaida in the Islamic Maghreb (QDe.014). Review pursuant to Security Council resolution 2368 (2017) was concluded on 4 Dec. 2019. INTERPOL-UN Security Council Special Notice web link: https://www.interpol.int/en/How-we-work/Notices/View-UN-Notices-Individuals click here

QDi.314 Name: 1: ABDERRAHMANE 2: OULD EL AMAR 3: na 4: na
Name (original script): عبد الرحمن ولد العامر
Title: na **Designation:** na **DOB:** Between 1977 and 1982 **POB:** Tabankort, Mali **Good quality a.k.a.: a)** Ahmed el Tilemsi **b)** Abderrahmane Ould el Amar Ould Sidahmed Loukbeiti **c)** Ahmad Ould Amar **Low quality a.k.a.:** na **Nationality:** Mali **Passport no:** na **National identification no:** na **Address: a)** Gao, Mali **b)** Tabankort, Mali **c)** In Khalil, Mali **d)** Al Moustarat, Mali **Listed on:** 22 Feb. 2013 (amended on 1 May 2019, 2 Feb. 2023) **Other information:** Reportedly deceased as of December 2014. Leader of the Mouvement pour l'Unification et le Jihad en Afrique de l'Ouest (MUJAO) (QDe.134). Member of The Organization of Al-Qaida in the Islamic Maghreb (QDe.014). Arrested in April 2005 in Mauritania, escaped from Nouakchott jail on 26 Apr. 2006. Re-arrested in Sep. 2008 in Mali and released on 15 Apr. 2009. Associated with Mokhtar Belmokhtar (QDi.136). Father's name is Leewemere. Review pursuant to Security Council resolution 2253 (2015) was concluded on 21 Feb. 2019. INTERPOL-UN Security Council Special Notice web link: https://www.interpol.int/en/How-we-work/Notices/View-UN-Notices-Individuals click here

QDi.315 Name: 1: HAMADA 2: OULD MOHAMED EL KHAIRY 3: na 4: na

Name (original script): حماده ولد محمد الخيري

Title: na **Designation:** na **DOB:** 1970 **POB:** Nouakchott, Mauritania **Good quality a.k.a.: a)** Hamada Ould Mohamed Lemine Ould Mohamed el Khairy **b)** Ould Kheirou **c)** Hamad el Khairy **Low quality a.k.a.:** Abou QumQum **Nationality: a)** Mauritania **b)** Mali **Passport no:** Mali number A1447120 (expired on 19 Oct. 2011) **National identification no:** na **Address:** Gao, Mali **Listed on:** 22 Feb. 2013 (amended on 1 May 2019) **Other information:** Leader of the Mouvement pour l' Unification et le Jihad en Afrique de l' Ouest (MUJAO) (QDe.134). Has provided logistical support to the Sahelian group Al Moulathamine, linked with the Organization of Al-Qaida in the Islamic Maghreb (QDe.014). International arrest warrant issued by Mauritania. Mother' s name is Tijal Bint Mohamed Dadda. Review pursuant to Security Council resolution 2253 (2015) was concluded on 21 Feb. 2019.Review pursuant to Security Council resolution 2610 (2021) was concluded on 8 November 2022. INTERPOL-UN Security Council Special Notice web link: https://www.interpol.int/en/How-we-work/Notices/View-UN-Notices-Individuals click here

QDi.298 Name: 1: ABD AL-RAHMAN 2: OULD MUHAMMAD AL-HUSAYN 3: OULD MUHAMMAD SALIM 4: na

Name (original script): عبد الرحمن ولد محمد الحسين ولد محمد سليم

Title: na **Designation:** na **DOB:** Approximately 1981 **POB:** Saudi Arabia **Good quality a.k.a.: a)** Abdarrahmane ould Mohamed el Houcein ould Mohamed Salem **b)** شيخ يونس الموريتاني (Yunis al-Mauritani; Younis al-Mauritani; Sheikh Yunis al-Mauritani; Shaykh Yunis the Mauritanian) **Low quality a.k.a.: a)** Salih the Mauritanian **b)** Mohamed Salem **c)** Youssef Ould Abdel Jelil **d)** El Hadj Ould Abdel Ghader **e)** Abdel Khader **f)** Abou Souleimane **g)** Chingheity **Nationality:** Mauritania **Passport no:** na **National identification no:** na **Address:** na **Listed on:** 15 Sep. 2011 (amended on 24 Nov. 2020) **Other information:** Pakistan-based senior Al-Qaida (QDe.004) leader also associated with The Organization of Al-Qaida in the Islamic Maghreb (QDe.014). Wanted by Mauritanian authorities. Review pursuant to Security Council resolution 2368 (2017) was concluded on 24 November 2020. INTERPOL-UN Security Council Special Notice web link: https://www.interpol.int/en/How-we-work/Notices/View-UN-Notices-Individuals click here

QDi.353 Name: 1: ALI 2: BEN TAHER 3: BEN FALEH 4: OUNI HARZI

Name (original script): علي بن الطاهر بن الفالح العوني الحرزي

Title: na **Designation:** na **DOB:** 9 Mar. 1986 **POB:** Ariana, Tunisia **Good quality a.k.a.:** na **Low quality a.k.a.:** Abou Zoubair **Nationality:** Tunisia **Passport no:** Tunisian passport number W342058, issued on 14 Mar. 2011 (expires on 13 Mar 2016) **National identification no:** Tunisia National Identity Card number 08705184, issued on 24 Feb. 2011 **Address: a)** 18 Mediterranean Street, Ariana, Tunisia **b)** Syrian Arab Republic (located in as at Mar. 2015) **c)** Iraq (possible alternative location as at Mar. 2015) **d)** Libya (previously located in) **Listed on:** 10 Apr. 2015 (amended on 14 Sep. 2016, 1 May 2019) **Other information:** Physical description: eye colour: brown; height: 171cm. Photo available for inclusion in the INTERPOL-UN Security Council Special Notice. Previous occupation: trading agent. A member of Ansar al-Shari' a in Tunisia (QDe.143), active in recruitment of foreign terrorist fighters and arms smuggling. Detained and sentenced to 30 months imprisonment for planning terrorist acts in 2005 in Tunisia. Planned and perpetrated the attack against the Consulate of the United States in Benghazi, Libya on 11 Sep. 2012. Arrest warrant issued by the Tunisian National Guard (as at Mar. 2015). Father' s name is Taher Ouni Harzi, mother' s name is Borkana Bedairia. Reportedly killed in an airstrike in Mosul, Iraq, in Jun. 2015. Review pursuant to Security Council resolution 2253 (2015) was concluded on 21 Feb. 2019. Review pursuant to Security Council resolution 2368 (2017) was concluded on 15 November 2021. INTERPOL-UN Security Council Special Notice web link: https://www.interpol.int/en/How-we-work/Notices/View-UN-Notices-Individuals click here

QDi.354 Name: 1: TARAK 2: BEN TAHER 3: BEN FALEH 4: OUNI HARZI

Name (original script): طارق بن الطاهر بن الفالح العوني الحرزي

Title: na **Designation:** na **DOB:** 3 May 1982 **POB:** Tunis, Tunisia **Good quality a.k.a.:** na **Low quality a.k.a.:** Abou Omar Al Tounisi **Nationality:** Tunisia **Passport no:** Tunisia number Z050399,

issued on 9 Dec. 2003 (expired on 8 Dec. 2008) **National identification no:** Tunisia National Identification Number 04711809, issued on 13 Nov. 2003 **Address: a)** 18 Mediterranean Street, Ariana, Tunisia **b)** Syrian Arab Republic (located in as at Mar. 2015) **c)** Iraq (possible alternative location as at Mar. 2015) **d)** Libya (previously located in) **Listed on:** 10 Apr. 2015 (amended on 14 Sep. 2016, 1 May 2019) **Other information:** Physical description: eye colour: brown; height: 172cm. Photo available for inclusion in the INTERPOL-UN Security Council Special Notice. Previous occupation: worker. A dangerous and active member of Al Qaida in Iraq (QDe.115) in 2004, also active in facilitating and hosting members of Ansar al-Shari' a in Tunisia (QDe.143) in Syria. Sentenced, in absentia, on 30 October 2007, to 24 years imprisonment for terrorist activities by the Appeals Court of Tunis. Father' s name is Taher Ouni Harzi, mother' s name is Borkana Bedairia. Reportedly killed in Syria in Jun. 2015. Review pursuant to Security Council resolution 2253 (2015) was concluded on 21 Feb. 2019. Review pursuant to Security Council resolution 2368 (2017) was concluded on 15 November 2021. INTERPOL-UN Security Council Special Notice web link: https://www.interpol.int/en/How-we-work/Notices/View-UN-Notices-Individuals click here

QDi.242 Name: 1: DINNO AMOR 2: ROSALEJOS 3: PAREJA 4: na
Title: na **Designation:** na **DOB:** 19 Jul. 1981 **POB:** Cebu City, Philippines **Good quality a.k.a.: a)** Johnny Pareja **b)** Khalil Pareja **Low quality a.k.a.: a)** Mohammad **b)** Akmad **c)** Mighty **d)** Rash **Nationality:** Philippines **Passport no:** na **National identification no:** na **Address:** Atimonana, Quezon Province, Philippines **Listed on:** 4 Jun. 2008 (amended on 3 Jun. 2009, 13 Dec. 2011, 6 Dec. 2019) **Other information:** Member of the Rajah Solaiman Movement (QDe.128). Father's name is Amorsolo Jarabata Pareja. Mother's name is Leonila Cambaya Rosalejos. Review pursuant to Security Council resolution 1822 (2008) was concluded on 13 May 2010. Review pursuant to Security Council resolution 2368 (2017) was concluded on 4 Dec. 2019. INTERPOL-UN Security Council Special Notice web link: https://www.interpol.int/en/How-we-work/Notices/View-UN-Notices-Individuals click here

QDi.294 Name: 1: UMAR 2: PATEK 3: na 4: na
Title: na **Designation:** na **DOB:** 20 Jul. 1966 **POB:** Central Java, Indonesia **Good quality a.k.a.: a)** Omar Patek **b)** Mike Arsalan **c)** Hisyam Bin Zein **d)** Anis Alawi Jafar **Low quality a.k.a.: a)** Pa'tek **b)** Pak Taek **c)** Umar Kecil **d)** Al Abu Syekh Al Zacky **e)** Umangis Mike **Nationality:** Indonesia **Passport no:** na **National identification no:** na **Address:** Indonesia **Listed on:** 19 Jul. 2011 (amended on 23 Feb. 2012, 22 Sep. 2017) **Other information:** Senior member of Jemaah Islamiyah (QDe.092) involved in planning and funding multiple terrorist attacks in the Philippines and Indonesia. Provided training to Abu Sayyaf Group (QDe.001). Convicted for his role in the 2002 Bali bombings and sentenced to 20 years in prison in Jun. 2012. Remains in custody in Indonesia as at May 2015. Review pursuant to Security Council resolution 2368 (2017) was concluded on 15 November 2021. Photos included in INTERPOL-UN Security Council Special Notice web link: https://www.interpol.int/en/How-we-work/Notices/View-UN-Notices-Individuals click here

QDi.348 Name: 1: ANGGA 2: DIMAS 3: PERSHADA 4: na
Title: Secretary General (as at mid-2014) **Designation:** na **DOB:** 4 Mar. 1985 **POB:** Jakarta, Indonesia **Good quality a.k.a.: a)** Angga Dimas Persada born 4 Mar. 1985 in Jakarta, Indonesia **b)** Angga Dimas Persadha born 4 Mar. 1985 in Jakarta, Indonesia **c)** Angga Dimas Prasondha born 4 Mar. 1985 in Jakarta, Indonesia **Low quality a.k.a.:** na **Nationality:** Indonesia **Passport no:** Indonesian passport number W344982 (issued under name Angga Dimas Peshada, born 4 Mar.1985 in Jakarta, Indonesia) **National identification no:** na **Address:** na **Listed on:** 13 Mar. 2015 (amended on 24 Nov. 2020) **Other information:** Member of Jemaah Islamiyah (QDe.092) and leader of Hilal Ahmar Society Indonesia (HASI) (QDe.147). Review pursuant to Security Council resolution 2368 (2017) was concluded on 24 November 2020. INTERPOL-UN Security Council Special Notice web link: https://www.interpol.int/en/How-we-work/Notices/View-UN-Notices-Individuals click here

QDi.271 Name: 1: ARIF 2: QASMANI 3: na 4: na

Title: na **Designation:** na **DOB:** Approximately 1944 **POB:** Pakistan **Good quality a.k.a.: a)** Muhammad Arif Qasmani **b)** Muhammad 'Arif Qasmani **c)** Mohammad Arif Qasmani **d)** Arif Umer **e)** Qasmani Baba **f)** Memon Baba **g)** Baba Ji **Low quality a.k.a.:** na **Nationality:** Pakistan **Passport no:** na **National identification no:** na **Address:** House Number 136, KDA Scheme No. 1, Tipu Sultan Road, Karachi, Pakistan **Listed on:** 29 Jun. 2009 (amended on 1 May 2019) **Other information:** Associated with Lashkar-e-Tayyiba (QDe.118) and Al-Qaida (QDe.004). In detention as at June 2009. Review pursuant to Security Council resolution 2253 (2015) was concluded on 21 Feb. 2019. Review pursuant to Security Council resolution 2610 (2021) was concluded on 8 November 2022. INTERPOL-UN Security Council Special Notice web link: https://www.interpol.int/en/How-we-work/Notices/View-UN-Notices-Individuals click here

QDi.303 Name: 1: FAZAL 2: RAHIM 3: na 4: na
Name (original script): فضل رحيم
Title: na **Designation:** na **DOB: a)** 5 Jan. 1974 **b)** 1977 **c)** 1975 **d)** 24 Jan. 1973 **POB:** Kabul, Afghanistan **Good quality a.k.a.: a)** Fazel Rahim; Fazil Rahim **b)** Fazil Rahman **Low quality a.k.a.:** na **Nationality:** Afghanistan **Passport no:** Afghanistan number R512768 **National identification no:** na **Address: a)** (Afghanistan/Pakistan border region (previous address)) **b)** (A2, City Computer Plaza, Shar-e-Now, Kabul, Afghanistan (previous address)) **c)** Microrayan 3rd, Apt. 45, block 21, Kabul, Afghanistan (previous address) **Listed on:** 6 Mar. 2012 **Other information:** Was a financial facilitator for the Islamic Movement of Uzbekistan (QDe.010) and Al-Qaida (QDe.004). Was associated with Tohir Abdulkhalilovich Yuldashev. As of late 2010, in custody of Pakistan authorities. Father's name is Fazal Ahmad. Review pursuant to Security Council resolution 2368 (2017) was concluded on 15 November 2021. INTERPOL-UN Security Council Special Notice web link: https://www.interpol.int/en/How-we-work/Notices/View-UN-Notices-Individuals click here

QDi.075 Name: 1: ABDELHALIM 2: HAFED 3: ABDELFATTAH 4: REMADNA
Name (original script): عبدالحليم حافظ عبدالفتاح رمادنا
Title: na **Designation:** na **DOB:** 2 Apr. 1966 **POB:** Biskra, Algeria **Good quality a.k.a.:** Abdelhalim Remadna **Low quality a.k.a.:** Jalloul **Nationality:** Algeria **Passport no:** na **National identification no:** na **Address:** Algeria **Listed on:** 3 Sep. 2002 (amended on 12 Apr. 2006, 7 Apr. 2008, 3 Jun. 2009, 25 Jan. 2010, 1 May 2019, 23 Dec. 2010) **Other information:** Deported from Italy to Algeria on 12 Aug. 2006. Review pursuant to Security Council resolution 1822 (2008) was concluded on 8 Dec. 2009. Review pursuant to Security Council resolution 2253 (2015) was concluded on 21 Feb. 2019. Review pursuant to Security Council resolution 2610 (2021) was concluded on 8 November 2022. INTERPOL-UN Security Council Special Notice web link: https://www.interpol.int/en/How-we-work/Notices/View-UN-Notices-Individuals click here

QDi.219 Name: 1: TAUFIK 2: RIFKI 3: na 4: na
Title: na **Designation:** na **DOB:** 19 Aug. 1974 **POB:** Dacusuman Surakarta, Central Java, Indonesia **Good quality a.k.a.: a)** Refke, Taufek **b)** Rifqi, Taufik **c)** Rifqi, Tawfiq **d)** Ami Iraq **e)** Ami Irza **f)** Amy Erja **g)** Ammy Erza **h)** Ammy Izza **i)** Ami Kusoman **j)** Abu Obaida **k)** Abu Obaidah **l)** Abu Obeida **m)** Abu Ubaidah **n)** Obaidah **o)** Abu Obayda **p)** Izza Kusoman **q)** Yacub, Eric **Low quality a.k.a.:** na **Nationality:** Indonesia **Passport no:** na **National identification no:** na **Address:** Philippines **Listed on:** 21 Apr. 2006 (amended on 13 Dec. 2011, 24 Nov. 2020) **Other information:** In detention in the Philippines as at May 2011. Review pursuant to Security Council resolution 1822 (2008) was concluded on 8 Jun. 2010. Review pursuant to Security Council resolution 2368 (2017) was concluded on 24 November 2020. INTERPOL-UN Security Council Special Notice web link: https://www.interpol.int/en/How-we-work/Notices/View-UN-Notices-Individuals click here

QDi.407 Name: 1: OMAN 2: ROCHMAN 3: na 4: na
Title: Ustadz **Designation:** na **DOB:** 5 Jan. 1972 **POB:** Sumedang, Indonesia **Good quality a.k.a.: a)** Oman Rahman **b)** Abu Sulaiman Aman Abdurrahman Al-Arkhabiliy **c)** Aman Abdul Rahman **d)** Aman Abdurahman **e)** Aman Abdurrachman **f)** Oman Abdulrohman **g)** Oman Abdurrahman **h)** Aman Abdurrahman **Low quality a.k.a.:** na **Nationality:** Indonesia **Passport no:** na **National**

identification no: na **Address:** Pasir Putih Prison, Nusa Kambangan Island, Indonesia **Listed on:** 20 Jul. 2017 (amended on 2 Feb. 2023) **Other information:** De facto leader for all Islamic State in Iraq and the Levant, listed as Al-Qaida in Iraq (QDe.115), supporters in Indonesia, despite his incarceration in Indonesia since December 2010. Sentenced to death by the Indonesian Supreme Court. INTERPOL-UN Security Council Special Notice web link: https://www.interpol.int/en/How-we-work/Notices/View-UN-Notices-Individuals click here

QDi.150 Name: 1: AL-AZHAR 2: BEN KHALIFA 3: BEN AHMED 4: ROUINE
Name (original script): الأزهر بن خليفة بن احمد روين
Title: na **Designation:** na **DOB:** 20 Nov. 1975 **POB:** Sfax, Tunisia **Good quality a.k.a.:** na **Low quality a.k.a.:** **a)** Salmane **b)** Lazhar **Nationality:** Tunisia **Passport no:** Tunisia number P182583, issued on 13 Sep. 2003 (expired on 12 Sep. 2007) **National identification no:** 05258253 **Address:** No.2 89th Street Zehrouni, Tunis, Tunisia **Listed on:** 12 Nov. 2003 (amended on 20 Dec. 2005, 21 Dec. 2007, 30 Jan. 2009, 16 May 2011, 23 Feb. 2016, 24 Nov. 2020) **Other information:** Sentenced to six years and ten months of imprisonment for membership of a terrorist association by the Appeal Court of Milan, Italy, on 7 Feb. 2008. Imprisoned in Sfax Prison on 5 June 2007 pursuant to an order issued by the Appeals Tribunal in Tunisia for joining an organization linked to terrorist crimes (case No.9301/207). Sentenced to two years and 15 days' imprisonment and released on 18 June 2008.U Considered a fugitive from justice by the Italian authorities as at Jul. 2008. Under administrative control measure in Tunisia as at 2010. Review pursuant to Security Council resolution 1822 (2008) was concluded on 21 Jun. 2010. Review pursuant to Security Council resolution 2368 (2017) was concluded on 24 November 2020. INTERPOL-UN Security Council Special Notice web link: https://www.interpol.int/en/How-we-work/Notices/View-UN-Notices-Individuals click here

QDi.186 Name: 1: ABU 2: RUSDAN 3: na 4: na
Title: na **Designation:** na **DOB:** 16 Aug. 1960 **POB:** Kudus, Central Java, Indonesia **Good quality a.k.a.:** na **Low quality a.k.a.:** **a)** Abu Thoriq **b)** Rusdjan **c)** Rusjan **d)** Rusydan **e)** Thoriquddin **f)** Thoriquiddin **g)** Thoriquidin **h)** Toriquddin **Nationality:** na **Passport no:** na **National identification no:** Indonesia 1608600001 **Address:** na **Listed on:** 16 May 2005 (amended on 12 Dec. 2014, 1 May 2019, 2 Feb. 2023) **Other information:** Arrested in Indonesia in 2021. Was the acting Jemaah Islamiyah (JI, QDe.092) emir before his arrest, and remains a senior figure of JI. Review pursuant to Security Council resolution 1822 (2008) was concluded on 8 Jun. 2010. Review pursuant to Security Council resolution 2253 (2015) was concluded on 21 Feb. 2019. INTERPOL-UN Security Council Special Notice web link: https://www.interpol.int/en/How-we-work/Notices/View-UN-Notices-Individuals click here

QDi.403 Name: 1: FARED 2: SAAL 3: na 4: na
Title: na **Designation:** na **DOB:** 18 Feb. 1989 **POB:** Bonn, Germany **Good quality a.k.a.:** na **Low quality a.k.a.:** **a)** Abu Luqmaan Al Almani **b)** Abu Lugmaan **Nationality:** **a)** Germany **b)** Algeria **Passport no:** na **National identification no:** Germany national identity card number 5802098444, issued in Bonn, Germany (on 15 Apr. 2010, expired on 14 Apr. 2016) **Address:** na **Listed on:** 16 Jun. 2017 **Other information:** German foreign terrorist fighter for Islamic State in Iraq and the Levant, listed as Al-Qaida in Iraq (QDe.115). Physical description: eye colour: brown; hair colour: black; height: 178cm; weight: 80kg. European arrest warrant issued by the investigating judge of the German Federal Supreme Court on 13 Aug. 2014. Review pursuant to Security Council resolution 2610 (2021) was concluded on 8 November 2022. INTERPOL-UN Security Council Special Notice web link: https://www.interpol.int/en/How-we-work/Notices/View-UN-Notices-Individuals click here

QDi.002 Name: 1: AMIN 2: MUHAMMAD 3: UL HAQ 4: SAAM KHAN
Title: na **Designation:** na **DOB:** 1960 **POB:** Nangarhar Province, Afghanistan **Good quality a.k.a.:** **a)** Al-Haq, Amin **b)** Amin, Muhammad **Low quality a.k.a.:** **a)** Dr. Amin **b)** Ul-Haq, Dr. Amin **Nationality:** Afghanistan **Passport no:** na **National identification no:** na **Address:** na **Listed on:** 25 Jan. 2001 (amended on 18 Jul. 2007, 16 Dec. 2010, 6 Dec. 2019, 2 Feb. 2023) **Other**

information: Security coordinator for Usama bin Laden (deceased). Repatriated to Afghanistan in February 2006. He was in Afghanistan as of August 2021. Review pursuant to Security Council resolution 1822 (2008) was concluded on 15 Jun. 2010. Review pursuant to Security Council resolution 2368 (2017) was concluded on 4 Dec. 2019 INTERPOL-UN Security Council Special Notice web link: https://www.interpol.int/en/How-we-work/Notices/View-UN-Notices-Individuals click here

QDi.020 Name: 1: MOHAMMAD 2: HAMDI 3: MOHAMMAD 4: SADIQ AL-AHDAL
Name (original script): محمد حمدي محمد صادق الأهدل
Title: na **Designation:** na **DOB:** 19 Nov. 1971 **POB:** Medina, Saudi Arabia **Good quality a.k.a.: a)** Al-Hamati, Muhammad **b)** Muhammad Muhammad Abdullah Al-Ahdal **c)** Mohamed Mohamed Abdullah Al-Ahdal **Low quality a.k.a.: a)** Abu Asim Al-Makki **b)** Ahmed **Nationality:** Yemen **Passport no:** Yemen number 541939, issued on 31 Jul. 2000, issued in Al-Hudaydah, Yemen (in the name of Muhammad Muhammad Abdullah Al-Ahdal) **National identification no:** Yemeni identity card number 216040 **Address:** Jamal street, Al-Dahima alley, Al-Hudaydah, Yemen **Listed on:** 17 Oct. 2001 (amended on 30 Jan. 2009, 25 Jan. 2010, 6 Dec. 2019) **Other information:** Responsible for the finances of Al-Qa' ida (QDe.004) in Yemen. Accused of involvement in the attack on the USS Cole in 2000. Arrested in Yemen in Nov. 2003. Sentenced to three years and one month of imprisonment by the specialized criminal court of first instance in Yemen. Released on 25 Dec. 2006 after the completion of his sentence. Review pursuant to Security Council resolution 1822 (2008) was concluded on 8 Jul. 2010. Review pursuant to Security Council resolution 2368 (2017) was concluded on 4 Dec. 2019 INTERPOL-UN Security Council Special Notice web link: https://www.interpol.int/en/How-we-work/Notices/View-UN-Notices-Individuals click here

QDi.263 Name: 1: HAFIZ 2: MUHAMMAD 3: SAEED 4: na
Title: na **Designation:** na **DOB:** 5 Jun. 1950 **POB:** Sargodha, Punjab, Pakistan **Good quality a.k.a.: a)** Hafiz Mohammad Sahib **b)** Hafiz Mohammad Sayid **c)** Hafiz Muhammad **d)** Hafiz Saeed **e)** Hafez Mohammad Saeed **f)** Hafiz Mohammad Sayeed **g)** Tata Mohammad Syeed **h)** Mohammad Sayed **i)** Muhammad Saeed **Low quality a.k.a.:** Hafiz Ji **Nationality:** Pakistan **Passport no:** na **National identification no:** Pakistan 3520025509842-7 **Address:** House No. 116E, Mohalla Johar, Lahore, Tehsil, Lahore City, Lahore District, Pakistan (location as at May 2008) **Listed on:** 10 Dec. 2008 (amended on 17 Jul. 2009) **Other information:** Muhammad Saeed is the leader of Lashkar-e-Tayyiba (QDe.118). INTERPOL-UN Security Council Special Notice web link: https://www.interpol.int/en/How-we-work/Notices/View-UN-Notices-Individuals click here

QDi.208 Name: 1: RADULAN 2: SAHIRON 3: na 4: na
Title: na **Designation:** na **DOB:** 1955 **POB:** Kaunayan, Patikul, Jolo Island, Philippines **Good quality a.k.a.: a)** Radullan Sahiron **b)** Radulan Sahirun **c)** Radulan Sajirun **Low quality a.k.a.:** Commander Putol **Nationality:** Philippines **Passport no:** na **National identification no:** na **Address:** Sulu region, Philippines (reported location) **Listed on:** 6 Dec. 2005 (amended on 22 Sep. 2017, 24 Nov. 2020) **Other information:** Physical description: eye colour: black; hair colour: gray; height: 5 feet 6 inches – 168 cm; weight: 140 pounds – 64 kg; build: slight; right arm is amputated above his elbow. Review pursuant to Security Council resolution 1822 (2008) was concluded on 8 Jun. 2010. Wanted by the Philippines authorities for terrorist offences and by authorities of the United States of America for involvement in the kidnapping of its national. Photos included in. Review pursuant to Security Council resolution 2368 (2017) was concluded on 24 November 2020. INTERPOL-UN Security Council Special Notice web link: https://www.interpol.int/en/How-we-work/Notices/View-UN-Notices-Individuals click here

QDi.222 Name: 1: NESSIM 2: BEN ROMDHANE 3: SAHRAOUI 4: na
Name (original script): نسيم بن رمضان صحراوي
Title: na **Designation:** na **DOB:** 3 Aug. 1973 **POB:** Bizerta, Tunisia **Good quality a.k.a.: a)** Dass **b)** Nasim al-Sahrawi **Low quality a.k.a.:** na **Nationality:** Tunisia **Passport no:** na **National identification no:** na **Address:** Tunisia **Listed on:** 2 Aug. 2006 (amended on 1 Sep. 2009, 25 Jan. 2010, 13 Dec. 2011, 24 Nov. 2020) **Other information:** Considered a fugitive from justice by the

Italian authorities and sentenced in absentia to 6 years detention on 20 Nov. 2008. Sentenced in Tunisia to 4 years imprisonment for terrorist activity and in detention in Tunisia as at Jun. 2009. Review pursuant to Security Council resolution 1822 (2008) was concluded on 20 Jul. 2009. Review pursuant to Security Council resolution 2368 (2017) was concluded on 24 November 2020. INTERPOL-UN Security Council Special Notice web link: https://www.interpol.int/en/How-we-work/Notices/View-UN-Notices-Individuals click here

QDi.387 Name: 1: MOHAMMED 2: ABDEL-HALIM 3: HEMAIDA 4: SALEH
Title: na **Designation:** na **DOB:** **a)** 22 Sep. 1988 **b)** 22 Sep. 1989 **POB:** Alexandria, Egypt **Good quality a.k.a.:** **a)** Muhammad Hameida Saleh **b)** Muhammad Abd-al-Halim Humaydah **c)** Faris Baluchistan **Low quality a.k.a.:** na **Nationality:** Egypt **Passport no:** na **National identification no:** na **Address:** Egypt **Listed on:** 29 Feb. 2016 **Other information:** Member of Al-Qaida (QDe.004). Involved in recruiting suicide bombers to go to Syrian Arab Republic and planning terrorist activities against targets in Europe. Arrested in Cairo, Egypt in 2013. Review pursuant to Security Council resolution 2368 (2017) was concluded on 15 November 2021. INTERPOL-UN Security Council Special Notice web link: https://www.interpol.int/en/How-we-work/Notices/View-UN-Notices-Individuals click here

QDi.244 Name: 1: HILARION 2: DEL ROSARIO 3: SANTOS 4: na
Title: "Amir" **Designation:** na **DOB:** 12 Mar. 1966 **POB:** 686 A. Mabini Street, Sangandaan, Caloocan City, Philippines **Good quality a.k.a.:** **a)** Akmad Santos **b)** Ahmed Islam **c)** Ahmad Islam Santos **d)** Hilarion Santos, III (third) **e)** Hilarion Del Rosario Santos, III (third) **f)** Abu Abdullah Santos **g)** Faisal Santos **Low quality a.k.a.:** **a)** Lakay **b)** Aki **c)** Aqi **d)** Abu Hamsa **Nationality:** Philippines **Passport no:** Philippines number AA780554 **National identification no:** na **Address:** 50, Purdue Street, Cubao, Quezon City, Philippines **Listed on:** 4 Jun. 2008 (amended on 13 Dec. 2011, 25 Oct. 2016, 22 Sep. 2017, 24 Nov. 2020) **Other information:** Founder and leader of the Rajah Solaiman Movement (QDe.128) and linked to the Abu Sayyaf Group (QDe.001). In detention in the Philippines as of May 2011. Review pursuant to Security Council resolution 1822 (2008) was concluded on 13 May 2010. Photos included in Review pursuant to Security Council resolution 2368 (2017) was concluded on 24 November 2020. INTERPOL-UN Security Council Special Notice web link: https://www.interpol.int/en/How-we-work/Notices/View-UN-Notices-Individuals click here

QDi.350 Name: 1: WIJI 2: JOKO 3: SANTOSO 4: na
Title: na **Designation:** na **DOB:** 14 Jul. 1975 **POB:** Rembang, Jawa Tengah, Indonesia **Good quality a.k.a.:** Wijijoko Santoso born 14 Jul. 1975 in Rembang, Jawa Tengah, Indonesia **Low quality a.k.a.:** **a)** Abu Seif al-Jawi **b)** Abu Seif **Nationality:** Indonesia **Passport no:** Indonesia number A2823222, issued on 28 May 2012 (expires 28 May 2017, issued under name Wiji Joko Santoso, born 14 Jul. 1975 in Rembang, Jawa Tengah, Indonesia) **National identification no:** na **Address:** na **Listed on:** 13 Mar. 2015 (amended on 24 Nov. 2020) **Other information:** Head of the foreign affairs division and key outreach player of Jemaah Islamiyah (QDe.092). Associated with Hilal Ahmar Society Indonesia (HASI) (QDe.147). Review pursuant to Security Council resolution 2368 (2017) was concluded on 24 November 2020. INTERPOL-UN Security Council Special Notice web link: https://www.interpol.int/en/How-we-work/Notices/View-UN-Notices-Individuals click here

QDi.322 Name: 1: ABUBAKAR 2: MOHAMMED 3: SHEKAU 4: na
Name (original script): أبو بكر محمد الشكوى
Title: na **Designation:** na **DOB:** 1969 **POB:** Shekau Village, Yobe State, Nigeria **Good quality a.k.a.:** Abubakar Shekau **Low quality a.k.a.:** **a)** Abu Mohammed Abubakar bin Mohammed **b)** Abu Muhammed Abubakar bi Mohammed **c)** Shekau **d)** Shehu **e)** Shayku **f)** Imam Darul Tauhid **g)** Imam Darul Tawheed **Nationality:** Nigeria **Passport no:** na **National identification no:** na **Address:** Nigeria **Listed on:** 26 Jun. 2014 (amended on 6 Dec. 2019) **Other information:** Member of the Kanuri tribe. Physical description: eye colour: black; hair colour: black. Photo available for inclusion in the INTERPOL-UN Security Council Special Notice. Leader of Jama'atu Ahlis Sunna Lidda'Awati Wal-Jihad (Boko Haram) (QDe.138). Under Shekau’ s leadership, Boko Haram has been responsible

for a series of major terrorist attacks. Review pursuant to Security Council resolution 2368 (2017) was concluded on 4 Dec. 2019 INTERPOL-UN Security Council Special Notice web link: https://www.interpol.int/en/How-we-work/Notices/View-UN-Notices-Individuals click here

QDi.017 Name: 1: THARWAT 2: SALAH 3: SHIHATA 4: na
Name (original script): ثروت صالح شحاته
Title: na **Designation:** na **DOB:** 29 Jun. 1960 **POB:** Egypt **Good quality a.k.a.: a)** Tarwat Salah Abdallah **b)** Salah Shihata Thirwat **c)** Shahata Thirwat **d)** Tharwat Salah Shihata Ali (previously listed as) **Low quality a.k.a.:** na **Nationality:** Egypt **Passport no:** na **National identification no:** na **Address:** na **Listed on:** 6 Oct. 2001 (amended on 26 Nov. 2004, 16 Dec. 2010, 6 Dec. 2019) **Other information:** Review pursuant to Security Council resolution 1822 (2008) was concluded on 15 Jun. 2010. Review pursuant to Security Council resolution 2368 (2017) was concluded on 4 Dec. 2019. Review pursuant to Security Council resolution 2610 (2021) was concluded on 8 November 2022. INTERPOL-UN Security Council Special Notice web link: https://www.interpol.int/en/How-we-work/Notices/View-UN-Notices-Individuals click here

QDi.122 Name: 1: PARLINDUNGAN 2: SIREGAR 3: na 4: na
Title: na **Designation:** na **DOB: a)** 25 Apr. 1957 **b)** 25 Apr. 1967 **POB:** Indonesia **Good quality a.k.a.: a)** Siregar, Parlin **b)** Siregar, Saleh Parlindungan **Low quality a.k.a.:** na **Nationality:** Indonesia **Passport no:** na **National identification no:** na **Address:** na **Listed on:** 9 Sep. 2003 (amended on 6 Dec. 2019) **Other information:** Review pursuant to Security Council resolution 1822 (2008) was concluded on 25 May 2010. Review pursuant to Security Council resolution 2368 (2017) was concluded on 4 Dec. 2019. Review pursuant to Security Council resolution 2610 (2021) was concluded on 8 November 2022. INTERPOL-UN Security Council Special Notice web link: https://www.interpol.int/en/How-we-work/Notices/View-UN-Notices-Individuals click here

QDi.124 Name: 1: YAZID 2: SUFAAT 3: na 4: na
Title: na **Designation:** na **DOB:** 20 Jan. 1964 **POB:** Johor, Malaysia **Good quality a.k.a.:** na **Low quality a.k.a.: a)** Joe **b)** Abu Zufar **Nationality:** Malaysia **Passport no:** A 10472263 **National identification no:** 640120-01-5529 **Address: a)** Taman Bukit Ampang, State of Selangor, Malaysia (previous address) **b)** Malaysia (in prison since 2013) **Listed on:** 9 Sep. 2003 (amended on 3 May 2004, 1 Feb. 2008, 10 Aug. 2009, 25 Jan. 2010, 16 May 2011, 11 Oct. 2016, 22 Sep. 2017, 1 May 2019) **Other information:** Founding member of Jemaah Islamiyah (JI) (QDe.092) who worked on Al-Qaida' s (QDe.004) biological weapons program, provided support to those involved in Al-Qaida' s 11 Sep. 2001 attacks in the United States of America, and was involved in JI bombing operations. Detained in Malaysia from 2001 to 2008. Arrested in Malaysia in 2013 and sentenced to 7 years in Jan. 2016 for failing to report information relating to terrorist acts. Due for release in Feb. 2020. Review pursuant to Security Council resolution 1989 (2011) was concluded on 6 Mar. 2014. Photos included in INTERPOL-UN Security Council Special Notice web link: https://www.interpol.int/en/notice/search/un/1424794. Review pursuant to Security Council resolution 2253 (2015) was concluded on 21 Feb. 2019. INTERPOL-UN Security Council Special Notice web link: https://www.interpol.int/en/How-we-work/Notices/View-UN-Notices-Individuals click here

QDi.349 Name: 1: BAMBANG 2: SUKIRNO 3: na 4: na
Title: na **Designation:** na **DOB:** 5 Apr. 1975 **POB:** Indonesia **Good quality a.k.a.:** na **Low quality a.k.a.: a)** Pak Zahra **b)** Abu Zahra **Nationality:** Indonesia **Passport no:** Indonesia number A2062513 **National identification no:** na **Address:** na **Listed on:** 13 Mar. 2015 (amended on 24 Nov. 2020) **Other information:** A senior leader of Jemaah Islamiyah (QDe.092) who has held leadership positions in Hilal Ahmar Society Indonesia (HASI) (QDe.147). Review pursuant to Security Council resolution 2368 (2017) was concluded on 24 November 2020. INTERPOL-UN Security Council Special Notice web link: https://www.interpol.int/en/How-we-work/Notices/View-UN-Notices-Individuals click here

QDi.187 Name: 1: ARIS 2: SUMARSONO 3: na 4: na
Title: na **Designation:** na **DOB:** 19 Apr. 1963 **POB:** Gebang village, Masaran, Sragen, Central Java, Indonesia **Good quality a.k.a.: a)** Zulkarnan **b)** Zulkarnain **c)** Zulkarnin **d)** Arif Sunarso **e)** Zulkarnaen **f)** Aris Sunarso **g)** Ustad Daud Zulkarnaen **Low quality a.k.a.: a)** Murshid **b)** Daud **c)** Pak Ud **d)** Mbah Zul **e)** Zainal Arifin **f)** Zul **g)** Abdullah Abdurrahman **h)** Abdul **i)** Abdurrahman **Nationality:** Indonesia **Passport no:** na **National identification no:** na **Address: a)** Desa Gebang, Kecamatan Masaran, Kabupaten Sragen, Jawa Tengah, Indonesia **b)** Desa Taman Fajar, Kecamatan Probolinggo, Kabupaten Lampung Timur, Lampung, Indonesia **Listed on:** 16 May 2005 (amended on 27 May 2022, 17 Apr. 2019) **Other information:** Review pursuant to Security Council resolution 1822 (2008) was concluded on 8 Jun. 2010. Review pursuant to Security Council resolution 2253 (2015) was concluded on 7 June 2018. Review pursuant to Security Council resolution 2368 (2017) was concluded on 15 November 2021. INTERPOL-UN Security Council Special Notice web link: https://www.interpol.int/en/How-we-work/Notices/View-UN-Notices-Individuals click here

QDi.123 Name: 1: YASSIN 2: SYAWAL 3: na 4: na
Title: na **Designation:** na **DOB:** 3 Sep. 1962 **POB:** Makassar, Indonesia **Good quality a.k.a.: a)** Salim Yasin **b)** Yasin Mahmud Mochtar **c)** Abdul Hadi Yasin **d)** Muhamad Mubarok **e)** Muhammad Syawal **f)** Yassin Sywal (formerly listed as) **Low quality a.k.a.: a)** Abu Seta **b)** Mahmud **c)** Abu Muamar **d)** Mubarok **Nationality:** Indonesia **Passport no:** na **National identification no:** na **Address:** na **Listed on:** 9 Sep. 2003 (amended on 12 Dec. 2014, 29 Mar. 2019, 1 May 2019) **Other information:** At large as at Dec. 2003. Review pursuant to Security Council resolution 1822 (2008) was concluded on 25 May 2010. Review pursuant to Security Council resolution 2253 (2015) was concluded on 21 February 2019. Review pursuant to Security Council resolution 2610 (2021) was concluded on 8 November 2022. INTERPOL-UN Security Council Special Notice web link: https://www.interpol.int/en/How-we-work/Notices/View-UN-Notices-Individuals click here

QDi.057 Name: 1: IBRAHIM 2: ALI 3: ABU BAKR 4: TANTOUSH
Name (original script): ابراهيم علي أبو بكر تنتوش
Title: na **Designation:** na **DOB:** 2 Feb. 1966 **POB:** al Aziziyya, Libya **Good quality a.k.a.: a)** Abd al-Muhsin **b)** Ibrahim Ali Muhammad Abu Bakr **c)** Abdul Rahman **d)** Abu Anas **e)** Ibrahim Abubaker Tantouche **f)** Ibrahim Abubaker Tantoush **g)** 'Abd al-Muhsi **h)** 'Abd al-Rahman **i)** Abdel Ilah Sabri (false identity related to fraudulent South African identification number 6910275240086 linked to South African passport number 434021161, both documents have been confiscated) **Low quality a.k.a.:** Al-Libi **Nationality:** Libya **Passport no: a)** Libya number 203037, issued in Tripoli, Libya **b)** (Libyan passport number 347834, issued under name Ibrahim Ali Tantoush, expired on 21 Feb. 2014) **National identification no:** na **Address:** Tripoli, Libya (as at Feb. 2014) **Listed on:** 11 Jan. 2002 (amended on 31 Jul. 2006, 4 Oct. 2006, 16 May 2011, 10 Jul. 2015, 24 Nov. 2020) **Other information:** Associated with Afghan Support Committee (ASC) (QDe.069), Revival of Islamic Heritage Society (RIHS)(QDe.070) and the Libyan Islamic Fighting Group (LIFG) (QDe.011). Photograph and fingerprints available for inclusion in the INTERPOL-UNSC Special Notice. Review pursuant to Security Council resolution 1822 (2008) was concluded on 8 Jun. 2010. Review pursuant to Security Council resolution 2368 (2017) was concluded on 24 November 2020. INTERPOL-UN Security Council Special Notice web link: https://www.interpol.int/en/How-we-work/Notices/View-UN-Notices-Individuals click here

QDi.241 Name: 1: ANGELO 2: RAMIREZ 3: TRINIDAD 4: na
Name (original script):
Title: na **Designation:** na **DOB:** 20 Mar. 1978 **POB:** Gattaran, Cagayan Province, Philippines **Good quality a.k.a.: a)** Calib Trinidad **b)** Kalib Trinidad **Low quality a.k.a.: a)** Abdul Khalil **b)** Abdukahlil **c)** Abu Khalil **d)** Anis **Nationality:** Philippines **Passport no:** na **National identification no:** na **Address:** 3111 Ma. Bautista, Punta, Santa Ana, Manila, Philippines **Listed on:** 4 Jun. 2008 (amended on 13 Dec. 2011, 6 Dec. 2019) **Other information:** Distinguishing marks include scars on both legs. Member of the Rajah Solaiman Movement (QDe.128), and associated with the Abu Sayyaf Group

(QDe.001) and the Jemaah Islamiyah (QDe.092). In detention in the Philippines as of May 2011. Review pursuant to Security Council resolution 1822 (2008) was concluded on 13 May 2010. Review pursuant to Security Council resolution 2368 (2017) was concluded on 4 Dec. 2019. INTERPOL-UN Security Council Special Notice web link: https://www.interpol.int/en/How-we-work/Notices/View-UN-Notices-Individuals click here

QDi.056 Name: 1: MOHAMMED 2: TUFAIL 3: na 4: na
Title: na **Designation:** na **DOB:** 5 May 1930 **POB:** na **Good quality a.k.a.: a)** Tufail, S.M. **b)** Tuffail, Sheik Mohammed **Low quality a.k.a.:** na **Nationality:** Pakistan **Passport no:** na **National identification no:** na **Address:** na **Listed on:** 24 Dec. 2001 (amended on 19 Jan. 2011, 1 May 2019, 2 Feb. 2023) **Other information:** Served as a director of Ummah Tameer e-Nau (UTN) (QDe.068). Reportedly deceased. Review pursuant to Security Council resolution 1822 (2008) was concluded on 1 Jun. 2010. Review pursuant to Security Council resolution 2253 (2015) was concluded on 21 Feb. 2019. INTERPOL-UN Security Council Special Notice web link: https://www.interpol.int/en/How-we-work/Notices/View-UN-Notices-Individuals click here

QDi.290 Name: 1: DOKU 2: KHAMATOVICH 3: UMAROV 4: na
Name (original script): Умаров Доку Хаматович
Title: na **Designation:** na **DOB: a)** 13 Apr. 1964 **b)** 13 Apr. 1965 **c)** 12 May 1964 **d)** 1955 **POB:** Kharsenoy Village, Shatoyskiy (Sovetskiy) District, Chechenskaya Respublika, Russian Federation **Good quality a.k.a.: a)** Lom-ali Butayev (Butaev) born 1955 **b)** Dokka Umarov born 13 Apr. 1964 **c)** Dokka Umarov born 13 Apr. 1965 **Low quality a.k.a.:** na **Nationality: a)** Russian Federation **b)** USSR (until 1991) **Passport no:** Russian Federation number 96 03 464086, issued on 1 Jun. 2003 **National identification no:** na **Address:** na **Listed on:** 10 Mar. 2011 (amended on 2 Jun. 2014, 30 Dec. 2014, 1 May 2019) **Other information:** Physical description: 180 cm tall, dark hair, 7-9 cm. long scar on the face, part of the tongue is missing, has a speech defect. Resides in the Russian Federation as at Nov. 2010. International arrest warrant issued in the year 2000. INTERPOL Special Notice contains biometric information. Reportedly deceased as of April 2014. Review pursuant to Security Council resolution 2253 (2015) was concluded on 21 Feb. 2019. Review pursuant to Security Council resolution 2368 (2017) was concluded on 15 November 2021. INTERPOL-UN Security Council Special Notice web link: https://www.interpol.int/en/How-we-work/Notices/View-UN-Notices-Individuals click here

QDi.031 Name: 1: OMAR 2: MAHMOUD 3: UTHMAN 4: na
Name (original script): عمر محمود عثمان
Title: na **Designation:** na **DOB: a)** 30 Dec. 1960 **b)** 13 Dec. 1960 **POB:** Bethlehem, West Bank, Palestinian Territories **Good quality a.k.a.: a)** Al-Samman Uthman **b)** Umar Uthman **c)** Omar Mohammed Othman **Low quality a.k.a.: a)** Abu Qatada Al-Filistini **b)** Abu Umr Takfiri **c)** Abu Omar Abu Umar **d)** Abu Umar Umar **e)** Abu Ismail **Nationality:** Jordan **Passport no:** na **National identification no:** na **Address:** Jordan (since July 2013) **Listed on:** 17 Oct. 2001 (amended on 14 Mar. 2008, 24 Mar. 2009, 25 Jan. 2010, 22 Jul. 2013, 6 Dec. 2019) **Other information:** Associated with Al-Qaida-related groups in the United Kingdom and other countries. Convicted in absentia in Jordan for involvement in terrorist acts in 1998. Arrested in Feb. 2001 in the United Kingdom, was further detained between Oct. 2002 and Mar. 2005 and between Aug. 2005 and Jun. 2008. In custody since Dec. 2008. Deported to Jordan from the United Kingdom on 7 July 2013 to face terrorism charges. Review pursuant to Security Council resolution 1822 (2008) was concluded on 19 Oct. 2009. Review pursuant to Security Council resolution 2368 (2017) was concluded on 4 Dec. 2019. INTERPOL-UN Security Council Special Notice web link: https://www.interpol.int/en/How-we-work/Notices/View-UN-Notices-Individuals click here

QDi.397 Name: 1: AYRAT 2: NASIMOVICH 3: VAKHITOV 4: na
Name (original script): Айрат Насимович Вахитов
Title: na **Designation:** na **DOB:** 27 Mar. 1977 **POB:** Naberezhnye Chelny, Republic of Tatarstan, Russian Federation **Good quality a.k.a.:** Salman Bulgarskiy (original script: Салман Булгарский)

Low quality a.k.a.: na **Nationality:** Russian Federation **Passport no:** na **National identification no:** na **Address:** na **Listed on:** 3 Aug. 2016 **Other information:** May use a fake passport of a Syrian or Iraqi citizen. Member of the Al-Nusrah Front for the People of the Levant (ANF) (QDe.137), "Bulgar Group", leads a group of 100 fighters. Photo available for inclusion in the INTERPOL-UN Security Council Special Notice. Review pursuant to Security Council resolution 2368 (2017) was concluded on 15 November 2021. INTERPOL-UN Security Council Special Notice web link: https://www.interpol.int/en/How-we-work/Notices/View-UN-Notices-Individuals click here

QDi.037 Name: 1: ABDUL RAHMAN 2: YASIN 3: na 4: na
Name (original script): عبد الرحمن ياسين
Title: na **Designation:** na **DOB:** 10 Apr. 1960 **POB:** Bloomington, Indiana, United States of America **Good quality a.k.a.:** **a)** Taha, Abdul Rahman S. **b)** Taher, Abdul Rahman S. **c)** Yasin, Abdul Rahman Said **d)** Yasin, Aboud **Low quality a.k.a.:** na **Nationality:** United States of America **Passport no:** **a)** 27082171 (United States of America, issued on 21 Jun. 1992 in Amman, Jordan) **b)** Iraq number MO887925 **National identification no:** (SSN 156-92-9858 (United States of America)) **Address:** na **Listed on:** 17 Oct. 2001 (amended on 10 Apr. 2003, 6 Dec. 2019) **Other information:** Abdul Rahman Yasin is in Iraq. Review pursuant to Security Council resolution 1822 (2008) was concluded on 21 Jun. 2010. Review pursuant to Security Council resolution 2368 (2017) was concluded on 4 Dec. 2019. INTERPOL-UN Security Council Special Notice web link: https://www.interpol.int/en/How-we-work/Notices/View-UN-Notices-Individuals click here

QDi.261 Name: 1: ADEM 2: YILMAZ 3: na 4: na
Title: na **Designation:** na **DOB:** 4 Nov. 1978 **POB:** Bayburt, Turkey **Good quality a.k.a.:** na **Low quality a.k.a.:** Talha **Nationality:** Turkey **Passport no:** Turkey number TR-P 614 166 (issued by the Turkish Consulate General in Frankfurt/M. on 22 Mar. 2006, expired on 15 Sep. 2009.) **National identification no:** na **Address:** **a)** (In prison in Germany (since Sep. 2007).) **b)** Südliche Ringstrasse 133, Langen, 63225, Germany (previous address) **Listed on:** 27 Oct. 2008 (amended on 13 Dec. 2011, 6 Dec. 2019) **Other information:** Associated with the Islamic Jihad Union (IJU), also known as the Islamic Jihad Group (QDe.119). Associated with Fritz Martin Gelowicz (QDi.259). In detention in Germany as of Jun. 2010. Review pursuant to Security Council resolution 2368 (2017) was concluded on 4 Dec. 2019. INTERPOL-UN Security Council Special Notice web link: https://www.interpol.int/en/How-we-work/Notices/View-UN-Notices-Individuals click here

QDi.198 Name: 1: HANI 2: AL-SAYYID 3: AL-SEBAI 4: YUSIF
Name (original script): هاني السيد السباعي يوسف
Title: na **Designation:** na **DOB:** **a)** 1 Mar. 1961 **b)** 16 Jun. 1960 **POB:** Qaylubiyah, Egypt **Good quality a.k.a.:** **a)** Hani Yousef Al-Sebai **b)** Hani Youssef **c)** Hany Youseff **d)** Hani Yusef **e)** Hani al-Sayyid Al-Sabai **f)** Hani al-Sayyid El Sebai **g)** Hani al-Sayyid Al Siba'i **h)** Hani al-Sayyid El Sabaay **i)** El-Sababt **j)** Abu Tusnin **k)** Abu Akram **l)** Hani El Sayyed Elsebai Yusef **m)** Abu Karim **n)** Hany Elsayed Youssef **Low quality a.k.a.:** na **Nationality:** Egypt **Passport no:** na **National identification no:** na **Address:** London, United Kingdom of Great Britain and Northern Ireland **Listed on:** 29 Sep. 2005 (amended on 6 Oct. 2005, 18 Aug. 2006, 25 Jan. 2012, 6 Dec. 2019) **Other information:** Father's name is Mohamed Elsayed Elsebai. Review pursuant to Security Council resolution 1822 (2008) was concluded on 29 Jul. 2010. Review pursuant to Security Council resolution 2368 (2017) was concluded on 4 Dec. 2019. INTERPOL-UN Security Council Special Notice web link: https://www.interpol.int/en/How-we-work/Notices/View-UN-Notices-Individuals click here

QDi.139 Name: 1: IMED 2: BEN MEKKI 3: ZARKAOUI 4: na
Name (original script): عماد بن مكي زرقاوي
Title: na **Designation:** na **DOB:** 15 Jan. 1973 **POB:** Tunis, Tunisia **Good quality a.k.a.:** **a)** Dour Nadre born 15 Jan. 1974 in Morocco **b)** Dour Nadre born 15 Jan. 1973 in Morocco **c)** Daour Nadre born 31 Mar. 1975 in Algeria **d)** Imad ben al-Mekki ben al-Akhdar al-Zarkaoui (previously listed as) **Low quality a.k.a.:** **a)** Zarga **b)** Nadra **Nationality:** Tunisia **Passport no:** Tunisia number M174950, issued on 27 Apr. 1999 (expired on 26 Apr. 2004) **National identification no:** na **Address:** 41-45

Rue Estienne d' Orves, Pré Saint Gervais, France **Listed on:** 12 Nov. 2003 (amended on 20 Dec. 2005, 31 Jul. 2006, 10 Aug. 2009, 16 May 2011, 6 Dec. 2019, 10 Sep. 2020) **Other information:** Mother' s name is Zina al-Zarkaoui. Sentenced to seven years and one month of imprisonment by the Court of Appeals of Milan in Italy. Released on 31 Mar. 2014 on early release. Review pursuant to Security Council resolution 1822 (2008) was concluded on 6 May 2010. Review pursuant to Security Council resolution 2368 (2017) was concluded on 4 Dec. 2019. INTERPOL-UN Security Council Special Notice web link: https://www.interpol.int/en/How-we-work/Notices/View-UN-Notices-Individuals click here

QDi.428 Name: 1: JAMAL 2: HUSSEIN 3: HASSAN 4: ZEINIYE
Name (original script): جمال حسين حسن زينيه
Title: na **Designation:** na **DOB: a)** 17 Aug. 1972 **b)** 1 Jan. 1972 **POB: a)** Benghazi, Libya **b)** Al Tall, Syrian Arab Republic **c)** Tell Mnin, Syrian Arab Republic **Good quality a.k.a.:** Jamal Husayn Zayniyah **Low quality a.k.a.: a)** Abu Malek El Talleh **b)** Abu Hussein **c)** abu-Malik al-Ansari **d)** Abu-Malik al-Shami **e)** Abu-Malik al-Talli **Nationality:** Syrian Arab Republic **Passport no:** Syrian Arab Republic number 3987189 **National identification no: a)** 13080011550 **b)** Syrian Arab Republic Identification Card No. 5877002, issued on 25 May 2011 **Address: a)** Syrian Arab Republic **b)** Arsal, Bekaa, Lebanon **Listed on:** 8 Oct. 2020 **Other information:** Leader of AL-NUSRAH FRONT FOR THE PEOPLE OF THE LEVANT (QDe.137) in West Kalamoun, Syrian Arab Republic. Mother' s name is Amina Tohmeh. INTERPOL-UN Security Council Special Notice web link: https://www.interpol.int/en/How-we-work/Notices/View-UN-Notices-Individuals click here

QDi.001 Name: 1: MOHAMMED 2: SALAHALDIN 3: ABD EL HALIM 4: ZIDANE
Name (original script): محمد صلاح الدين عبدالحليم زيدان
Title: na **Designation:** na **DOB: a)** 11 Apr. 1963 **b)** 11 Apr. 1960 **POB:** Monufia Governate, Egypt **Good quality a.k.a.: a)** Sayf-Al Adl (DOB: 11 Apr. 1963. POB: Monufia Governorate, Egypt. Nationality: Egypt. In Arabic: سيف العدل) **b)** Muhamad Ibrahim Makkawi (DOB: a) 11 Apr. 1960 b) 11 Apr. 1963. POB: Egypt. Nationality: Egypt) **Low quality a.k.a.: a)** Ibrahim al-Madani **b)** Saif Al-'Adil **c)** Seif al Adel **Nationality:** Egypt **Passport no:** na **National identification no:** na **Address:** na **Listed on:** 25 Jan. 2001 (amended on 16 Dec. 2010, 24 Jul. 2013, 15 Feb. 2017, 29 Mar. 2019, 1 May 2019) **Other information:** Responsible for Usama bin Laden' s (deceased) security. Hair: Dark. Eyes: Dark. Photo available for inclusion in the INTERPOL-UN Security Council Special Notice . Review pursuant to Security Council resolution 1822 (2008) was concluded on 15 Jun. 2010. Review pursuant to Security Council resolution 2253 (2015) was concluded on 21 February 2019. Review pursuant to Security Council resolution 2610 (2021) was concluded on 8 November 2022. INTERPOL-UN Security Council Special Notice web link: https://www.interpol.int/en/How-we-work/Notices/View-UN-Notices-Individuals click here

QDi.223 Name: 1: MERAI 2: ABDEFATTAH 3: KHALIL 4: ZOGHBI
Name (original script): مرعي عبدفتاح خليل زغبي
Title: na **Designation:** na **DOB: a)** 4 Apr. 1969 **b)** 4 Apr. 1960 **c)** 4 Jun. 1960 **POB:** Bengasi, Libya **Good quality a.k.a.: a)** Mohamed Lebachir born 14 Jan. 1968 in Morocco **b)** Meri Albdelfattah Zgbye born 4 Jun. 1960 in Bendasi, Libya **c)** Zoghbai Merai Abdul Fattah **d)** Lazrag Faraj born 13 Nov. 1960 in Libya **e)** Larzg Ben Ila born 11 Aug. 1960 in Libya **f)** Muhammed El Besir **g)** Merai Zoghbai (previously listed as, in Arabic: مرعي زغبي) **Low quality a.k.a.: a)** F' raji di Singapore **b)** F' raji il Libico **c)** Farag **d)** Fredj born 13 Nov. 1960 in Libya **Nationality:** Libya **Passport no:** na **National identification no:** na **Address:** na **Listed on:** 2 Aug. 2006 (amended on 3 Jun. 2009, 1 Sep. 2009, 13 Dec. 2011, 21 Mar. 2017, 24 Nov. 2020) **Other information:** Considered a fugitive from justice by the Italian authorities and sentenced in absentia to 6 years imprisonment on 20 Nov. 2008. Member of Libyan Islamic Fighting Group (QDe.011). Son of Wanisa Abdessalam. Review pursuant to Security Council resolution 1822 (2008) was concluded on 20 Jul. 2009. Review pursuant to Security Council resolution 2368 (2017) was concluded on 24 November 2020. Review pursuant to Security Council resolution 2610 (2021) was concluded on 8 November 2022. INTERPOL-UN

Security Council Special Notice web link: https://www.interpol.int/en/How-we-work/Notices/View-UN-Notices-Individuals click here

QDi.426 Name: 1: Amir 2: Muhammad Sa’id 3: Abdal-Rahman 4: al-Salbi
Name (original script): أمير محمد سعيد عبد الرحمن السلبي
Title: na **Designation:** na **DOB:** **a)** 5 Oct. 1976 **b)** 1 Oct. 1976 **c)** 6 Jan. 1976 **POB:** **a)** Tall’Afar, Iraq **b)** Mosul, Iraq **Good quality a.k.a.:** **a)** Abu Ibrahim al-Hashimi al-Qurashi **b)** Hajji Abdallah **c)** Abu ‘Umar al-Turkmani **d)** Abdullah Qardash **e)** Abu ‘Abdullah Qardash **f)** al-Hajj Abdullah Qardash **g)** Hajji Abdullah Al-Afari **h)** `Abdul Amir Muhammad Sa'id Salbi **i)** Muhammad Sa'id `Abd-al-Rahman al-Mawla **j)** Amir Muhammad Sa’id ‘Abd-al-Rahman Muhammad al-Mula **k)** Amir Muhammad Sa’id Abdal-Rahman al-Mawla (previously listed as) **Low quality a.k.a.:** **a)** Al-Ustadh **b)** Ustadh Ahmad **Nationality:** Iraq **Passport no:** na **National identification no:** na **Address:** **a)** House 110, Street 704, District 704, Tall ‘Afar, Iraq (previous address) **b)** near Shahid Mazen Mosque and al-Khansa Hospital, Mosul, Iraq (previous address) **c)** Idlib, Syrian Arab Republic **Listed on:** 21 May 2020 (amended on 27 May 2022) **Other information:** Leader of Islamic State in Iraq and the Levant, listed as Al-Qaida in Iraq (QDe.115). Mother’s name: Samira Shareef (سميرة شريف) or Sahra Sharif Abd al-Qader (سهرة شريف عبد القادر). Height 170 cm, right leg amputated. Photo available for inclusion in the INTERPOL-UN Security Council Special Notice. Arrest warrant issued by Iraq 2018. Reportedly deceased as of 3 February 2022. INTERPOL-UN Security Council Special Notice web link: https://www.interpol.int/en/How-we-work/Notices/View-UN-Notices-Individuals click here

QDi.289 Name: 1: SAID JAN 2: ‘ABD AL-SALAM 3: na 4: na
Name (original script): سعيد جان عبد السلام
Title: na **Designation:** na **DOB:** **a)** 5 Feb. 1981 **b)** 1 Jan. 1972 **POB:** na **Good quality a.k.a.:** **a)** Sa'id Jan 'Abd-al-Salam **b)** Dilawar Khan Zain Khan born 1 Jan. 1972 **Low quality a.k.a.:** **a)** Qazi 'Abdallah **b)** Qazi Abdullah **c)** Ibrahim Walid **d)** Qasi Sa'id Jan **e)** Said Jhan **f)** Farhan Khan **g)** Aziz Cairo **h)** Nangiali **Nationality:** Afghanistan **Passport no:** **a)** Afghan number OR801168, issued on 28 Feb. 2006 (expires 27 Feb. 2011, under name Said Jan 'Abd al-Salam) **b)** Pakistan number 4117921, issued on 9 Sep. 2008 (expires 9 Sep. 2013, issued under name Dilawar Khan Zain Khan) **National identification no:** Kuwait Civil Identification number 281020505755 (issued under name Said Jan 'Abd al-Salam) **Address:** na **Listed on:** 9 Feb. 2011 (amended on 1 May 2019) **Other information:** In approximately 2005, ran a "basic training" camp for Al-Qaida (QDe.004) in Pakistan. Review pursuant to Security Council resolution 2253 (2015) was concluded on 21 Feb. 2019. INTERPOL-UN Security Council Special Notice web link: https://www.interpol.int/en/How-we-work/Notices/View-UN-Notices-Individuals click here

B. Entities and other groups

QDe.144 Name: ABDALLAH AZZAM BRIGADES (AAB)
A.k.a.: **a)** Abdullah Azzam Brigades **b)** Ziyad al-Jarrah Battalions of the Abdallah Azzam Brigades **c)** Yusuf al-'Uyayri Battalions of the Abdallah Azzam Brigades **F.k.a.:** na **Address:** (Operates in Lebanon, Syria and the Arabian Peninsula) **Listed on:** 23 Sep. 2014 (amended on 6 Dec. 2019) **Other information:** An armed group that has carried out joint attacks with Al-Nusrah Front for the People of the Levant (QDe.137). Review pursuant to Security Council resolution 2368 (2017) was concluded on 4 Dec. 2019. INTERPOL-UN Security Council Special Notice web link: https://www.interpol.int/en/How-we-work/Notices/View-UN-Notices-Entities click here

QDe.001 Name: ABU SAYYAF GROUP
A.k.a.: Al Harakat Al Islamiyya **F.k.a.:** na **Address:** Philippines **Listed on:** 6 Oct. 2001 (amended on 13 Dec. 2011, 24 Nov. 2020) **Other information:** Associated with Jemaah Islamiyah (JI) (QDe.092). Current leader is Radulan Sahiron (QDi.208). Review pursuant to Security Council resolution 1822 (2008) was concluded on 21 Jun. 2010. Review pursuant to Security Council resolution 2368 (2017)

was concluded on 24 November 2020. INTERPOL-UN Security Council Special Notice web link: https://www.interpol.int/en/How-we-work/Notices/View-UN-Notices-Entities click here

QDe.069 Name: AFGHAN SUPPORT COMMITTEE (ASC)
A.k.a.: a) Lajnat ul Masa Eidatul Afghania **b)** Jamiat Ayat-ur-Rhas al Islamiac **c)** Jamiat Ihya ul Turath al Islamia **d)** Ahya ul Turas **F.k.a.:** na **Address: a)** Headquarters – G.T. Road (probably Grand Trunk Road), near Pushtoon Garhi Pabbi, Peshawar, Pakistan **b)** Cheprahar Hadda, Mia Omar Sabaqah School, Jalabad, Afghanistan **Listed on:** 11 Jan. 2002 (amended on 13 Dec. 2011) **Other information:** Associated with the Revival of Islamic Heritage Society (QDe.070). Abu Bakr al-Jaziri (QDi.058) served as finance chief of ASC. Review pursuant to Security Council resolution 1822 (2008) was concluded on 8 Jun. 2010. Review pursuant to Security Council resolution 2368 (2017) was concluded on 15 November 2021. INTERPOL-UN Security Council Special Notice web link: https://www.interpol.int/en/How-we-work/Notices/View-UN-Notices-Entities click here

QDe.107 Name: AL FURQAN
A.k.a.: a) Dzemilijati Furkan **b)** Dzem'ijjetul Furqan **c)** Association for Citizens Rights and Resistance to Lies **d)** Dzemijetul Furkan **e)** Association of Citizens for the Support of Truth and Supression of Lies **f)** Sirat **g)** Association for Education, Culture and Building Society-Sirat **h)** Association for Education, Cultural, and to Create Society -Sirat **i)** Istikamet **j)** In Siratel **k)** Citizens' Association for Support and Prevention of lies – Furqan **F.k.a.:** na **Address: a)** 30a Put Mladih Muslimana (ex Pavla Lukaca Street), 71 000 Sarajevo, Bosnia and Herzegovina **b)** 72 ul. Strossmajerova, Zenica, Bosnia and Herzegovina **c)** 42 Muhameda Hadzijahica, Sarajevo, Bosnia and Herzegovina **d)** 70 and 53 Strosmajerova Street, Zenica, Bosnia and Herzegovina **e)** Zlatnih Ljiljana Street, Zavidovici, Bosnia and Herzegovina **Listed on:** 11 May 2004 (amended on 26 Nov. 2004, 24 Mar. 2009, 1 May 2019) **Other information:** Registered in Bosnia and Herzegovina as a citizens' association under the name of "Citizens' Association for Support and Prevention of lies – Furqan" on 26 Sep. 1997. Al Furqan ceased its work by decision of the Ministry of Justice of the Bosnia and Herzegovina Federation (decision number 03-054-286/97 dated 8 Nov. 2002). Al Furqan was no longer in existence as at Dec. 2008. Review pursuant to Security Council resolution 1822 (2008) was concluded on 15 Jun. 2010. Review pursuant to Security Council resolution 2253 (2015) was concluded on 21 Feb. 2019. Review pursuant to Security Council resolution 2368 (2017) was concluded on 15 November 2021. INTERPOL-UN Security Council Special Notice web link: https://www.interpol.int/en/How-we-work/Notices/View-UN-Notices-Entities click here

QDe.139 Name: AL MOUAKAOUNE BIDDAM
Name (original script): الموقعون بالدم
A.k.a.: a) Les Signataires par le Sang **b)** Ceux Qui Signent avec le Sang **c)** Those Who Sign in Blood **F.k.a.:** na **Address:** Mali **Listed on:** 2 Jun. 2014 (amended on 6 Dec. 2019) **Other information:** Associated with the Organization of Al-Qaida in the Islamic Maghreb (QDe.014) and led by Mokhtar Belmokhtar (QDi.136). Active in the Sahel/Sahara region. Review pursuant to Security Council resolution 2368 (2017) was concluded on 4 Dec. 2019 INTERPOL-UN Security Council Special Notice web link: https://www.interpol.int/en/How-we-work/Notices/View-UN-Notices-Entities click here

QDe.140 Name: AL MOULATHAMOUN
Name (original script): الملثمون
A.k.a.: a) Les Enturbannés **b)** The Veiled **F.k.a.:** na **Address: a)** Mali **b)** Niger **c)** Algeria **Listed on:** 2 Jun. 2014 (amended on 6 Dec. 2019) **Other information:** Founded in 2012 as a splinter group of the Organization of Al-Qaida in the Islamic Maghreb (QDe.014). On 20 Aug. 2013, Al Moulathamoun merged with the Mouvement pour l' Unification et le Jihad en Afrique de l' Ouest (MUJAO) (QDe.134) and established Al Mourabitoun (QDe.141). Associated with the Organization of Al-Qaida in the Islamic Maghreb (QDe.014) and led by Mokhtar Belmokhtar (QDi.136). Active in the Sahel/Sahara region. Review pursuant to Security Council resolution 2368 (2017) was concluded on

4 Dec. 2019 INTERPOL-UN Security Council Special Notice web link: https://www.interpol.int/en/How-we-work/Notices/View-UN-Notices-Entities click here

QDe.141 Name: AL MOURABITOUN
Name (original script): المرابطون
A.k.a.: a) Les Sentinelles **b)** The Sentinels **F.k.a.:** na **Address:** Mali **Listed on:** 2 Jun. 2014 (amended on 6 Dec. 2019) **Other information:** Founded on 20 Aug. 2013 as result of a merger between Al Moulathamoun (QDe.140) and the Mouvement pour l' Unification et le Jihad en Afrique de l' Ouest (MUJAO) (QDe.134). Associated with the Organization of Al-Qaida in the Islamic Maghreb (QDe.014) and led by Mokhtar Belmokhtar (QDi.136). Active in the Sahel/Sahara region. Review pursuant to Security Council resolution 2368 (2017) was concluded on 4 Dec. 2019 INTERPOL-UN Security Council Special Notice web link: https://www.interpol.int/en/How-we-work/Notices/View-UN-Notices-Entities click here

QDe.005 Name: AL RASHID TRUST
A.k.a.: a) Al-Rasheed Trust **b)** Al Rasheed Trust **c)** Al-Rashid Trust **d)** Aid Organization of the Ulema, Pakistan **e)** Al Amin Welfare Trust **f)** Al Amin Trust **g)** Al Ameen Trust **h)** Al-Ameen Trust **i)** Al Madina Trust **j)** Al-Madina Trust **F.k.a.:** na **Address: a)** Kitas Ghar, Nazimabad 4, Dahgel-Iftah, Karachi, Pakistan **b)** Jamia Maajid, Sulalman Park, Melgium Pura, Lahore, Pakistan **c)** (Office Dha' rbi-M' unin, Opposite Khyber Bank, Abbottabad Road, Mansehra, Pakistan) **d)** (Office Dha' rbi-M' unin ZR Brothers, Katcherry Road, Chowk Yadgaar, Peshawar, Pakistan) **e)** (Office Dha' rbi-M' unin, Rm No. 3, Moti Plaza, Near Liaquat Bagh, Muree Road, Rawalpindi, Pakistan) **f)** (Office Dha' rbi-M' unin, Top Floor, Dr. Dawa Khan Dental Clinic Surgeon, Main Baxae, Mingora, Swat, Pakistan) **g)** (Kitab Ghar, Darul Ifta Wal Irshad, Nazimabad No. 4, Karachi, Pakistan, Phone 6683301; Phone 0300-8209199; Fax 6623814) **h)** (302b-40, Good Earth Court, Opposite Pia Planitarium, Block 13a, Gulshan -l Igbal, Karachi, Pakistan; Phone 4979263) **i)** (617 Clifton Center, Block 5, 6th Floor, Clifton, Karachi, Pakistan; Phone 587-2545) **j)** (605 Landmark Plaza, 11 Chundrigar Road, Opposite Jang Building, Karachi, Pakistan; Phone 2623818-19) **k)** (Jamia Masjid, Sulaiman Park, Begum Pura, Lahore, Pakistan; Phone 042-6812081) **Listed on:** 6 Oct. 2001 (amended on 21 Oct. 2008, 10 Dec. 2008, 13 Dec. 2011, 6 Dec. 2019) **Other information:** Headquarters are in Pakistan. Operations in Afghanistan: Herat Jalalabad, Kabul, Kandahar, Mazar Sherif. Also operations in Kosovo, Chechnya. Involved in the financing of Al-Qaida and the Taliban. Until 21 Oct. 2008, this entity appeared also as "Aid Organization of the Ulema, Pakistan" (QDe.073), listed on 24 Apr. 2002 and amended on 25 Jul. 2006. The two entries Al Rashid Trust (QDe.005) and Aid Organization of the Ulema, Pakistan (QDe.073) were consolidated into this entity on 21 Oct. 2008. Founded by Mufti Rashid Ahmad Ledahyanoy (deceased). Associated with Jaish-i-Mohammed (QDe.019). Banned in Pakistan since Oct. 2001. Despite the closure of its offices in Pakistan in February 2007 it has continued its activities. Review pursuant to Security Council resolution 1822 (2008) was concluded on 6 May 2010. Review pursuant to Security Council resolution 2368 (2017) was concluded on 4 Dec. 2019. INTERPOL-UN Security Council Special Notice web link: https://www.interpol.int/en/How-we-work/Notices/View-UN-Notices-Entities click here

QDe.121 Name: AL-AKHTAR TRUST INTERNATIONAL
A.k.a.: a) Al Akhtar Trust **b)** Al-Akhtar Medical Centre **c)** Akhtarabad Medical Camp **d)** Pakistan Relief Foundation **e)** Pakistani Relief Foundation **f)** Azmat-e-Pakistan Trust **g)** Azmat Pakistan Trust **F.k.a.:** na **Address: a)** ST-1/A, Gulsahn-e-Iqbal, Block 2, Karachi, 25300, Pakistan **b)** Gulistan-e-Jauhar, Block 12, Karachi, Pakistan **Listed on:** 17 Aug. 2005 (amended on 10 Dec. 2008, 13 Dec. 2011, 1 May 2019) **Other information:** Regional offices in Pakistan: Bahawalpur, Bawalnagar, Gilgit, Islamabad, Mirpur Khas, Tando-Jan-Muhammad. Akhtarabad Medical Camp is in Spin Boldak, Afghanistan. Registered by members of Jaish-i-Mohammed (QDe.019). Associated with Harakat ul-Mujahidin/ HUM (QDe.008), Lashkar I Jhanghvi (LJ) (QDe.096) and Lashkar-e-Tayyiba (QDe.118). Banned in Pakistan. Review pursuant to Security Council resolution 1822 (2008) was concluded on 14 Sep. 2009. Review pursuant to Security Council resolution 2253 (2015) was concluded on 21 Feb. 2019. Review pursuant to Security Council resolution 2610 (2021) was concluded on 8 November

2022. INTERPOL-UN Security Council Special Notice web link: https://www.interpol.int/en/How-we-work/Notices/View-UN-Notices-Entities click here

QDe.109 Name: AL-HARAMAIN & AL MASJED AL-AQSA CHARITY FOUNDATION
A.k.a.: a) Al Haramain Al Masjed Al Aqsa **b)** Al Haramayn Al Masjid Al Aqsa **c)** Al-Haramayn and Al Masjid Al Aqsa Charitable Foundation **d)** Al Harammein Al Masjed Al-Aqsa Charity Foundation **F.k.a.:** na **Address: a)** Branch Address: 2A Hasiba Brankovica, Sarajevo, Bosnia and Herzegovina **b)** 14 Bihacka Street, Sarajevo, Bosnia and Herzegovina **c)** 64 Potur mahala Street, Travnik, Bosnia and Herzegovina **d)** Zenica, Bosnia and Herzegovina **Listed on:** 28 Jun. 2004 (amended on 26 Nov. 2004, 16 Sep. 2008, 24 Mar. 2009, 24 Nov. 2020, 2 Feb. 2023) **Other information:** Reportedly defunct. Used to be officially registered in Bosnia and Herzegovina under registry number 24. Al-Haramain & Al Masjed Al-Aqsa Charity Foundation ceased its work by decision of the Ministry of Justice of the Bosnia and Herzegovina Federation (decision on cessation of operation number 03-05-2-203/04). It was no longer in existence as of Dec. 2008. Its premises and humanitarian activities were transferred under Government supervision to a new entity called Sretna Buducnost. Review pursuant to Security Council resolution 1822 (2008) was concluded on 22 Jun. 2010. Review pursuant to Security Council resolution 2368 (2017) was concluded on 24 November 2020. INTERPOL-UN Security Council Special Notice web link: https://www.interpol.int/en/How-we-work/Notices/View-UN-Notices-Entities click here

QDe.104 Name: AL-HARAMAIN FOUNDATION (PAKISTAN)
A.k.a.: na **F.k.a.:** na **Address:** House #279, Nazimuddin Road, F-10/1, Islamabad, Pakistan (at time of listing) **Listed on:** 26 Jan. 2004 (amended on 21 Mar. 2012, 24 Nov. 2020) **Other information:** Review pursuant to Security Council resolution 1822 (2008) was concluded on 19 Oct. 2009. Review pursuant to Security Council resolution 2368 (2017) was concluded on 24 November 2020. INTERPOL-UN Security Council Special Notice web link: https://www.interpol.int/en/How-we-work/Notices/View-UN-Notices-Entities click here

QDe.116 Name: AL-HARAMAIN FOUNDATION (UNION OF THE COMOROS)
A.k.a.: na **F.k.a.:** na **Address:** B/P: 1652 Moroni, Union of the Comoros (at time of listing) **Listed on:** 28 Sep. 2004 (amended on 21 Mar. 2012, 24 Nov. 2020) **Other information:** Review pursuant to Security Council resolution 1822 (2008) was concluded on 22 Jun. 2010. Review pursuant to Security Council resolution 2368 (2017) was concluded on 24 November 2020. Review pursuant to Security Council resolution 2610 (2021) was concluded on 8 November 2022. INTERPOL-UN Security Council Special Notice web link: https://www.interpol.int/en/How-we-work/Notices/View-UN-Notices-Entities click here

QDe.110 Name: AL-HARAMAIN: AFGHANISTAN BRANCH
A.k.a.: na **F.k.a.:** na **Address:** Afghanistan (at time of listing) **Listed on:** 6 Jul. 2004 (amended on 21 Mar. 2012, 24 Nov. 2020) **Other information:** Review pursuant to Security Council resolution 1822 (2008) was concluded on 22 Jun. 2010. Review pursuant to Security Council resolution 2368 (2017) was concluded on 24 November 2020. INTERPOL-UN Security Council Special Notice web link: https://www.interpol.int/en/How-we-work/Notices/View-UN-Notices-Entities click here

QDe.111 Name: AL-HARAMAIN: ALBANIA BRANCH
A.k.a.: na **F.k.a.:** na **Address:** Irfan Tomini Street, #58, Tirana, Albania (at time of listing) **Listed on:** 6 Jul. 2004 (amended on 21 Mar. 2012, 24 Nov. 2020) **Other information:** Review pursuant to Security Council resolution 1822 (2008) was concluded on 22 Jun. 2010. Review pursuant to Security Council resolution 2368 (2017) was concluded on 24 November 2020. INTERPOL-UN Security Council Special Notice web link: https://www.interpol.int/en/How-we-work/Notices/View-UN-Notices-Entities click here

QDe.112 Name: AL-HARAMAIN: BANGLADESH BRANCH
A.k.a.: na **F.k.a.:** na **Address:** House 1, Road 1, S-6, Uttara, Dhaka, Bangladesh (at time of listing)

Listed on: 6 Jul. 2004 (amended on 21 Mar. 2012, 24 Nov. 2020) **Other information:** Review pursuant to Security Council resolution 1822 (2008) was concluded on 22 Jun. 2010. Review pursuant to Security Council resolution 2368 (2017) was concluded on 24 November 2020. INTERPOL-UN Security Council Special Notice web link: https://www.interpol.int/en/How-we-work/Notices/View-UN-Notices-Entities click here

QDe.113 Name: AL-HARAMAIN: ETHIOPIA BRANCH
A.k.a.: na **F.k.a.:** na **Address:** Woreda District 24 Kebele Section 13, Addis Ababa, Ethiopia (at time of listing) **Listed on:** 6 Jul. 2004 (amended on 21 Mar. 2012, 24 Nov. 2020) **Other information:** Review pursuant to Security Council resolution 1822 (2008) was concluded on 22 Jun. 2010. Review pursuant to Security Council resolution 2368 (2017) was concluded on 24 November 2020. INTERPOL-UN Security Council Special Notice web link: https://www.interpol.int/en/How-we-work/Notices/View-UN-Notices-Entities click here

QDe.114 Name: AL-HARAMAIN: THE NETHERLANDS BRANCH
A.k.a.: Stichting Al Haramain Humanitarian Aid **F.k.a.:** na **Address:** Jan Hanzenstraat 114, 1053SV, Amsterdam, The Netherlands (at time of listing) **Listed on:** 6 Jul. 2004 (amended on 13 Apr. 2012, 15 Jun. 2015, 24 Nov. 2020, 2 Feb. 2023) **Other information:** Reportedly defunct.Review pursuant to Security Council resolution 1822 (2008) was concluded on 28 Jun. 2010. Review pursuant to Security Council resolution 2368 (2017) was concluded on 24 November 2020. INTERPOL-UN Security Council Special Notice web link: https://www.interpol.int/en/How-we-work/Notices/View-UN-Notices-Entities click here

QDe.105 Name: AL-HARAMAYN FOUNDATION (KENYA)
A.k.a.: na **F.k.a.:** na **Address: a)** Nairobi, Kenya (at time of listing) **b)** Garissa, Kenya (at time of listing) **c)** Dadaab, Kenya (at time of listing) **Listed on:** 26 Jan. 2004 (amended on 21 Mar. 2012, 1 May 2019) **Other information:** Review pursuant to Security Council resolution 1822 (2008) was concluded on 22 Jun. 2010. Review pursuant to Security Council resolution 2253 (2015) was concluded on 21 Feb. 2019. Review pursuant to Security Council resolution 2610 (2021) was concluded on 8 November 2022. INTERPOL-UN Security Council Special Notice web link: https://www.interpol.int/en/How-we-work/Notices/View-UN-Notices-Entities click here

QDe.106 Name: AL-HARAMAYN FOUNDATION (TANZANIA)
A.k.a.: na **F.k.a.:** na **Address: a)** P.O. Box 3616, Dar es Salaam, Tanzania (at time of listing) **b)** Tanga, Tanzania (at time of listing) **c)** Singida, Tanzania (at time of listing) **Listed on:** 26 Jan. 2004 (amended on 21 Mar. 2012, 24 Nov. 2020) **Other information:** Review pursuant to Security Council resolution 1822 (2008) was concluded on 22 Jun. 2010. Review pursuant to Security Council resolution 2368 (2017) was concluded on 24 November 2020. INTERPOL-UN Security Council Special Notice web link: https://www.interpol.int/en/How-we-work/Notices/View-UN-Notices-Entities click here

QDe.002 Name: AL-ITIHAAD AL-ISLAMIYA / AIAI
Name (original script): الاتحاد الاسلامي
A.k.a.: na **F.k.a.:** na **Address:** na **Listed on:** 6 Oct. 2001 (amended on 21 Dec. 2007, 13 Dec. 2011, 18 Mar. 2013, 9 Aug. 2019, 24 Nov. 2020) **Other information:** Reported to have operated in Somalia and Ethiopia and to have merged with Harakat Al-Shabaab Al-Mujaahidiin (Al-Shabaab), which was accepted as an affiliate of Al-Qaida (QDe.004) by Aiman Muhammed Rabi al-Zawahiri (QDi.006) in Feb. 2012, and is also subject to the sanctions measures set out in Security Council resolution 1844 (2008) concerning Somalia and Eritrea (see https://www.un.org/sc/suborg/en/sanctions/751). Leadership included Hassan Dahir Aweys (QDi.042). AIAI has received funds through the Al-Haramain Islamic Foundation (Somalia) (QDe.072). Review pursuant to Security Council resolution 1822 (2008) was concluded on 21 Jun. 2010. Review pursuant to Security Council resolution 2368 (2017) was concluded on 24 November

2020. INTERPOL-UN Security Council Special Notice web link: https://www.interpol.int/en/How-we-work/Notices/View-UN-Notices-Entities click here

QDe.157 Name: AL-KAWTHAR MONEY EXCHANGE
Name (original script): شركة الكوثر للتوسط ببيع وشراء العملات الأجنبية
A.k.a.: a) Al Kawthar Co. **b)** Al Kawthar Company **c)** Al-Kawthar Hawala **F.k.a.:** na **Address:** Al-Qaim, Al Anbar Province, Iraq **Listed on:** 6 Mar. 2018 **Other information:** Money exchange business and owned by Umar Mahmud Irhayyim al-Kubaysi (QDi.412) as of mid-2016. Facilitated financial transactions on behalf of companies associated with Islamic State in Iraq and the Levant (ISIL), listed as Al-Qaida in Iraq (QDe.115). Established in 2000 under License number 202, issued on 17 May 2000, and since withdrawn. INTERPOL-UN Security Council Special Notice web link: https://www.interpol.int/en/How-we-work/Notices/View-UN-Notices-Entities click here

QDe.137 Name: AL-NUSRAH FRONT FOR THE PEOPLE OF THE LEVANT
Name (original script): جبهة النصرة لأهل الشام
A.k.a.: a) Hay’ at Tahrir al-Sham (HTS) (هيئة تحرير الشام (original script); Hay’ at Tahrir al-Sham; Hay’ et Tahrir al-Sham; Hayat Tahrir al-Sham; Assembly for the Liberation of Syria; Assembly for the Liberation of the Levant; Liberation of al-Sham Commission; Liberation of the Levant Organisation Tahrir al-Sham; Tahrir al-Sham Hay’ at) **b)** جبهة النصرة (the Victory Front; Jabhat al-Nusrah; Jabhet al-Nusra; Al-Nusrah Front; Al-Nusra Front) **c)** جبهة فتح الشام (Jabhat Fath al Sham; Jabhat Fath al-Sham; Jabhat Fatah al-Sham; Jabhat Fateh Al-Sham; Fatah al-Sham Front; Fateh al-Sham Front) **d)** Conquest of the Levant Front **e)** The Front for the Liberation of al Sham **f)** Front for the Conquest of Syria/the Levant **g)** Front for the Liberation of the Levant **h)** Front for the Conquest of Syria **i)** شبكة أنصار المجاهدين (Ansar al-Mujahideen Network - sub-unit name) **j)** مجاهدو الشام في ساحات الجهاد (Levantine Mujahideen on the Battlefields of Jihad - sub-unit name) **F.k.a.:** na **Address: a)** Syrian Arab Republic (Operates in) **b)** Iraq (Support network) **Listed on:** 14 May 2014 (amended on 7 Jun. 2017, 5 Jun. 2018) **Other information:** Associated with Al-Qaida (QDe.004). Brought Syrian and foreign Al-Qaida in Iraq (QDe.115) and Asbat al-Ansar (QDe.007) fighters, along with other foreign Al-Qaida operatives, to join local elements in Syrian Arab Republic to carry out terrorist and guerrilla operations there. Previously associated with the Islamic State in Iraq and the Levant (ISIL), listed as Al-Qaida in Iraq (QDe.115), and its leader Ibrahim Awwad Ibrahim Ali al-Badri al-Samarrai (QDi.299) but separated from that group in 2013. In Jul. 2016, Abu Mohammed Al-Jawlani (QDi.317), the leader of Al-Nusrah Front for the People of the Levant, announced the group had changed its name to Jabhat Fath al-Sham and was no longer affiliated with any external entity. Despite the announcement and attempts to distinguish itself from Al-Nusrah Front for the People of the Levant, the group remains aligned with Al-Qaida and continues to carry out terrorist operations under this new name. In January 2017, Al-Nusrah Front created Hay’ at Tahrir al-Sham (HTS) as a vehicle to advance its position in the Syrian insurgency and further its own goals as Al-Qaida’ s affiliate in Syria. Previously listed between 30 May 2013 and 13 May 2014 as an aka of Al-Qaida in Iraq (QDe.115). Review pursuant to Security Council resolution 2368 (2017) was concluded on 15 November 2021. INTERPOL-UN Security Council Special Notice web link: https://www.interpol.int/en/How-we-work/Notices/View-UN-Notices-Entities click here

QDe.004 Name: AL-QAIDA
Name (original script): القاعدة
A.k.a.: a) "The Base" **b)** Al Qaeda **c)** Islamic Salvation Foundation **d)** The Group for the Preservation of the Holy Sites **e)** The Islamic Army for the Liberation of Holy Places **f)** The World Islamic Front for Jihad Against Jews and Crusaders **g)** Usama Bin Laden Network **h)** Usama Bin Laden Organization **i)** Al Qa'ida **j)** Al Qa’ ida/Islamic Army **F.k.a.:** na **Address:** na **Listed on:** 6 Oct. 2001 (amended on 5 Mar. 2009, 21 Mar. 2012, 24 Nov. 2020) **Other information:** Review pursuant to Security Council resolution 1822 (2008) was concluded on 21 Jun. 2010. Review pursuant to Security Council resolution 2368 (2017) was concluded on 24 November 2020. INTERPOL-UN Security Council Special Notice web link: https://www.interpol.int/en/How-we-work/Notices/View-UN-Notices-Entities click here

QDe.115 Name: AL-QAIDA IN IRAQ
Name (original script): القاعده في العراق
A.k.a.: a) AQI **b)** al-Tawhid **c)** the Monotheism and Jihad Group **d)** Qaida of the Jihad in the Land of the Two Rivers **e)** Al-Qaida of Jihad in the Land of the Two Rivers **f)** The Organization of Jihad's Base in the Country of the Two Rivers **g)** The Organization Base of Jihad/Country of the Two Rivers **h)** The Organization Base of Jihad/Mesopotamia **i)** Tanzim Qa'idat Al-Jihad fi Bilad al-Rafidayn **j)** Tanzeem Qa'idat al Jihad/Bilad al Raafidaini **k)** Jama'at Al-Tawhid Wa'al-Jihad **l)** JTJ **m)** Islamic State of Iraq **n)** ISI **o)** al-Zarqawi network **p)** Islamic State in Iraq and the Levant **F.k.a.:** na **Address:** na **Listed on:** 18 Oct. 2004 (amended on 2 Dec. 2004, 5 Mar. 2009, 13 Dec. 2011, 30 May 2013, 14 May 2014, 2 Jun. 2014, 24 Nov. 2020) **Other information:** Review pursuant to Security Council resolution 1822 (2008) was concluded on 25 May 2010. Review pursuant to Security Council resolution 2368 (2017) was concluded on 24 November 2020. INTERPOL-UN Security Council Special Notice web link: https://www.interpol.int/en/How-we-work/Notices/View-UN-Notices-Entities click here

QDe.129 Name: AL-QAIDA IN THE ARABIAN PENINSULA (AQAP)
Name (original script): القاعدة في جزيرة العرب
A.k.a.: a) Al-Qaida of Jihad Organization in the Arabian Peninsula **b)** Tanzim Qa'idat al-Jihad fi Jazirat al-Arab **c)** Al-Qaida Organization in the Arabian Peninsula (AQAP) **d)** Al-Qaida in the South Arabian Peninsula **e)** Ansar al-Shari'a (AAS) **F.k.a.:** Al-Qaida in Yemen (AQY) **Address:** na **Listed on:** 19 Jan. 2010 (amended on 4 Oct. 2012, 15 Jun. 2015, 24 Jun. 2016) **Other information:** AQAP is a regional affiliate of Al-Qaida (QDe.004) and an armed group operating primarily in Arabian Peninsula. Location: Yemen. Alternative location: Saudi Arabia (2004 – 2006). Formed in Jan. 2009 when Al-Qaida in Yemen combined with Saudi Arabian Al-Qaida operatives. Leader of AQAP is Qasim Mohamed Mahdi Al-Rimi (QDi.282). Ansar al-Shari'a was formed in early 2011 by AQAP and has taken responsibility for multiple attacks in Yemen against both government and civilian targets. Review pursuant to Security Council resolution 2368 (2017) was concluded on 15 November 2021. INTERPOL-UN Security Council Special Notice web link: https://www.interpol.int/en/How-we-work/Notices/View-UN-Notices-Entities click here

QDe.146 Name: ANSAR AL CHARIA BENGHAZI
Name (original script): أنصار الشريعة - بنغازي
A.k.a.: a) نصار الشريعة (Ansar al Charia) **b)** Ansar al-Charia **c)** Ansar al-Sharia **d)** Ansar al-Charia Benghazi **e)** Ansar al-Sharia Benghazi **f)** أنصار الشريعة بليبيا (Ansar al Charia in Libya (ASL)) **g)** كتيبة أنصار الشريعة (Katibat Ansar al Charia) **h)** Ansar al Sharia **F.k.a.:** na **Address: a)** (Operates in Benghazi, Libya) **b)** (Support network in Tunisia) **Listed on:** 19 Nov. 2014 (amended on 6 Dec. 2019) **Other information:** Associated with the Organization of Al-Qaida in the Islamic Maghreb (QDe.014), Al Mourabitoun (QDe.141), Ansar al-Shari'a in Tunisia (AAS-T) (QDe.143), and Ansar al Charia Derna (QDe.145). The leader is Mohamed al-Zahawi (not listed). Runs training camps for foreign terrorist fighters travelling to Syria, Iraq and Mali. Review pursuant to Security Council resolution 2368 (2017) was concluded on 4 Dec. 2019 INTERPOL-UN Security Council Special Notice web link: https://www.interpol.int/en/How-we-work/Notices/View-UN-Notices-Entities click here

QDe.145 Name: ANSAR AL CHARIA DERNA
Name (original script): أنصار الشريعة – درنة
A.k.a.: a) Ansar al-Charia Derna **b)** Ansar al-Sharia Derna **c)** أنصار الشريعة (Ansar al Charia) **d)** Ansar al-Sharia **e)** Ansar al Sharia **F.k.a.:** na **Address: a)** (Operates in Derna and Jebel Akhdar, Libya) **b)** (Support network in Tunisia) **Listed on:** 19 Nov. 2014 (amended on 6 Dec. 2019) **Other information:** Associated with the Organization of Al-Qaida in the Islamic Maghreb (QDe.014), Ansar al-Shari'a in Tunisia (AAS-T) (QDe.143) and Ansar al Charia Benghazi (QDe.146). Runs training camps for foreign terrorist fighters travelling to Syria and Iraq. Review pursuant to Security Council resolution 2368 (2017) was concluded on 4 Dec. 2019 INTERPOL-UN Security Council Special Notice web link: https://www.interpol.int/en/How-we-work/Notices/View-UN-Notices-Entities click here

QDe.098 Name: ANSAR AL-ISLAM
Name (original script): أنصار الاسلام
A.k.a.: a) Devotees of Islam **b)** Jund al-Islam **c)** Soldiers of Islam **d)** Kurdistan Supporters of Islam **e)** Supporters of Islam in Kurdistan **f)** Followers of Islam in Kurdistan **g)** Kurdish Taliban **h)** Soldiers of God **i)** Ansar al-Sunna Army **j)** Jaish Ansar al-Sunna **k)** Ansar al-Sunna **F.k.a.:** na **Address:** na **Listed on:** 24 Feb. 2003 (amended on 31 Mar. 2004, 5 Mar. 2009, 18 Mar. 2009, 21 Oct. 2010, 13 Dec. 2011, 24 Nov. 2020) **Other information:** The founder is Najmuddin Faraj Ahmad (QDi.226). Associated with Al-Qaida in Iraq (QDe.115). Located and primarily active in northern Iraq but maintains a presence in western and central Iraq. Review pursuant to Security Council resolution 1822 (2008) was concluded on 21 Jun. 2010. Review pursuant to Security Council resolution 2368 (2017) was concluded on 24 November 2020. INTERPOL-UN Security Council Special Notice web link: https://www.interpol.int/en/How-we-work/Notices/View-UN-Notices-Entities click here

QDe.143 Name: ANSAR AL-SHARI' A IN TUNISIA (AAS-T)
A.k.a.: a) Ansar al-Sharia in Tunisia **b)** Ansar al-Shari' ah in Tunisia **c)** Ansar al-Shari' ah **d)** Ansar al-Sharia **e)** Supporters of Islamic Law **f)** Al-Qayrawan Media Foundation **F.k.a.:** na **Address:** Tunisia **Listed on:** 23 Sep. 2014 (amended on 6 Dec. 2019) **Other information:** A Tunisian armed group with links to the Organization of Al-Qaida in the Islamic Maghreb (QDe.014). The leader is Seifallah ben Hassine (QDi.333). Review pursuant to Security Council resolution 2368 (2017) was concluded on 4 Dec. 2019 INTERPOL-UN Security Council Special Notice web link: https://www.interpol.int/en/How-we-work/Notices/View-UN-Notices-Entities click here

QDe.135 Name: ANSAR EDDINE
Name (original script): انصار الدين
A.k.a.: Ansar Dine **F.k.a.:** na **Address:** Mali **Listed on:** 20 Mar. 2013 (amended on 1 May 2019) **Other information:** Was founded in December 2011 by Iyad ag Ghali (QDi.316). Linked to the Organization of Al-Qaida in the Islamic Maghreb (QDe.014) and Mouvement pour l' Unification et le Jihad en Afrique de l' Ouest (MUJAO) (QDe.134). Associated with Abdelmalek Droukdel (QDi.232). Review pursuant to Security Council resolution 2253 (2015) was concluded on 21 Feb. 2019. Review pursuant to Security Council resolution 2610 (2021) was concluded on 8 November 2022. INTERPOL-UN Security Council Special Notice web link: https://www.interpol.int/en/How-we-work/Notices/View-UN-Notices-Entities click here

QDe.142 Name: ANSARUL MUSLIMINA FI BILADIS SUDAN
Name (original script): أنصار المسلمين في بلاد السودان
A.k.a.: a) Ansaru **b)** Jama'atu Ansaril Muslimina fi Biladis Sudan (JAMBS) **c)** Jama' atu Ansarul Muslimina fi Biladis-Sudan (JAMBS) **d)** Jamma' atu Ansarul Muslimina fi Biladis-Sudan (JAMBS) **e)** Vanguards for the Protection of Muslims in Black Africa **f)** Vanguard for the Protection of Muslims in Black Africa **F.k.a.:** na **Address:** Nigeria **Listed on:** 26 Jun. 2014 (amended on 6 Dec. 2019, 18 Mar. 2020) **Other information:** Terrorist and paramilitary group established in 2012 and operating in Nigeria. Associated with the Organization of Al-Qaida in the Islamic Maghreb (AQIM) (QDe.014), Jama'atu Ahlis Sunna Lidda'Awati Wal-Jihad (Boko Haram) (QDe.138) and Abubakar Mohammed Shekau (QDi322). Review pursuant to Security Council resolution 2368 (2017) was concluded on 4 Dec. 2019 INTERPOL-UN Security Council Special Notice web link: https://www.interpol.int/en/How-we-work/Notices/View-UN-Notices-Entities click here

QDe.006 Name: ARMED ISLAMIC GROUP
Name (original script): الجماعة الاسلامية المسلحة
A.k.a.: a) Al Jamm' ah Al-Islamiah Al- Musallah **b)** GIA **c)** Groupe Islamique Armé **F.k.a.:** na **Address:** Algeria **Listed on:** 6 Oct. 2001 (amended on 7 Apr. 2008, 13 Dec. 2011, 24 Nov. 2020) **Other information:** Review pursuant to Security Council resolution 1822 (2008) was concluded on 21 Jun. 2010. Review pursuant to Security Council resolution 2368 (2017) was concluded on 24

November 2020. INTERPOL-UN Security Council Special Notice web link: https://www.interpol.int/en/How-we-work/Notices/View-UN-Notices-Entities click here

QDe.007 Name: ASBAT AL-ANSAR
Name (original script): عصبة الأنصار
A.k.a.: na **F.k.a.:** na **Address:** Ein el-Hilweh camp, Lebanon **Listed on:** 6 Oct. 2001 (amended on 30 Jan. 2009, 13 Dec. 2011, 24 Nov. 2020) **Other information:** Active in northern Iraq. Associated with Al-Qaida in Iraq (QDe.115). Review pursuant to Security Council resolution 1822 (2008) was concluded on 21 Jun. 2010. Review pursuant to Security Council resolution 2368 (2017) was concluded on 24 November 2020. INTERPOL-UN Security Council Special Notice web link: https://www.interpol.int/en/How-we-work/Notices/View-UN-Notices-Entities click here

QDe.093 Name: BENEVOLENCE INTERNATIONAL FOUNDATION
A.k.a.: a) Al Bir Al Dawalia **b)** BIF **c)** BIF-USA **d)** Mezhdunarodnyj Blagotvoritel'nyl Fond **F.k.a.:** na **Address: a)** 8820 Mobile Avenue, IA, Oak Lawn, Illinois, 60453, United States of America **b)** P.O. Box 548, Worth, Illinois, 60482, United States of America **c)** (Formerly located at) 9838 S. Roberts Road, Suite 1W, Palos Hills, Illinois, 60465, United States of America **d)** (Formerly located at) 20-24 Branford Place, Suite 705, Newark, New Jersey, 07102, United States of America **e)** P.O. Box 1937, Khartoum, Sudan **f)** Bangladesh **g)** (Gaza Strip) **h)** Yemen **Listed on:** 21 Nov. 2002 (amended on 24 Jan. 2003, 28 Apr. 2011, 18 May 2012, 6 Dec. 2019) **Other information:** Employer Identification Number (United States of America): 36-3823186. Review pursuant to Security Council resolution 1822 (2008) was concluded on 22 Jun. 2010. Review pursuant to Security Council resolution 2368 (2017) was concluded on 4 Dec. 2019. INTERPOL-UN Security Council Special Notice web link: https://www.interpol.int/en/How-we-work/Notices/View-UN-Notices-Entities click here

QDe.088 Name: EASTERN TURKISTAN ISLAMIC MOVEMENT (ETIM)
A.k.a.: a) The Eastern Turkistan Islamic Party **b)** The Eastern Turkistan Islamic Party of Allah **c)** Islamic Party of Turkestan **d)** Djamaat Turkistan **F.k.a.:** na **Address:** na **Listed on:** 11 Sep. 2002 (amended on 3 Oct. 2008, 13 Dec. 2011, 24 Nov. 2020) **Other information:** Active in China, South Asia and Central Asia. Review pursuant to Security Council resolution 1822 (2008) was concluded on 20 May 2010. Review pursuant to Security Council resolution 2368 (2017) was concluded on 24 November 2020. INTERPOL-UN Security Council Special Notice web link: https://www.interpol.int/en/How-we-work/Notices/View-UN-Notices-Entities click here

QDe.003 Name: EGYPTIAN ISLAMIC JIHAD
Name (original script): الجهاد الاسلامي المصري
A.k.a.: a) Egyptian Al-Jihad **b)** Jihad Group **c)** New Jihad **d)** Al-Jihad **e)** Egyptian Islamic Movement **F.k.a.:** na **Address:** na **Listed on:** 6 Oct. 2001 (amended on 5 Mar. 2009, 13 Dec. 2011, 24 Nov. 2020) **Other information:** Co-founded by Aiman Muhammed Rabi al-Zawahiri (QDi.006), who was also its military leader. Review pursuant to Security Council resolution 1822 (2008) was concluded on 21 Jun. 2010. Review pursuant to Security Council resolution 2368 (2017) was concluded on 24 November 2020. Review pursuant to Security Council resolution 2610 (2021) was concluded on 8 November 2022. INTERPOL-UN Security Council Special Notice web link: https://www.interpol.int/en/How-we-work/Notices/View-UN-Notices-Entities click here

QDe.131 Name: EMARAT KAVKAZ
Name (original script): Эмират Кавказ
A.k.a.: na **F.k.a.:** na **Address:** na **Listed on:** 29 Jul. 2011 (amended on 24 Nov. 2020) **Other information:** Mainly active in the Russian Federation, Afghanistan and Pakistan. Led by Doku Khamatovich Umarov (QDi.290). Review pursuant to Security Council resolution 2368 (2017) was concluded on 24 November 2020. INTERPOL-UN Security Council Special Notice web link: https://www.interpol.int/en/How-we-work/Notices/View-UN-Notices-Entities click here

QDe.091 Name: GLOBAL RELIEF FOUNDATION (GRF)

A.k.a.: na **F.k.a.:** na **Address:** **a)** 9935 South 76th Avenue, Unit 1, Bridgeview, Illinois, 60455, United States of America **b)** P.O. Box 1406, Bridgeview, Illinois, 60455, United States of America **Listed on:** 22 Oct. 2002 (amended on 26 Nov. 2004, 20 Dec. 2005, 25 Jul. 2006, 24 Mar. 2009, 11 Mar. 2010, 25 Mar. 2010, 28 Apr. 2011, 21 Feb. 2012, 14 Feb. 2014, 6 Dec. 2019) **Other information:** Other Foreign Locations: Afghanistan, Bangladesh, Eritrea, Ethiopia, India, Iraq, West Bank and Gaza, Somalia and Syria. Federal Employer Identification Number (United States of America): 36-3804626. Review pursuant to Security Council resolution 1822 (2008) was concluded on 21 Jun. 2010. Review pursuant to Security Council resolution 2368 (2017) was concluded on 4 Dec. 2019. INTERPOL-UN Security Council Special Notice web link: https://www.interpol.int/en/How-we-work/Notices/View-UN-Notices-Entities click here

QDe.153 Name: HANIFA MONEY EXCHANGE OFFICE (BRANCH LOCATED IN ALBU KAMAL, SYRIAN ARAB REPUBLIC)
Name (original script): مكتب حنيفة للصرافة
A.k.a.: **a)** Hanifah Currency Exchange **b)** Hanifeh Exchange **c)** Hanifa Exchange **d)** Hunaifa Office **e)** Hanifah Exchange Company **f)** Hanifa Money Exchange Office **F.k.a.:** na **Address:** Albu Kamal (Al-Bukamal), Syrian Arab Republic **Listed on:** 20 Jul. 2017 **Other information:** Money exchange business in Albu Kamal (Al-Bukamal), Syrian Arab Republic, facilitating the movement of funds on behalf of Islamic State in Iraq and the Levant (ISIL), listed as Al-Qaida in Iraq (QDe.115). Used exclusively for ISIL-related transactions. Review pursuant to Security Council resolution 2610 (2021) was concluded on 8 November 2022. INTERPOL-UN Security Council Special Notice web link: https://www.interpol.int/en/How-we-work/Notices/View-UN-Notices-Entities click here

QDe.149 Name: HARAKAT SHAM AL-ISLAM
A.k.a.: **a)** Haraket Sham al-Islam **b)** Sham al-Islam **c)** Sham al-Islam Movement **F.k.a.:** na **Address:** Syrian Arab Republic **Listed on:** 29 Feb. 2016 **Other information:** Moroccan-led terrorist organization formed in Aug. 2013 and operating in Syrian Arab Republic. Principally composed of foreign terrorist fighters and associated with Al-Nusrah Front for the People of the Levant (QDe.137). INTERPOL-UN Security Council Special Notice web link: https://www.interpol.int/en/How-we-work/Notices/View-UN-Notices-Entities click here

QDe.008 Name: HARAKAT UL-MUJAHIDIN / HUM
A.k.a.: **a)** Al-Faran **b)** Al-Hadid **c)** Al-Hadith **d)** Harakat Ul-Ansar **e)** HUA **f)** Harakat Ul-Mujahideen **F.k.a.:** na **Address:** Pakistan **Listed on:** 6 Oct. 2001 (amended on 13 Dec. 2011, 24 Nov. 2020) **Other information:** Associated with Jaish-i-Mohammed (QDe.019), Lashkar i Jhangvi (LJ) (QDe.096) and Lashkar-e-Tayyiba (QDe.118). Active in Pakistan and Afghanistan. Banned in Pakistan. Review pursuant to Security Council resolution 1822 (2008) was concluded on 21 Jun. 2010. Review pursuant to Security Council resolution 2368 (2017) was concluded on 24 November 2020. INTERPOL-UN Security Council Special Notice web link: https://www.interpol.int/en/How-we-work/Notices/View-UN-Notices-Entities click here

QDe.130 Name: HARAKAT-UL JIHAD ISLAMI
Name (original script): حرکت الجہاد الاسلامی
A.k.a.: **a)** HUJI **b)** Movement of Islamic Holy War **c)** Harkat-ul-Jihad-al Islami **d)** Harkat-al-Jihad-ul Islami **e)** Harkat-ul-Jehad-al-Islami **f)** Harakat ul Jihad-e-Islami **F.k.a.:** **a)** Harakat-ul-Ansar **b)** HUA **Address:** na **Listed on:** 6 Aug. 2010 (amended on 13 Dec. 2011, 24 Nov. 2020) **Other information:** Was established in Afghanistan in 1980. In 1993, Harakat-ul Jihad Islami merged with Harakat ul-Mujahidin (QDe.008) to form Harakat ul-Ansar. In 1997, Harakat-ul Jihad Islami split from Harakat ul-Ansar and resumed using its former name. Operations are in India, Pakistan and Afghanistan. Banned in Pakistan. Review pursuant to Security Council resolution 2368 (2017) was concluded on 24 November 2020. INTERPOL-UN Security Council Special Notice web link: https://www.interpol.int/en/How-we-work/Notices/View-UN-Notices-Entities click here

QDe.147 Name: HILAL AHMAR SOCIETY INDONESIA (HASI)

A.k.a.: a) Yayasan Hilal Ahmar **b)** Indonesia Hilal Ahmar Society for Syria **F.k.a.:** na **Address:** na **Listed on:** 13 Mar. 2015 (amended on 24 Nov. 2020) **Other information:** Ostensibly humanitarian wing of Jemaah Islamiyah (QDe.092). Operates in Lampung, Jakarta, Semarang, Yogyakarta, Solo, Surabaya and Makassar, Indonesia. Has been recruiting, funding and facilitating travel of foreign terrorist fighters to Syria. Not affiliated with the humanitarian group International Federation of the Red Cross and Red Crescent Societies (IFRC). Review pursuant to Security Council resolution 2368 (2017) was concluded on 24 November 2020. INTERPOL-UN Security Council Special Notice web link: https://www.interpol.int/en/How-we-work/Notices/View-UN-Notices-Entities click here

QDe.009 Name: ISLAMIC ARMY OF ADEN
A.k.a.: na **F.k.a.:** na **Address:** na **Listed on:** 6 Oct. 2001 **Other information:** Review pursuant to Security Council resolution 1822 (2008) was concluded on 9 Jul. 2010. Review pursuant to Security Council resolution 2368 (2017) was concluded on 15 November 2021. INTERPOL-UN Security Council Special Notice web link: https://www.interpol.int/en/How-we-work/Notices/View-UN-Notices-Entities click here

QDe.099 Name: ISLAMIC INTERNATIONAL BRIGADE (IIB)
A.k.a.: a) The Islamic Peacekeeping Brigade **b)** The Islamic Peacekeeping Army **c)** The International Brigade **d)** Islamic Peacekeeping Battalion **e)** International Battalion **f)** Islamic Peacekeeping International Brigade **F.k.a.:** na **Address:** na **Listed on:** 4 Mar. 2003 (amended on 13 Dec. 2011, 6 Dec. 2019) **Other information:** Linked to the Riyadus-Salikhin Reconnaissance and Sabotage Battalion of Chechen Martyrs (RSRSBCM) (QDe.100) and the Special Purpose Islamic Regiment (SPIR) (QDe.101). Review pursuant to Security Council resolution 1822 (2008) was concluded on 17 May 2010. Review pursuant to Security Council resolution 2368 (2017) was concluded on 4 Dec. 2019. Review pursuant to Security Council resolution 2368 (2017) was concluded on 15 November 2021. INTERPOL-UN Security Council Special Notice web link: https://www.interpol.int/en/How-we-work/Notices/View-UN-Notices-Entities click here

QDe.119 Name: ISLAMIC JIHAD GROUP
A.k.a.: a) Jama' at al-Jihad **b)** Libyan Society **c)** Kazakh Jama' at **d)** Jamaat Mojahedin **e)** Jamiyat **f)** Jamiat al-Jihad al-Islami **g)** Dzhamaat Modzhakhedov **h)** Islamic Jihad Group of Uzbekistan **i)** al-Djihad al-Islami **j)** Zamaat Modzhakhedov Tsentralnoy Asii **k)** Islamic Jihad Union **F.k.a.:** na **Address:** na **Listed on:** 1 Jun. 2005 (amended on 19 Apr. 2006, 20 Feb. 2008, 13 Dec. 2011, 24 Nov. 2020) **Other information:** Founded and led by Najmiddin Kamolitdinovich Jalolov (deceased) and Suhayl Fatilloevich Buranov (deceased). Associated with the Islamic Movement of Uzbekistan (QDe.010) and Emarat Kavkaz (QDe.131). Active in the Afghanistan/Pakistan border area, Central Asia, South Asia region and some European States. Review pursuant to Security Council resolution 1822 (2008) was concluded on 20 May 2010. Review pursuant to Security Council resolution 2368 (2017) was concluded on 24 November 2020. INTERPOL-UN Security Council Special Notice web link: https://www.interpol.int/en/How-we-work/Notices/View-UN-Notices-Entities click here

QDe.010 Name: ISLAMIC MOVEMENT OF UZBEKISTAN
A.k.a.: IMU **F.k.a.:** na **Address:** na **Listed on:** 6 Oct. 2001 (amended on 13 Dec. 2011, 24 Nov. 2020) **Other information:** Associated with the Eastern Turkistan Islamic Movement (QDe.088), Islamic Jihad Group (QDe.119) and Emarat Kavkaz (QDe.131). Active in the Afghanistan/Pakistan border area, northern Afghanistan and Central Asia. Review pursuant to Security Council resolution 1822 (2008) was concluded on 21 Jun. 2010. Review pursuant to Security Council resolution 2368 (2017) was concluded on 24 November 2020. INTERPOL-UN Security Council Special Notice web link: https://www.interpol.int/en/How-we-work/Notices/View-UN-Notices-Entities click here

QDe.161 Name: ISLAMIC STATE IN IRAQ AND THE LEVANT - KHORASAN (ISIL-K)
A.k.a.: a) ISIL KHORASAN **b)** ISLAMIC STATE' S KHORASAN PROVINCE **c)** ISIS WILAYAT KHORASAN **d)** ISIL' S SOUTH ASIA BRANCH **e)** SOUTH ASIAN CHAPTER OF ISIL **f)** The Islamic State

of Iraq and ash-Sham—Khorasan Province **g)** The Islamic State of Iraq and Syria—Khorasan **h)** Islamic State of Iraq and Levant in Khorasan Province **i)** Islamic State Khurasan **j)** ISIS-K **k)** ISISK **l)** IS-Khorasan **F.k.a.:** na **Address:** na **Listed on:** 14 May 2019 (amended on 1 Apr. 2022) **Other information:** Islamic State of Iraq and the Levant - Khorasan (ISIL - K) was formed on January 10, 2015 by a former Tehrik-e Taliban Pakistan (TTP) (QDe.132) commander and was established by former Taliban faction commanders who swore an oath of allegiance to the Islamic State of Iraq and the Levant (listed as Al-Qaida in Iraq (QDe.115)). ISIL – K has claimed responsibility for numerous attacks in both Afghanistan and Pakistan. INTERPOL-UN Security Council Special Notice web link: https://www.interpol.int/en/How-we-work/Notices/View-UN-Notices-Entities click here

QDe.165 Name: ISLAMIC STATE IN IRAQ AND THE LEVANT - LIBYA
Name (original script): الدولة الإسلامية في العراق والشام - ليبيا
A.k.a.: **a)** Islamic state of Iraq and the Levant in Libya **b)** Wilayat Barqa **c)** Wilayat Fezzan **d)** Wilayat Tripolitania **e)** Wilayat Tarablus **f)** Wilayat Al-Tarablus **F.k.a.:** na **Address:** na **Listed on:** 4 Mar. 2020 **Other information:** Formed in November 2014 upon announcement by Abu Bakr Al-Baghdadi, listed as Ibrahim Awwad Ibrahim Ali Al-Badri Al-Samarrai (QDi.299). Associated with Islamic State in Iraq and the Levant, listed as Al-Qaida in Iraq (QDe.115). INTERPOL-UN Security Council Special Notice web link: https://www.interpol.int/en/How-we-work/Notices/View-UN-Notices-Entities click here

QDe.166 Name: ISLAMIC STATE IN IRAQ AND THE LEVANT - YEMEN
Name (original script): الدولة الإسلامية في العراق والشام - اليمن
A.k.a.: **a)** Islamic State of Iraq and the Levant of Yemen **b)** Islamic State in Yemen **c)** ISIL in Yemen **d)** ISIS in Yemen **e)** Wilayat al-Yemen, Province of Yemen **F.k.a.:** na **Address:** na **Listed on:** 4 Mar. 2020 **Other information:** Formed in November 2014 upon acceptance of oaths of allegiance by Abu Bakr Al-Baghdadi, listed as Ibrahim Awwad Ibrahim Ali Al-Badri Al-Samarrai (QDi.299). Associated with Islamic State in Iraq and the Levant, listed as Al-Qaida in Iraq (QDe.115). INTERPOL-UN Security Council Special Notice web link: https://www.interpol.int/en/How-we-work/Notices/View-UN-Notices-Entities click here

QDe.163 Name: ISLAMIC STATE IN THE GREATER SAHARA (ISGS)
A.k.a.: **a)** Islamic State in Iraq and Syria – Greater Sahara (ISIS-GS) **b)** Islamic State of Iraq and Syria – Greater Sahara (ISIS-GS) **c)** Islamic State of Iraq and the Levant - Greater Sahara (ISIL-GS) **d)** Islamic State of the Greater Sahel **e)** ISIS in the Greater Sahel **f)** ISIS in the Greater Sahara **g)** ISIS in the Islamic Sahel **F.k.a.:** na **Address:** na **Listed on:** 23 Feb. 2020 **Other information:** Formed in May 2015 by Adnan Abu Walid al-Sahraoui (QDi.415). Associated with the Islamic State in Iraq and the Levant (listed as Al-Qaida in Iraq (QDe.115)). Splinter group of Al-Mourabitoun (QDe.141). Committed terrorist attacks in Mali, Niger and Burkina Faso. INTERPOL-UN Security Council Special Notice web link: https://www.interpol.int/en/How-we-work/Notices/View-UN-Notices-Entities click here

QDe.162 Name: ISLAMIC STATE WEST AFRICA PROVINCE (ISWAP)
A.k.a.: **a)** Islamic State in Iraq and the Levant – West Africa (ISIL-WA) **b)** Islamic State of Iraq and Syria – West Africa (ISIS-WA) **c)** Islamic State of Iraq and Syria West Africa Province (ISISWAP) **d)** Islamic State of Iraq and the Levant – West Africa **F.k.a.:** na **Address:** na **Listed on:** 23 Feb. 2020 **Other information:** Associated with the Islamic State in Iraq and the Levant (listed as Al-Qaida in Iraq (QDe.115)). Formed in March 2015 by Abubakar Shekau (QDi.322). Splinter group of Jama'atu Ahlis Sunna Lidda'Awati Wal-Jihad (Boko Haram) (QDe.138). Committed terrorist attacks in Nigeria. INTERPOL-UN Security Council Special Notice web link: https://www.interpol.int/en/How-we-work/Notices/View-UN-Notices-Entities click here

QDe.169 Name: Islamic State In Iraq And the Levant In South-East Asia (ISIL-SEA, ISIL-South East Asia)
A.k.a.: **a)** Islamic State East Asia Division **b)** Dawlatul Islamiyah Waliyatul Mashriq **F.k.a.:**

na **Address:** na **Listed on:** 27 Jan. 2023 **Other information:** Formed in June 2016 upon announcement by now-deceased Isnilon Hapilon (QDi.204). Associated with Islamic State in Iraq and the Levant, listed as Al-Qaida in Iraq (QDe.115). INTERPOL-UN Security Council Special Notice web link: https://www.interpol.int/en/How-we-work/Notices/View-UN-Notices-Entities click here

QDe.019 Name: JAISH-I-MOHAMMED
A.k.a.: Army of Mohammed **F.k.a.:** na **Address:** Pakistan **Listed on:** 17 Oct. 2001 (amended on 13 Dec. 2011, 24 Nov. 2020) **Other information:** Based in Peshawar and Muzaffarabad, Pakistan Associated with Harakat ul-Mujahidin / HUM (QDe.008), Lashkar-e-Tayyiba (QDe.118), Al-Akhtar Trust International (QDe.121), and Harakat-ul Jihad Islami (QDe.130). Banned in Pakistan. Review pursuant to Security Council resolution 1822 (2008) was concluded on 21 Jun. 2010. Review pursuant to Security Council resolution 2368 (2017) was concluded on 24 November 2020. INTERPOL-UN Security Council Special Notice web link: https://www.interpol.int/en/How-we-work/Notices/View-UN-Notices-Entities click here

QDe.020 Name: JAM'YAH TA'AWUN AL-ISLAMIA
A.k.a.: **a)** Society of Islamic Cooperation **b)** Jam'iyat Al Ta'awun Al Islamiyya **c)** Jit **F.k.a.:** na **Address:** Kandahar City, Afghanistan **Listed on:** 17 Oct. 2001 (amended on 13 Dec. 2011) **Other information:** Founded by Usama Mohammad Awad bin Laden (deceased) in 2001. Review pursuant to Security Council resolution 1822 (2008) was concluded on 21 Jun. 2010. Review pursuant to Security Council resolution 2368 (2017) was concluded on 15 November 2021. INTERPOL-UN Security Council Special Notice web link: https://www.interpol.int/en/How-we-work/Notices/View-UN-Notices-Entities click here

QDe.159 Name: JAMA'A NUSRAT UL-ISLAM WA AL-MUSLIMIN (JNIM)
Name (original script): جماعة نصرة الإسلام والمسلمين
A.k.a.: na **F.k.a.:** na **Address:** na **Listed on:** 4 Oct. 2018 **Other information:** Associated with Al-Qaida (QDe.004), the Organization of Al-Qaida in the Islamic Maghreb (QDe.014), Ansar Eddine (QDe.135) and Al-Mourabitoun (QDe.141). Operations in Mali and Burkina Faso. INTERPOL-UN Security Council Special Notice web link: https://www.interpol.int/en/How-we-work/Notices/View-UN-Notices-Entities click here

QDe.138 Name: JAMA'ATU AHLIS SUNNA LIDDA'AWATI WAL-JIHAD
A.k.a.: **a)** Jama'atu Ahlus-Sunnah Lidda'Awati Wal Jihad **b)** Jama'atu Ahlus-Sunna Lidda'Awati Wal Jihad **c)** جماعة أهل السنة للدعوة والجهاد **d)** Boko Haram **e)** Western Education is a Sin **F.k.a.:** na **Address:** Nigeria **Listed on:** 22 May 2014 (amended on 6 Dec. 2019) **Other information:** Affiliate of Al-Qaida (QDe.004), and the Organization of Al-Qaida in the Islamic Maghreb (AQIM) (QDe.014). Associated with Jama'atu Ansarul Muslimina Fi Biladis-Sudan (Ansaru). The leader is Abubakar Shekau. Review pursuant to Security Council resolution 2368 (2017) was concluded on 4 Dec. 2019 INTERPOL-UN Security Council Special Notice web link: https://www.interpol.int/en/How-we-work/Notices/View-UN-Notices-Entities click here

QDe.164 Name: JAMAAH ANSHARUT DAULAH
Name (original script): JAMAAH ANSHARUT DAULAH
A.k.a.: **a)** Jemaah Anshorut Daulah **b)** Jamaah Ansharut Daulat **F.k.a.:** na **Address:** na **Listed on:** 4 Mar. 2020 **Other information:** Established in 2015 as an umbrella group of Indonesian extremist groups that pledged allegiance to then-ISIL leader Abu Bakr al-Baghdadi. Associated with Islamic State in Iraq and the Levant, listed as Al-Qaida in Iraq (QDe.115). INTERPOL-UN Security Council Special Notice web link: https://www.interpol.int/en/How-we-work/Notices/View-UN-Notices-Entities click here

QDe.152 Name: JAMAAT-UL-AHRAR (JuA)
Name (original script): جمات ال احرار
A.k.a.: **a)** Jamaat-e-Ahrar **b)** Tehrik-e Taliban Pakistan Jamaat ul Ahrar **F.k.a.:** Ahrar-ul-

Hind **Address: a)** Lalpura, Nangarhar Province, Afghanistan and Afghanistan-Pakistan border region(since Jun. 2015) **b)** Mohmand Agency, Pakistan (as at Aug. 2014) **Listed on:** 6 Jul. 2017 **Other information:** Splinter group of the Tehrik-e Taliban Pakistan (QDe.132). Associated with Islamic State in Iraq and the Levant, listed as Al-Qaida in Iraq (QDe.115). Formed in Aug. 2014 in Mohmand Agency, Pakistan. Operates from Nangarhar Province, Afghanistan and Pakistan-Afghanistan border region. Banned in Pakistan on 21 Nov. 2016. Review pursuant to Security Council resolution 2610 (2021) was concluded on 8 November 2022. INTERPOL-UN Security Council Special Notice web link: https://www.interpol.int/en/How-we-work/Notices/View-UN-Notices-Entities click here

QDe.092 Name: JEMAAH ISLAMIYAH
A.k.a.: a) Jema' ah Islamiyah **b)** Jemaah Islamiya **c)** Jemaah Islamiah **d)** Jamaah Islamiyah **e)** Jama' ah Islamiyah **F.k.a.:** na **Address:** na **Listed on:** 25 Oct. 2002 (amended on 13 Dec. 2011, 24 Nov. 2020) **Other information:** Operates in Southeast Asia, including Indonesia, Malaysia and the Philippines. Associated with the Abu Sayyaf Group (QDe.001). Review pursuant to Security Council resolution 1822 (2008) was concluded on 25 May 2010. Review pursuant to Security Council resolution 2368 (2017) was concluded on 24 November 2020. INTERPOL-UN Security Council Special Notice web link: https://www.interpol.int/en/How-we-work/Notices/View-UN-Notices-Entities click here

QDe.133 Name: JEMMAH ANSHORUT TAUHID (JAT)
A.k.a.: a) Jemaah Anshorut Tauhid **b)** Jemmah Ansharut Tauhid **c)** Jem' mah Ansharut Tauhid **d)** Jamaah Ansharut Tauhid **e)** Jama' ah Ansharut Tauhid **f)** Laskar 99 **F.k.a.:** na **Address:** Jl. Semenromo number 58, 04/XV Ngruki, Cemani, Grogol, Sukoharjo, Jawa Tengah, Indonesia (Telephone: 0271-2167285, Email: info@ansharuttauhid.com) **Listed on:** 12 Mar. 2012 (amended on 17 Jul. 2018) **Other information:** A group affiliated with the Islamic State in Iraq and the Levant (ISIL), listed as Al-Qaida in Iraq (QDe.115), that has perpetrated attacks in Indonesia. Founded and led by Abu Bakar Ba'asyir (QDi.217). Established on 27 Jul. 2008 in Solo, Indonesia. Had been associated with Jemmah Islamiya (JI) (QDe.092). Review pursuant to Security Council resolution 2253 (2015) was concluded on 7 June 2018. Website: http://ansharuttauhid.com/ Review pursuant to Security Council resolution 2368 (2017) was concluded on 15 November 2021. INTERPOL-UN Security Council Special Notice web link: https://www.interpol.int/en/How-we-work/Notices/View-UN-Notices-Entities click here

QDe.156 Name: JUND AL AQSA
A.k.a.: a) The Soldiers of Aqsa **b)** Soldiers of Aqsa **c)** Sarayat Al Quds **F.k.a.:** na **Address: a)** Idlib Governorate, Syrian Arab Republic **b)** Hama Governorate, Syrian Arab Republic **Listed on:** 20 Jul. 2017 **Other information:** Associated with the Al Nusrah Front for the People of the Levant (QDe.137). Review pursuant to Security Council resolution 2610 (2021) was concluded on 8 November 2022. INTERPOL-UN Security Council Special Notice web link: https://www.interpol.int/en/How-we-work/Notices/View-UN-Notices-Entities click here

QDe.151 Name: JUND AL-KHILAFAH IN ALGERIA (JAK-A)
A.k.a.: a) Jund al Khalifa **b)** Jund al-Khilafah fi Ard al-Jaza' ir **c)** Jund al-Khalifa fi Ard al-Jazayer **d)** Soldiers of the Caliphate in Algeria **e)** Soldiers of the Caliphate of Algeria **f)** Soldiers of the Caliphate in the Land of Algeria **F.k.a.:** na **Address:** Kabylie region, Algeria **Listed on:** 29 Sep. 2015 (amended on 24 Nov. 2020) **Other information:** Emerged on 13 Sep. 2014. Most known for its abduction and subsequent beheading of French national Herve Gourdel. Claimed responsibility for attacking police and gendarmes in Algeria and continued planning future attacks. Review pursuant to Security Council resolution 2368 (2017) was concluded on 24 November 2020. INTERPOL-UN Security Council Special Notice web link: https://www.interpol.int/en/How-we-work/Notices/View-UN-Notices-Entities click here

QDe.167 Name: JUND AL-KHILAFAH IN TUNISIA (JAK-T)

Name (original script): جند الخلافة في تونس
A.k.a.: a) ISIL-Tunisia **b)** ISIL-Tunisia Province **c)** Soldiers of the Caliphate **d)** Jund al-Khilafa **e)** Jund al Khilafah **f)** Jund al-Khilafah fi Tunis **g)** Soldiers of the Caliphate in Tunisia **h)** Tala I Jund al-Khilafah **i)** Vanguards of the Soldiers of the Caliphate **j)** Daesh Tunisia **k)** Ajnad **F.k.a.:** na **Address:** na **Listed on:** 29 Dec. 2021 **Other information:** Formed in November 2014.Associated with Islamic State in Iraq and the Levant, listed as Al-Qaida in Iraq (QDe.115). INTERPOL-UN Security Council Special Notice web link: https://www.interpol.int/en/How-we-work/Notices/View-UN-Notices-Entities click here

QDe.155 Name: Jaysh Khalid Ibn al Waleed
A.k.a.: a) Khalid ibn al-Walid Army **b)** Liwa Shuhada al-Yarmouk **c)** Harakat al-Muthanna al-Islamia **F.k.a.:** na **Address:** na **Listed on:** 20 Jul. 2017 **Other information:** Joined the Islamic State in Iraq and the Levant (ISIL), listed as Al-Qaida in Iraq (QDe.115), in May 2015. Review pursuant to Security Council resolution 2610 (2021) was concluded on 8 November 2022. INTERPOL-UN Security Council Special Notice web link: https://www.interpol.int/en/How-we-work/Notices/View-UN-Notices-Entities click here

QDe.168 Name: KHATIBA AL-TAWHID WAL-JIHAD (KTJ)
Name (original script): Катиба ат-Таухид валь-Джихад
A.k.a.: a) JANNAT OSHIKLARI **b)** Jama`at al-Tawhid wal-Jihad **F.k.a.:** JANNAT OSHIKLARI **Address:** na **Listed on:** 7 Mar. 2022 **Other information:** Khatiba al-Tawhid wal-Jihad (formerly known as Jannat Oshiklari) is a terrorist organization operating under the umbrella of the international terrorist organization Al-Nusrah Front for the People of the Levant (QDe.137). The group mainly operates in the provinces of Hama, Idlib and Ladhiqiyah, in the Syrian Arab Republic, and also conduct operations in Turkey, Kyrgyzstan, Uzbekistan, Russian Federation, Tajikistan, Kazakhstan, Egypt, Afghanistan, Ukraine. The number of fighters of KTJ is about 500. KTJ also cooperates with such terrorist organizations as Khatiba Imam al-Bukhari (QDe.158) and the Islamic Jihad Group (QDe.119). INTERPOL-UN Security Council Special Notice web link: https://www.interpol.int/en/How-we-work/Notices/View-UN-Notices-Entities click here

QDe.158 Name: KHATIBA IMAM AL-BUKHARI (KIB)
A.k.a.: Khataib al-Imam al-Bukhari **F.k.a.:** na **Address: a)** Afghanistan/Pakistan border area(previous location) **b)** Khan-Shaykhun, Syrian Arab Republic (53 km south of Idlib, location as at Mar. 2018) **c)** Idlib, Aleppo and Khama, Syrian Arab Republic (operation zone) **Listed on:** 29 Mar. 2018 **Other information:** Associated with Al-Nusrah Front for the People of the Levant (QDe.137). Committed terrorist attacks in the Syrian Arab Republic. Since 2016 redeployed to Northern Afghanistan to project attacks against Central Asia countries. INTERPOL-UN Security Council Special Notice web link: https://www.interpol.int/en/How-we-work/Notices/View-UN-Notices-Entities click here

QDe.096 Name: LASHKAR I JHANGVI (LJ)
A.k.a.: na **F.k.a.:** na **Address:** na **Listed on:** 3 Feb. 2003 (amended on 13 Dec. 2011, 20 Nov. 2017) **Other information:** Based primarily in Pakistan' s Punjab region and in the city of Karachi. Active in Pakistan although banned as at 2010. Review pursuant to Security Council resolution 2161 (2014) was concluded on 23 Dec. 2016. Review pursuant to Security Council resolution 2368 (2017) was concluded on 15 November 2021. INTERPOL-UN Security Council Special Notice web link: https://www.interpol.int/en/How-we-work/Notices/View-UN-Notices-Entities click here

QDe.118 Name: LASHKAR-E-TAYYIBA
A.k.a.: a) Lashkar-e-Toiba **b)** Lashkar-i-Taiba **c)** al Mansoorian **d)** al Mansooreen **e)** Army of the Pure **f)** Army of the Righteous **g)** Army of the Pure and Righteous **h)** Paasban-e-Kashmir **i)** Paasban-i-Ahle-Hadith **j)** Pasban-e-Kashmir **k)** Pasban-e-Ahle-Hadith **l)** Paasban-e-Ahle-Hadis **m)** Pashan-e-ahle Hadis **n)** Lashkar e Tayyaba **o)** LET **p)** Jamaat-ud-Dawa **q)** JUD **r)** Jama'at al-Dawa **s)** Jamaat ud-Daawa **t)** Jamaat ul-Dawah **u)** Jamaat-ul-Dawa **v)** Jama'at-i-Dawat **w)** Jamaiat-ud-Dawa

x) Jama'at-ud-Da'awah **y)** Jama'at-ud-Da'awa **z)** Jamaati-ud-Dawa **aa)** Falah-i-Insaniat Foundation (FIF) **F.k.a.:** na **Address:** na **Listed on:** 2 May 2005 (amended on 3 Nov. 2005, 10 Dec. 2008, 14 Mar. 2012, 24 Nov. 2020) **Other information:** Associated with Hafiz Muhammad Saeed (QDi.263) who is the leader of Lashkar-e-Tayyiba. Review pursuant to Security Council resolution 1822 (2008) was concluded on 8 Jun. 2010. Review pursuant to Security Council resolution 2368 (2017) was concluded on 24 November 2020. INTERPOL-UN Security Council Special Notice web link: https://www.interpol.int/en/How-we-work/Notices/View-UN-Notices-Entities click here

QDe.011 Name: LIBYAN ISLAMIC FIGHTING GROUP
Name (original script): الجماعة الاسلامية المقاتلة الليبية
A.k.a.: LIFG **F.k.a.:** na **Address:** Libya **Listed on:** 6 Oct. 2001 (amended on 5 Mar. 2009, 13 Dec. 2011, 24 Nov. 2020) **Other information:** Members in Afghanistan merged with Al-Qaida (QDe.004) in Nov. 2007. Review pursuant to Security Council resolution 1822 (2008) was concluded on 21 Jun. 2010. Review pursuant to Security Council resolution 2368 (2017) was concluded on 24 November 2020. INTERPOL-UN Security Council Special Notice web link: https://www.interpol.int/en/How-we-work/Notices/View-UN-Notices-Entities click here

QDe.012 Name: MAKHTAB AL-KHIDAMAT
Name (original script): مكتب الخدمات
A.k.a.: a) MAK **b)** Al Kifah **F.k.a.:** na **Address:** na **Listed on:** 6 Oct. 2001 (amended on 5 Mar. 2009, 13 Dec. 2011, 24 Nov. 2020) **Other information:** Absorbed into Al-Qaida (QDe.004). Review pursuant to Security Council resolution 1822 (2008) was concluded on 21 Jun. 2010. Review pursuant to Security Council resolution 2368 (2017) was concluded on 24 November 2020. Review pursuant to Security Council resolution 2610 (2021) was concluded on 8 November 2022. INTERPOL-UN Security Council Special Notice web link: https://www.interpol.int/en/How-we-work/Notices/View-UN-Notices-Entities click here

QDe.089 Name: MOROCCAN ISLAMIC COMBATANT GROUP
Name (original script): الجماعة الاسلامية المغربية المقاتلة
A.k.a.: a) Groupe Islamique Combattant Marocain **b)** GICM **F.k.a.:** na **Address:** Morocco **Listed on:** 10 Oct. 2002 (amended on 5 Mar. 2009, 24 Nov. 2020) **Other information:** Associated with the Organization of Al-Qaida in the Islamic Maghreb (QDe.014). Review pursuant to Security Council resolution 1822 (2008) was concluded on 20 May 2010. Review pursuant to Security Council resolution 2368 (2017) was concluded on 24 November 2020. INTERPOL-UN Security Council Special Notice web link: https://www.interpol.int/en/How-we-work/Notices/View-UN-Notices-Entities click here

QDe.134 Name: MOUVEMENT POUR L' UNIFICATION ET LE JIHAD EN AFRIQUE DE L' OUEST (MUJAO)
Name (original script): حركة التوحيد والجهاد في غرب إفريقيا
A.k.a.: na **F.k.a.:** na **Address: a)** Mali **b)** Algeria **Listed on:** 5 Dec. 2012 **Other information:** Associated with The Organization of Al-Qaida in the Islamic Maghreb (QDe.014) and Mokhtar Belmokhtar (QDi.136). Active in the Sahel/Sahara region. Review pursuant to Security Council resolution 2368 (2017) was concluded on 15 November 2021. INTERPOL-UN Security Council Special Notice web link: https://www.interpol.int/en/How-we-work/Notices/View-UN-Notices-Entities click here

QDe.136 Name: MUHAMMAD JAMAL NETWORK (MJN)
Name (original script): شبكة محمد جمال
A.k.a.: a) Muhammad Jamal Group **b)** Jamal Network **c)** Abu Ahmed Group **d)** Al-Qaida in Egypt (AQE) **F.k.a.:** na **Address:** Operates in Egypt, Libya and Mali **Listed on:** 21 Oct. 2013 (amended on 1 May 2019) **Other information:** Terrorist and paramilitary group established by Muhammad Jamal al Kashif (QDi.318) in 2011 and linked to Al-Qaida (QDe.004), Aiman al-Zawahiri (QDi.006), and the leadership of Al-Qaida in the Arabian Peninsula (AQAP) (QDe.129) and the Organization of Al-Qaida

in the Islamic Maghreb (AQIM) (QDe.014). Funded and supported by AQAP. Multiple terrorist training camps in Egypt and Libya. Reportedly acquiring weapons, conducting training and establishing terrorist groups in the Sinai, Egypt. Training suicide bombers, foreign fighters and planning terrorist attacks in Egypt, Libya and elsewhere as of Sep. 2013. MJN members were reported to be involved in the attack on the United States Mission in Benghazi, Libya, on 11 Sep. 2012. Review pursuant to Security Council resolution 2253 (2015) was concluded on 21 Feb. 2019. Review pursuant to Security Council resolution 2610 (2021) was concluded on 8 November 2022. INTERPOL-UN Security Council Special Notice web link: https://www.interpol.int/en/How-we-work/Notices/View-UN-Notices-Entities click here

QDe.150 Name: MUJAHIDIN INDONESIAN TIMUR (MIT)
A.k.a.: a) Mujahidin of Eastern Indonesia **b)** East Indonesia Mujahideen **c)** Mujahidin Indonesia Timor **d)** Mujahidin Indonesia Barat (MIB) **e)** Mujahidin of Western Indonesia **F.k.a.:** na **Address:** Indonesia **Listed on:** 29 Sep. 2015 (amended on 30 Mar. 2017) **Other information:** Terrorist group linked to Islamic State in Iraq and the Levant (ISIL), listed as Al-Qaida in Iraq (QDe.115), Jemaah Islamiyah (JI) (QDe.092), and Jemmah Anshorut Tauhid (JAT) (QDe.133). Operates in Java and Sulawesi, Indonesia and also active in Indonesia’ s eastern provinces. Its former leader was Abu Wardah, a.k.a. Santoso (deceased). Review pursuant to Security Council resolution 2368 (2017) was concluded on 15 November 2021. INTERPOL-UN Security Council Special Notice web link: https://www.interpol.int/en/How-we-work/Notices/View-UN-Notices-Entities click here

QDe.021 Name: RABITA TRUST
A.k.a.: na **F.k.a.:** na **Address: a)** Room 9a, 2nd Floor, Wahdat Road, Education Town, Lahore, Pakistan **b)** Wares Colony, Lahore, Pakistan (at time of listing) **Listed on:** 17 Oct. 2001 (amended on 21 Mar. 2012, 18 Jun. 2015, 24 Nov. 2020) **Other information:** Banned in Pakistan. Review pursuant to Security Council resolution 1822 (2008) was concluded on 21 Jun. 2010. Review pursuant to Security Council resolution 2368 (2017) was concluded on 24 November 2020. INTERPOL-UN Security Council Special Notice web link: https://www.interpol.int/en/How-we-work/Notices/View-UN-Notices-Entities click here

QDe.128 Name: RAJAH SOLAIMAN MOVEMENT
A.k.a.: a) Rajah Solaiman Islamic Movement **b)** Rajah Solaiman Revolutionary Movement **F.k.a.:** na **Address: a)** Barangay Mal-Ong, Anda, Pangasinan Province, Philippines **b)** Sitio Dueg, Barangay Maasin, San Clemente, Tarlac Province, Philippines **c)** Number 50, Purdue Street, Cubao, Quezon City, Philippines **Listed on:** 4 Jun. 2008 (amended on 13 Dec. 2011, 9 May 2018, 6 Dec. 2019) **Other information:** Founded and headed by Hilarion Del Rosario Santos III (QDi.244). Associated with the Abu Sayyaf Group (QDe.001), Jemaah Islamiyah (QDe.092) and Khadafi Abubakar Janjalani (deceased). Review pursuant to Security Council resolution 1822 (2008) was concluded on 13 May 2010. Review pursuant to Security Council resolution 2368 (2017) was concluded on 4 Dec. 2019. INTERPOL-UN Security Council Special Notice web link: https://www.interpol.int/en/How-we-work/Notices/View-UN-Notices-Entities click here

QDe.070 Name: REVIVAL OF ISLAMIC HERITAGE SOCIETY
Name (original script): جمعية احياء التراث الاسلامي
A.k.a.: a) Revival of Islamic Society Heritage on the African Continent **b)** Jamia Ihya ul Turath **c)** RIHS **d)** Jamiat Ihia Al-Turath Al-Islamiya **e)** Al-Furqan Foundation Welfare Trust **f)** Al-Furqan Welfare Foundation **F.k.a.:** na **Address: a)** Pakistan **b)** Afghanistan **Listed on:** 11 Jan. 2002 (amended on 25 Jul. 2006, 5 Mar. 2009, 13 Dec. 2011, 15 Jun. 2015, 24 Nov. 2020) **Other information:** NOTE: Only the Pakistan and Afghanistan offices of this entity are designated. Associated with Abu Bakr al-Jaziri (QDi.058) and Afghan Support Committee (ASC) (QDe.069). Review pursuant to Security Council resolution 1822 (2008) was concluded on 8 Jun. 2010. Review pursuant to Security Council resolution 2368 (2017) was concluded on 24 November 2020. INTERPOL-UN Security Council Special Notice web link: https://www.interpol.int/en/How-we-work/Notices/View-UN-Notices-Entities click here

QDe.100 Name: RIYADUS-SALIKHIN RECONNAISSANCE AND SABOTAGE BATTALION OF CHECHEN MARTYRS (RSRSBCM)
A.k.a.: a) Riyadus-Salikhin Reconnaissance and Sabotage Battalion **b)** Riyadh-as-Saliheen **c)** The Sabotage and Military Surveillance Group of the Riyadh al-Salihin Martyrs **d)** Firqat al-Takhrib wa al-Istitla al-Askariyah li Shuhada Riyadh al-Salihin **e)** Riyadus-Salikhin Reconnaissance and Sabotage battalion of Shahids (martyrs) **F.k.a.:** na **Address:** na **Listed on:** 4 Mar. 2003 (amended on 25 Jul. 2006, 13 Dec. 2011, 24 Nov. 2020) **Other information:** Associated with the Islamic International Brigade (IIB) (QDe.099), the Special Purpose Islamic Regiment (SPIR) (QDe.101) and Emarat Kavkaz (QDe.131). Review pursuant to Security Council resolution 1822 (2008) was concluded on 17 May 2010. Review pursuant to Security Council resolution 2368 (2017) was concluded on 24 November 2020. Review pursuant to Security Council resolution 2610 (2021) was concluded on 8 November 2022. INTERPOL-UN Security Council Special Notice web link: https://www.interpol.int/en/How-we-work/Notices/View-UN-Notices-Entities click here

QDe.154 Name: SELSELAT AL-THAHAB
Name (original script): سلسلة الذهب للصرافة
A.k.a.: a) Silsilet al Thahab **b)** Selselat al Thahab For Money Exchange **c)** Silsilat Money Exchange Company **d)** Silsilah Money Exchange Company **e)** Al Silsilah al Dhahaba **f)** Silsalat al Dhab **F.k.a.:** na **Address: a)** Al-Kadhumi Complex, Al-Harthia, Baghdad, Iraq **b)** Al-Abbas Street, Karbala, Iraq **Listed on:** 20 Jul. 2017 **Other information:** Money exchange business facilitating the movement of funds on behalf of Islamic State in Iraq and the Levant (ISIL), listed as Al-Qaida in Iraq (QDe.115), as of Apr. 2016. Conducted over one hundred financial transfers into ISIL-controlled territory. Review pursuant to Security Council resolution 2610 (2021) was concluded on 8 November 2022. INTERPOL-UN Security Council Special Notice web link: https://www.interpol.int/en/How-we-work/Notices/View-UN-Notices-Entities click here

QDe.101 Name: SPECIAL PURPOSE ISLAMIC REGIMENT (SPIR)
A.k.a.: a) The Islamic Special Purpose Regiment **b)** The al-Jihad-Fisi-Sabililah Special Islamic Regiment **c)** Islamic Regiment of Special Meaning **F.k.a.:** na **Address:** na **Listed on:** 4 Mar. 2003 (amended on 25 Jul. 2006, 13 Dec. 2011, 24 Nov. 2020) **Other information:** Linked to the Islamic International Brigade (IIB) (QDe.099) and the Riyadus-Salikhin Reconnaissance and Sabotage Battalion of Chechen Martyrs (RSRSBCM) (QDe.100). Review pursuant to Security Council resolution 1822 (2008) was concluded on 17 May 2010. Review pursuant to Security Council resolution 2368 (2017) was concluded on 24 November 2020. INTERPOL-UN Security Council Special Notice web link: https://www.interpol.int/en/How-we-work/Notices/View-UN-Notices-Entities click here

QDe.108 Name: TAIBAH INTERNATIONAL-BOSNIA OFFICES
A.k.a.: a) Taibah International Aid Agency **b)** Taibah International Aid Association **c)** Al Taibah, Intl. **d)** Taibah International Aide Association **F.k.a.:** na **Address: a)** 6 Avde Smajlovica Street, Novo Sarajevo, Bosnia and Herzegovina **b)** 26 Tabhanska Street, Visoko, Bosnia and Herzegovina **c)** 3 Velika Cilna Ulica, Visoko, Bosnia and Herzegovina **d)** 26 Tabhanska Street, Visoko, Bosnia and Herzegovina **Listed on:** 11 May 2004 (amended on 24 Mar. 2009, 24 Nov. 2020, 2 Feb. 2023) **Other information:** Reportedly defunct. In 2002-2004, Taibah International – Bosnia offices used premises of the Culture Home in Hadzici, Sarajevo, Bosnia and Herzegovina. The organization was officially registered in Bosnia and Herzegovina as a branch of Taibah International Aid Association under registry number 7. Taibah International – Bosnia offices ceased its work by decision of the Ministry of Justice of the Bosnia and Herzegovina Federation (decision on cessation of operation number 03-05-2-70/03). Review pursuant to Security Council resolution 1822 (2008) was concluded on 21 Jun. 2010. Review pursuant to Security Council resolution 2368 (2017) was concluded on 24 November 2020. INTERPOL-UN Security Council Special Notice web link: https://www.interpol.int/en/How-we-work/Notices/View-UN-Notices-Entities click here

QDe.160 Name: TARIQ GIDAR GROUP (TGG)

Name (original script): طارق گیدڑ گروپ
A.k.a.: a) TEHRIK-E-TALIBAN-TARIQ GIDAR GROUP **b)** TTP-TARIQ GIDAR GROUP **c)** TEHREEK-I-TALIBAN PAKISTAN GEEDAR GROUP **d)** TTP GEEDAR GROUP **e)** TARIQ GEEDAR GROUP **f)** COMMANDER TARIQ AFRIDI GROUP **g)** TARIQ AFRIDI GROUP **h)** TARIQ GIDAR AFRIDI GROUP **i)** THE ASIAN TIGERS **F.k.a.:** na **Address:** (Afghanistan/Pakistan border region) **Listed on:** 22 Mar. 2019 **Other information:** Splinter group of Tehrik-e Taliban Pakistan (TTP) (QDe.132). The group was formed in Darra Adam Khel, Federally Administered Tribal Area (FATA), Pakistan, in 2007. INTERPOL-UN Security Council Special Notice web link: https://www.interpol.int/en/How-we-work/Notices/View-UN-Notices-Entities click here

QDe.132 Name: TEHRIK-E TALIBAN PAKISTAN (TTP)
Name (original script): تحریک طالبان پاکستان
A.k.a.: a) Tehrik-I-Taliban Pakistan **b)** Tehrik-e-Taliban **c)** Pakistani Taliban **d)** Tehreek-e-Taliban **F.k.a.:** na **Address:** na **Listed on:** 29 Jul. 2011 (amended on 15 Jun. 2015, 24 Nov. 2020) **Other information:** Tehrik-e Taliban is based in the tribal areas along the Afghanistan/Pakistan border. Formed in 2007, its leader is Maulana Fazlullah (QDi.352). Review pursuant to Security Council resolution 2368 (2017) was concluded on 24 November 2020. INTERPOL-UN Security Council Special Notice web link: https://www.interpol.int/en/How-we-work/Notices/View-UN-Notices-Entities click here

QDe.148 Name: THE ARMY OF EMIGRANTS AND SUPPORTERS
Name (original script): تنظيم جيش المهاجرين و الأنصار
A.k.a.: a) Battalion of Emigrants and Supporters **b)** Army of Emigrants and Supporters organization **c)** Battalion of Emigrants and Ansar **d)** Jaysh al-Muhajirin wal-Ansar (JAMWA) **F.k.a.:** na **Address:** Jabal Turkuman area, Lattakia Governorate, Syrian Arab Republic **Listed on:** 6 Aug. 2015 (amended on 24 Nov. 2020) **Other information:** Established by foreign terrorist fighters in 2013. Location: Syrian Arab Republic. Affiliated with Islamic State in Iraq and the Levant, listed as Al-Qaida in Iraq (QDe.115) and Al-Nusrah Front for the People of the Levant (QDe.137). Review pursuant to Security Council resolution 2368 (2017) was concluded on 24 November 2020. INTERPOL-UN Security Council Special Notice web link: https://www.interpol.int/en/How-we-work/Notices/View-UN-Notices-Entities click here

QDe.014 Name: THE ORGANIZATION OF AL-QAIDA IN THE ISLAMIC MAGHREB
Name (original script): تنظيم القا عدة ببلاد المغرب الاسلامي
A.k.a.: a) AQIM **b)** Al Qaïda au Maghreb islamique (AQMI) **F.k.a.: a)** Le Groupe Salafiste pour La Prédication et le Combat (GSPC) **b)** Salafist Group For Call and Combat **Address: a)** Algeria **b)** Mali **c)** Mauritania **d)** Morocco **e)** Niger **f)** Tunisia **Listed on:** 6 Oct. 2001 (amended on 26 Apr. 2007, 7 Apr. 2008, 17 Jul. 2009, 13 Dec. 2011, 24 Nov. 2020) **Other information:** Headed by Abdelmalek Droukdel (QDi.232). Zone of operation includes Algeria and parts of Mali, Mauritania, Niger, Tunisia and Morocco. Review pursuant to Security Council resolution 1822 (2008) was concluded on 21 Jun. 2010. Review pursuant to Security Council resolution 2368 (2017) was concluded on 24 November 2020. INTERPOL-UN Security Council Special Notice web link: https://www.interpol.int/en/How-we-work/Notices/View-UN-Notices-Entities click here

QDe.090 Name: TUNISIAN COMBATANT GROUP
Name (original script): الجماعة التونسية المقاتلة
A.k.a.: a) Groupe Combattant Tunisien **b)** Groupe Islamiste Combattant Tunisien **c)** GICT **F.k.a.:** na **Address:** Tunisia **Listed on:** 10 Oct. 2002 (amended on 26 Nov. 2004, 5 Mar. 2009, 13 Dec. 2011, 24 Nov. 2020) **Other information:** Associated with the Organization of Al-Qaida in the Islamic Maghreb (QDe.014). Review pursuant to Security Council resolution 1822 (2008) was concluded on 6 May 2010. Review pursuant to Security Council resolution 2368 (2017) was concluded on 24 November 2020. INTERPOL-UN Security Council Special Notice web link: https://www.interpol.int/en/How-we-work/Notices/View-UN-Notices-Entities click here

QDe.068 Name: UMMAH TAMEER E-NAU (UTN)
A.k.a.: na **F.k.a.:** na **Address: a)** Street 13, Wazir Akbar Khan, Kabul, Afghanistan **b)** Pakistan **Listed on:** 24 Dec. 2001 (amended on 13 Dec. 2011) **Other information:** Its directors included Mahmood Sultan Bashir-Ud-Din (QDi.055), Majeed Abdul Chaudhry (QDi.054) and Mohammed Tufail (QDi.056). Banned in Pakistan. Review pursuant to Security Council resolution 1822 (2008) was concluded on 21 Jun. 2010. Review pursuant to Security Council resolution 2368 (2017) was concluded on 15 November 2021. INTERPOL-UN Security Council Special Notice web link: https://www.interpol.int/en/How-we-work/Notices/View-UN-Notices-Entities click here

QDe.015 Name: WAFA HUMANITARIAN ORGANIZATION
A.k.a.: a) Al Wafa **b)** Al Wafa Organization **c)** Wafa Al-Igatha Al-Islamia **F.k.a.:** na **Address: a)** Jordan House No. 125, Street 54, Phase II Hayatabad, Peshawar, Pakistan (at time of listing) **b)** Kuwait (at time of listing) **c)** United Arab Emirates (at time of listing) **d)** Afghanistan (at time of listing) **Listed on:** 6 Oct. 2001 (amended on 21 Mar. 2012, 6 Dec. 2019, 10 Sep. 2020) **Other information:** Headquarters was in Kandahar, Afghanistan as at 2001. Wafa was a component of Al-Qaida (QDe.004) in 2001. Review pursuant to Security Council resolution 1822 (2008) was concluded on 21 Jun. 2010. Review pursuant to Security Council resolution 2368 (2017) was concluded on 4 Dec. 2019. INTERPOL-UN Security Council Special Notice web link: https://www.interpol.int/en/How-we-work/Notices/View-UN-Notices-Entities click here
