# Supplementary figures and images for: Evolution of the global terrorist organizational cooperation network
Source: PLoS One. 2024 Jan 22;19(1):e0281615. doi: 10.1371/journal.pone.0281615 (PMC10824412; doi:10.1371/journal.pone.0281615)

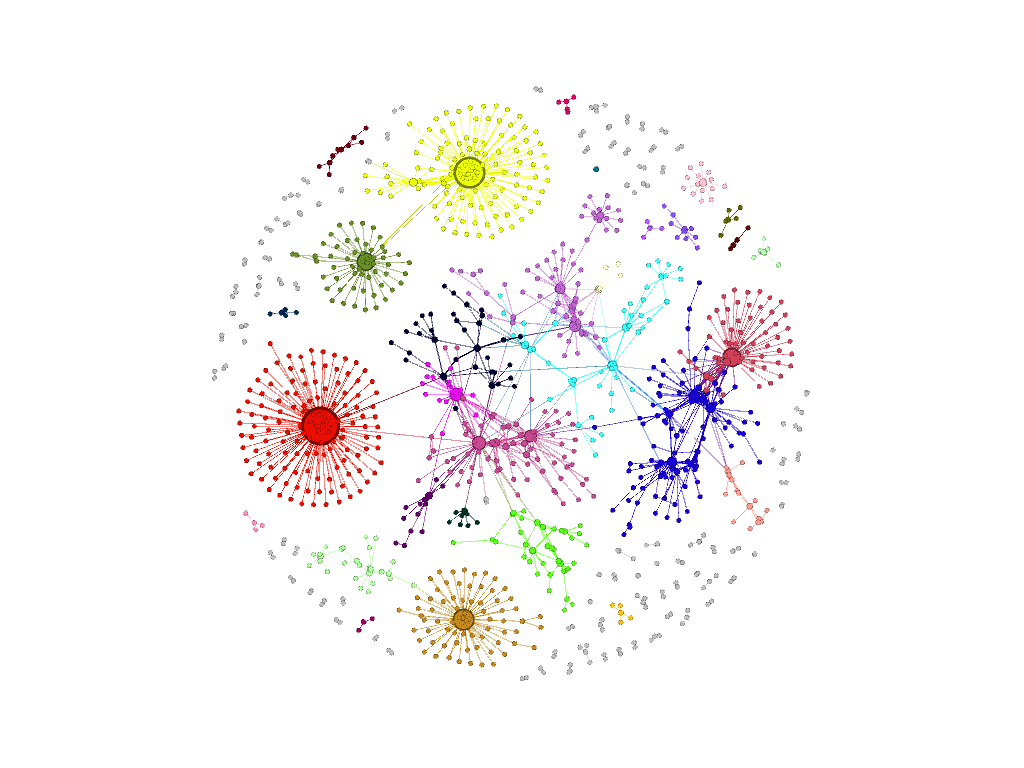

Supplement: S2 Fig — The global terrorist organization cooperation network from 2001 to 2018 was established by using SQL server software to clear the GTD data of individual organization events, collating and retaining the data of multiple organization cooperation, and incorporating the cooperation relationship of the UN sanctions list into the data to be added. (PNG) [file pone.0281615.s002.png]

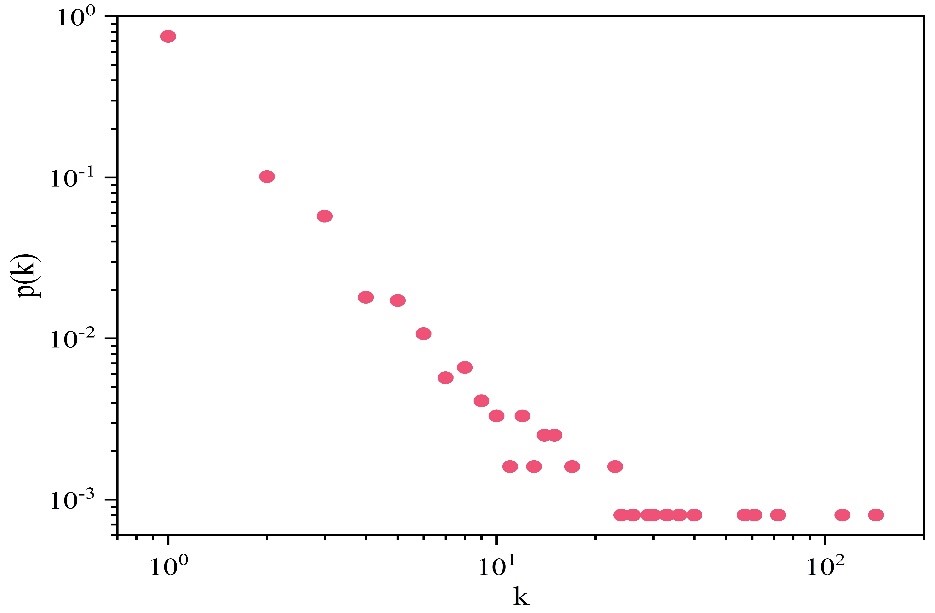

Supplement: S3 Fig — Degree distribution is the statistics and display of the distribution of the number of cooperative relations between terrorist organizations. (JPG) [file pone.0281615.s003.jpg]

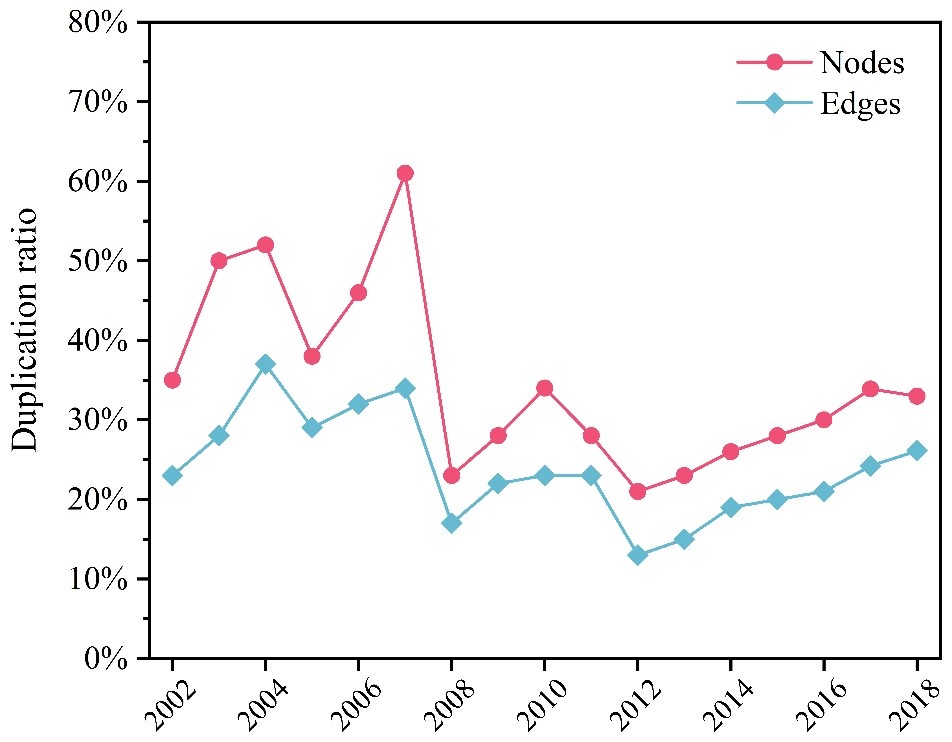

Supplement: S4 Fig — By tallying the number of duplicated nodes and edges in each year and the previous year and calculating the ratio between them and the two years’ nodes and edges, we can determine the proportion of duplicated nodes and edges in the two adjacent years relative to the total number of nodes and edges in the two adjacent years. (JPG) [file pone.0281615.s004.jpg]

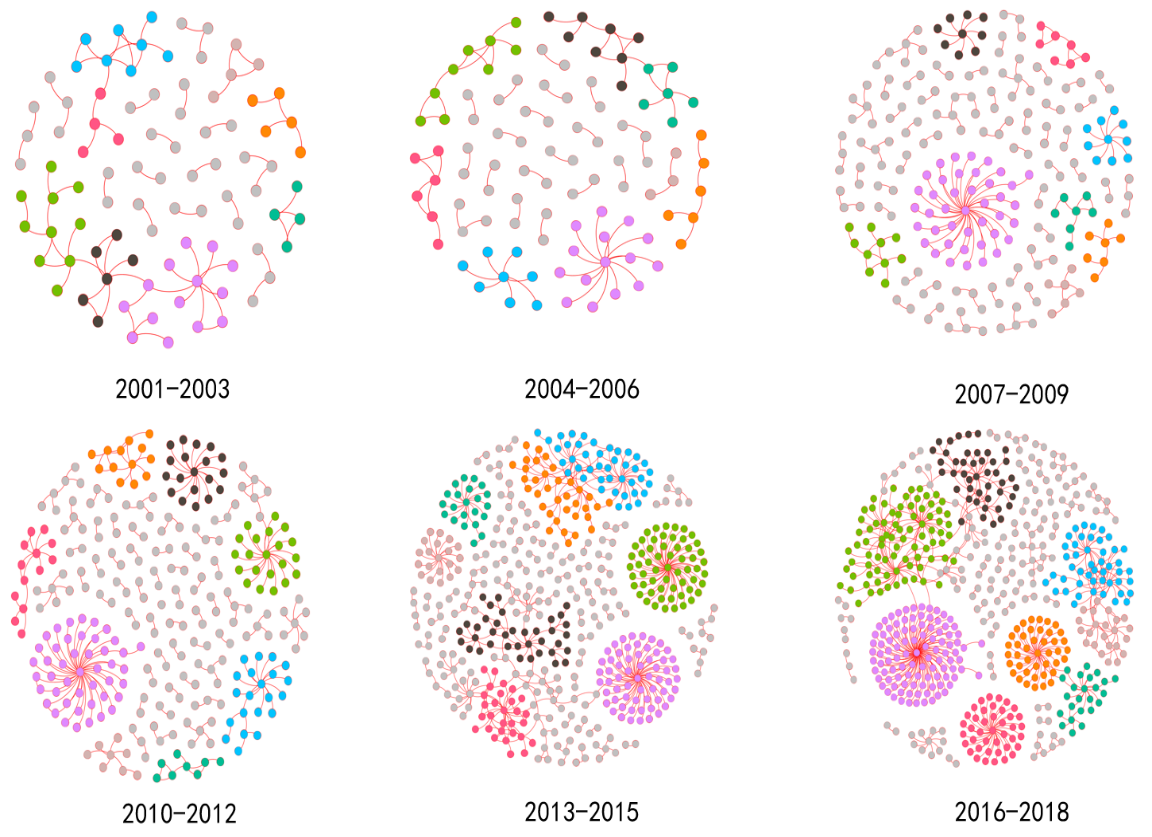

Supplement: S5 Fig — The overall network Is divided into six time slices according to the year in chronological order, and each time slice scale is 3 years. The data is used to visualize and analyze the network topology. (PNG) [file pone.0281615.s005.png]

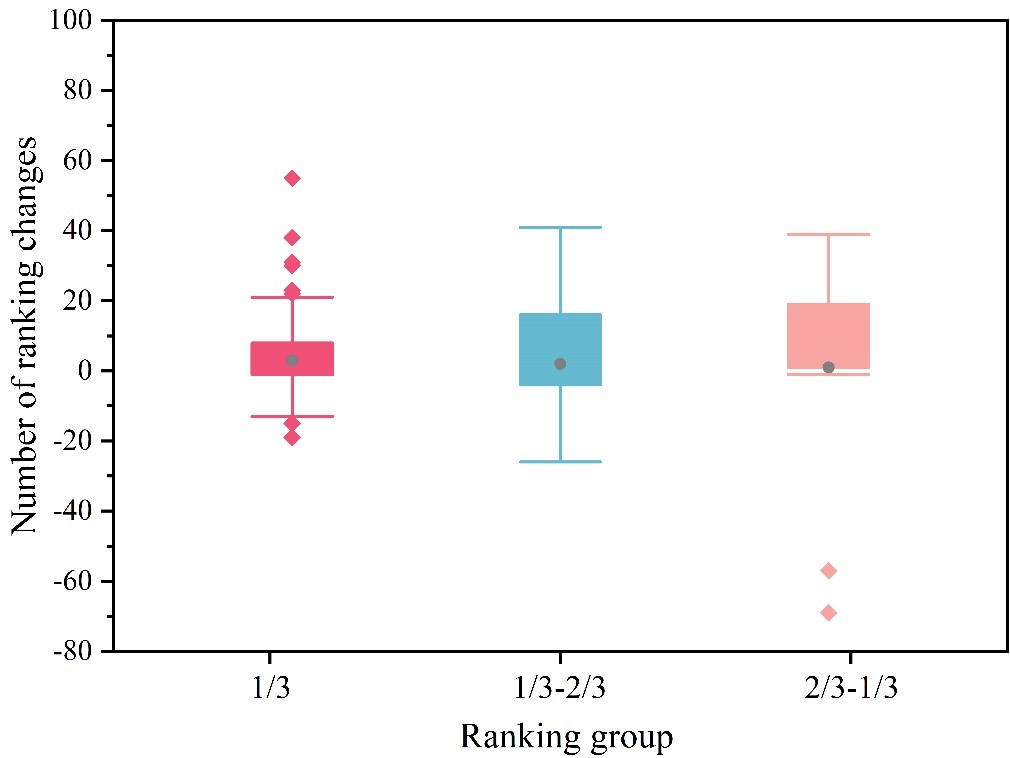

Supplement: S6 Fig — depicts the fluctuation of the ranking change, where the vertical coordinate represents the value of the ranking change and the three portions of the horizontal coordinate correspond to the first 1/3, 1/3-2/3, and last 1/3 of the organization ranking, respectively. (JPG) [file pone.0281615.s006.jpg]

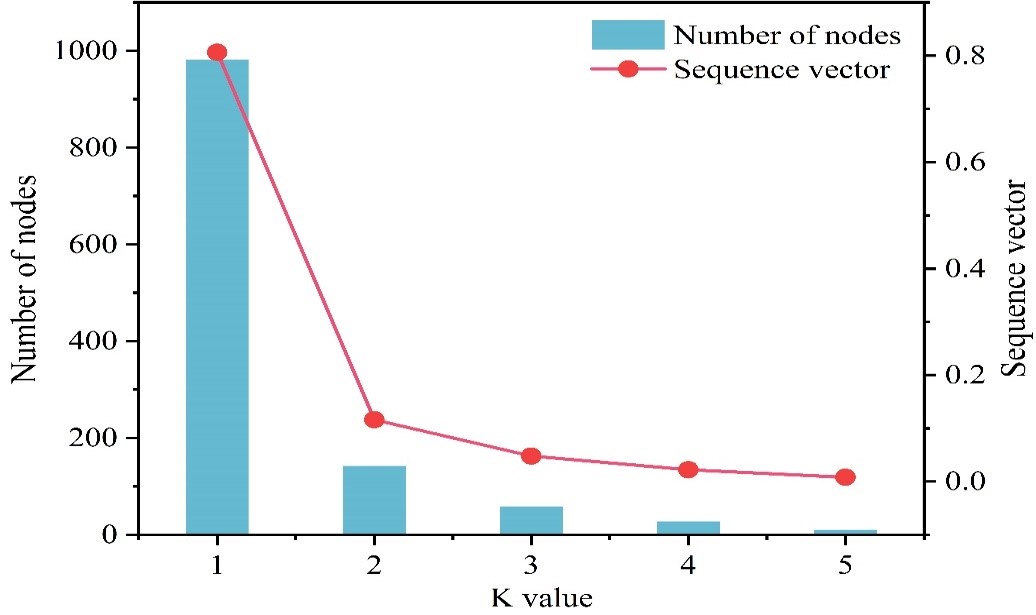

Supplement: S7 Fig — Each layer of the network, as it is clustered from the outside to the interior, may yield a certain number of residual nodes, resulting in a core collapse. When the k value is increased by one, the collapse sequence refers to the number and fraction of vertices lost. The network k-core analysis measured data were manually eliminated, and the number of collapsed nodes formed while increasing k from 1 to 5 was (982, 142, 58, 27, 10) in order, resulting in a kernel collapse sequence of (0.806, 0.116, 0.048, 0.022, 0.008). (JPG) [file pone.0281615.s007.jpg]

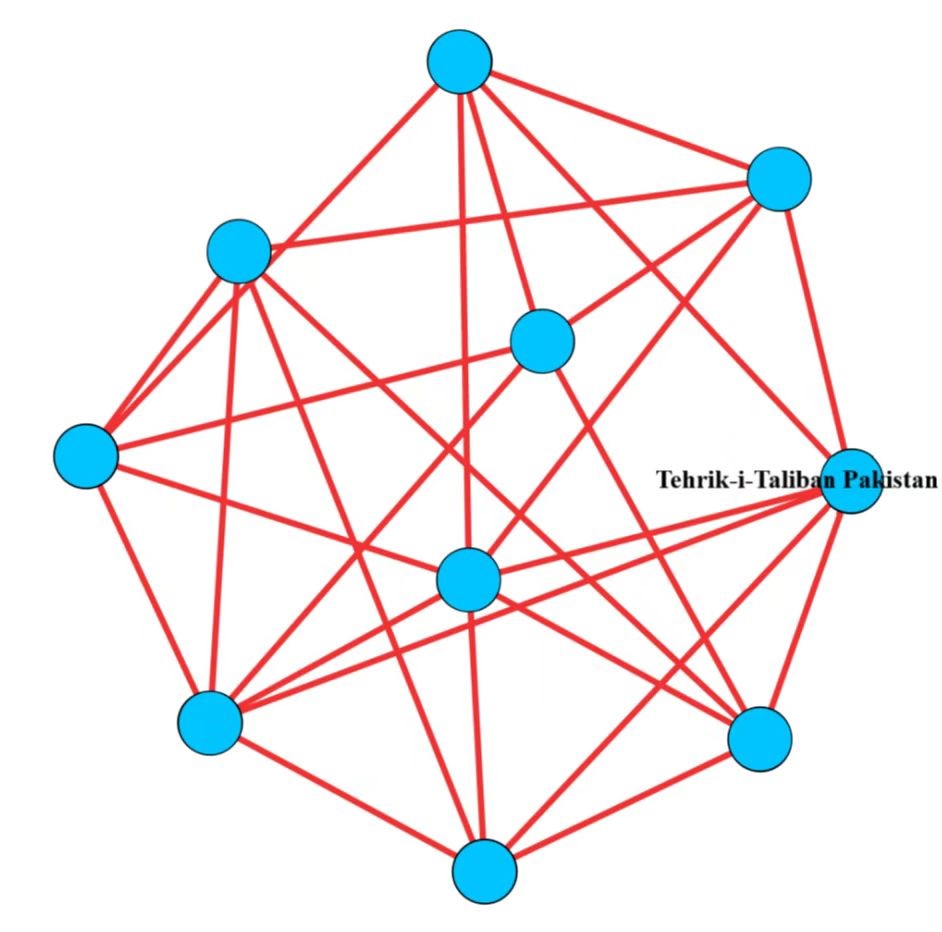

Supplement: S8 Fig — The highest level of the terrorist organization cooperation network is the 5-core group, which contains a total of 10 terrorist organizations, which are closely connected to each other, and each organization has a direct cooperation relationship with at least 5 other organizations in the group. (JPG) [file pone.0281615.s008.jpg]

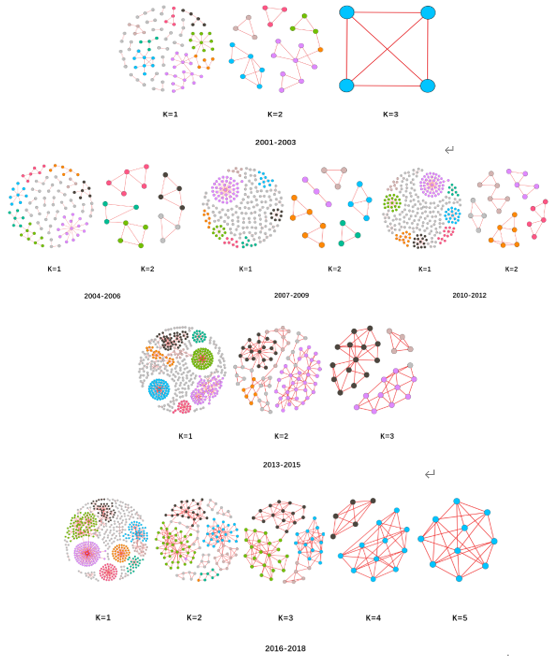

Supplement: S9 Fig — By doing k-core decomposition of the network for six time slices from 2001–2018,the figure visualizes the stochastic core collapse process of the terrorist organization cooperation network. (PNG) [file pone.0281615.s009.png]
